# Dose-dependent spatiotemporal responses of mammalian cells to an alkylating agent

**DOI:** 10.1371/journal.pone.0214512

**Published:** 2019-03-29

**Authors:** Ann Rancourt, Sachiko Sato, Masahiko S. Satoh

**Affiliations:** 1 Laboratory of Glycobiology and Bioimaging, Research Centre for Infectious Diseases, CHUQ, Faculty of Medicine, Laval University, Quebec, Quebec, Canada; 2 Laboratory of DNA Damage Responses and Bioimaging, CHUQ, Faculty of Medicine, Laval University, Quebec, Quebec, Canada; University of South Alabama Mitchell Cancer Institute, UNITED STATES

## Abstract

Cultured cell populations are composed of heterogeneous cells, and previous single-cell lineage tracking analysis of individual HeLa cells provided empirical evidence for significant heterogeneity of the rate of cell proliferation and induction of cell death. Nevertheless, such cell lines have been used for investigations of cellular responses to various substances, resulting in incomplete characterizations. This problem caused by heterogeneity within cell lines could be overcome by investigating the spatiotemporal responses of individual cells to a substance. However, no approach to investigate the responses by analyzing spatiotemporal data is currently available. Thus, this study aimed to analyze the spatiotemporal responses of individual HeLa cells to cytotoxic, sub-cytotoxic, and non-cytotoxic doses of the well-characterized carcinogen, *N*-methyl-*N*'-nitro-*N*-nitrosoguanidine (MNNG). Although cytotoxic doses of MNNG are known to induce cell death, the single-cell tracking approach revealed that cell death occurred following at least four different cellular events, suggesting that cell death is induced via multiple processes. We also found that HeLa cells exposed to a sub-cytotoxic dose of MNNG were in a state of equilibrium between cell proliferation and cell death, with cell death again induced through different processes. However, exposure of cells to a non-cytotoxic dose of MNNG promoted growth by reducing the cell doubling time, thus promoting the growth of a sub-population of cells. A single-cell lineage tracking approach could dissect processes leading to cell death in a spatiotemporal manner and the results suggest that spatiotemporal data obtained by tracking individual cells can be used as a new type of bioinformatics data resource that enables the examination of cellular responses to various external substances.

## Introduction

Cellular responses to genotoxic insults have been investigated using various end-point analyses that measure alterations induced in cells at a specific moment in time, and then deduce the likely cellular responses by evaluating the data obtained at different time points. These results may be valid if all the individual cells within a cell population share similar characteristics and the cellular responses are induced in a stochastic manner. However, cell-to-cell heterogeneity has been demonstrated among cultured cells, although no empirical data revealing the spatiotemporal heterogeneity between individual cells was available. We thus previously developed a novel chronological, single-cell lineage tracking analysis that could record cellular events and movements of live cultured cells in a continuous manner using differential interference contrast (DIC) imaging [1]. The resulting live cell imaging videos contain multidimensional information, including cell morphology, the position of individual cells within a cell population, and the types of cellular events occurring in a cell. Single-cell tracking analysis was previously carried out using 16-mm film to make a live-cell movie, and individual cells were then tracked by morphological observation [2]; however, tracking individual cells by that method is laborious. We therefore used computer-controlled microscopes to create live cell imaging videos, and extracted critical information on individual cells by single-cell tracking to create a cell lineage database [1]. Using this database, we empirically showed significant cell-to-cell heterogeneity in cultured HeLa cells and demonstrated that the fates of individual cells were diverse, indicating that the HeLa cell line comprises a highly heterogeneous population of cells [1]. Moreover, some cells had a reproductive ability >20 times higher than that of other cells [1]. The A549 lung carcinoma cell line and mouse embryonic fibroblasts have also been shown to comprise heterogeneous cells [3, 4]. These observations suggest that individual cells within a cell line may not share similar characteristics, and an accurate determination of cellular responses to genotoxic insults thus requires investigation of the spatiotemporal responses of individual cells to the insults. However, no such approach has yet been fully developed.

The alkylating agent *N*-methyl-*N'*-nitro-*N*-nitrosoguanidine (MNNG) is one of the best-characterized carcinogens, mutagens, and DNA-damaging agents. MNNG produces methylated DNA bases, such as *N*^7^-methyl guanine, which can be efficiently repaired by base excision repair [5–9]. Exposure of cells to cytotoxic doses of MNNG activates poly(ADP-ribose) polymerase-1 during the repair process, and the synthesis of poly-ADP ribose polymer from NAD^+^ leads to cell death (CD) due to the depletion of intracellular NAD^+^ or activation of ADP-ribose polymer-induced apoptosis [10–15]. Another MNNG-induced methylated DNA base, *O*^6^-methyl guanine, causes formation of *O*^6^-methyl G:T and *O*^6^-methyl G:C mismatches when the DNA containing *O*^6^-methyl G is replicated [16, 17]. It has been suggested that these mismatches are repaired by mismatch correction [18–20], which has also been suggested to be involved in CD induced by low levels of alkylating agents [21–24]. Clinically relevant doses of alkylating agents, as used for anticancer treatment, also induce this type of CD [25], although various models have been proposed regarding the induction of CD [21–24, 26–32].

In this study, we analyzed the spatiotemporal responses of HeLa cells to exposure to non-cytotoxic, sub-cytotoxic, and cytotoxic doses of MNNG using single-cell lineage tracking analysis, to develop an approach for the characterization of cellular responses in a heterogeneous cell population. Our results and analyses at the single cell level suggest that individual cells respond to MNNG via different processes and their responses to different doses also significantly vary, while the lineage-based analyses revealed some levels of consistency in dose-dependent responses, which had not been previously revealed. Given that classical end-point analyses, which have previously been used to investigate various biological processes, are unable to determine the specific processes whereby cells eventually undergo CD, spatiotemporal data will be essential for conducting accurate investigations of cellular responses to various external substances.

## Results

### Accuracy of analysis using spatiotemporal data and effect of MNNG exposure on cell doubling time

**Fig 1:**
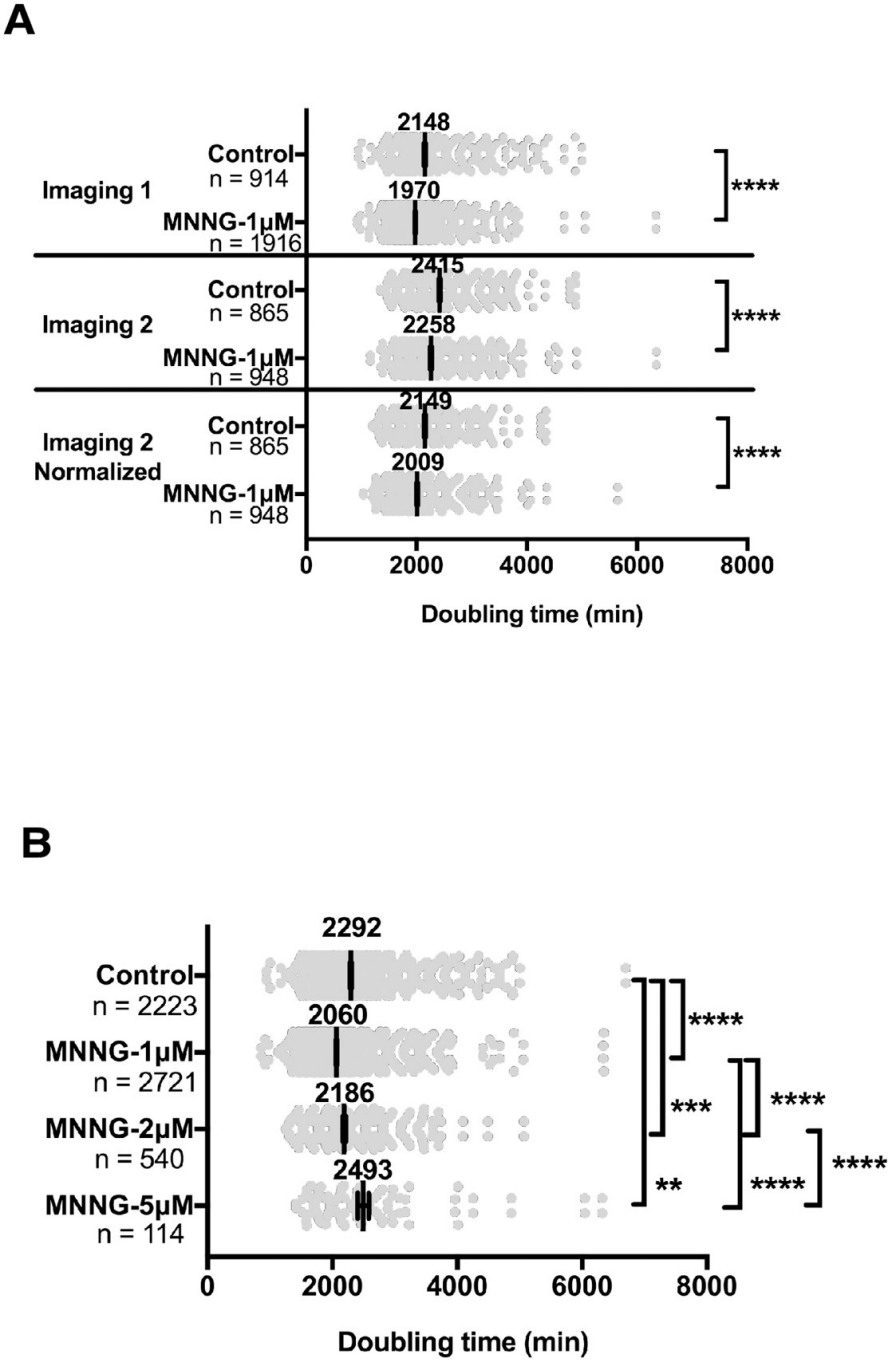
Analysis of cell-doubling time. **A**. Two sets of videos for Control and MNNG-1μM-exposed cells were created by independent long-term live cell imaging and used for cell tracking analysis (Imaging 1 and 2). Cell doubling times analyzed by Imaging 2 were normalized by a factor of 1.124 (Imaging 2, Normalized). Welch’s *t*-tests were performed to analyze the statistical significance between Control and MNNG-1μM; ****p<0.0001. Results shown as the mean ± standard error (SEM). **B**. A cell lineage database was created by merging multiple cell lineage databases. Cell doubling times were analyzed for Control, MNNG-1μM-, MNNG-2μM-, and MNNG-5μM-exposed cells. One-way ANOVA (Tukey’s multiple comparison test) was performed; **p<0.01, ***p<0.001 and ****p<0.0001.

HeLa cells were exposed to three different doses of MNNG for 30 min, as follows: non-cytotoxic (1 μM dose at which cell proliferation was not reduced; MNNG-1μM), sub-cytotoxic (2 μM dose at which CD was induced but proliferation also occurred; MNNG-2μM), and cytotoxic dose (5 μM dose at which CD predominantly occurred; MNNG-5μM) (see S1–S4 and S5 Movies for exposure to a lethal dose of MNNG-40μM). We referred to cells identified at time point 1 as progenitor cells, and these cells and their progeny were then tracked over time and cellular events were identified by morphological observation, which allowed visual detection of all cellular events recorded on the videos, regardless of the molecular events occurring in the cells, to create cell lineage maps (S1–S5 Figs) and a cell lineage database. In the current study, we characterized the responses of HeLa cells to MNNG by live cell imaging of cells exposed to MNNG. We previously reported the reproducibility of the single-cell lineage tracking analysis by evaluating cell-growth curves based on three independent live cell imaging videos of untreated cells [1]. Consistent with the report, a comparison between experiments (Fig 1A, Control, Imaging 1 vs. Imaging 2) showed a 1.124-fold difference in mean cell doubling times. Then, the cell doubling times of Imaging 2 of Control and MNNG-1μM cells were normalized by a factor of 1.124, and the cell doubling time of Imaging 1 and that of normalized Imaging 2 were subjected to statistical analysis. Both experiments showed that MNNG-1μM consistently reduced the doubling time, confirming that variation between imaging studies did not affect the analysis. In the current study, we used merged databases for subsequent analyses. As shown Fig 1B, the cell doubling time of MNNG-1μM cells was significantly shorter than that of Control cells (Fig 1B, Control [2292 min] vs. MNNG-1μM [2060 min], p<0.0001), suggesting that MNNG-1μM reduces the cell doubling time. The mean cell doubling time of cells exposed to MNNG-2μM (2186 min) was also significantly shorter than that of Control cells (Fig 1B, Control [2292 min], p<0.001) and MNNG-1μM-exposed cells (2060 min, p<0.0001). In the case of MNNG-5μM, the cell doubling time of cells exposed to MNNG-5μM was prolonged, possibly because of alterations in cell cycle progression [33] (Fig 1B, Control [2292 min] vs. MNNG-5μM [2493min], p<0.01). We also analyzed cell doubling time using a Gaussian distribution (S5 Fig) and confirmed that cells exposed to MNNG-1μM and MNNG-2μM showed shorter cell doubling times, suggesting that spatiotemporal data obtained by single-cell lineage tracking analysis allows a precise investigation of the effect of exposure to MNNG on cell doubling time.

### Effect of MNNG exposure on the rate of cell population expansion

**Fig 2:**
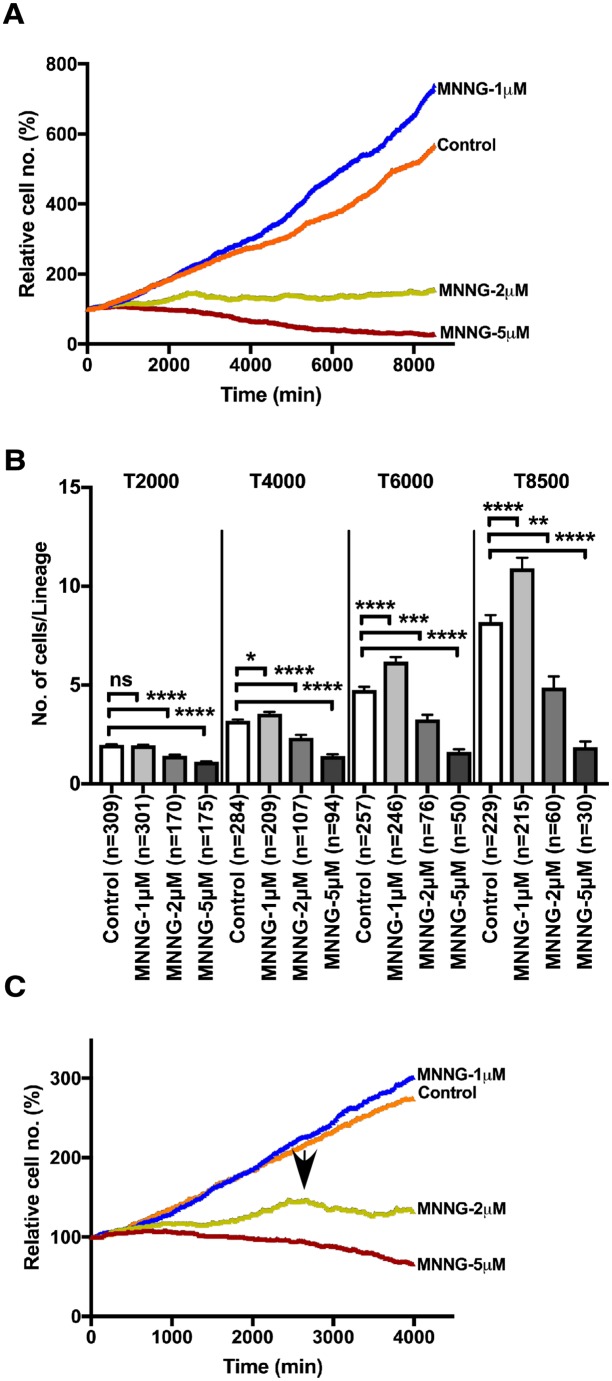
Effect of MNNG exposure on cell proliferation. **A**. The numbers of cells at each time point were determined using the cell lineage database. The initial numbers of cells were normalized by 100. **B**. The numbers of cells produced from each progenitor cell at the 2000, 4000, 6000, and 8500 min time points were calculated. One-way ANOVA (Tukey’s multiple comparison test) was performed. The statistical significance between Control with MNNG-1μM, MNNG-2μM, and MNNG-5μM are shown; ns: not significant, *p<0.05, **p<0.01, ***p<0.001 and ****p<0.0001. Results shown as the mean ± SEM. **C**. Growth curves for the time points from 1 to 4000 min are shown. Arrow indicates the time point at which the numbers of cells exposed to MNNG-2μM reached the maximum.

The effect of MNNG on the rate of cell population expansion was also studied using spatiotemporal data. Conventional methods often determine the rate of cell population expansion by time-course analysis, in which the numbers of cells at each time point were determined by averaging the results from multiple cell counts. In contrast, single-cell lineage tracking analysis determined the numbers of progenitor cells and/or progeny of each cell lineage at each time point (see cell lineage maps for S1–S4 Figs), and used the total for each time point to draw the cell population expansion curves (Fig 2A). We first evaluated the rate of cell population expansion of MNNG-1μM-exposed cells and showed that MNNG-1μM treatment significantly increased the numbers of cells at 8500 min (Fig 2B, T8500, Control vs. MNNG-1μM, p<0.0001), suggesting that MNNG-1μM could promote cell proliferation. In contrast, MNNG-2μM treatment suppressed cell proliferation (Fig 2A and 2B). Although there was a slight increase in cell number at approximately 2500 min (Fig 2C), the overall increase at 8500 min was 1.5-fold (Fig 2A, T8500, Control vs. MNNG-2μM, p<0.01), implying that cellular events, e.g. multipolar cell division (MD) [34, 35], cell fusion (CF), and CD, which cause suppression of cell growth, occur following exposure to MNNG-2μM. A cytotoxic dose of MNNG (Fig 2A, T8500, Control vs. MNNG-5μM, p<0.0001) significantly inhibited cell population expansion. These analyses also suggest that spatiotemporal data can be used to characterize cellular responses to MNNG.

### Characterization of cells exposed to MNNG-5μM using spatiotemporal data

**Fig 3:**
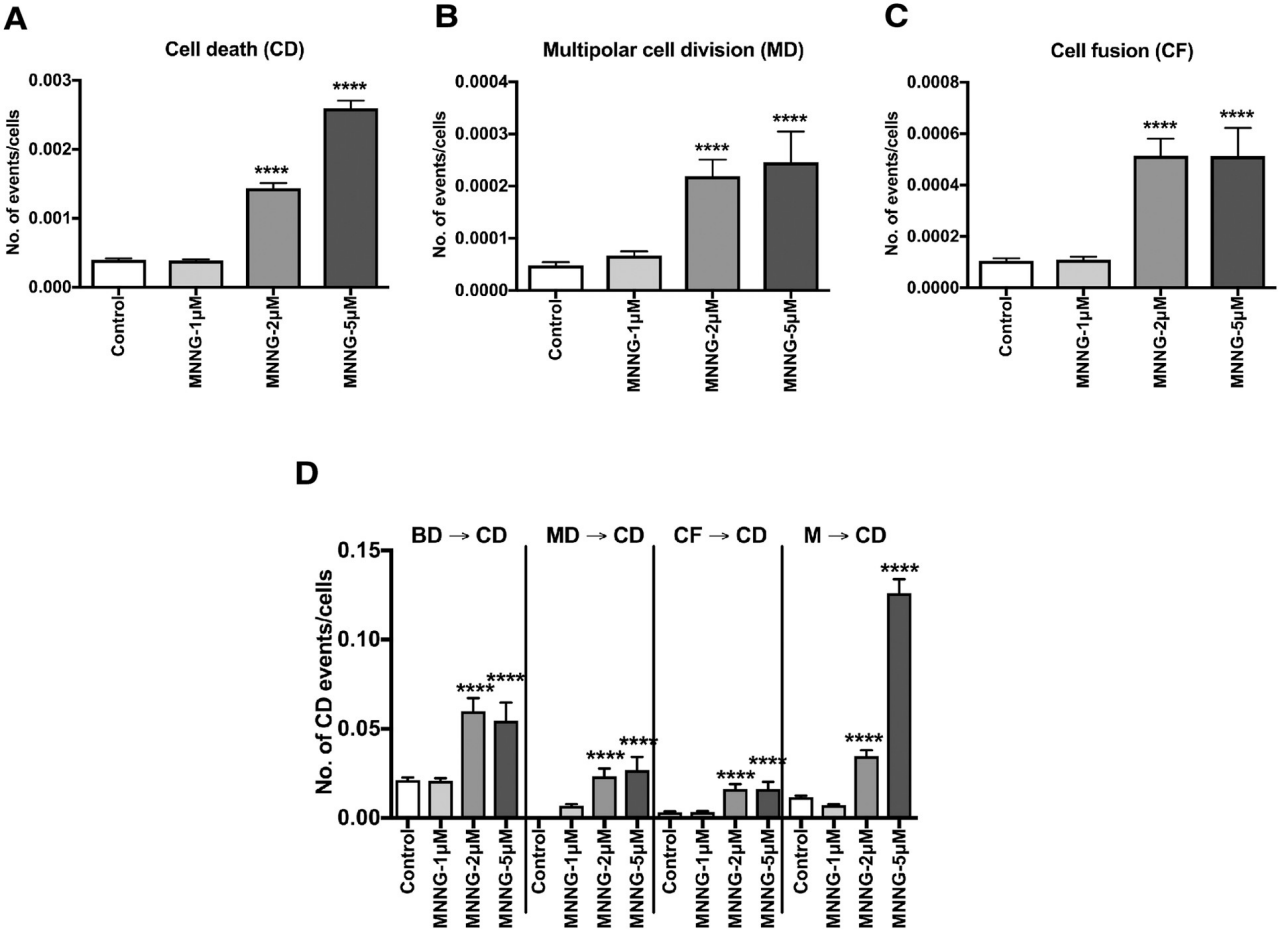
Numbers of CD, MD, and CF events in cells exposed to MNNG. Data in **A-C** were normalized by the total numbers of cells. The numbers of CD (**A**), MD (**B**), and CF (**C**) events that occurred in each cell lineage were determined using the cell lineage database. **D**. The numbers of CD events that occurred in each cell lineage following BD, MD, CF, or M are shown. If the event preceding M was MD or CF, the CD event following the M was included in MD → CD or CF → CD, respectively (see S6 Fig). **A-D**. One-way ANOVA (Tukey’s multiple comparison test) was performed. The statistical significance between Control with MNNG-1μM, MNNG-2μM, and MNNG-5μM are shown; ****p<0.0001. Results shown as the mean ± SEM. Sample sizes of Control, MNNG-1μM, MNNG-2μM and MNNG-5μM were 330, 317, 188 and 200, respectively.

We showed that a cytotoxic dose of MNNG (MNNG-5μM) prolonged cell doubling time (Fig 1B, Control [2292 min] vs. MNNG-5μM [2493min], p<0.01), inhibited cell population expansion (Fig 2A, T8500, Control vs. MNNG-5μM, p<0.0001), and induced CD (Fig 3A, Control vs. MNNG-5μM, p<0.0001). To clarify the individual cell- or cell population-based context leading to the induction of CD, we analyzed the spatiotemporal information on the cellular events experienced by individual cells. The results shown in Fig 3 were normalized to the total numbers of cells to evaluate the chances of CD, MD, and CF events in individual cells. The results suggest that cytotoxic doses of MNNG induce MD (Fig 3B, Control vs. MNNG-5μM, p<0.0001) and CF (Fig 3C, Control vs. MNNG-5μM, p<0.0001). Then, to investigate the relationships between MD and CF events and CD, we identified the cellular events occurring prior to CD. Indeed, 10.3% and 8.2% (see Table 1, S6 Fig for categorization of events and S7 Fig for an overview) of CD induced by MNNG-5μM occurred following MD and CF (Fig 3D, MNNG-5μM, MD → CD, p<0.0001 and CF → CD, p<0.0001). Furthermore, we found that 47.4% (Table 1, M → CD) and 21.0% (Table 1, MNNG-5μM, BD (exc. M) → CD) of CD events occurred following entry of MNNG-5μM-exposed cells into mitosis (M) and bipolar cell division (BD), respectively (Fig 3D, MNNG-5μM, M → CD, p<0.0001 and BD → CD, p<0.0001). Analysis of the responses of cells to MNNG-5μM showed that CD induced by cytotoxic doses of MMNG occurred in the following order: M, BD, MD, and CF prior to CD. As conventional end-point analyses cannot determine events occurring in individual cells prior to CD, the results obtained by end-point analyses may represent the average CD occurring after M, BD, MD, and CF, or alternatively could be biased towards CD induced after one of these events.

**Table 1:** Summary of processes leading to CD.

Treatments	Cell-doubling time		Fold increase in number of cells (relative to number of progenitor cells)	CD^2)^		
	min	fold (relative to Control)		Processes	%	No. of events/100 lineages (total no. of cells/100 lineages)
MNNG-40μM	nd^1)^	nd^1)^	nd^1)^	S5 Movie		
MNNG-5μM^21)^	2493	1.10	0.6	BD (exc. M) → CD^4)^	21.0	25.5
				M → CD^14)^	47.4	57.5
				*BD (M) → CD*^5)^	*8*.*7*	*10*.*5*
				*No Div*. *M → CD*^15)^	*38*.*7*	*47*.*0*
				MD → CD^16)^	10.3	12.5
				CF → CD^17)^	8.2	10.0
				No Div. → CD^18)^	11.9	14.5
				Incomplete DV → CD^19)^	1.2	1.5
				**Total**	**100**	**121.5 (234.0)** ^20)^
MNNG-2μM^21)^	2186	0.96	1.5	BD → CD^3)^	52.5	93.6
				*BD (DV1) → CD (G1)*^6)^	*12*.*3*	*22*.*3*
				*BD (DV1) → CD (S+)*^7)^	*9*.*0*	*16*.*4*
				*BD (DV2) → CD (G1)*^8)^	*6*.*5*	*11*.*0*
				*BD (DV2) → CD (S+)*^9)^	*6*.*5*	*11*.*0*
				*BD (DV3) → CD (G1)*^10)^	*5*.*6*	*10*.*1*
				*BD (DV3) → CD (S+)*^11)^	*7*.*6*	*13*.*8*
				*BD (DV4) → CD (G1)*^12)^	*3*.*8*	*6*.*9*
				*BD (DV4) → CD (S+)*^13^^*)*^	*1*.*2*	*2*.*1*
				No Div. M → CD^15)^	12.9	23.4
				MD → CD^16)^	16.1	29.3
				CF → CD^17)^	13.8	25.0
				No Div. → CD^18)^	4.7	8.5
				Incomplete DV → CD^19)^	0	0
				**Total**	**100**	**179.8 (671.2)** ^20)^
MNNG-1μM^21)^	2059	0.90	7.3	BD → CD^3)^	68.4	160.4
				*BD (DV1) → CD (G1)*^6)^	*2*.*6*	*6*.*0*
				*BD (DV1) → CD (S+)*^7)^	*6*.*9*	*16*.*1*
				*BD (DV2) → CD (G1)*^8)^	*5*.*1*	*12*.*0*
				*BD (DV2) → CD (S+)*^9)^	*11*.*3*	*26*.*5*
				*BD (DV3) → CD (G1)*^10)^	*6*.*9*	*16*.*1*
				*BD (DV3) → CD (S+)*^11)^	*16*.*3*	*38*.*2*
				*BD (DV4) → CD (G1)*^12)^	*11*.*0*	*25*.*9*
				*BD (DV4) → CD (S+)*^13)^	*8*.*3*	*19*.*6*
				No Div. M → CD^15)^	2.4	5.7
				MD → CD^16)^	17.2	40.4
				CF → CD^17)^	9.8	23.2
				No Div. → CD^18)^	1.8	4.1
				Incomplete DV → CD^19)^	0.4	0.9
				**Total**	**100**	**232.7 (1907.0)** ^20)^
Control^21)^	2276	1.0	6.0	BD → CD^3)^	76.2	231.4
				*BD (DV1) → CD (G1)*^6)^	*2*.*7*	*8*.*3*
				*BD (DV1) → CD (S+)*^7)^	*13*.*3*	*40*.*3*
				*BD (DV2) → CD (G1)*^8)^	*6*.*4*	*19*.*4*
				*BD (DV2) → CD (S+)*^9)^	*18*.*0*	*54*.*6*
				*BD (DV3) → CD (G1)*^10)^	*8*.*2*	*25*.*0*
				*BD (DV3) → CD (S+)*^11)^	*15*.*1*	*45*.*8*
				*BD (DV4) → CD (G1)*^12)^	*8*.*7*	*26*.*4*
				*BD (DV4) → CD (S+)*^13)^	*3*.*8*	*11*.*6*
				No Div. M → CD^15)^	1.7	5.1
				MD → CD^16)^	9.9	30.1
				CF → CD^17)^	10.1	30.6
				No Div. → CD^18)^	1.4	1.9
				Incomplete DV → CD^19)^	0.7	4.2
				**Total**	**100**	**303.3 (1507.3)** ^20)^

^1)^nd: not determined.

^2)^Aberrations used in the Table 1 are; BD, bipolar cell division; MD, multipolar cell division; M, mitosis; CD, cell death; CF, cell fusion, DV1, cell division of progenitor cells; DV2, cell division of daughter cells; DV3, cell division of granddaughter cell; DV4, cell division of grand-granddaughter cell; No Div., no cell division and Incomplete DV, cells, which undergo M, but fail to divide.

^3)^BD → CD: CD occurred following BD.

^4)^BD (exc. M) → CD: CD occurred prior to M.

^5)^BD (M) → CD: CD occurred following M.

^6)^BD (DV1) → CD(G1): CD occurred during G1 phase of daughter cell produced by DV1.

^7)^BD (DV1) → CD(S+): CD occurred during S+G2+M phase of daughter cell produced by DV1.

^8)^BD (DV2) → CD(G1): CD occurred during G1 phase of granddaughter cell produced by DV2.

^9)^BD (DV2) → CD(S+): CD occurred during S+G2+M phase of granddaughter cell produced by DV2.

^10)^BD (DV3) → CD(G1): CD occurred during G1 phase of grand-granddaughter cell produced by DV3.

^11)^BD (DV3) → CD(S+): CD occurred during S+G2+M phase of grand-granddaughter cell produced by DV3.

^12)^BD (DV4) → CD(G1): CD occurred during G1 phase of cells produced by DV4.

^13)^BD (DV4) → CD(S+): CD occurred during S+G2+M phase of cells produced by DV4.

^14)^M → CD: CD occurred following M.

^15)^No Div. M → CD: CD occurred following M, but preceding events of M were unable to be determined.

^16)^MD → CD: CD occurred following MD.

^17)^CF → CD: CD occurred following CF.

^18)^No Div. → CD: CD, of which preceding events were unable to be determined.

^19)^Incomplete DV → CD: CD occurred following incomplete DV.

^20)^Total numbers of cells per 100 cell lineages are shown.

^21)^In cells exposed to MNNG-5μM, CD occurred following BD and M were categorized into BD (exc. M) → CD and M → CD, respectively. M → CD was sub-categorized into BD (M) → CD and No Div. M → CD. In cells exposed to MNNG-2μM and MNNG-1μM, and Control cells, CD occurred following BD and M were categorized into BD → CD and No Div. M → CD, respectively. See S6 Fig for categorization of events and S7 Fig for an overview.

### Characterization of cells exposed to MNNG-2μM using spatiotemporal data

**Fig 4:**
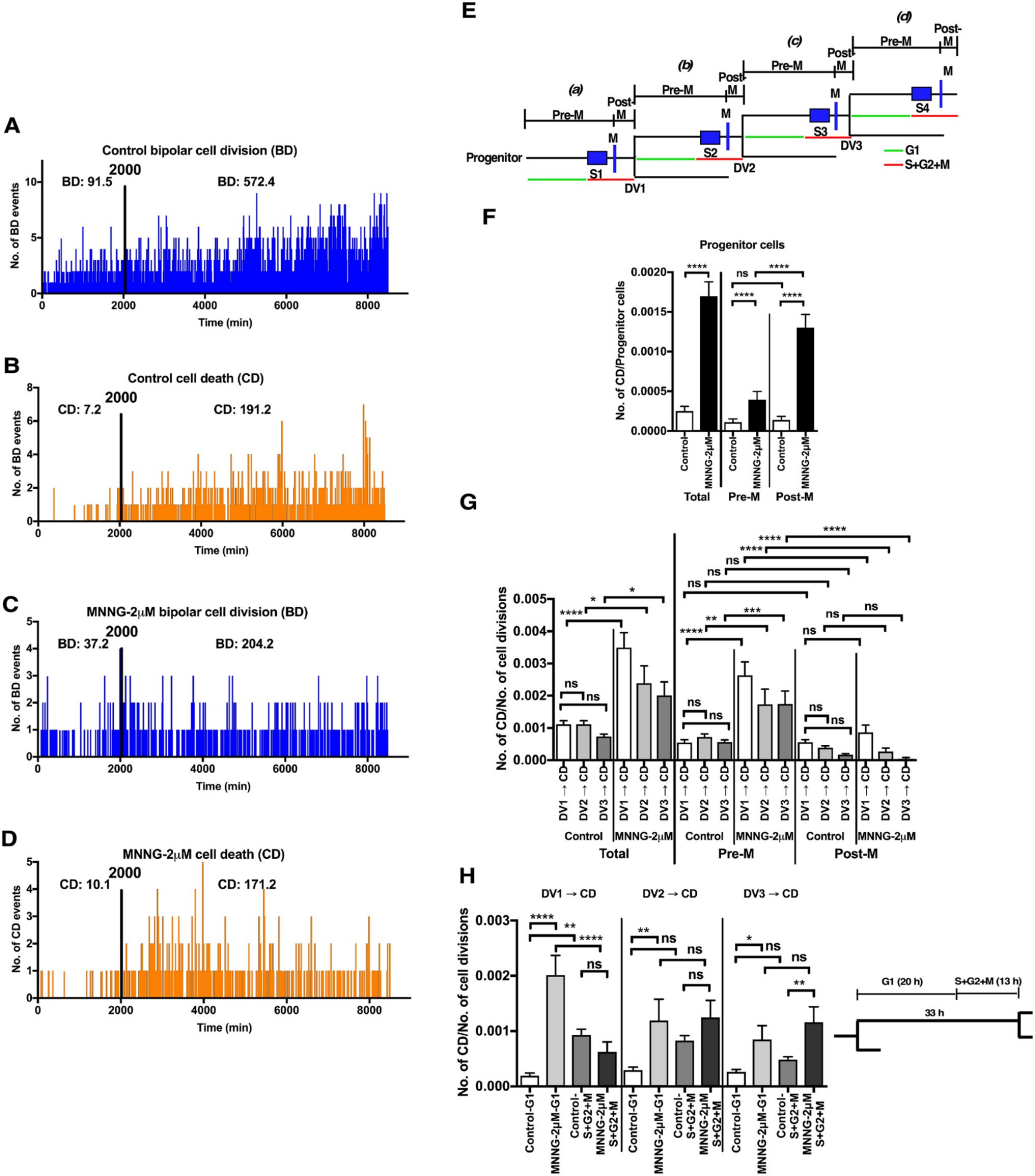
Analysis of cells exposed to MNNG-2μM. The numbers of BD (**A and C**) and CD (**B and D**) events occurring at each time point in Control cells (**A and B**) and cells exposed to MNNG-2μM (**C and D**) are plotted. The total numbers (per 100 cell lineages) of BD and CD events that occurred before and after 2000 min were determined (**A-D**). **E**. Definitions used in this Figure are listed. Progenitor cells ***(a)*** undergo the first S phase (S1), followed by mitosis (M) and the first cell division (DV1), and the periods before and after entering M are defined as Pre-M and post-M, respectively. Daughter ***(b)***, granddaughter (GD)***(c)***, and grand-granddaughter (GGD)***(d)*** cells undergo second, third, and fourth S phases (S2, S3, and S4, respectively). DV2 and DV3 represent the cell division of daughter and GD cells, respectively. Durations of G1 (green line) and S+G2+M (red line) are shown. **F**. The numbers of CD events occurring in progenitor cells ***(a)*** were normalized to the numbers of progenitor cells. Welch’s *t*-test was performed for Total; ****p<0.0001, and One-way ANOVA (Tukey’s multiple comparison test) was performed for Pre-M together with Post-M; ns: not significant and ****p<0.0001. Results shown as the mean ± SEM. **G**. The numbers of CD events that occurred in each cell lineage following DV1, DV2, and DV3 were determined, and, then, the numbers were normalized to the numbers of cell divisions (DV1, DV2, and DV3, respectively), to adjust for the growth of cells. One-way ANOVA (Tukey’s multiple comparison test) for Total and another One-way ANOVA for Pre-M together with Post-M were performed; ns: not significant, *p<0.05, **p<0.01, ***p<0.001 and ****p<0.0001. Results shown as the mean ± SEM. **F and G** Total: the total numbers of CD events occurred in each cell lineage during Pre-M and Post-M. **H**. The cell doubling time of cells exposed to MNNG-2μM was assumed to be 33 h; the duration of S1+G2+M phases was 13 h, and the duration of G1 was thus 20 h. CD events occurring during G1 and S+G2+M phases were determined. One-way ANOVA (Tukey’s multiple comparison test) for DV1 → CD, DV2 → CD and DV3 → CD was independently performed; ns: not significant, *p<0.05, **p<0.01, and ****p<0.0001. **F-H**. Sample sizes of Control and MNNG-2μM were 330 and 188, respectively. Results shown as the mean ± SEM.

In contrast to cells exposed to MNNG-5μM, cells treated with MNNG-2μM were able to proliferate (Fig 2C, MNNG-2μM), and did not appear to undergo immediate CD. Clinically relevant doses of alkylating agents do not induce immediate responses [36, 37], suggesting that MNNG-2μM may represent a clinically relevant dose equivalent. We therefore analyzed the responses of HeLa cells exposed to MNNG-2μM using spatiotemporal data. Similar to cells exposed to MNNG-5μM, the numbers of CD, MD, and CF events were significantly increased by exposure to MNNG-2μM (Fig 3A–3C, Control vs. MNNG-2μM). However, in contrast to MNNG-5μM-exposed cells, CD occurred more frequently in cells following BD (Fig 3D, BD → CD, Control vs. MNNG-2μM) compared with following entry into M (Fig 3D, M → CD, Control vs. MNNG-2μM), implying that the cellular response induced by MNNG-2μM is different from that induced by MNNG-5μM. Thus, we further explored the occurrence of CD induced by MNNG-2μM by comparing the frequencies of BD and CD at each time point, given that the growth of MNNG-2μM cells is likely to represent a balance between cell generation and CD (Fig 2A). The numbers of BD and CD events plotted at each time point are shown in Fig 4A–4D. BD occurred constantly throughout the observation period in Control cells (Fig 4A) and 96.4% of CD occurred after 2000 min (Fig 4B). BD also occurred throughout the observation period in MNNG-2μM-exposed cells, but there were fewer BD cells compared with Control cells (Fig 4A and 4C, 91.5 BD [Control] vs. 37.2 [MNNG-2μM]), while the majority of CD (94.4%) also occurred after 2000 min (Fig 4D). We evaluated the relationship between BD and CD quantitatively by calculating the CD/BD ratio. The CD/BD ratio in Control cells before 2000 min was 7.2/92.5 = 0.08, implying that BD without induction of CD was predominant. This ratio was increased to 10.1/37.2 = 0.27 by exposure to MNNG-2μM, due to a reduction in BD. However, BD was still predominant, resulting in a small increase in cell population size (Fig 2C). The CD/BD ratio in the Control cells after 2000 min was 197.2/572.4 = 0.33, reflecting the predominance of cell growth over CD, while the ratio in the MNNG-2μM-exposed cells was 171.4/204.2 = 0.84, which was close to 1.0, indicating that proliferation of MNNG-2μM-exposed cells is largely balanced by CD, resulting in a 1.5-fold increase in cell population size (Fig 2A).

Given that the mean cell doubling time of MNNG-2μM-exposed cells was 2186 min (Fig 1B, MNNG-2μM), these results also suggest that CD is likely to occur after a cell division. Indeed, previous end-point analyses suggest that CD induced by exposure to low levels of alkylating agents occurs after the first S phase by recognition of *O*^6^-methyl G:T and *O*^6^-methyl G:C mismatches [23, 24], the second S phase by removal of mismatches by mismatch correction [21, 22, 32] or the third S phase entering after the formation of multinuclear cell [29]. We therefore determined the frequency of CD occurring prior to the division of progenitor cells (DV1, Fig 4E***(a)***), daughter cells (DV2, Fig 4E***(b)***), and granddaughter (GD) cells (DV3, Fig 4E***(c)***). First, we asked whether MNNG-2μM induced CD in progenitor cells (Fig 4E***(a)***). As shown in Fig 4F, CD occurring during ***(a)*** was significantly increased in cells exposed to MNNG-2μM (Fig 4F, Total); the increase was also observed before (Pre-M) and after (Post-M) the entry of cells into M phase (Fig 4F, Pre-M and Post-M), although CD events were more frequent at Post-M (Fig 4F, MNNG-2μM, Pre-M vs. Post-M). These results support the hypothesis that CD induced by exposure to low levels of alkylating agents occurs after the first S phase (Fig 4E, S1 Fig) [23, 24]. CD occurring during ***(a)*** was, however, only 17.6% of CD occurring in cells exposed to MNNG-2μM (Pre-M corresponding to Table 1, MNNG-2μM, No Div. → CD (4.7%) + Post-M corresponding to No Div. M → CD (12.9%) = 17.6%)), implying that the remaining 82.4% of CDs occur following the first cell division. We thus determined the numbers of CD events induced after DV1, DV2, and DV3. As the numbers of cell divisions increased following cell growth, the numbers of CD events after DV1, DV2, and DV3 (Fig 4E***(b)***, 4E***(c)*** and 4E***(d)***) were normalized to the numbers of cell divisions, i.e. DV1, DV2, and DV3, respectively, to adjust for the effect of cell growth. In Control cells, CD was spontaneously induced during cell culture at a similar rate, as there was no significant difference between the frequency of CD in DV1 → CD with DV2 → CD and DV3 → CD (Fig 4G, Total, Control). By contrast, in MNNG-2μM treated cells, CD events following DV1 were significantly increased compared with those in Control cells, and a similar increase was observed after DV2 and DV3 (Fig 4G, Total, Control vs. MNNG-2μM). These results suggest that CD occurs more frequently in MNNG-2μM-exposed cells after DV1 than after DV2 and DV3, as reported previously [21, 22, 32], although significant CD was also induced by MNNG-2μM following DV2 and DV3.

As such an increase was found during Pre-M (Fig 4G, Pre-M), but not Post-M (Fig 4G, Post-M), CD induced by MNNG-2μM likely occurred prior to entering into M, i.e. during G1, S, and/or G2 phase. In this regard, it has been proposed that CD occurs following G2 arrest [21, 22, 32]; however, another study reports that the arrest is not involved in CD induced by low-dose alkylating agents [29]. We therefore asked whether CD was induced during G1, S, and/or G2. Because single-cell tracking analysis was performed by observation of cell morphology, these phases could not be directly detected. Instead, as the analysis allowed the precise determination of the cell doubling time of individual cells, we assumed that the duration of G1 phase was 20 h (Fig 4H, right) by subtracting 13 h, which is the total duration of the S, G2, and M phases, from the mean cell doubling time of cells exposed to MNNG-2μM (Fig 1B, 2186 min = 33 h). In Control cells, CD induced after DV1 occurred mainly in S+G2+M phase (Fig 4H, DV1 → CD, Control-G1 vs. Control-S+G2+M). MNNG-2μM-induced CD occurred predominantly during G1 phase (Fig 4H, DV1 → CD, MNNG-2μM-G1 vs. MNNG-2μM-S+G2+M), and a similar tendency was observed regarding CD induced after DV2 and DV3, supporting the hypothesis that some CD events are predominantly induced without cell cycle arrest at G2 phase [29]. In summary, CD events, which were induced by exposure of cells to MNNG-2μM, occurred through various processes as summarized in Table 1 and S7 Fig, implying that investigations of CD in MNNG-2μM-exposed cells using conventional end-point analyses may produce different conclusions depending on which specific processes are detected at the time of the analysis.

### Characterization of cells exposed to MNNG-1μM using spatiotemporal data

**Fig 5:**
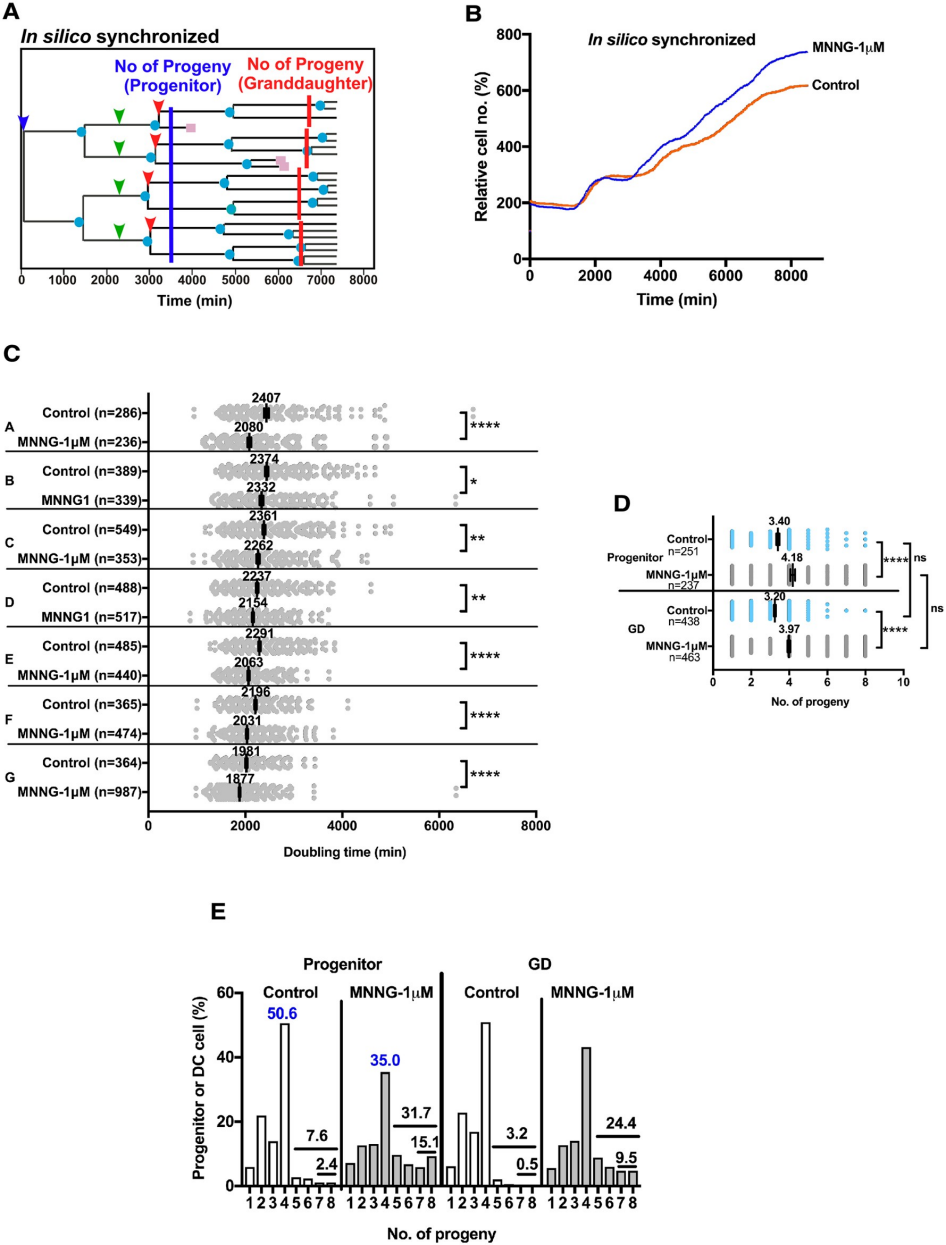
Analysis of progeny produced from Control and MNNG-1μM-exposed cells. **A**. The cell cycle was synchronized *in silico*. The time point when the progenitor cells divided was normalized as Time 1 (blue arrowhead). Blue line indicates the time point 3500 min after the division. GD cells are indicated by green arrowheads. The red line shows the time point 3500 min after the division of GD cells (red arrowheads). **B**. Cell growth curves determined after synchronization. **C**. Progenitor cells were grouped (A-G) according to the numbers of progeny cells, as shown in Table 2. Each group was composed of at least 200 cells. The cell doubling times of each group of cells were then determined to perform Welch’s *t*-tests; ns: not significant, and *p<0.05, **p<0.01 and ****p<0.0001 in relation to Control. Results shown as the mean ± SEM. An *in silico* synchronized-cell lineage database was used. **D and E**. The numbers of progeny produced from a progenitor at 3500 mins and from GD cells at 3500 min from their cell division were determined. **D**. One-way ANOVA (Tukey’s multiple comparison test) was performed; ns: not significant, and ****p<0.0001. Results shown as the mean ± SEM. **E**. The numbers of progenitor and GD cells producing 1–8 progeny cells are shown. The vertical scale represents the percentage of progenitor or GD cells producing 1–8 progeny cells. The values shown in the figure represent progenitor cells that produced 4 progeny (blue), and the percentages of the total numbers of progenitor and GD cells that produced 5, 6, 7, and 8 (upper line) and 7 and 8 progeny (lower line).

We defined a non-cytotoxic dose of MNNG as a dose at which cell proliferation was not reduced and investigated the cellular alterations and responses induced by such a non-cytotoxic dose of MNNG. MNNG-1μM shortened the cell doubling time (Fig 1B, Control [2292 min] vs. MNNG-1μM [2060 min]) and promoted cell proliferation (Fig 2A and 2B, Control vs. MNNG-1μM), suggesting that MNNG-1μM promotes growth of HeLa cells by reducing cell doubling time. We previously investigated the roles of individual HeLa and A549 cells in maintaining the cell population by synchronizing the cell cycle *in silico*, by grouping cells based on the numbers of progeny produced by a progenitor cell, and identified a well-growing sub-population [1, 4]. We used a similar approach to characterize cells exposed to MNNG-1μM in the current study. The *in silico* cell cycle synchronization process normalized the first cell division as time point 1 (Fig 5A, blue arrowhead) and time point data recorded in the cell lineage database were modified after normalization. Fig 5B shows the cell growth curve after synchronization of the cell cycle, confirming the *in silico* cell cycle synchronization (stair-shaped curve instead of the linear curve shown in Fig 2A). We then determined the numbers of progeny generated by a single progenitor cell at 8500 min using the synchronized data. As HeLa cells contain cell populations with different reproductive abilities [1], progenitor cells were classified based on the numbers of progeny produced from a progenitor cell as shown in Table 2 (Group A, 0–2; B, 3–5; C, 6–8; D, 9–11; E, 12–14; F, 15–17; and G, ≥ 18 cells) to study the effect of MNNG-1μM on cell doubling time. Any group of cells exposed to MNNG-1μM consistently showed a shorter cell doubling time (Table 2 and Fig 5C), suggesting that MNNG-1μM reduces cell doubling time regardless of their reproductive ability. Among the groups, Group G cells produced ≥18 progeny cells (Table 2, 4.29% of Control vs. 11.68% of cells exposed to MNNG-1μM), suggesting that cells with a higher reproductive ability than that of Control cells are produced by the exposure to MNNG. MNNG-1μM was therefore likely to stimulate the growth of cells by increasing their reproductive ability and shortening cell doubling time.

**Table 2:** The numbers of progeny produced from a progenitor cell and the mean cell doubling times.

Group: No. of progeny^1)^	Control		MNNG-1μM		MNNG-2μM		MNNG-5μM	
	% ^2)^	Doubling time (min)^3)^	%^2)^	Doubling time (min)^3)^	%^2)^	Doubling time (min)^3)^	%^2)^	Doubling time (min)^3)^
A: 0–2	35.64	***2435 ± 47*.*8*** (n = 286)	39.42	*2080 ± 38*.8**** (n = 236)	80.31	*2135 ± 42*.4**** (n = 160)	96.50	***2332 ± 38*.*2*** (n = 66)
B: 3–5	16.50	***2440 ± 33*.*6*** (n = 389)	12.93	*2332 ± 38*.2* (n = 339)	9.55	*2189 ± 58*.2*** (n = 100)	2.50	***2495 ± 116*.*9*** (n = 31)
C: 6–8	16.83	***2383 ± 0*.*4*** (n = 549)	9.15	*2262 ± 42*.4** (n = 353)	3.72	*2189 ± 58*.2** (n = 100)	1.00	*2076 ± 93*.2** (n = 25)
D: 9–11	11.55	***2240 ± 23*.*8*** (n = 488)	11.36	***2154 ± 20*.*6*** ** (n = 517)	3.19	***2249 ± 52*.*2*** (n = 112)	0	***0***
E: 12–14	9.57	***2290 ± 22*.*0***	7.89	*2063 ± 20*.0**** (n = 160)	2.17	*1985 ± 42*.3**** (n = 80)	0	***0***
F: 15–17	5.62	***2207 ± 22*.*1*** (n = 365)	7.57	*2031 ± 19*.4**** (n = 474)	0.53	*2004 ± 63*.0** (n = 30)	0	***0***
G: ≥ 18	4.29	***2019 ± 20*.*7*** (n = 364)	11.68	***1876 ± 13*.*3*** **** (n = 987)	0.53	*2210 ± 69*.2* (n = 39)	0	***0***
Total	100		100		100		100	

^1)^ Progenitor cells were grouped (Group A-G) by the numbers of progeny produced at time 8500 min.

^2)^ The % of progenitor cells that produced the numbers of progeny shown in the “Group: No. of progeny” column is shown.

^3)^ The mean cell doubling times and SEMs were calculated using the cell doubling times of each cell.

Welch’s *t-tests* were performed;

*p < 0.05,

**p < 0.01, and

****p < 0.0001 in relation to Control.

To further gain insight into the factors promoting the reproductive ability of cells in response to exposure to MNNG-1μM, we analyzed the numbers of progeny produced from individual cells. First, we compared the numbers of progeny produced from each progenitor cell and GD cell to determine whether the promotion occurred only in progenitor cells, which were exposed to MNNG-1μM, or whether the effect was maintained in the progeny, e.g. GD cells. For this purpose, we determined the numbers of progeny produced from a progenitor cell 3500 min after the first division of the progenitor cell (Fig 5A, blue line), and then identified the GD cells (Fig 5A, green arrowhead) of the progenitor cell. Then, we determined the numbers of progeny cells produced from the GD cells 3500 min (Fig 5A, red lines) after the first cell division (Fig 5A, indicated by red arrowheads) of the GD cells. If a GD cell retained the reproductive ability of its progenitor cell, the GD cell would be expected to produce a similar number of progeny cells as that of the progenitor cell. We found no significant difference between the numbers of progeny produced from MNNG-1μM-exposed progenitor cells and that of GD cells (Fig 5D, Progenitor vs. GD), indicating that the effect of MNNG-1μM exposure is retained in GD cells. Furthermore, these results confirmed that progenitor and GD cells exposed to MNNG-1μM produced more progeny than the respective Control cells (Fig 5D, Control vs. MNNG-1μM). Then, we reorganized the data in Fig 5D according to the numbers of progeny cells produced by a progenitor cell and a GD cell (Fig 5E). The figure shows that 50.6% of progenitor Control cells produced four progeny after 3500 min of culture, whereas the numbers of progenitor cells producing the same numbers of progeny was reduced to 35.0% by exposure of the cells to MNNG-1μM. On the other hand, the numbers of progenitor cells that produced 5–8 progeny were increased to 31.7% from 7.6%, and that of cells producing 7–8 progeny cells were increased to 15.1% from 2.4% (Fig 5D, Progenitor, Control vs. MNNG-1μM). A similar tendency was observed in GD cells. The difference in the percentages—e.g. 24.1% = 31.7% (Fig 5E, Progenitor, MNNG-1μM) minus 7.6% (Fig 5E, Progenitor, Control)—likely represented the conversion of cells with lower reproductive ability into more highly reproductive HeLa cells, suggesting that MNNG-1μM promotes the cellular reproductive ability. With regard to the highly reproductive cells, we previously reported that HeLa cells contain immortal cells, accounting for 3%–7% of the HeLa cell population, and these highly reproductive cells maintain the HeLa cell population by self-renewal and by producing mortal progeny; these highly reproductive cells were tentatively referred to as putative cancer stem cells [1]. Whether these cells are cancer stem cells that have been identified *in vivo* remains to be elucidated; however, it is likely that MNNG-1μM increased the cell population by generating this group of cells with a role in maintaining the HeLa cell population.

## Discussion

Various end-point analyses have been used to investigate cellular responses to exogenously added substances. These analyses generate data on the characteristics of cells at a specific moment in time, and the events are thus inferred based on data obtained at the specific time when the cells were analyzed. In addition, such deductions are often based on the assumption that 1. cells in the population are homogenous and 2. cells respond to an exogenously added substance in a stoichiometric manner; however, cells may not respond in such a manner, given that cultured cells are composed of heterogeneous cell sub-populations [1], which may have intrinsically different sensitivities to a substance. End-point analyses may thus have a limited ability to characterize the responses of such cells to an exogenous substance. In contrast, single-cell tracking analysis can provide spatiotemporal data, which allows the responses of cells to be analyzed without the need for relying on deductions and assumptions. However, such an approach for characterizing cells exposed to cytotoxic, mutagenic and carcinogenic substances has not yet been developed. In the present study, we validated this approach by characterizing cells exposed to various doses of MNNG using spatiotemporal data obtained from single-cell lineage tracking analysis. CD induced by different doses of MNNG is summarized in Table 1 and S7 Fig. A lethal dose of MNNG (40 μM) induced CD within 4 h (S5 Movie). A cytotoxic dose of MNNG predominantly induced CD following M (BD (M) → CD+No Div. M → CD = 47.4%) or BD (BD (exc. M) → CD, 21.0%). Thus, an end-point analysis to detect CD in cells exposed to cytotoxic doses of MNNG would have about a 50% and 20% chance of detecting CD events occurring following M and BD events, respectively, making it difficult for conventional end-point analyses to determine which type of CD was analyzed. In the case of cells exposed to a sub-cytotoxic dose of MNNG (MNNG-2μM), CD events were induced via more diverse processes than those induced by MNNG-5μM. Although CD occurring after BD (DV1–G1; i.e., CD occurred at G1 phase after the first BD) was a major cause of CD induced by MNNG-2μM, CD after MD and CF were also induced at similar frequencies. End-point analyses of cells exposed to MNNG-2μM may thus reach different conclusions depending on which CD process is analyzed. Indeed, previous reports suggest that CD occurs in cells exposed to low doses of an alkylating agent during S or G2 phase of daughter cells produced by BD of exposed progenitors, or following DV1, or DV2 [21–24, 26–32]. It is likely that these reports investigated a specific CD process among the various processes identified here. We therefore consider that spatiotemporal data are essential for studying cellular responses to sub-cytotoxic doses of MNNG.

In the current work, we also demonstrated that a non-cytotoxic dose of MNNG stimulated cell proliferation by promoting the reproductive ability of cells. MNNG is known to induce hyperplasia in rodents [38, 39], and non-cytotoxic dose of MNNG may thus act as a cell growth promoter. However, the doses of carcinogens used in laboratory tests are generally 100–1,000 times higher than the doses present in the environment [40], as environmental doses are unlikely to induce detectable responses in cultured cells. The biological responses induced by environmental doses of carcinogens are thus poorly understood. Although MNNG is not an environmental carcinogen, our results suggest that cellular responses induced by doses that are too low to cause significant induction of CD can still be analyzed based on spatiotemporal data for individual cells.

In summary, we propose that a single-cell lineage tracking analysis that creates spatiotemporal data for individual cells represents a novel and potential bioinformatics approach for elucidating the effects of cytotoxic, sub-cytotoxic and non-cytotoxic doses of various substances. It is plausible that cytotoxicity-induced CD is related to the events occurring prior to CD. Thus, spatiotemporal context of CD induction may have influence on the molecular processes that lead to CD, which is known to be induced by various mechanisms [41]. Taking the spatiotemporal context of CD induction into account will, therefore, allow a deeper understanding of the overall process of CD. On the other hand, single-cell lineage tracking analysis remains to be improved, as the analysis is a tedious process. Thus, as a research tool, it may be realistic to use this analysis together with existing end-point analyses, which are less time-consuming compared with the tracking analysis, after validation of end-point analyses using the single-cell lineage tracking approach. Nevertheless, full computerization of single-cell lineage tracking analysis is necessary for this type of analysis to become a routine method for characterizing cultured cell lines, which are likely composed of heterogeneous populations, and for studying the responses of cells exposed to genotoxic substances. Such computerization will eventually allow the investigation of spatiotemporal alterations or responses of individual cells at the molecular level. Thus, an approach that tracks individual cells, creates a cell lineage database, and uses the database to characterize cells, will promote the design of novel cell biological and bioinformatics research studies.

## Materials and methods

### Cell culture

HeLa cells were purchased from ATCC and cultured in DMEM containing 10% fetal bovine serum in a humidified 5% CO_2_. To plate cells onto a coverglass Lab-Tek 8 well chamber, 50 μl of HeLa suspension containing 3500 cells were placed at the center of each well and left until cells attached to the coverglass surface. Then, 0.75 ml of culture medium was added to each well. Cells were used for live cell imaging 18 h after the plating. The treatment of cells to MNNG were performed for 30 min in serum-free DMEM.

### Long-term live cell imaging and data analysis

Quorum WaveFX Spinning Disc Confocal System (Quorum Technologies Inc., Canada) with Leica microscope controlled by Volocity v4.0 was used for long-term live cell imaging. To minimize induction of CD by phototoxicity, DIC imaging was used and the images were taken through HCX PL APO 40x oil objectives (NA = 1.25) by using a Halogen lamp as a light source. Cells that were grown on a coverglass Lab-Tek 8 well chamber were then placed on a microscope stage and were cultured using an environmental chamber at 37°C with 7.5% humidified CO_2_ (Pathology Devices Inc, MD). In each well, a two-dimensional image acquisition array (filed of views: FOVs) was made to cover the area of interest [1]. XY positions of FOVs were then registered using Volocity v4.0. DIC images were captured every 10 min from + 10 to– 10 μm of the focal plane at every 1 μm using piezo focus drive. Exposure time was 34 msec. To make time-lapse movies, focused images were selected from 21 *z*-plane image files using Volocity v4.0. After the selection, files containing focused images were assembled into a movie using QuickTime Player Pro. Panorama views of time point = 1 were prepared and cell lineage numbers were assigned to cells in a selected area [1]. After assigning the cell lineage numbers, each cell was tracked using QuickTime Player Pro and the time points that M, BD, MD, CF, incomplete DV and CD occurred in each cell were determined to create a cell lineage database. To draw cell lineage maps and process data, C/C++ programs were written. Live cell imaging was started when 80% of the surface of cultured dish was occupied by cells and we have previously confirmed that cell growth was continued for at least 8500 min (see S1 and S2 Movies, Fig 2 and ref. [1]).

### Single-cell tracking

Single-cell tracking by morphological observation of cells was performed by displaying live cell imaging videos to a monitor using QuickTime Player Pro. Progenitor cells and their progeny were individually followed using QuickTime Player Pro and times that M, BD, MD, CF, CD and incomplete DV occurred were recorded. M was identified by the formation of round mitotic cells. BD was found by the division of mitotic cells into two daughter cells. In the case that cells were divided into three or four daughter cells, we defined the division as MD. CF was identified when two individual cells form one cell. CD can be found by disrupting the shape of cells. Incomplete DV is an event that cells undergo M, but fail to divide. Still images and videos of each event were reported previously [1]. In S6 Fig, we schematically summarized these cellular events.

### Determination of non-cytotoxic, sub-cytotoxic and cytotoxic doses of MNNG

To predetermine the dose range of MNNG, which was used for long-term live cell imaging, cells were plated onto a coverglass Lab-Tek 8 well chamber, exposed to various doses of MNNG for 30 min in serum-free DMEM and performed long-term live cell imaging by culturing cells in environmental chamber at 37°C with 7.5% humidified CO_2_. We first performed preliminary evaluation by analyzing the videos and by counting the numbers of cells found at 3000 and 6000 min. Based on the numbers of survived cells, we selected non-cytotoxic, sub-cytotoxic and cytotoxic doses of MNNG. After the selection, live cell imaging was carried out 2 to 3 times. For cells exposed to 2 μM and 5 μM of MNNG, we confirmed cytotoxic effects by preliminary counting, and then, 188 and 200 progenitor cells, respectively, were selected for detailed single-cell lineage tracking analyses as reported [1]. At the end of live cell imaging, the total numbers of tracked cells that were exposed to 2 μM and 5 μM of MNNG was 1425 and 478 cells, respectively. For Control cells and cells exposed to 1 μM of MNNG, we tracked 330 and 317 of progenitor cells and their progeny, respectively, and the total numbers of tracked cells at the end of live cell imaging were 4974 and 6048 cells, respectively. To evaluate the effects of selected doses of MNNG on the growth of cells cultured in a CO_2_ incubator, cells were plated on 3.5 cm dishes, marked on the base, exposed to MNNG as described, and cultured at 37°C with 5.0% humidified CO_2_. Cell images were then acquired by phase-contrast microscopy at the marks every 24 h for 6 days. The numbers of cells within a 500×500 pixel square in the acquired image were then determined. Cell growth rates were about 1.3-fold higher than on the microscope (S8A Fig), consistent with our previous report [1]. To confirm that these doses of MNNG induce DNA damage, we exposed the cells to various doses of MNNG as described, followed by washing three times with phosphate-buffered saline (PBS), fixing with 0.5% paraformaldehyde for 15 min at room temperature, permeabilized with PBS containing 0.1% Triton-X 100 and 0.5% bovine serum albumin (PBST). The cells were then incubated with anti-poly(ADP-ribose) polymerase-1 antibody (C-II-10, obtained from G.G. Poirier, 500-fold dilution in PBST) for 2 h at room temperature, followed by three washes with PBS for 15 min each. The cells were then incubated with secondary antibody, anti-mouse Alexa 647 (Molecular Probes, 1000-fold dilution in PBST) for 45 min at room temperature, followed by three washes with PBST for 15 min each. These cells were then incubated with anti-ADP-ribose polymer antibody (PL-90-10, obtained from G.G. Poirier, 100-fold dilution in PBST) for 2 h at room temperature, followed by three washes with PBST for 15 min each, and incubation with secondary antibody, anti-rabbit Alexa 488 (Molecular Probes, 1000-fold dilution in PBST) for 45 min at room temperature. The cells were subsequently washed three times with PBS and treated with DAPI by diluting two drops of NucBlue Fixed Cell ReadyProbes Reagent (Molecular Probes) with 1 ml Carbo-Free Blocking Solution (×1)(Vector Laboratories) for 15 min at room temperature, followed by three further washes with PBS. Cells were visualized using DIC imaging and fluorescence confocal imaging using a relevant wavelength of laser to excite Alexa 647 or Alexa 488. MNNG-induced ADP-ribose polymer formation was detected following exposure of the cells to MNNG at doses >2 μM (S8B Fig).

### Determination of cell doubling times

Time points at which BD or MD occurred were determined by tracking of individual cells as described in the “Single-cell tracking”, and we used the cell-lineage database that was created by the tracking to determine cell doubling time of individual cells. The time when a cell was produced by cell division (Time A) and the time when the same cell produced daughter cells (Time B) were determined, and cell doubling time was calculated by subtracting Time A from Time B.

### Data analysis

Cell lineage maps shown in S1–S4 Figs illustrate the growth profiles of each cell lineage, and maps allow determination of the numbers of cells that can found at each time point. In the analysis of the rate of cell population expansion, the numbers of cells of each lineage at all time points were determined, and each time point of e.g. Control cells, was thus composed of 330 data points. Then, the numbers were normalized to the numbers of cell lineages. These normalized data were used for statistical analysis. With regard to the analysis of cellular events, e.g. M, BD, MD, CF, and CD, the numbers of events found in each cell lineage were determined. Then, the numbers were normalized to the total numbers of cells to perform statistical analysis.

### Statistical analyses of cell doubling times

Cell-doubling times were analyzed by Welch’s *t*-tests (unpaired and two-tailed) or one-way ANOVA (Tukey’s multiple comparison test) using Prism 7.

### Statistical analyses of cell growth

The numbers of progenitor cells and/or progeny of each cell lineage found at 2000, 4000, 6000 and 8500 min were determined. The data was analyzed by one-way ANOVA (Tukey’s multiple comparison test) using Prism 7.

### Statistical analyses of cellular events

The numbers of BD, MD, CD, and CF that occurred in each cell lineage were determined. The data was analyzed by Welch’s *t*-tests (unpaired and two-tailed) or one-way ANOVA (Tukey’s multiple comparison test) using Prism 7.

## Supporting information

S1 Fig Cell lineage maps (Control). Cell lineage maps (Control) are shown. Light blue circle: mitosis, orange circle: cell fusion, pink square: cell death, blue square: incomplete cell division, black vertical line: bipolar cell division, red vertical line: multipolar cell division, and orange vertical line: cell fusion.
(PDF)

S2 Fig Cell lineage maps (MNNG-1μM). Cell lineage maps (MNNG-1μM: MNNG1) are shown. Light blue circle: mitosis, orange circle: cell fusion, pink square: cell death, blue square: incomplete cell division, black vertical line: bipolar cell division, red vertical line: multipolar cell division, and orange vertical line: cell fusion.
(PDF)

S3 Fig Cell lineage maps (MNNG-2μM). Cell lineage maps (MNNG-2μM; MNNG2) are shown. Light blue circle: mitosis, orange circle: cell fusion, pink square: cell death, blue square: incomplete cell division, black vertical line: bipolar cell division, red vertical line: multipolar cell division, and orange vertical line: cell fusion.
(PDF)

S4 Fig Cell lineage maps (MNNG-5μM). Cell lineage maps (MNNG-5μM:MNNG5) are shown. Light blue circle: mitosis, orange circle: cell fusion, pink square: cell death, blue square: incomplete cell division, black vertical line: bipolar cell division, red vertical line: multipolar cell division, and orange vertical line: cell fusion.
(PDF)

S5 Fig Outline of single-cell tracking using morphological observation and classification of cellular events. **A**. Individual cells recorded in a live cell imaging video were identified (represented by different color of circles) and tracked visually as indicated by arrows. **B**. List of categorized cellular events, M, BD, MD, CF, and CD, are shown. Tripolar cell division is shown as an example of MD.
(PDF)

S6 Fig Schematic illustrations of cellular events and patterns of CD inductions. Schematic illustrations of cellular events, i.e. BD, CF, CD and MD, are shown in the upper panel. The patterns of cell death induction listed in Table 1 are shown in the lower panel.
(PDF)

S7 Fig Overview of processes leading to CD. Results shown in Table 1 are illustrated schematically using pie charts. The left side of the pie chart corresponds to data shown in Table 1 (MNNG-5μM). The right side of the pie charts (MNNG-2μM, MNNG-1μM and Control) correspond to data shown in Table 1 (MNNG-2μM, MNNG-1μM and Control). The right side of the pie chart (MNNG-5μM) was created using the same categorization that was used for MNNG-2μM, MNNG-1μM and Control.
(PDF)

S8 Fig Growth of cells exposed to various doses of MNNG in CO_2_ incubator and MNNG-induced ADP ribose polymer formation. **A**. Numbers of cells were determined every 24 h (n = 6). The initial numbers of cells were normalized by 100. One-way ANOVA (Tukey’s multiple comparison test) was performed for each time point. The significance of differences between Control and MNNG-1μM, MNNG-2μM, and MNNG-5μM are shown: *p<0.05, **p<0.01, ***p<0.001, and ****p<0.0001. Results shown as the mean ± SEM. **B**. After exposure of cells to various doses of MNNG for 30 min, indirect immunofluorescence was performed using anti-poly(ADP-ribose) polymerase-1 (PARP-1) antibody and anti-ADP-ribose polymer (PAR) antibody. Cells were also stained with DAPI.
(PDF)

S1 Movie A time-lapse movie of HeLa cells (Control). A time-lapse movie (time 1–8500 min, one FOV) of HeLa cells (Control) is shown. BD and CD occurred constantly throughout the observation period in Control cells, while BD occurred predominantly (Fig 4A and 4B), resulting in constant expansion of the cell population (Fig 2A).
(MOV)

S2 Movie A time-lapse movie of HeLa cells (MNNG-1μM). A time-lapse movie (time 1–8500 min, one FOV) of HeLa cells (MNNG-1μM) is shown.
(MOV)

S3 Movie A time-lapse movie of HeLa cells (MNNG-2μM). A time-lapse movie (time 1–8500 min, one FOV) of HeLa cells (MNNG-2μM) is shown.
(MOV)

S4 Movie A time-lapse movie of HeLa cells (MNNG-5μM). A time-lapse movie (time 1–8500 min, one FOV) of HeLa cells (MNNG-5μM) is shown.
(MOV)

S5 Movie A time-lapse movie of HeLa cells exposed to MNNG-40μM. A time-lapse movie (time 1–930 min, one FOV) of HeLa cells exposed to MNNG-40μM is shown.
(MOV)
