# Supplementary material for: Dose-dependent spatiotemporal responses of mammalian cells to an alkylating agent
Source: PLoS One. 2019 Mar 29;14(3):e0214512. doi: 10.1371/journal.pone.0214512 (PMC6440626; doi:10.1371/journal.pone.0214512)

Supplementary Figure S1

Analysis: MNNG, Treat.: Control, Cell: HeLa

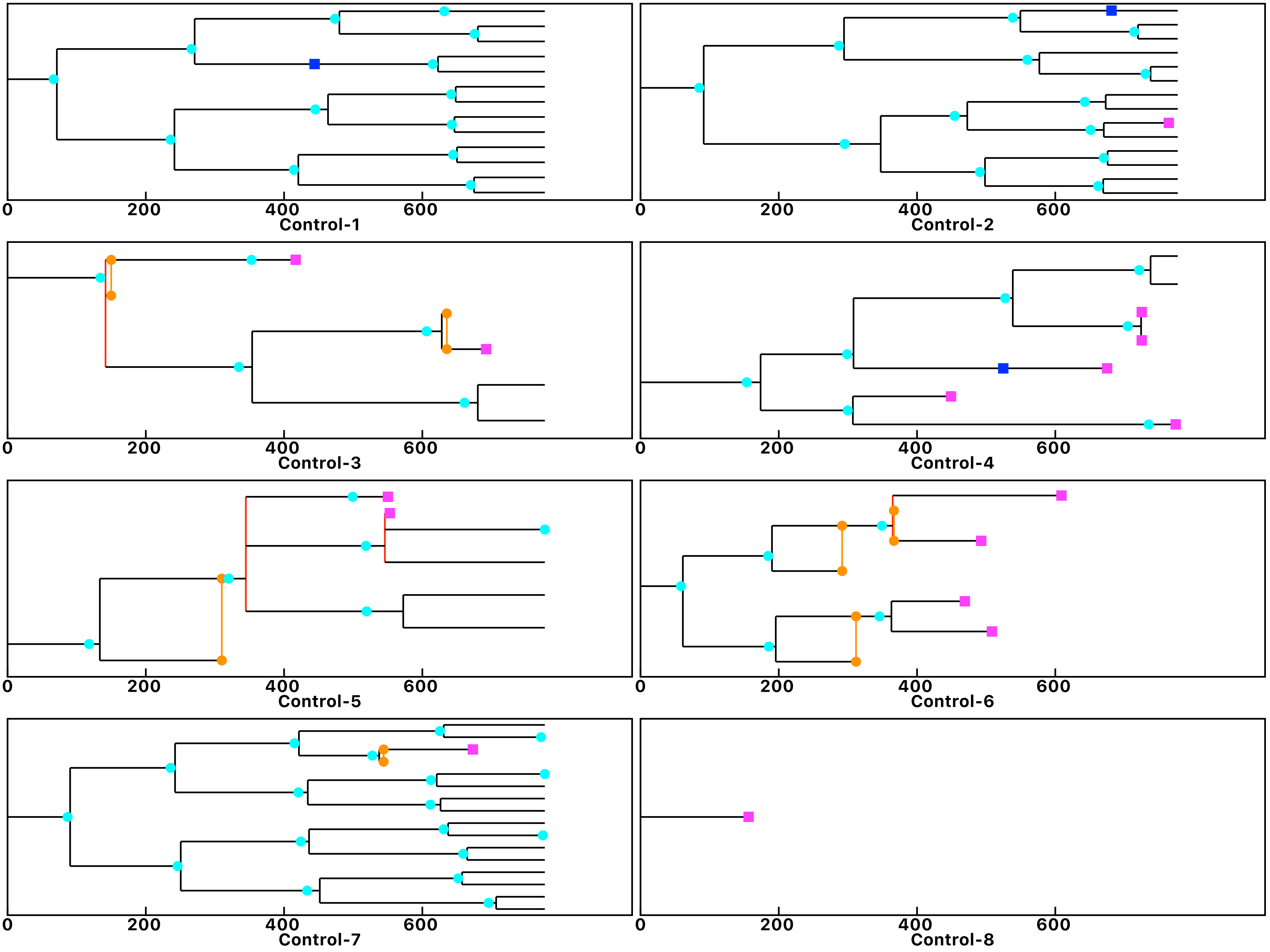

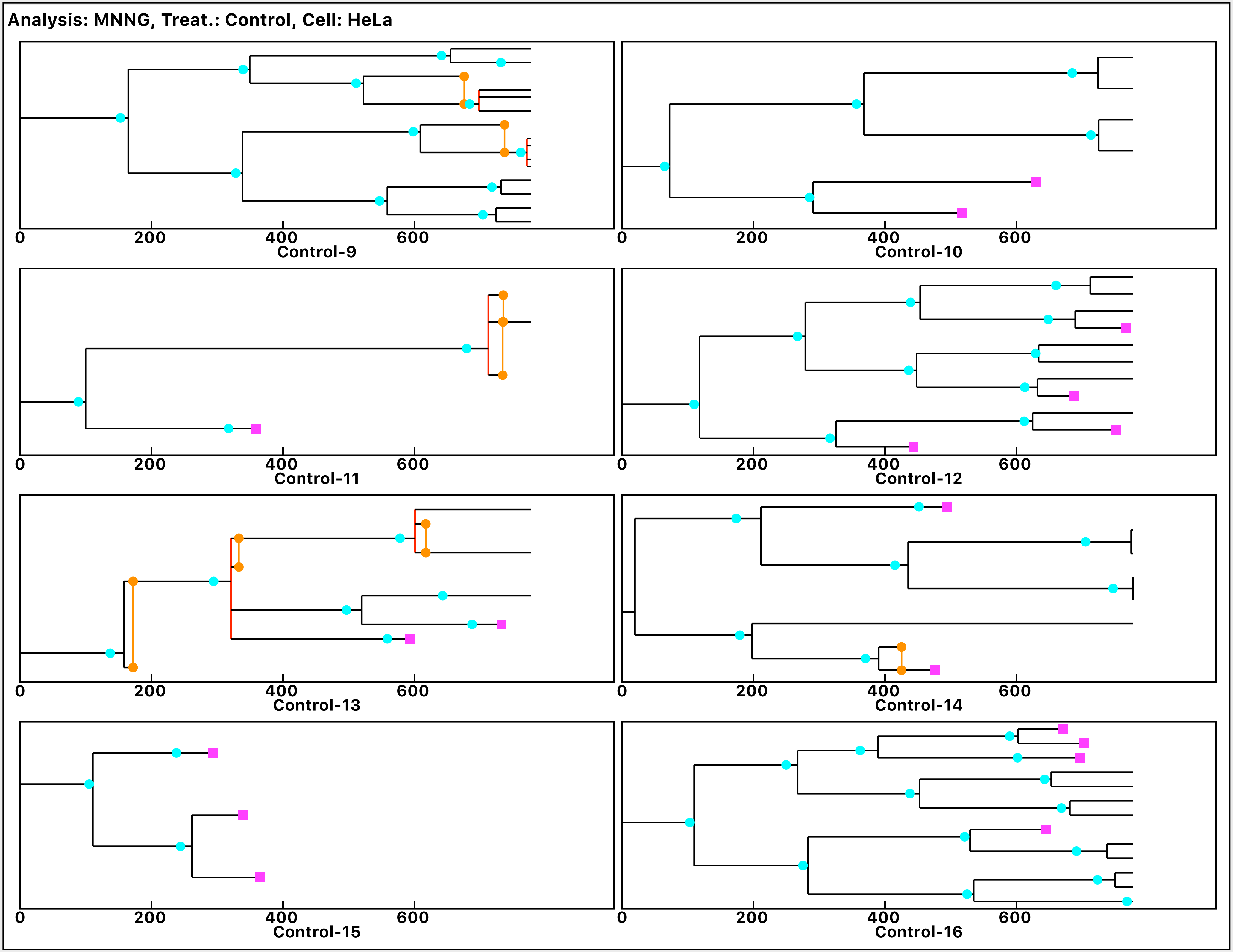

Analysis: MNNG, Treat.: Control, Cell: HeLa
0
200
400
600
Control-9
Control-10
Control-11
Control-12
Control-13
Control-14
Control-15
Control-16

Analysis: MNNG, Treat.: Control, Cell: HeLa

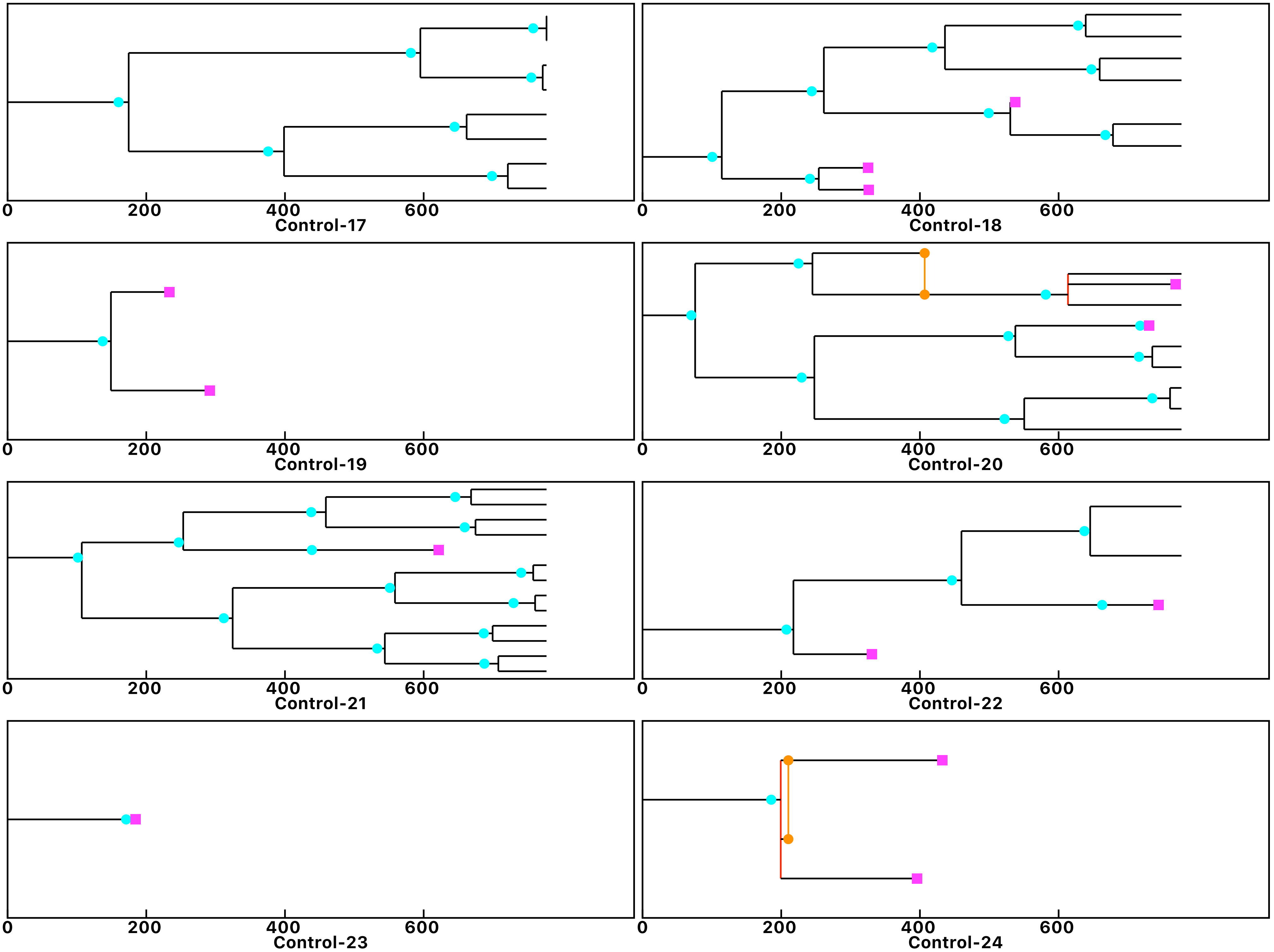

Analysis: MNNG, Treat.: Control, Cell: HeLa

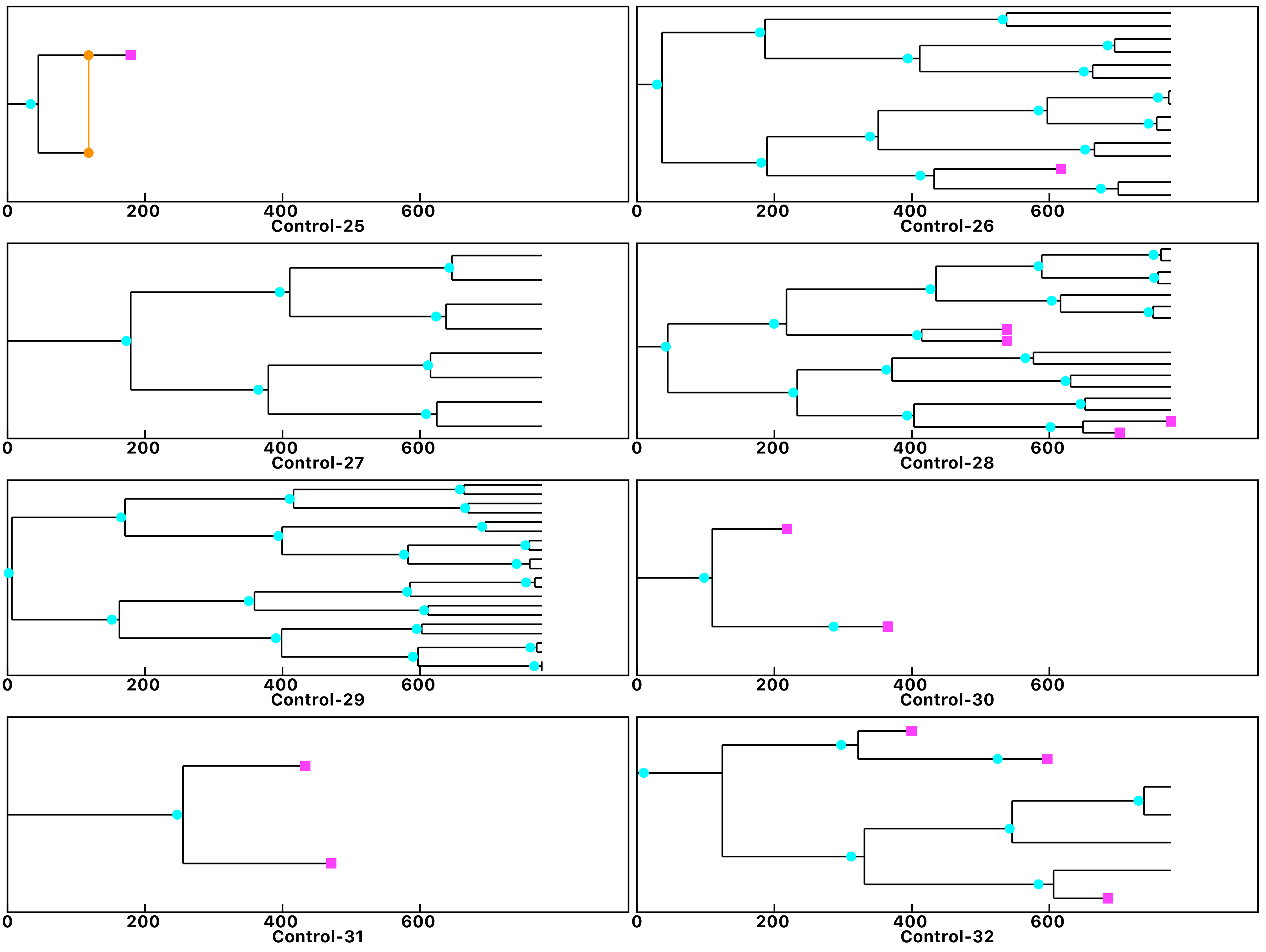

Analysis: MNNG, Treat.: Control, Cell: HeLa

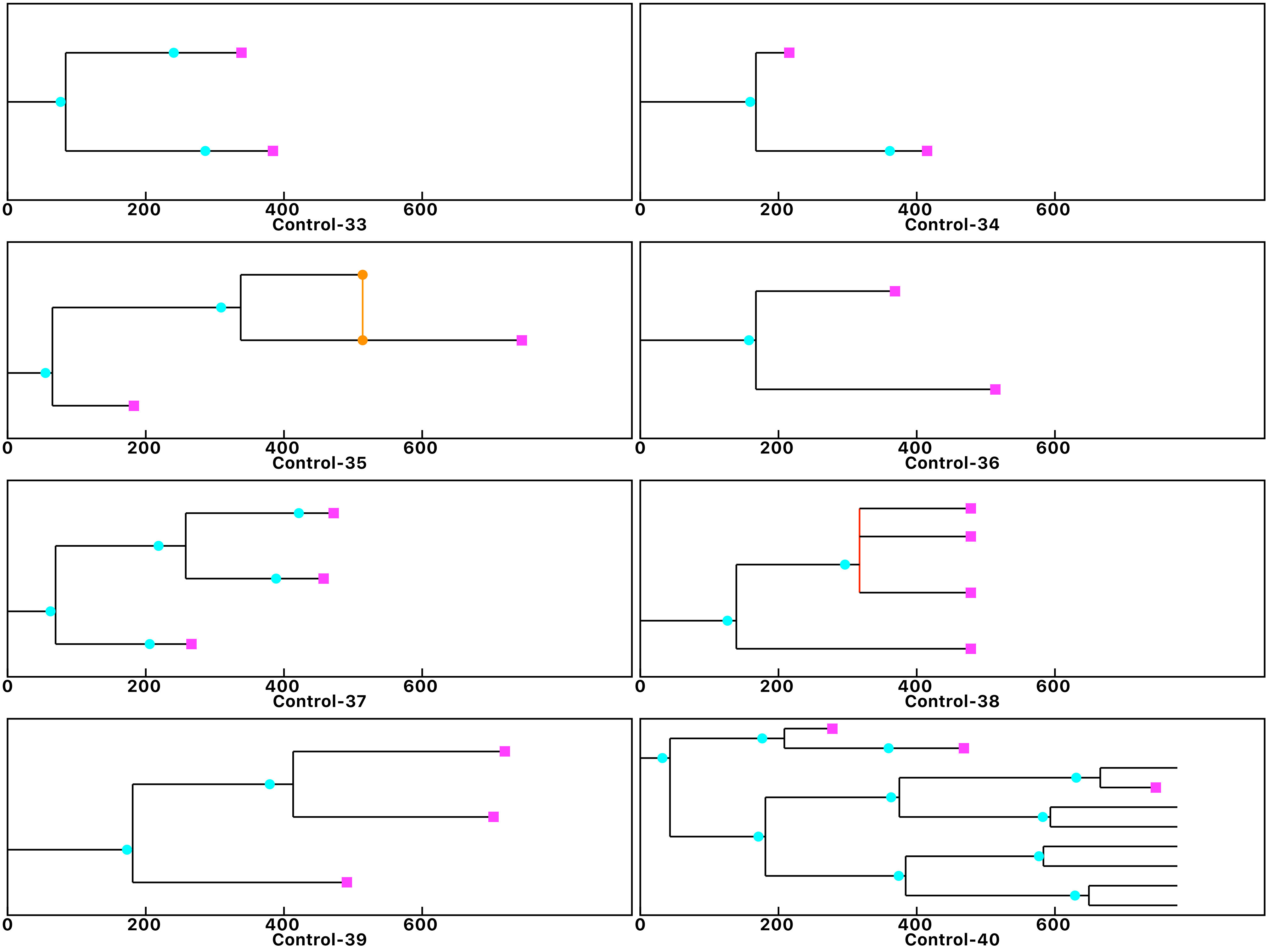

Analysis: MNNG, Treat.: Control, Cell: HeLa

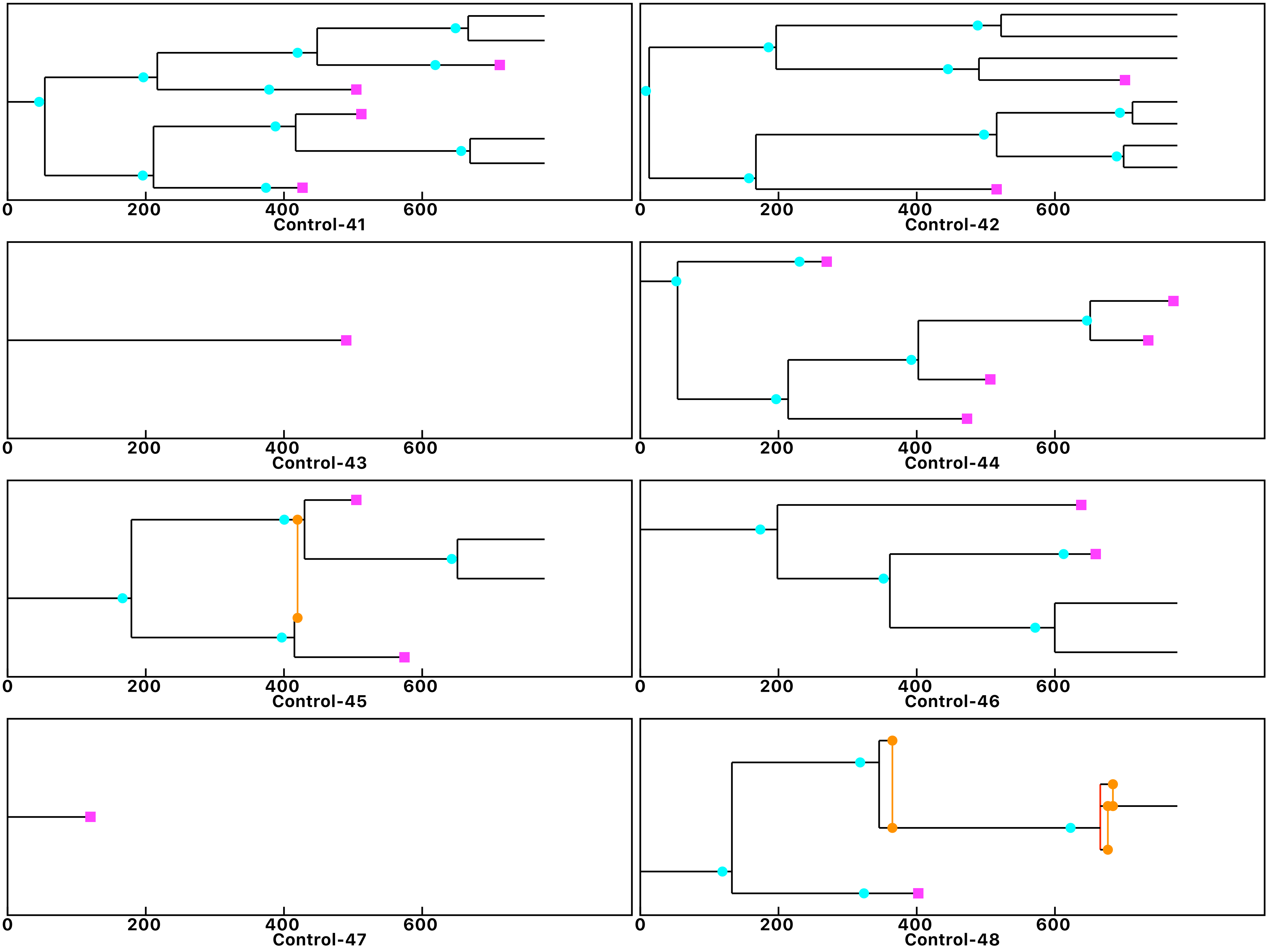

Analysis: MNNG, Treat.: Control, Cell: HeLa

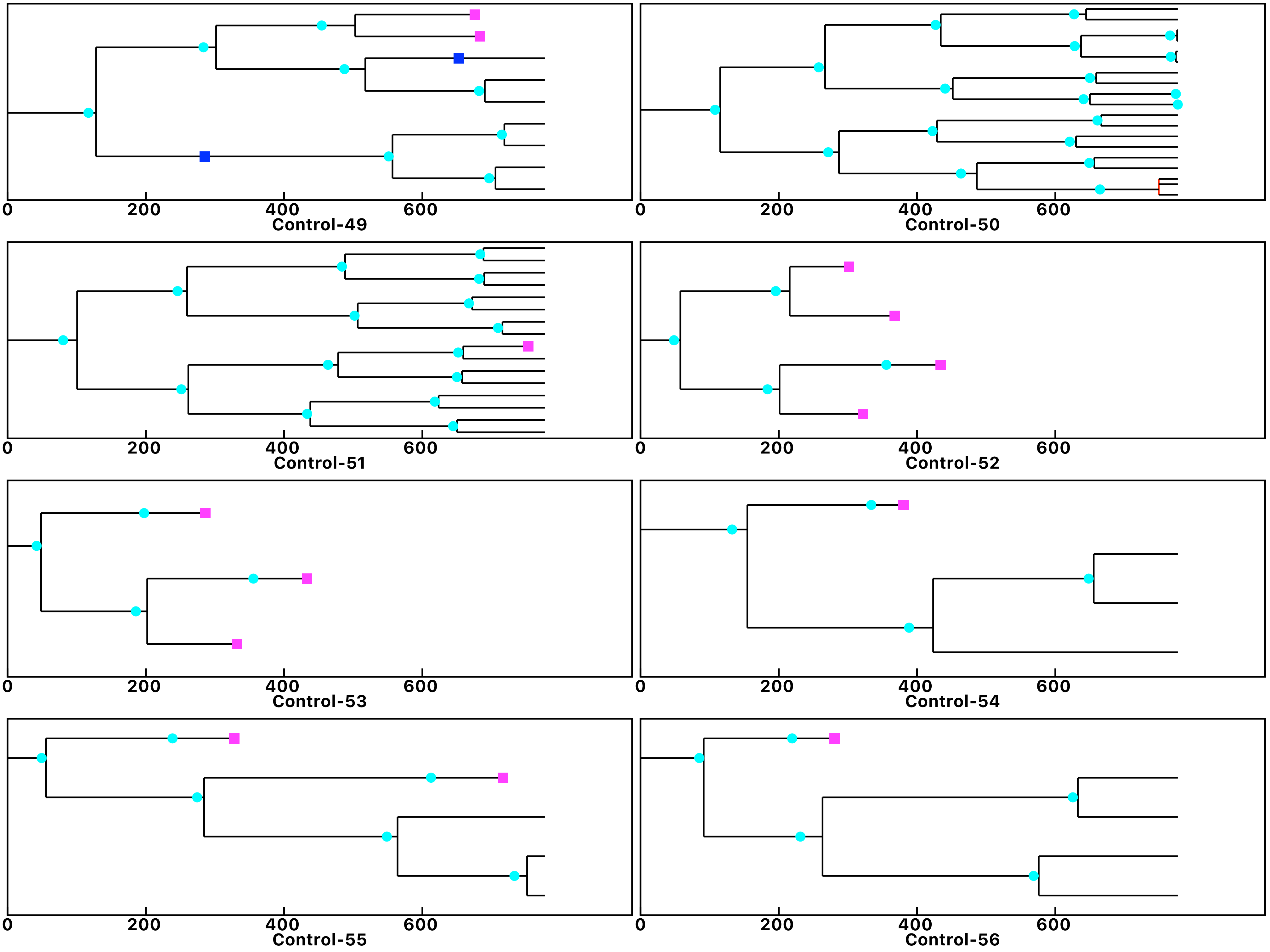

Analysis: MNNG, Treat.: Control, Cell: HeLa

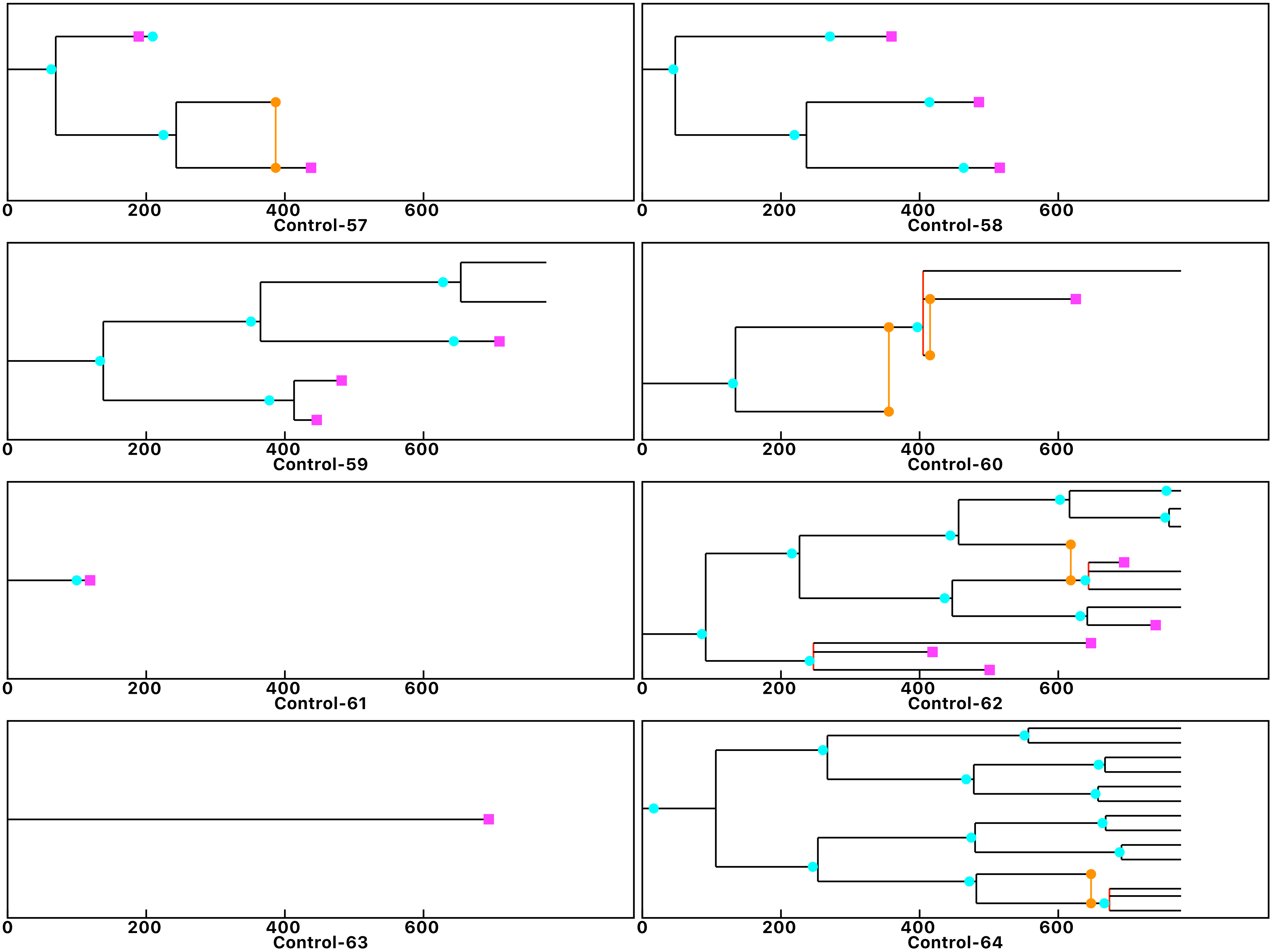

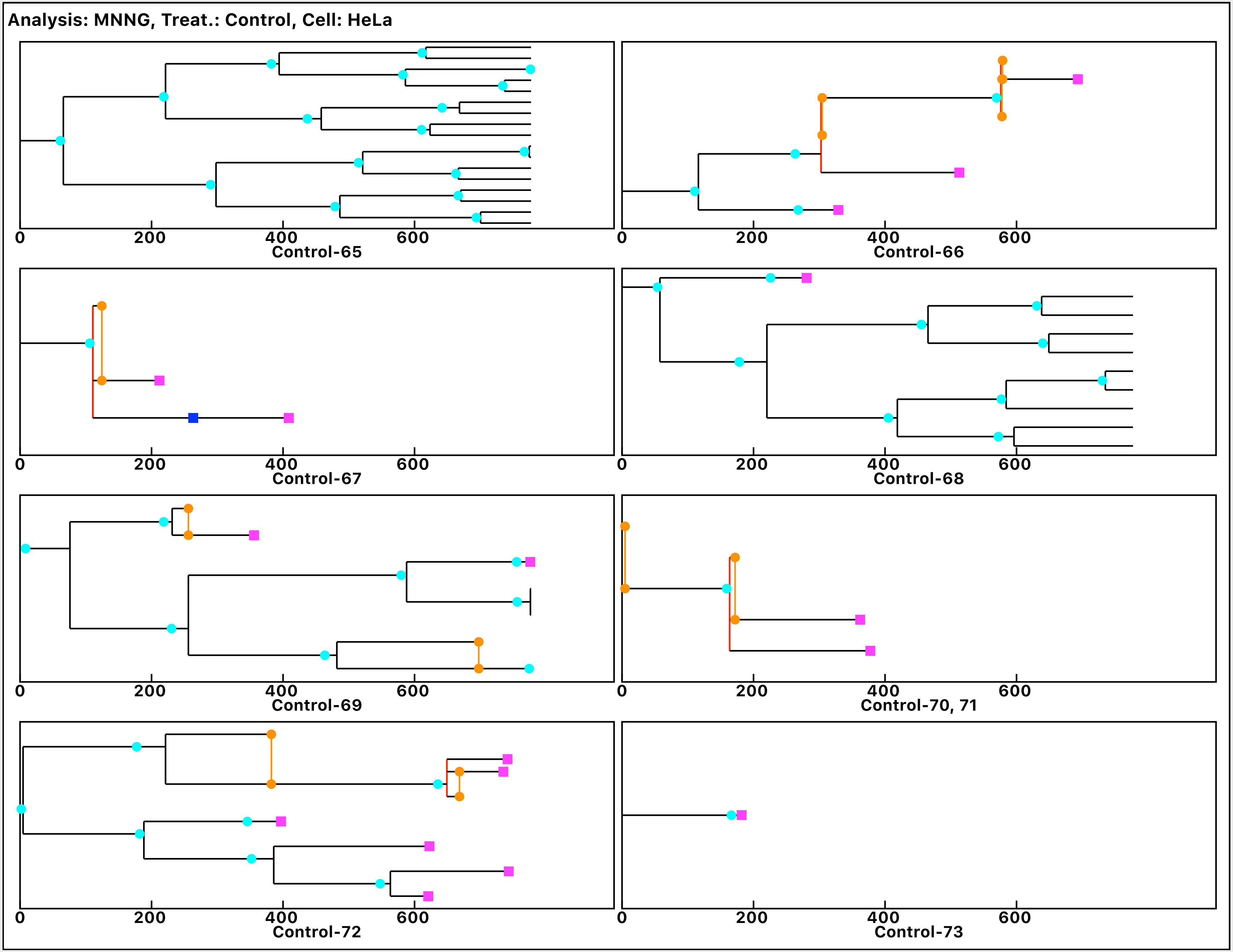

Analysis: MNNG, Treat.: Control, Cell: HeLa
0
200
400
600
Control-65
Control-66
Control-67
Control-68
Control-69
Control-70, 71
Control-72
Control-73

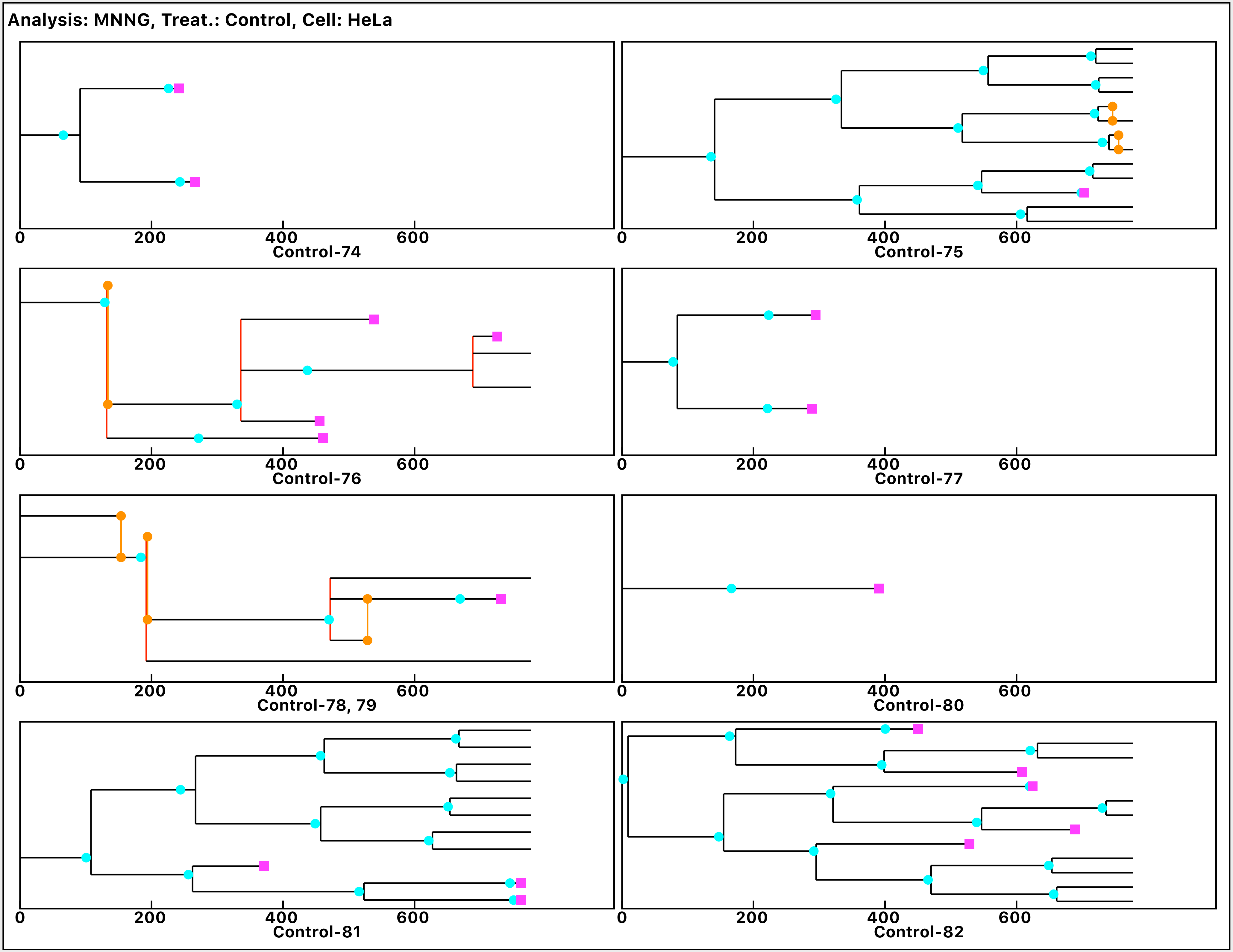

Analysis: MNNG, Treat.: Control, Cell: HeLa
0
200
400
600
Control-74
Control-75
Control-76
Control-77
Control-78, 79
Control-80
Control-81
Control-82

Analysis: MNNG, Treat.: Control, Cell: HeLa

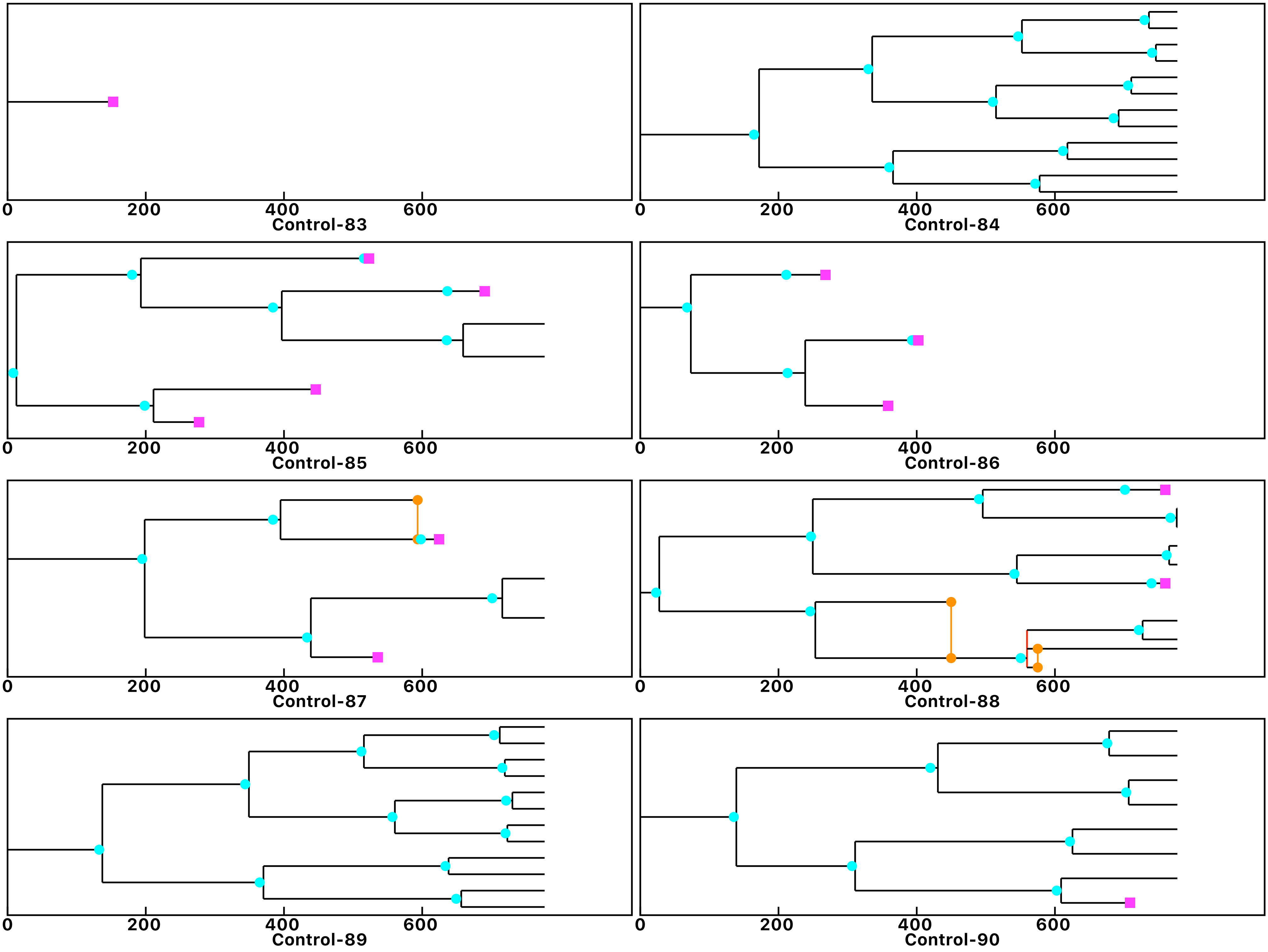

Analysis: MNNG, Treat.: Control, Cell: HeLa

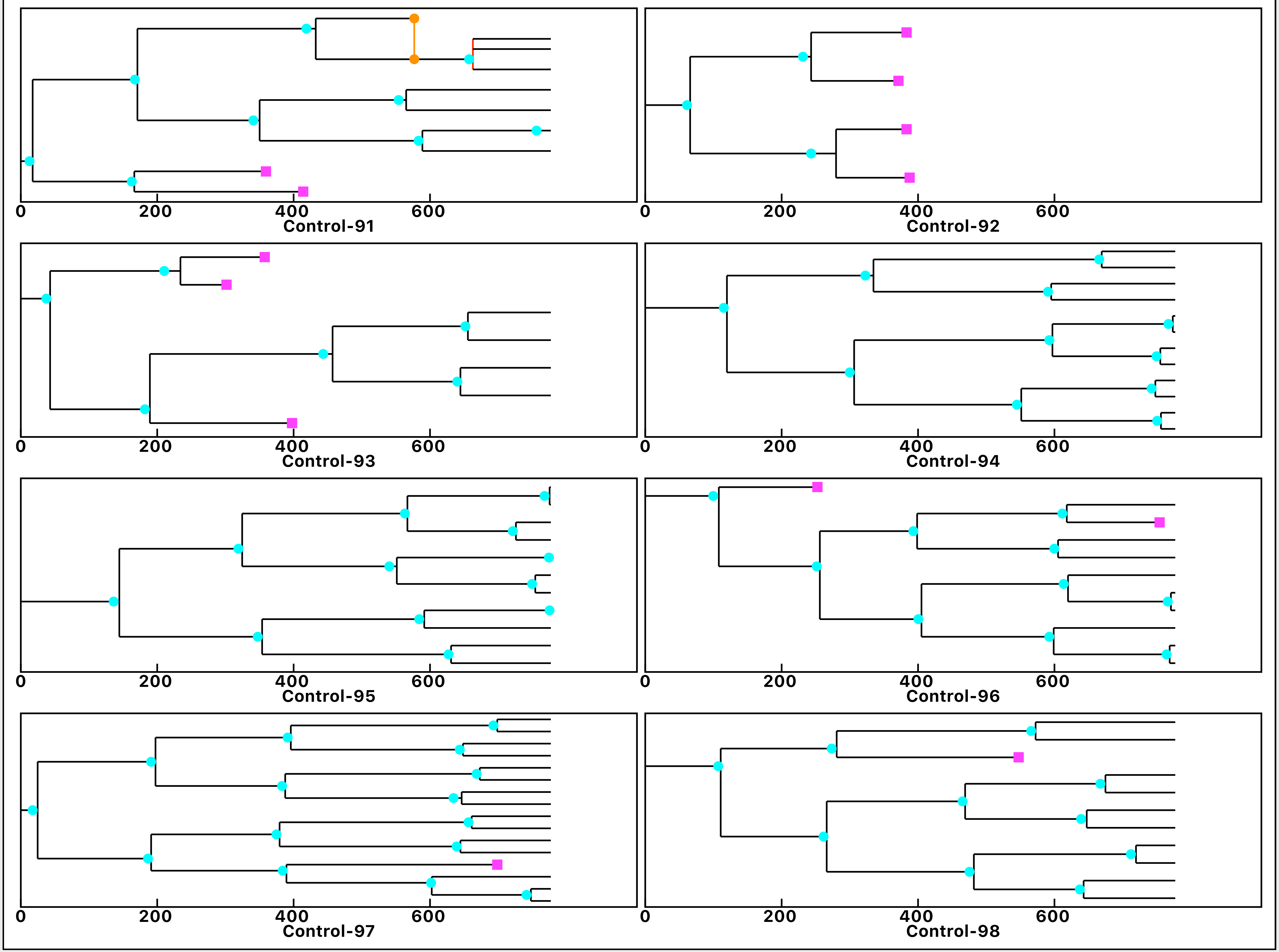

Analysis: MNNG, Treat.: Control, Cell: HeLa

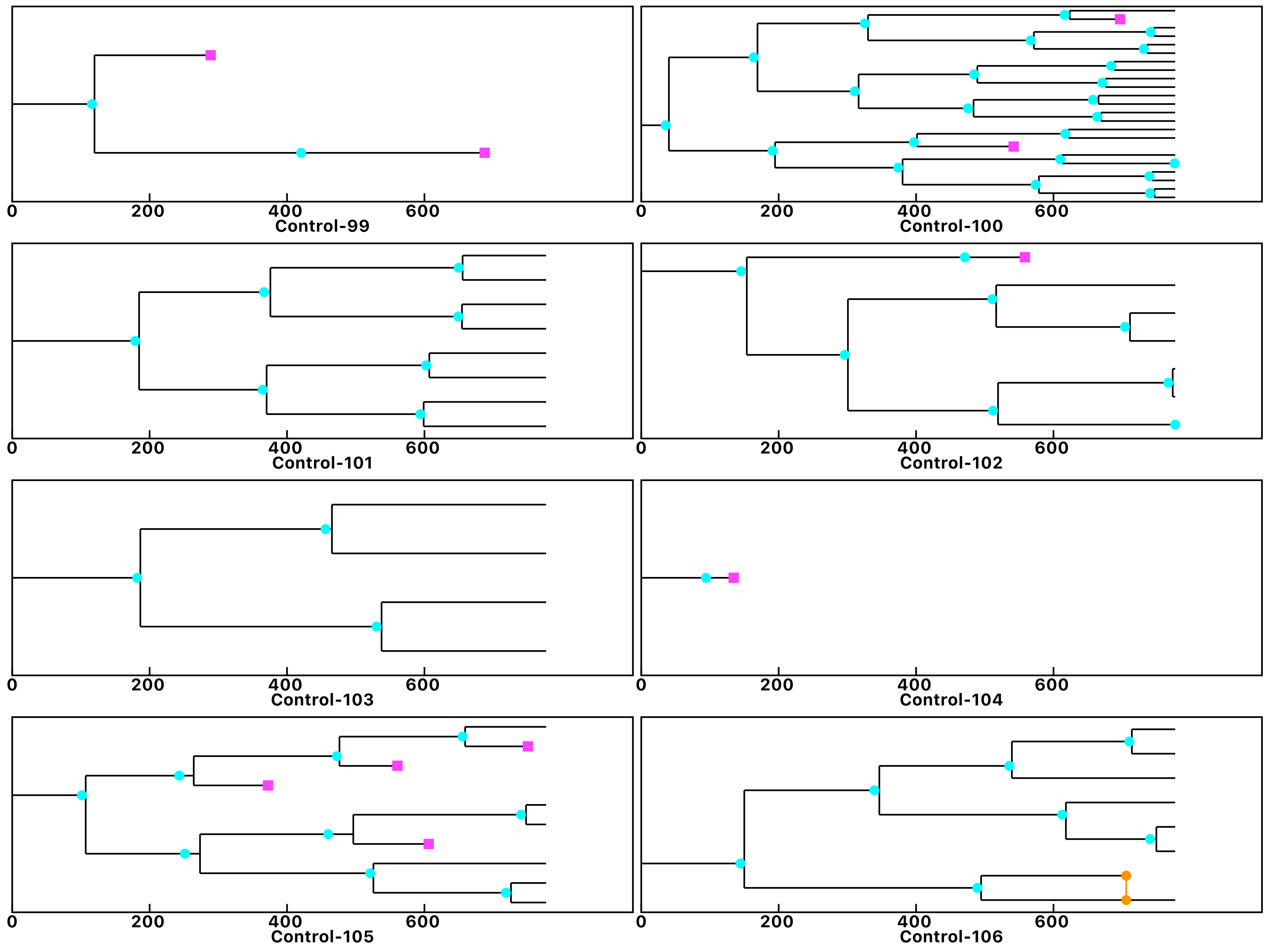

Analysis: MNNG, Treat.: Control, Cell: HeLa

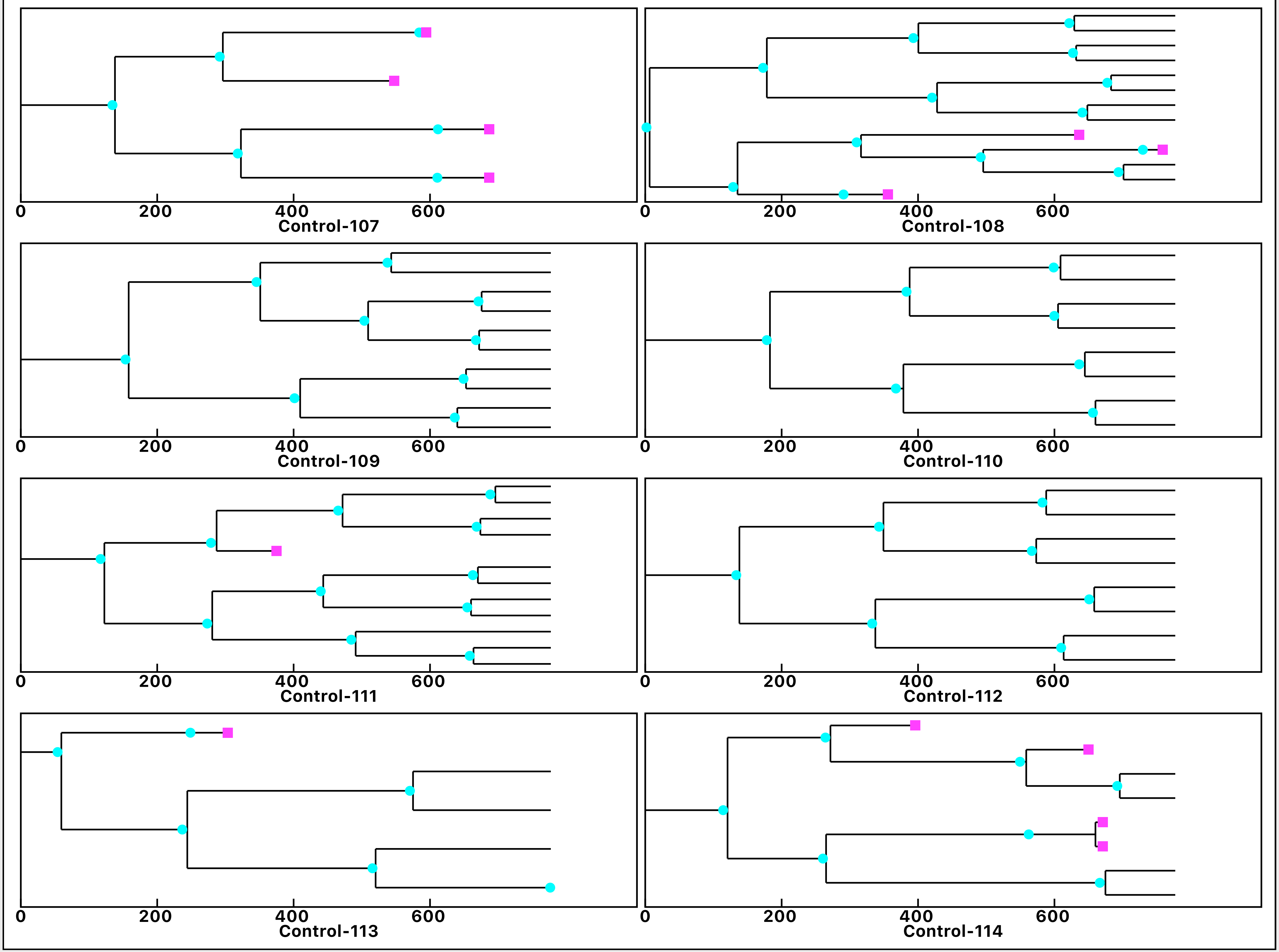

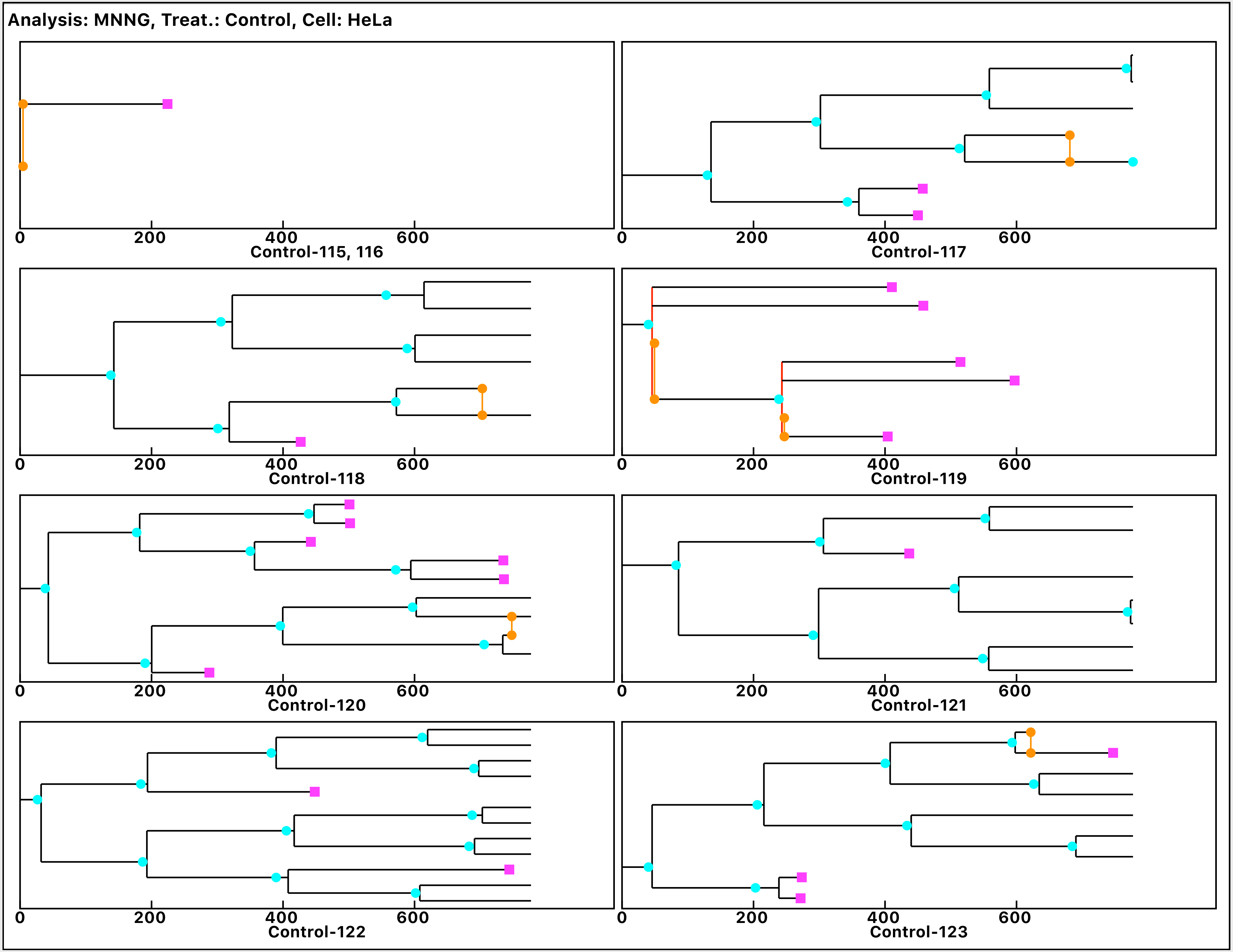

Analysis: MNNG, Treat.: Control, Cell: HeLa
0
200
400
600
Control-115, 116
Control-117
Control-118
Control-119
Control-120
Control-121
Control-122
Control-123

Analysis: MNNG, Treat.: Control, Cell: HeLa

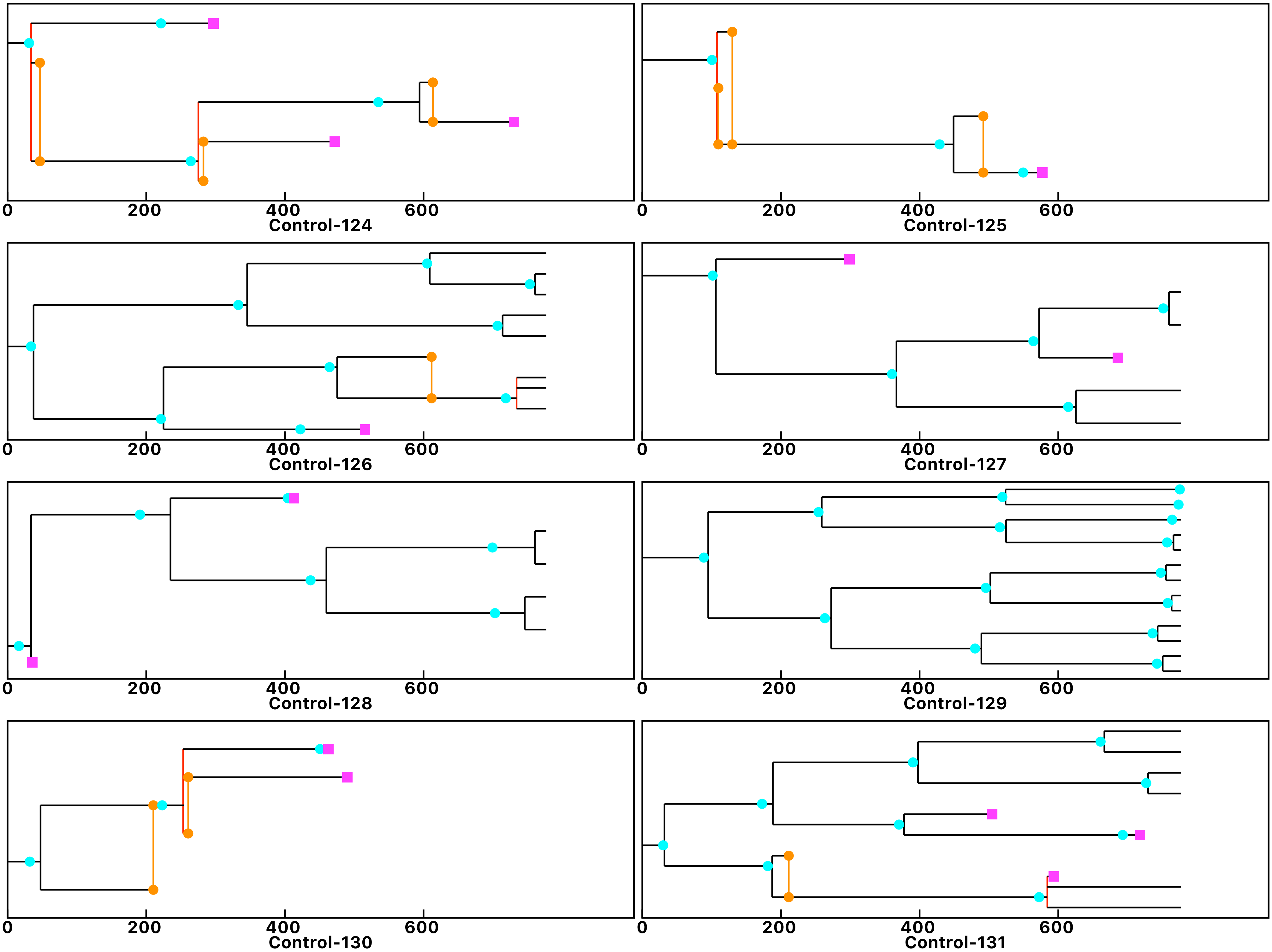

Analysis: MNNG, Treat.: Control, Cell: HeLa

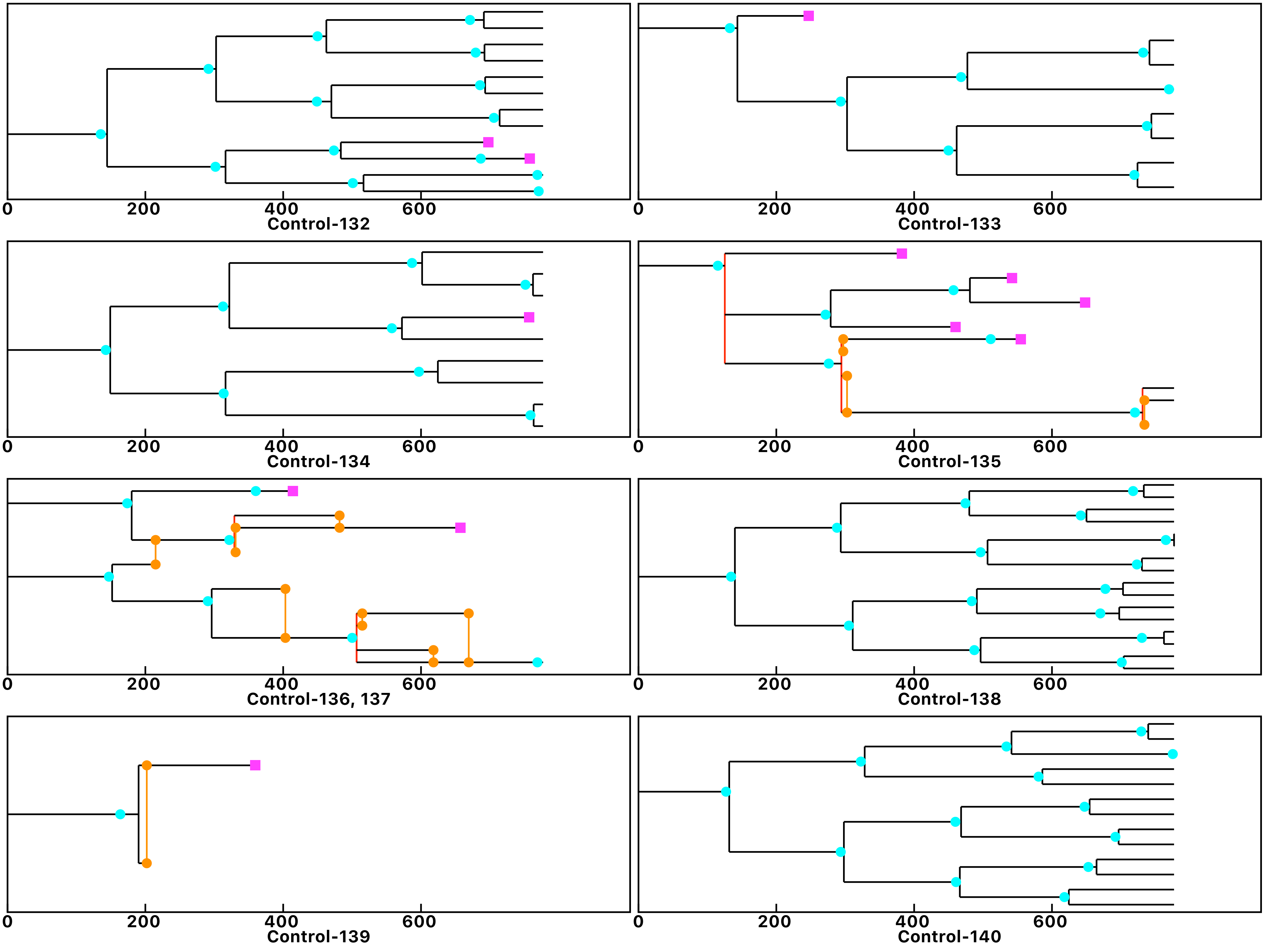

Analysis: MNNG, Treat.: Control, Cell: HeLa

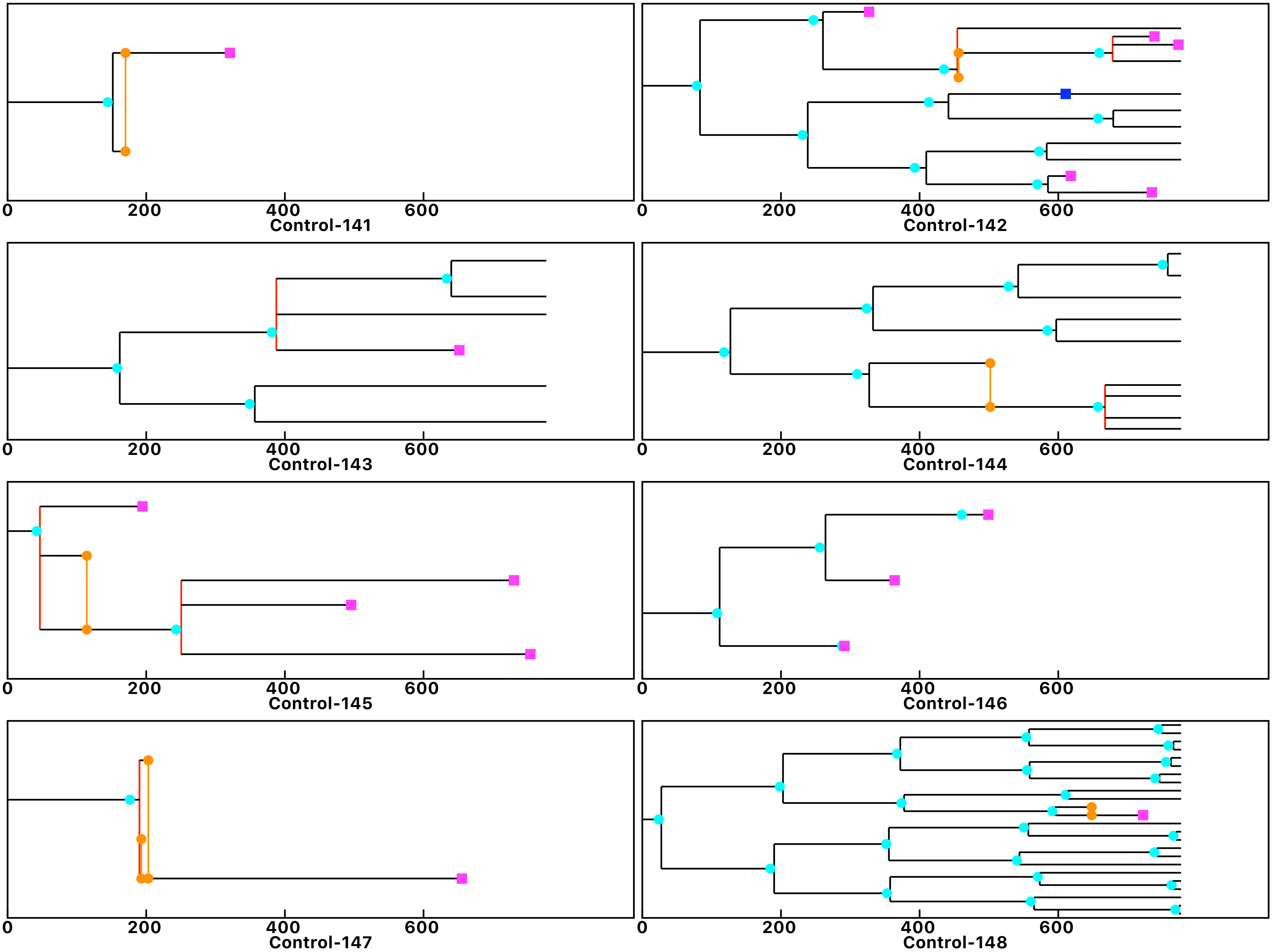

Analysis: MNNG, Treat.: Control, Cell: HeLa

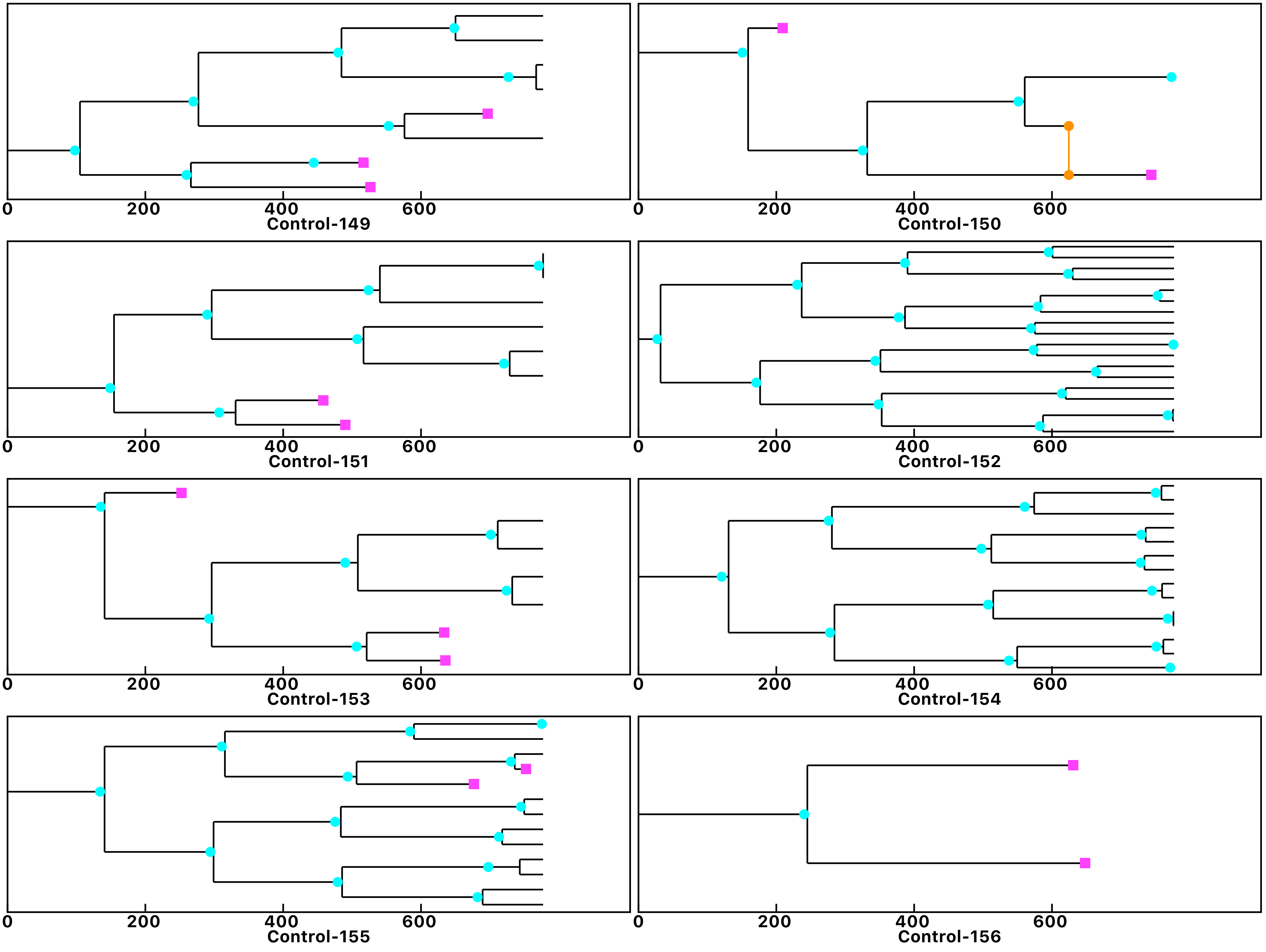

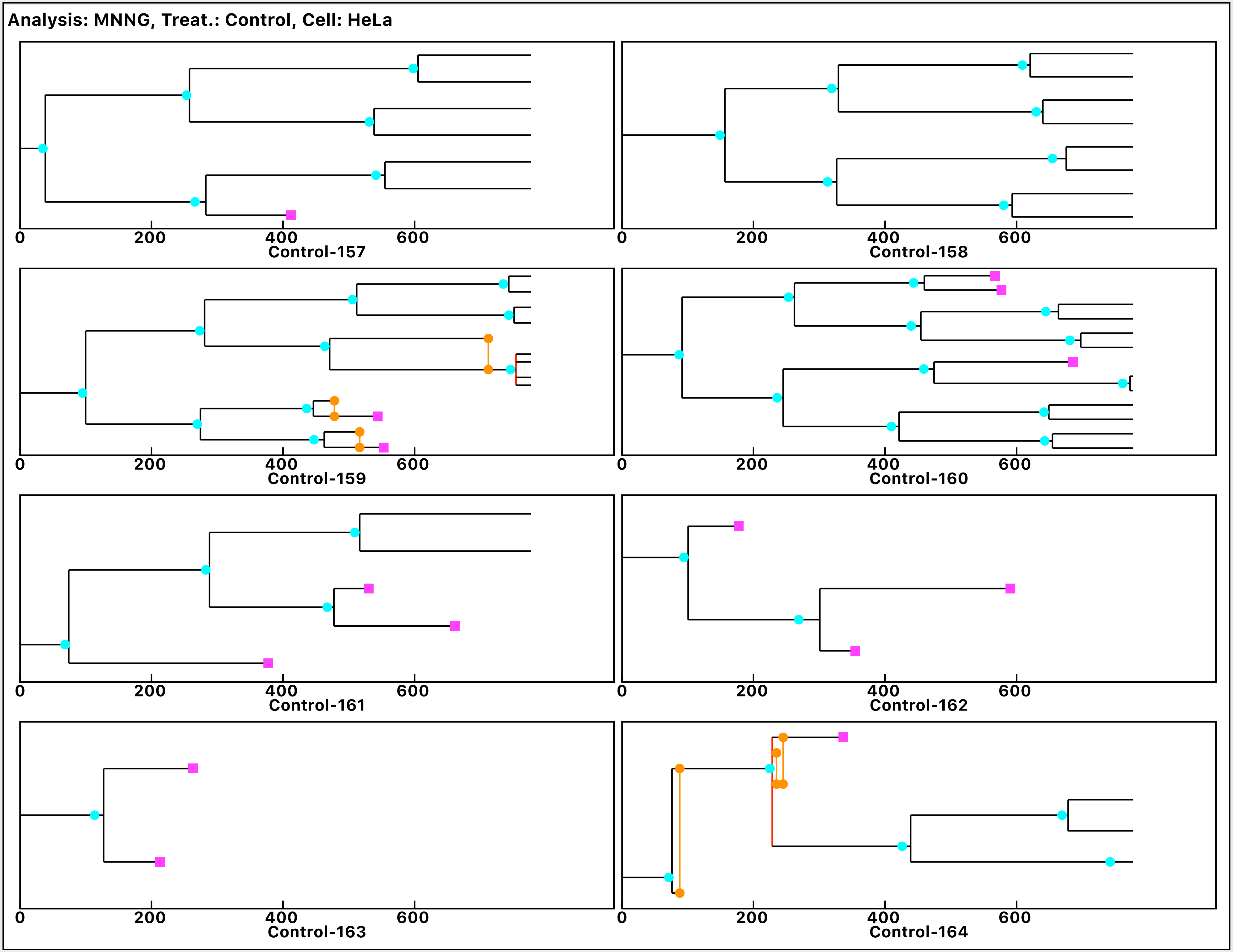

Analysis: MNNG, Treat.: Control, Cell: HeLa
0
200
400
600
Control-157
Control-158
Control-159
Control-160
Control-161
Control-162
Control-163
Control-164

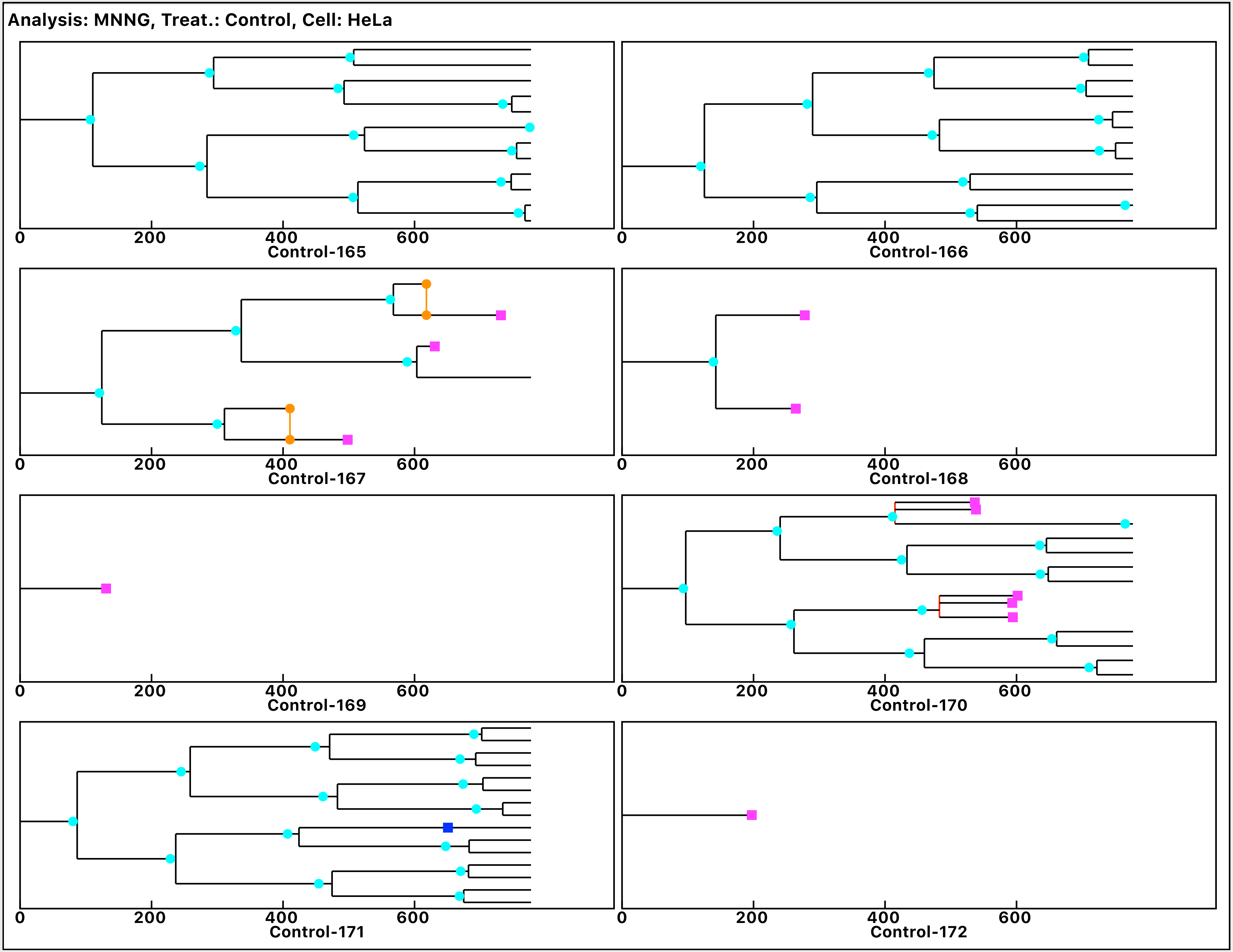
Analysis: MNNG, Treat.: Control, Cell: HeLa
0
200
400
600
Control-165
Control-166
Control-167
Control-168
Control-169
Control-170
Control-171
Control-172

Analysis: MNNG, Treat.: Control, Cell: HeLa

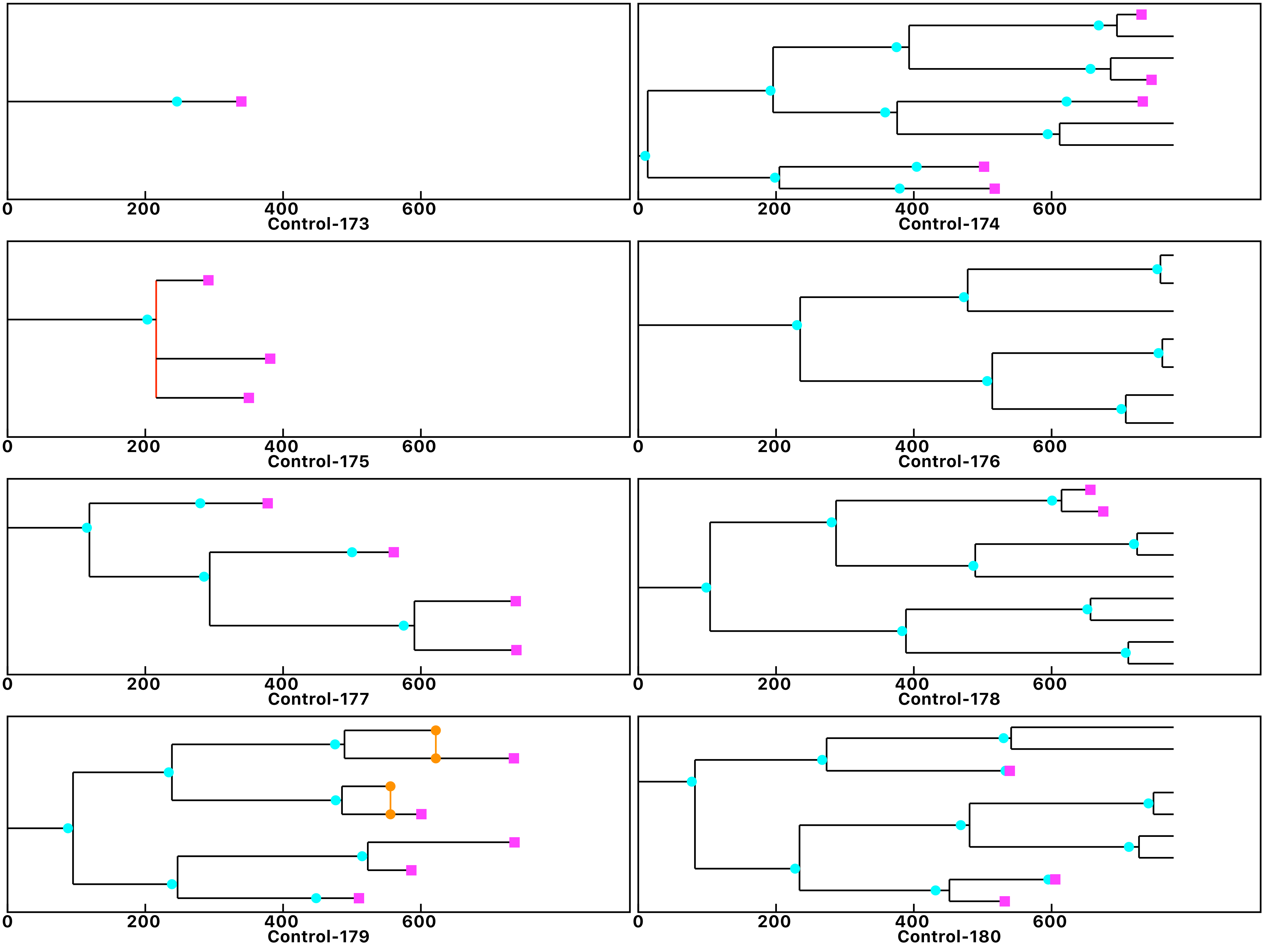

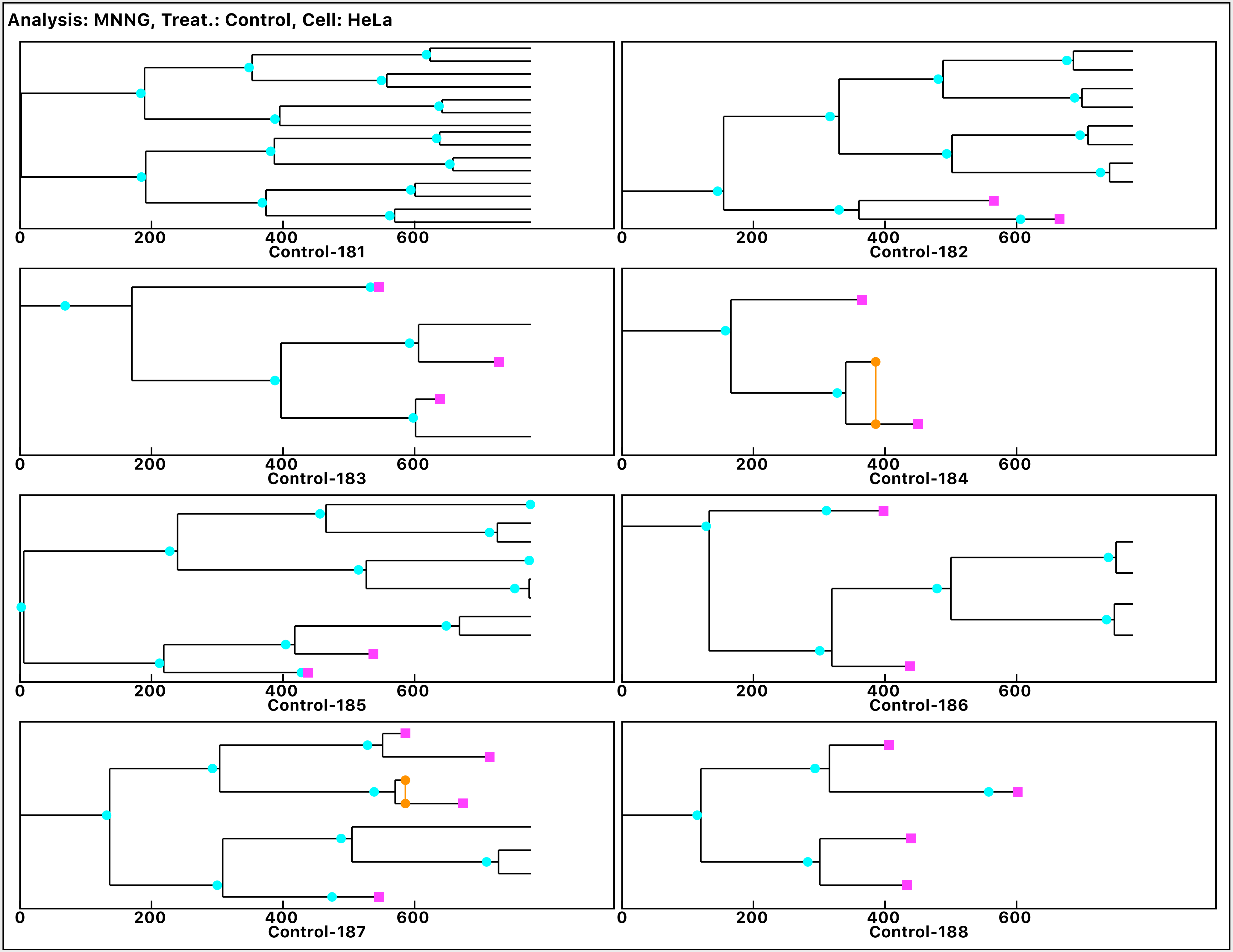
Analysis: MNNG, Treat.: Control, Cell: HeLa
0
200
400
600
Control-181
Control-182
Control-183
Control-184
Control-185
Control-186
Control-187
Control-188

Analysis: MNNG, Treat.: Control, Cell: HeLa

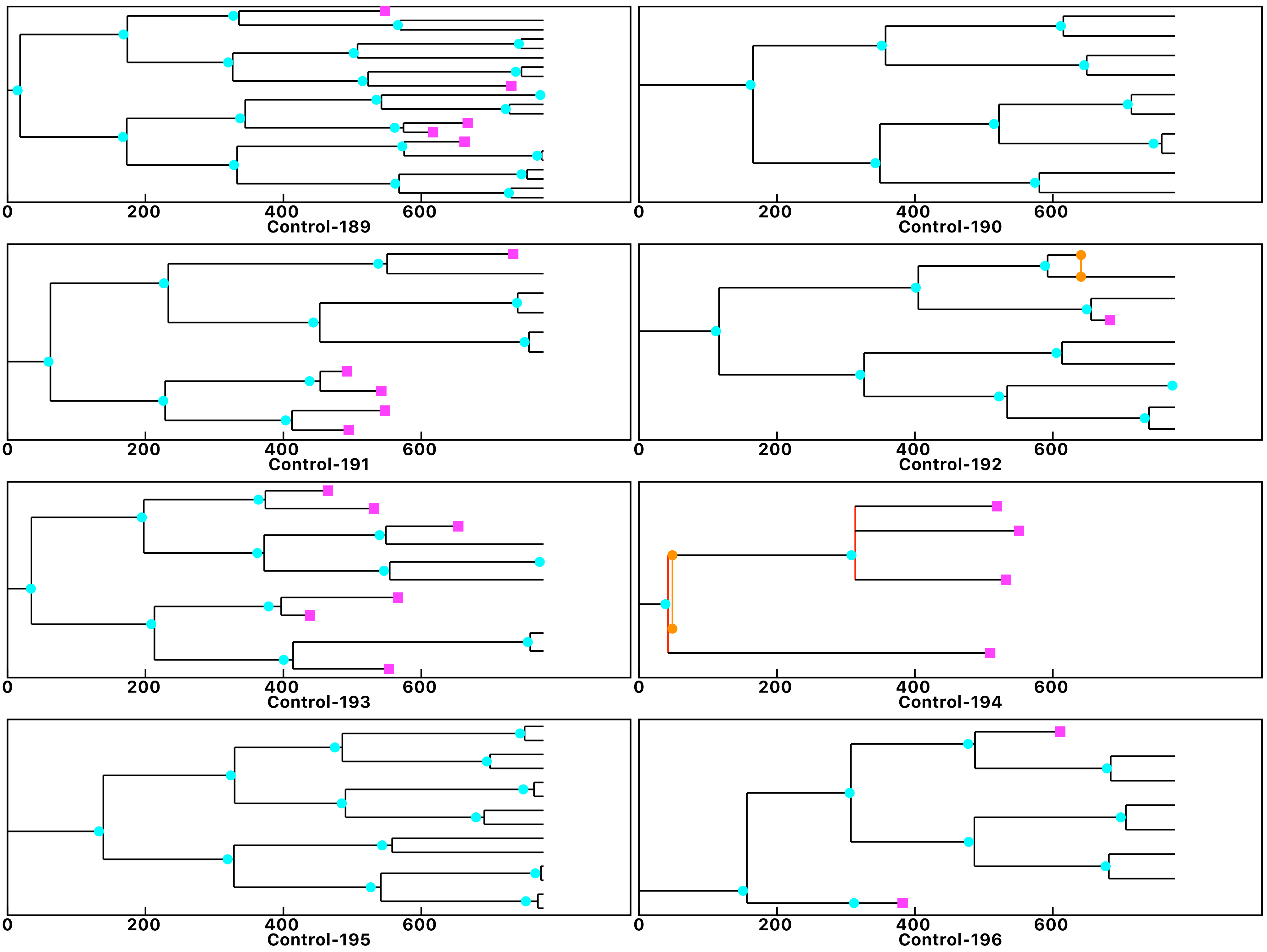

Analysis: MNNG, Treat.: Control, Cell: HeLa

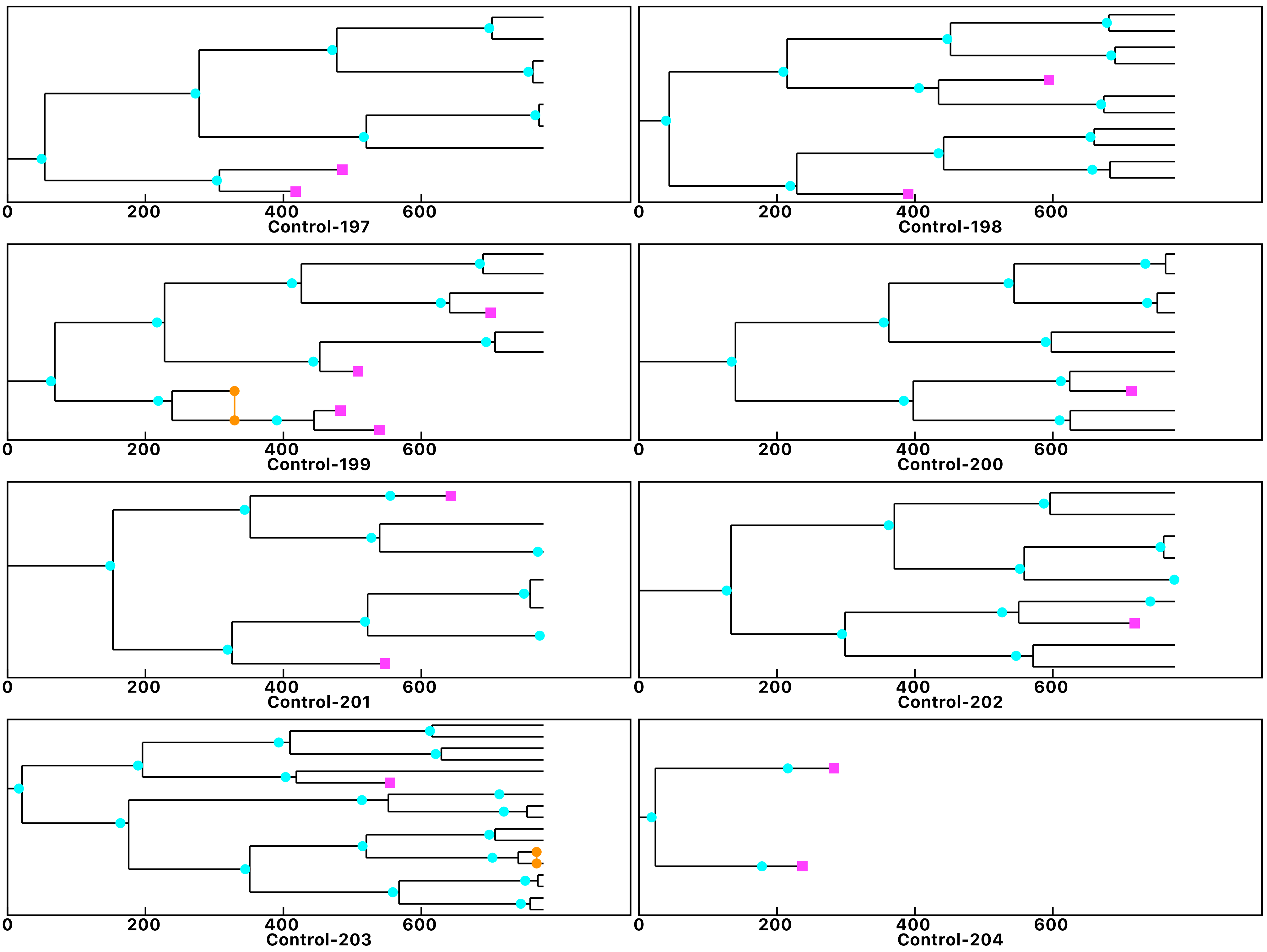

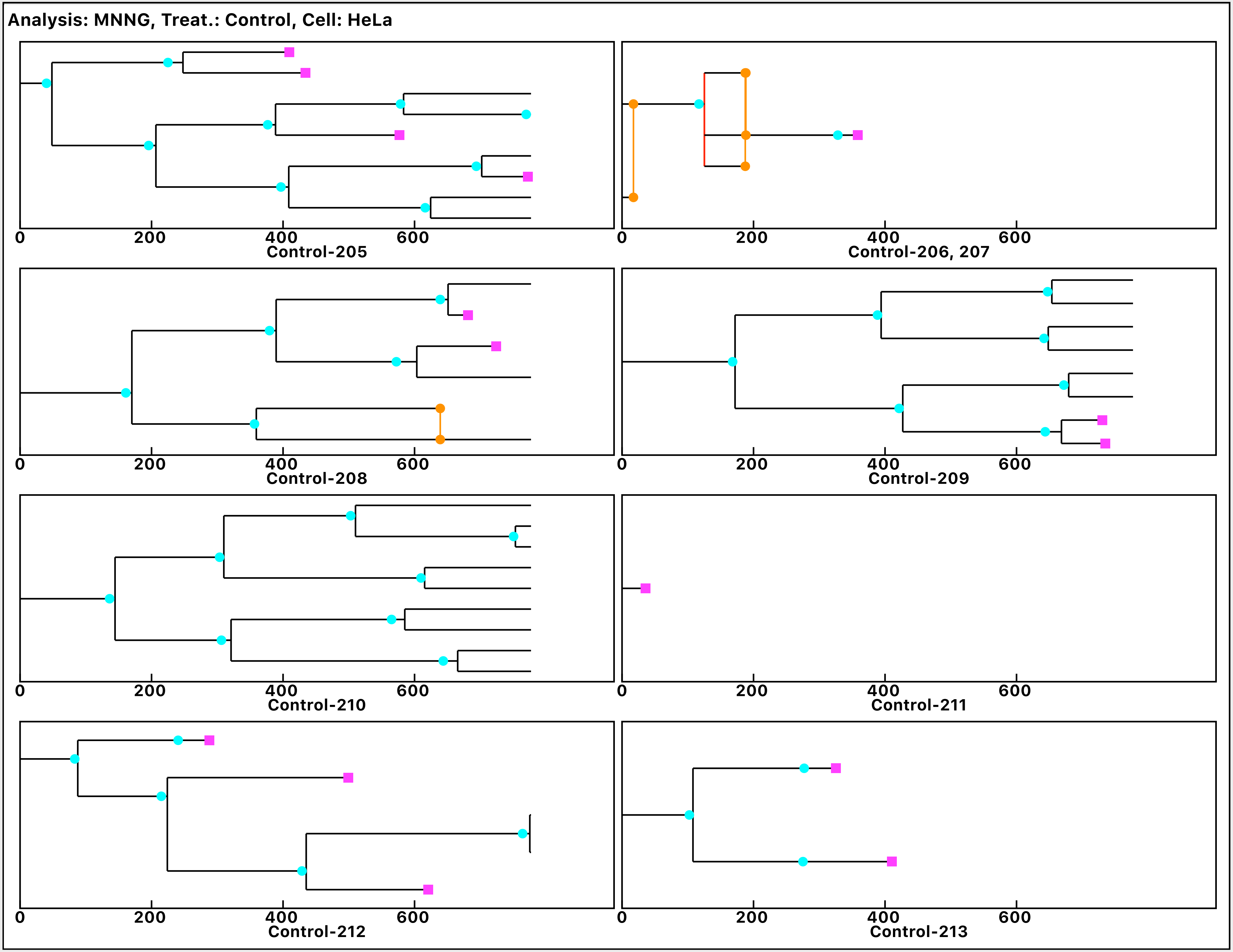
Analysis: MNNG, Treat.: Control, Cell: HeLa
0
200
400
600
Control-205
Control-206, 207
Control-208
Control-209
Control-210
Control-211
Control-212
Control-213

Analysis: MNNG, Treat.: Control, Cell: HeLa

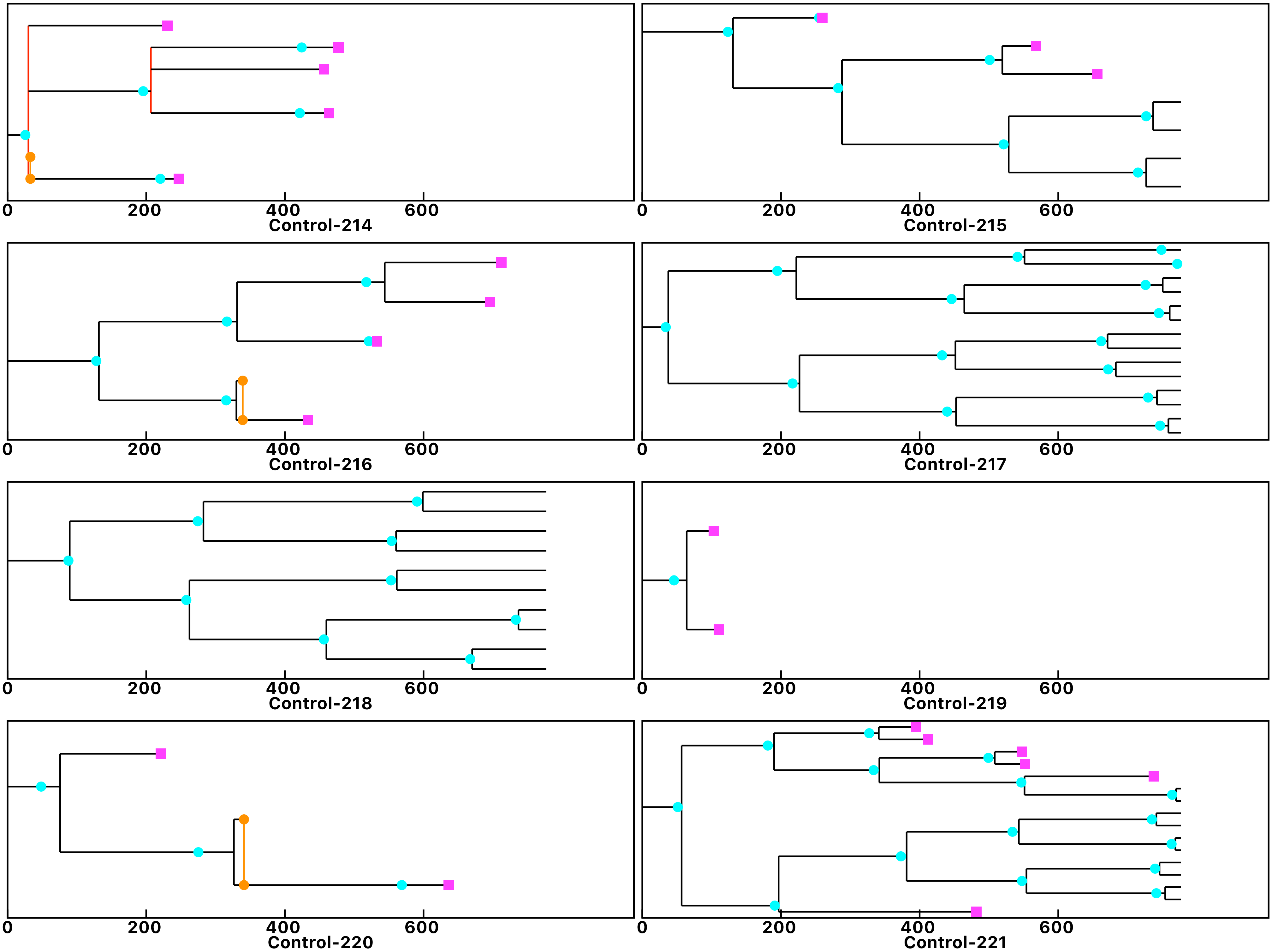

Analysis: MNNG, Treat.: Control, Cell: HeLa

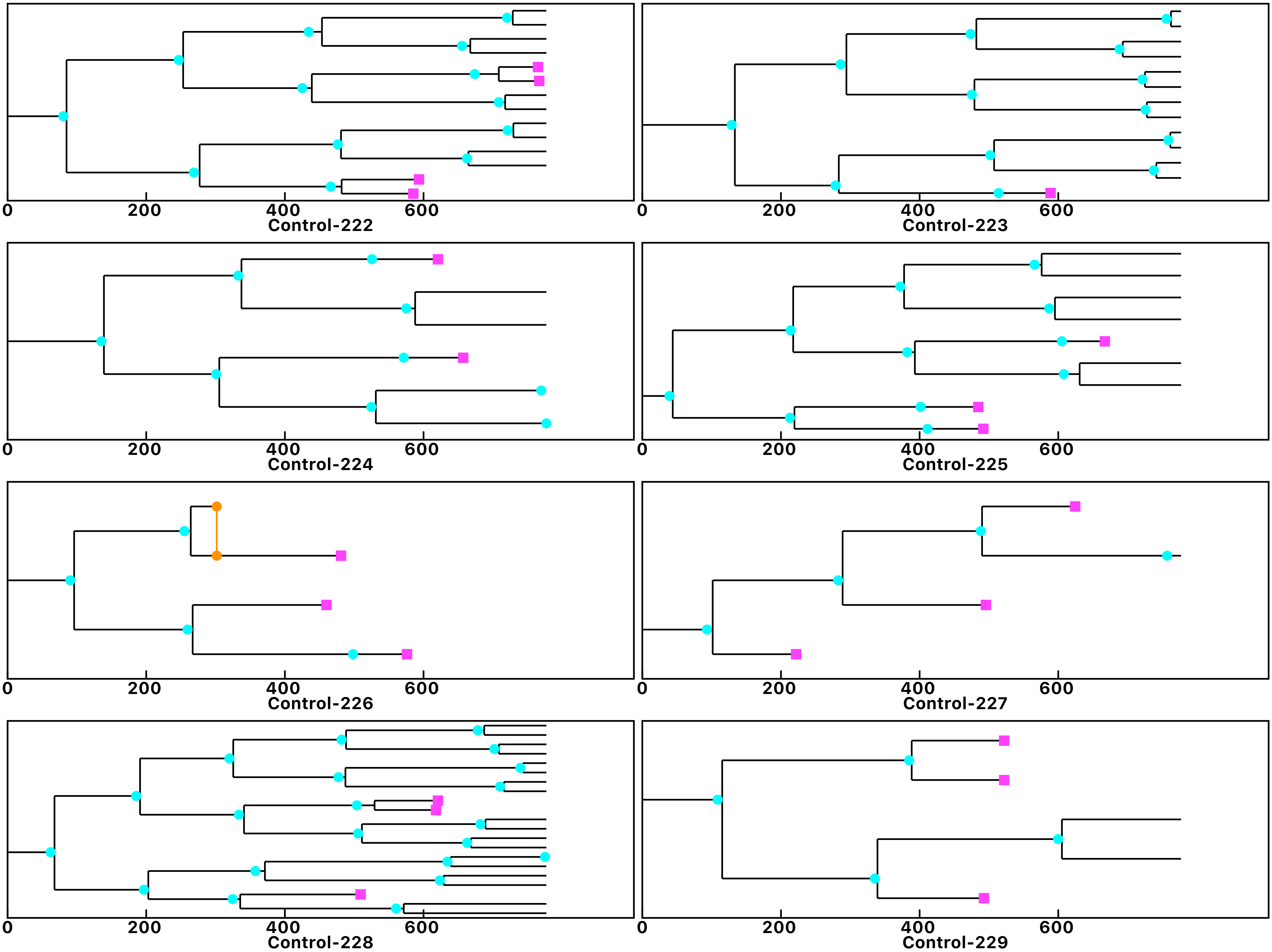

Analysis: MNNG, Treat.: Control, Cell: HeLa

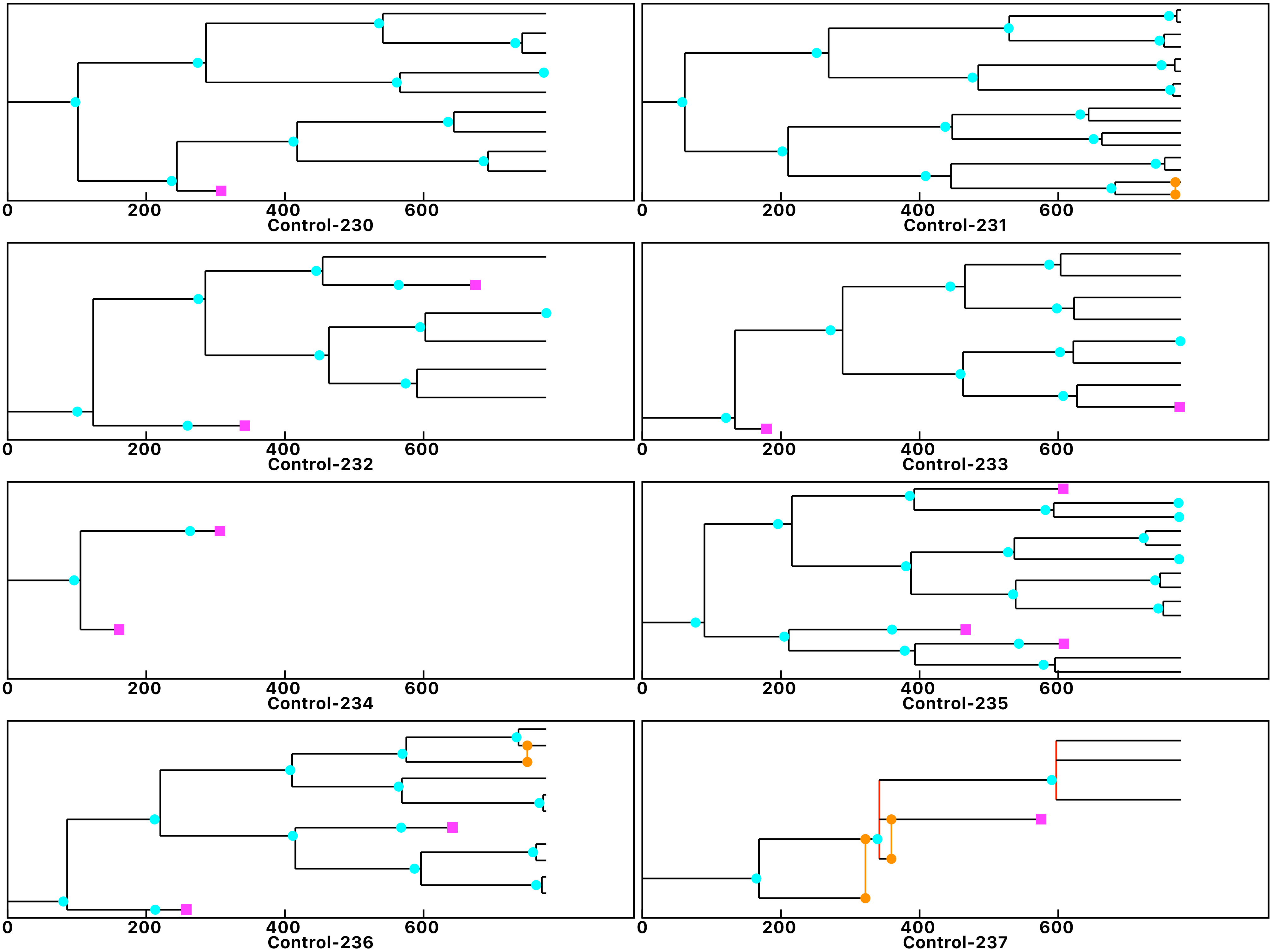

Analysis: MNNG, Treat.: Control, Cell: HeLa

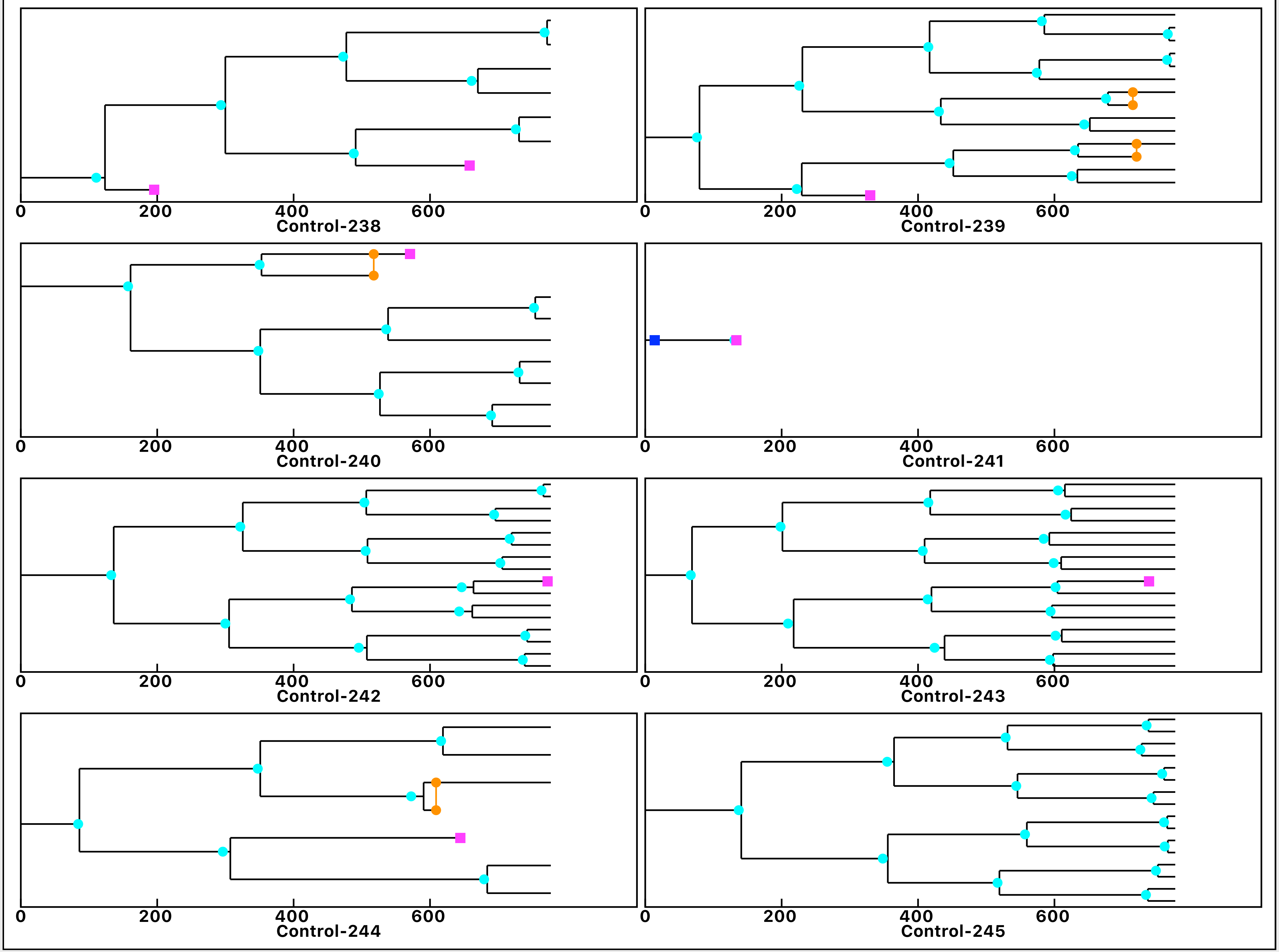

Analysis: MNNG, Treat.: Control, Cell: HeLa

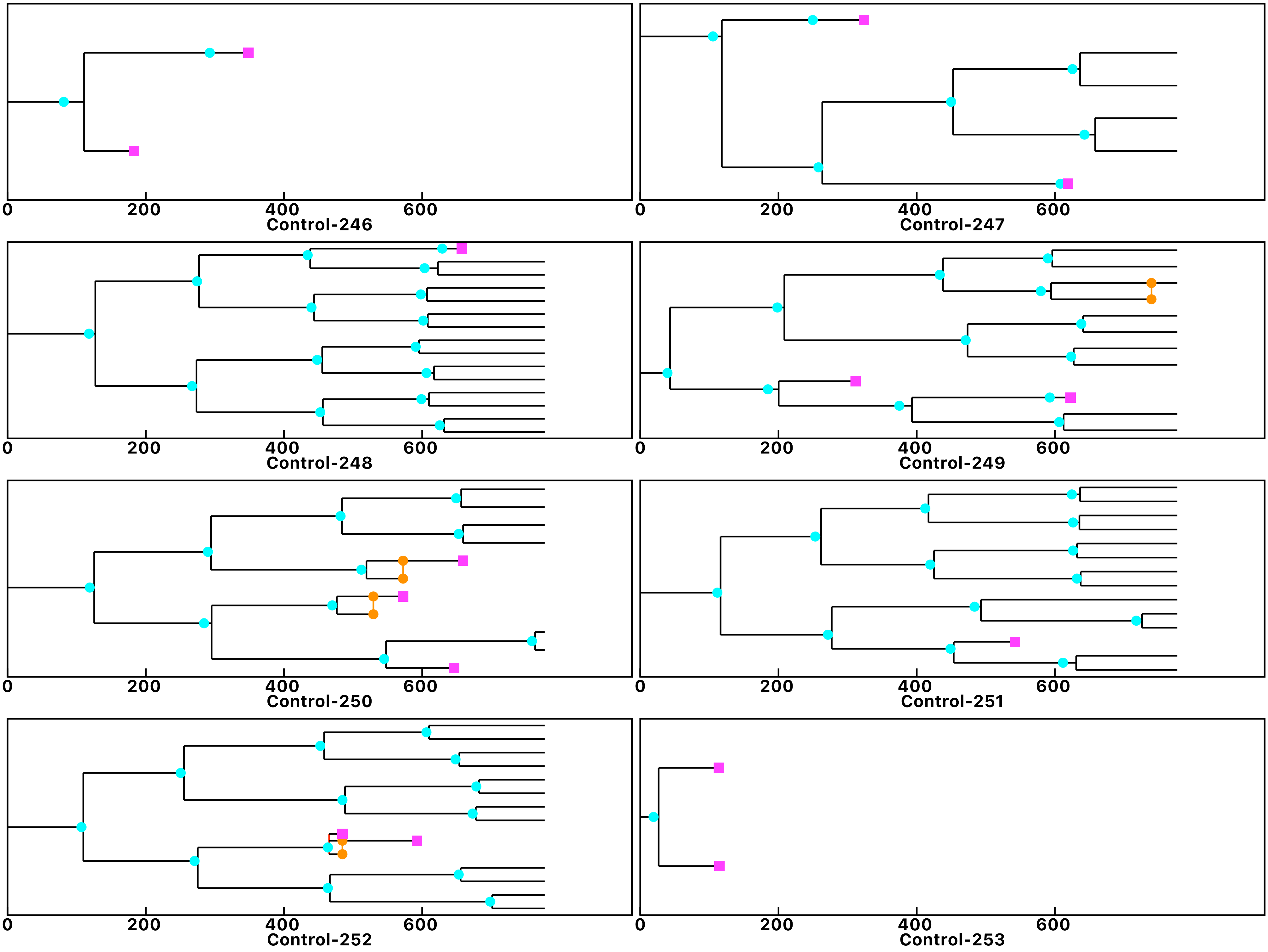

Analysis: MNNG, Treat.: Control, Cell: HeLa

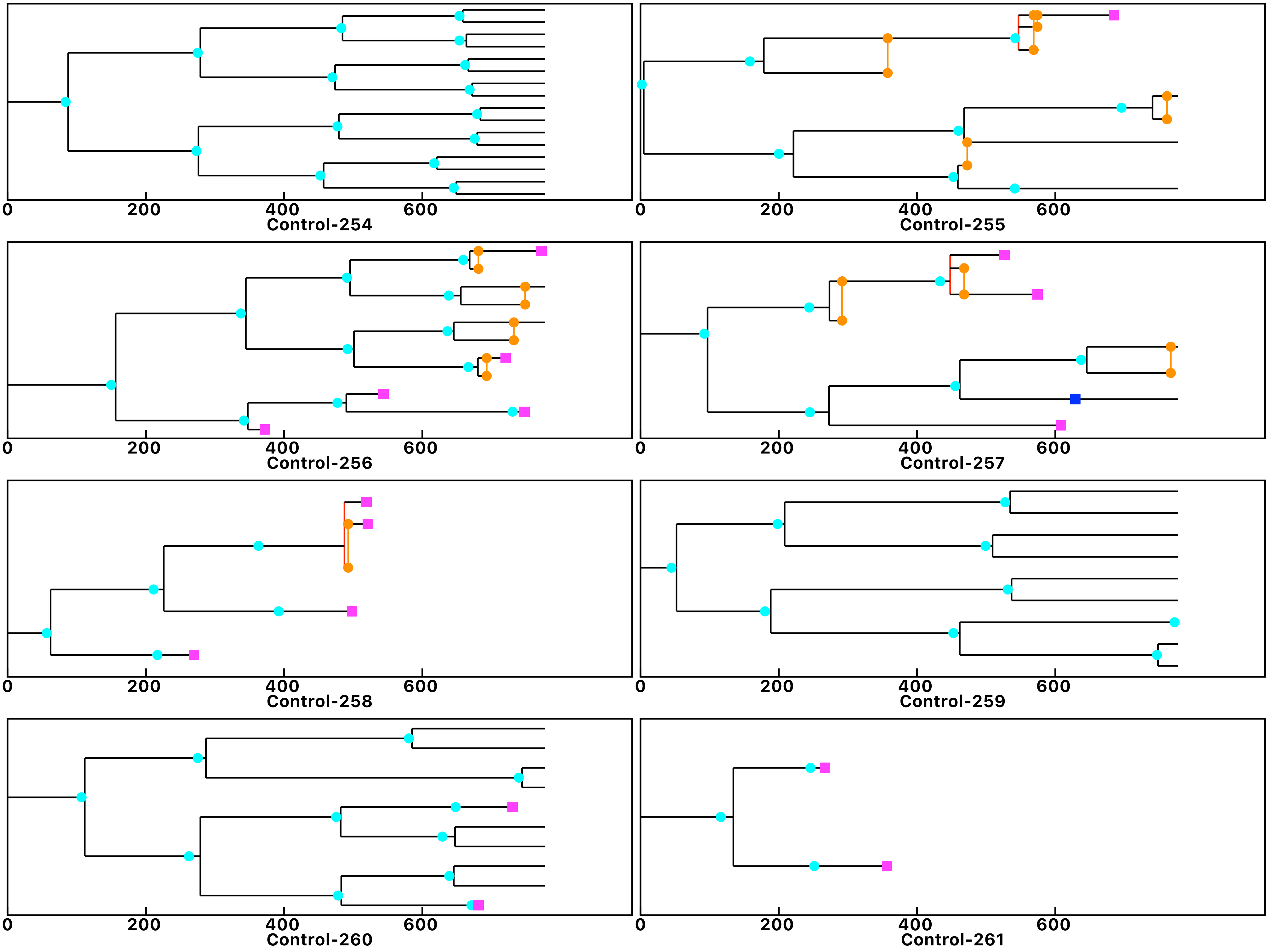

Analysis: MNNG, Treat.: Control, Cell: HeLa

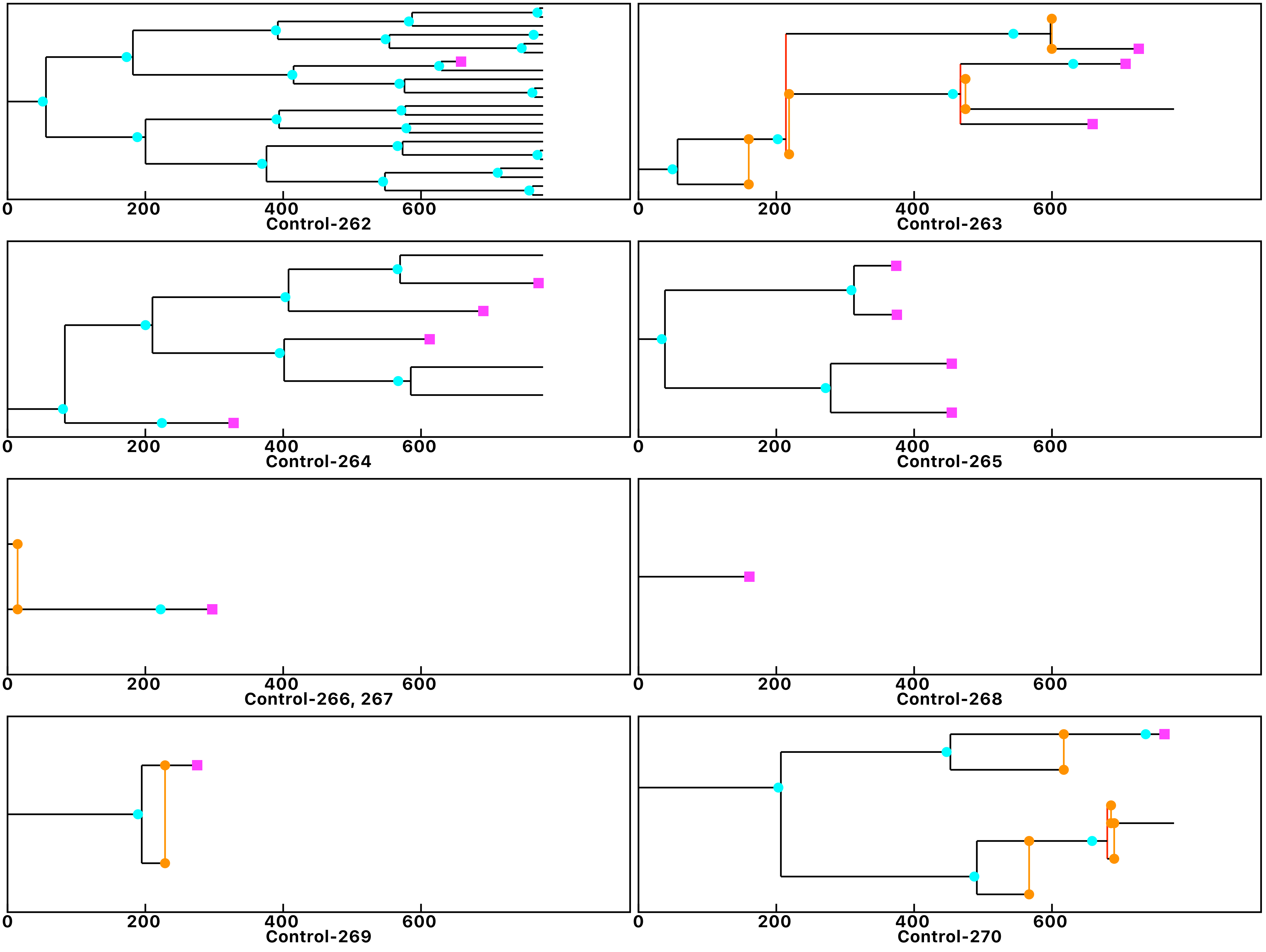

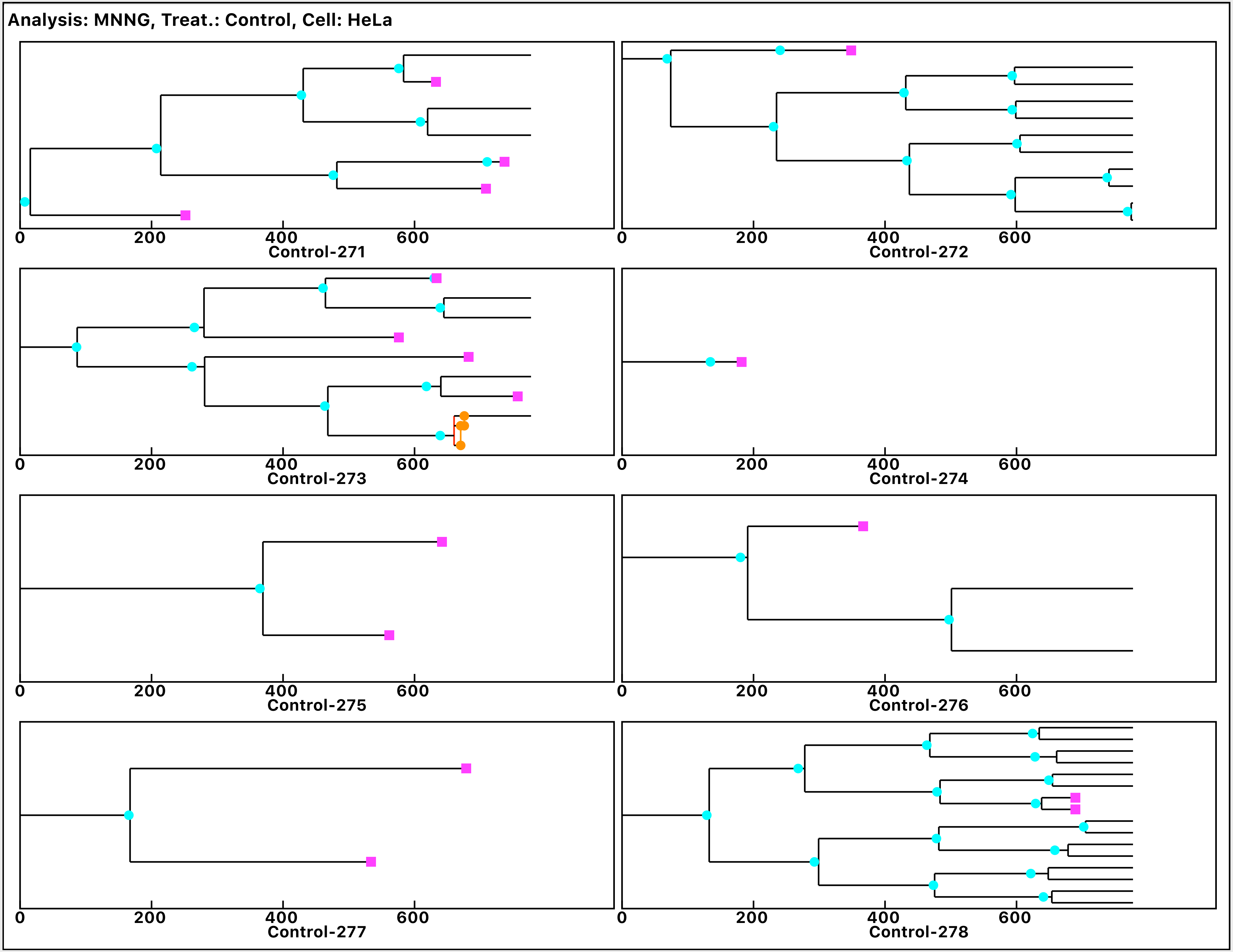
Analysis: MNNG, Treat.: Control, Cell: HeLa
0
200
400
600
Control-271
Control-272
Control-273
Control-274
Control-275
Control-276
Control-277
Control-278

Analysis: MNNG, Treat.: Control, Cell: HeLa

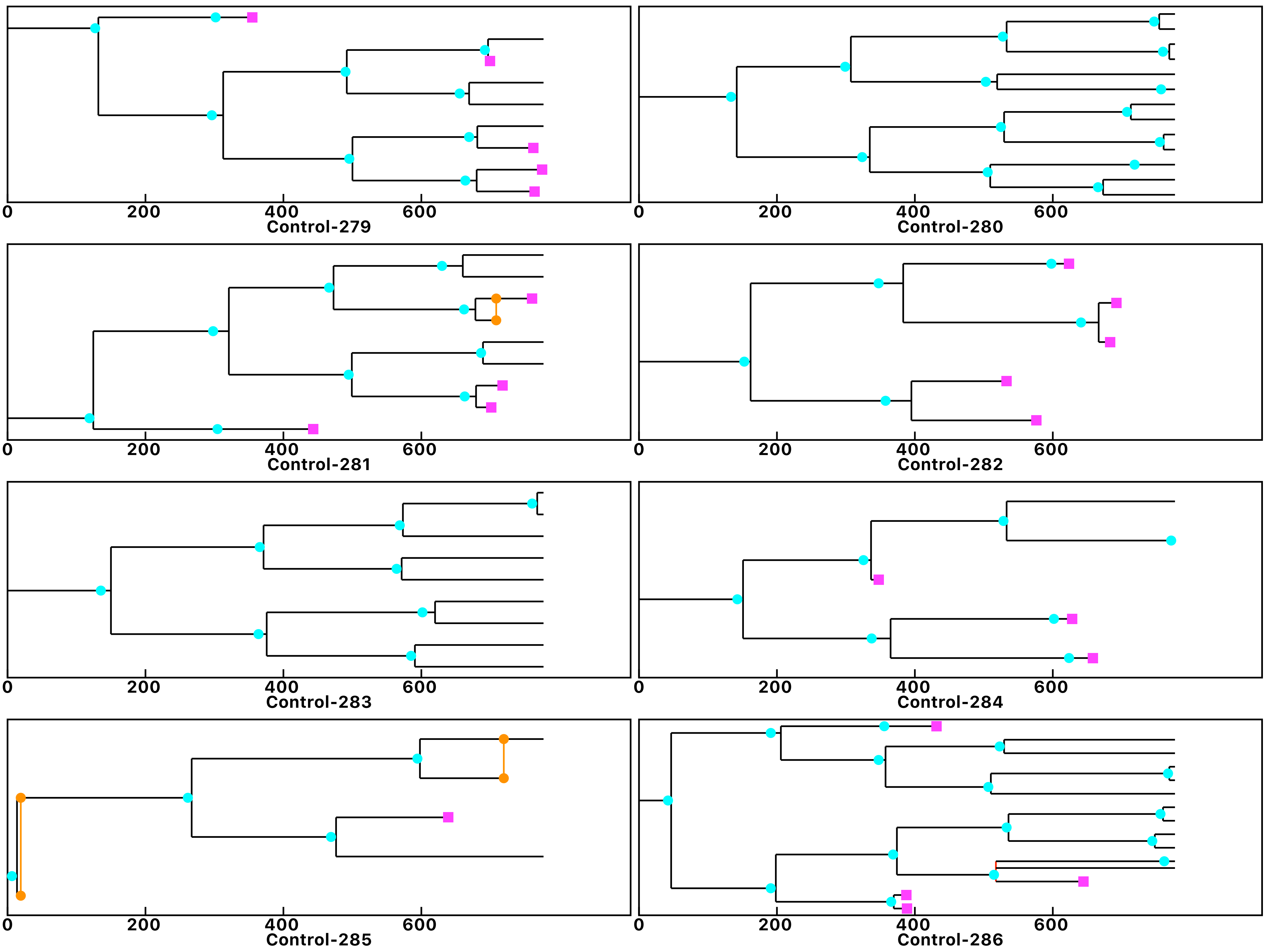

Analysis: MNNG, Treat.: Control, Cell: HeLa

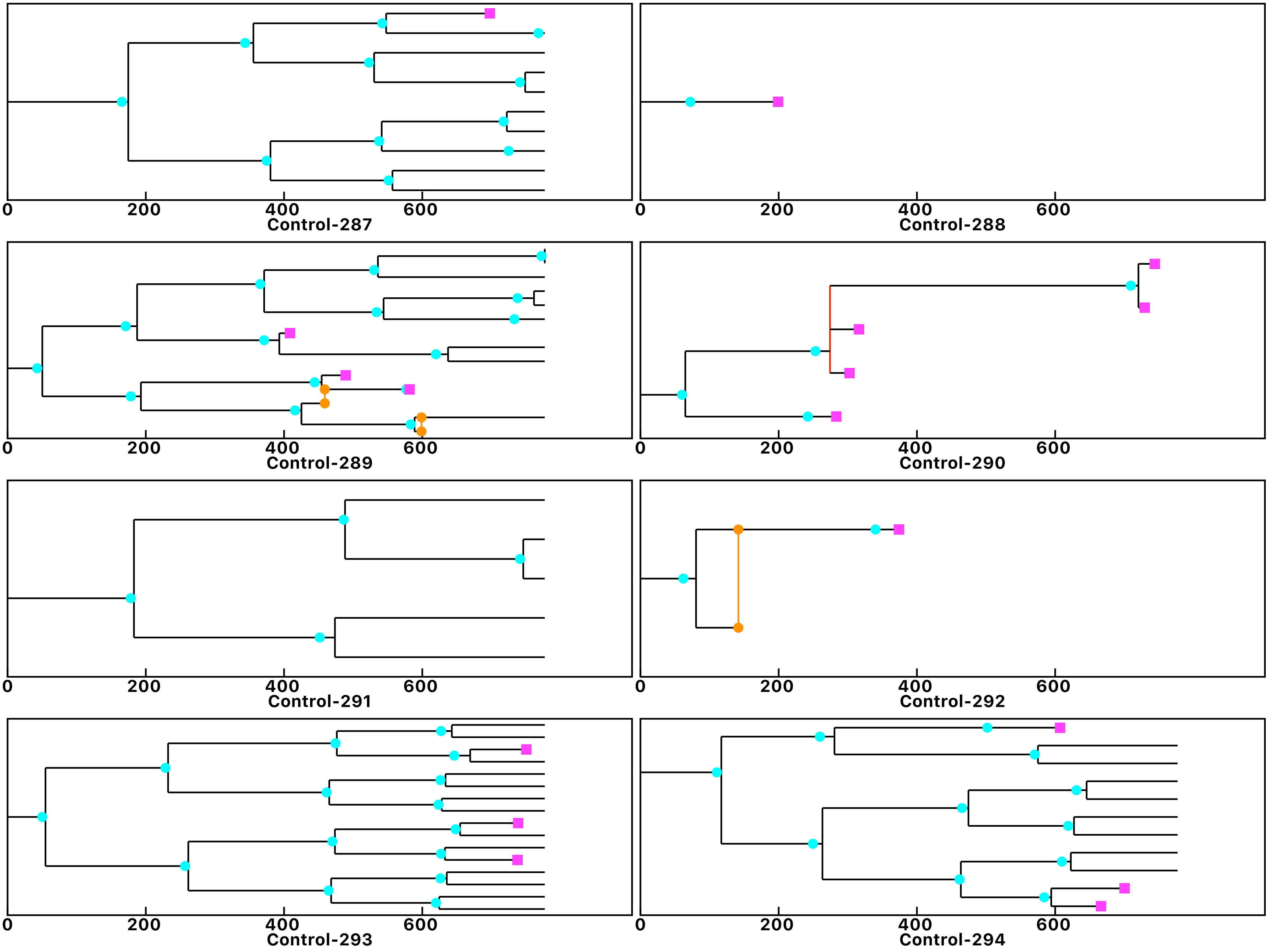

Analysis: MNNG, Treat.: Control, Cell: HeLa

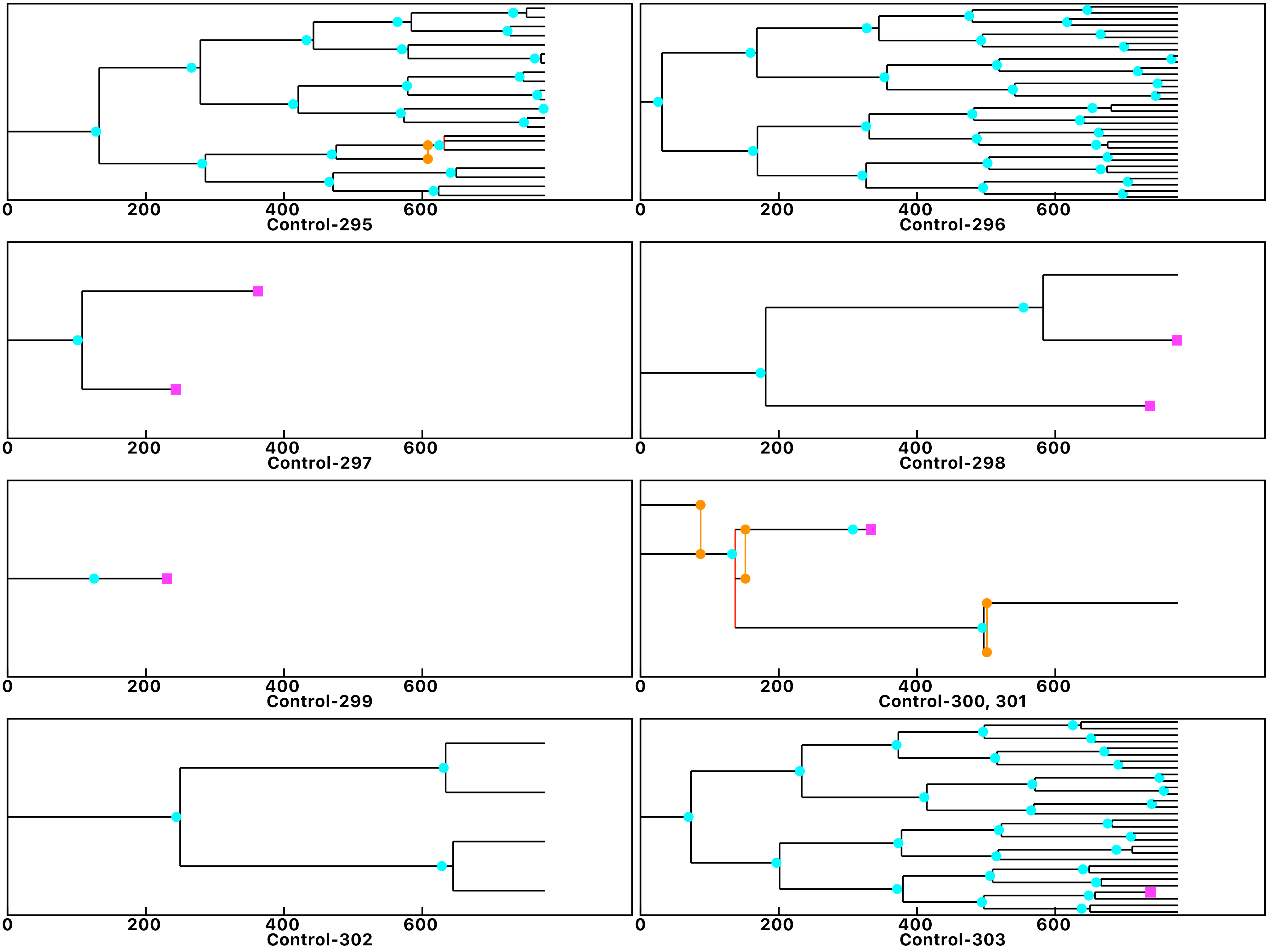

Analysis: MNNG, Treat.: Control, Cell: HeLa

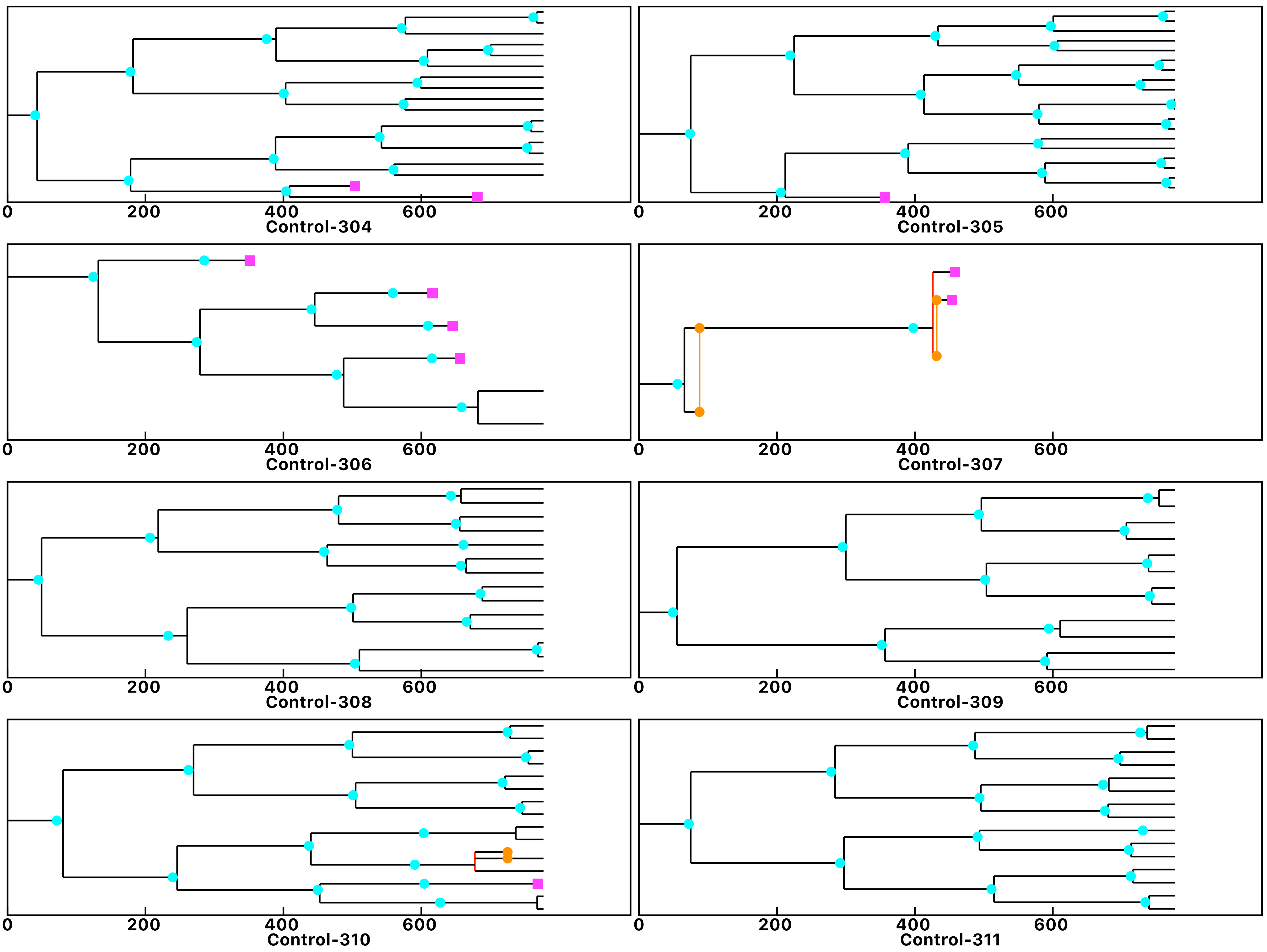

Analysis: MNNG, Treat.: Control, Cell: HeLa

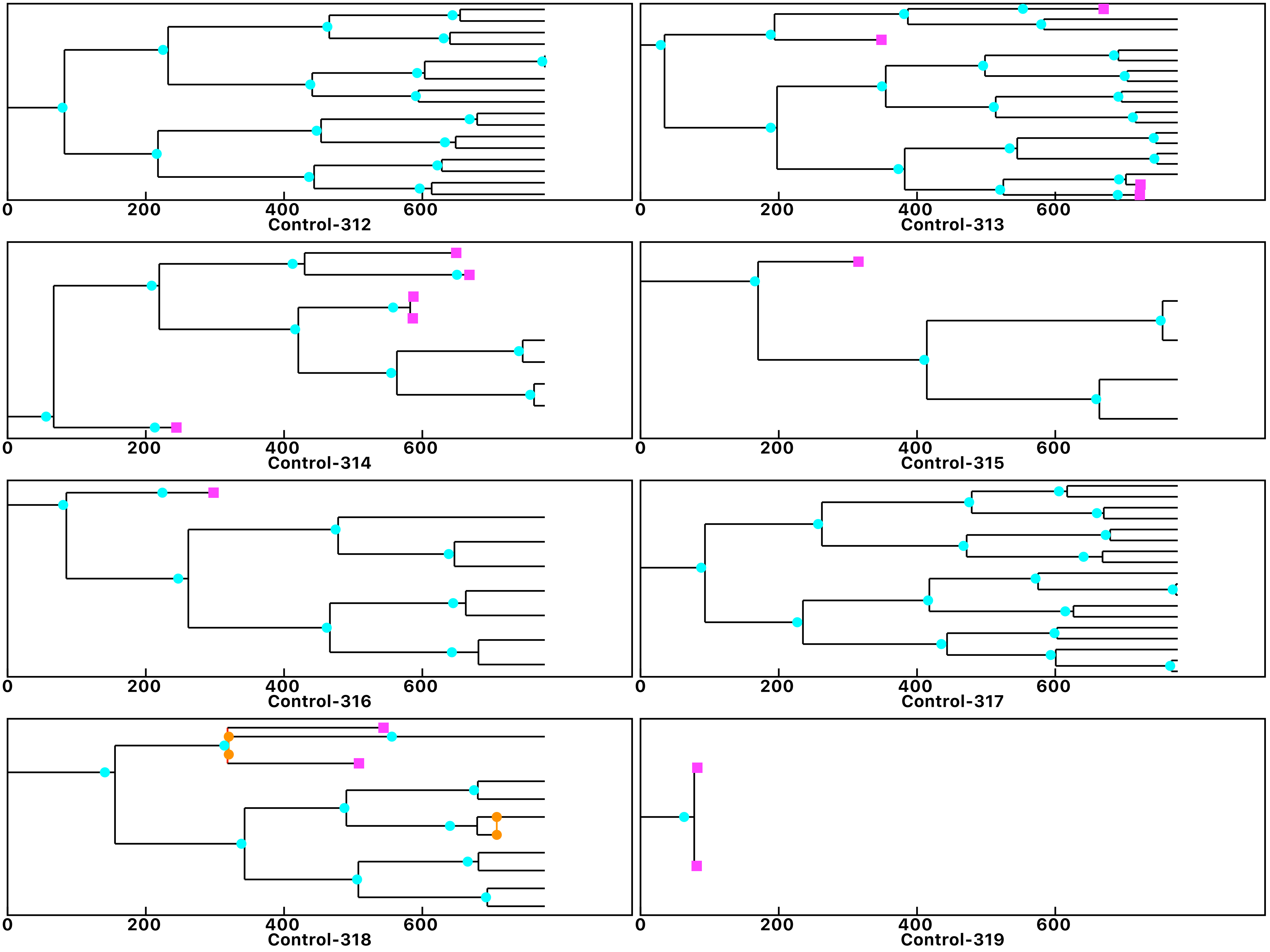

Analysis: MNNG, Treat.: Control, Cell: HeLa

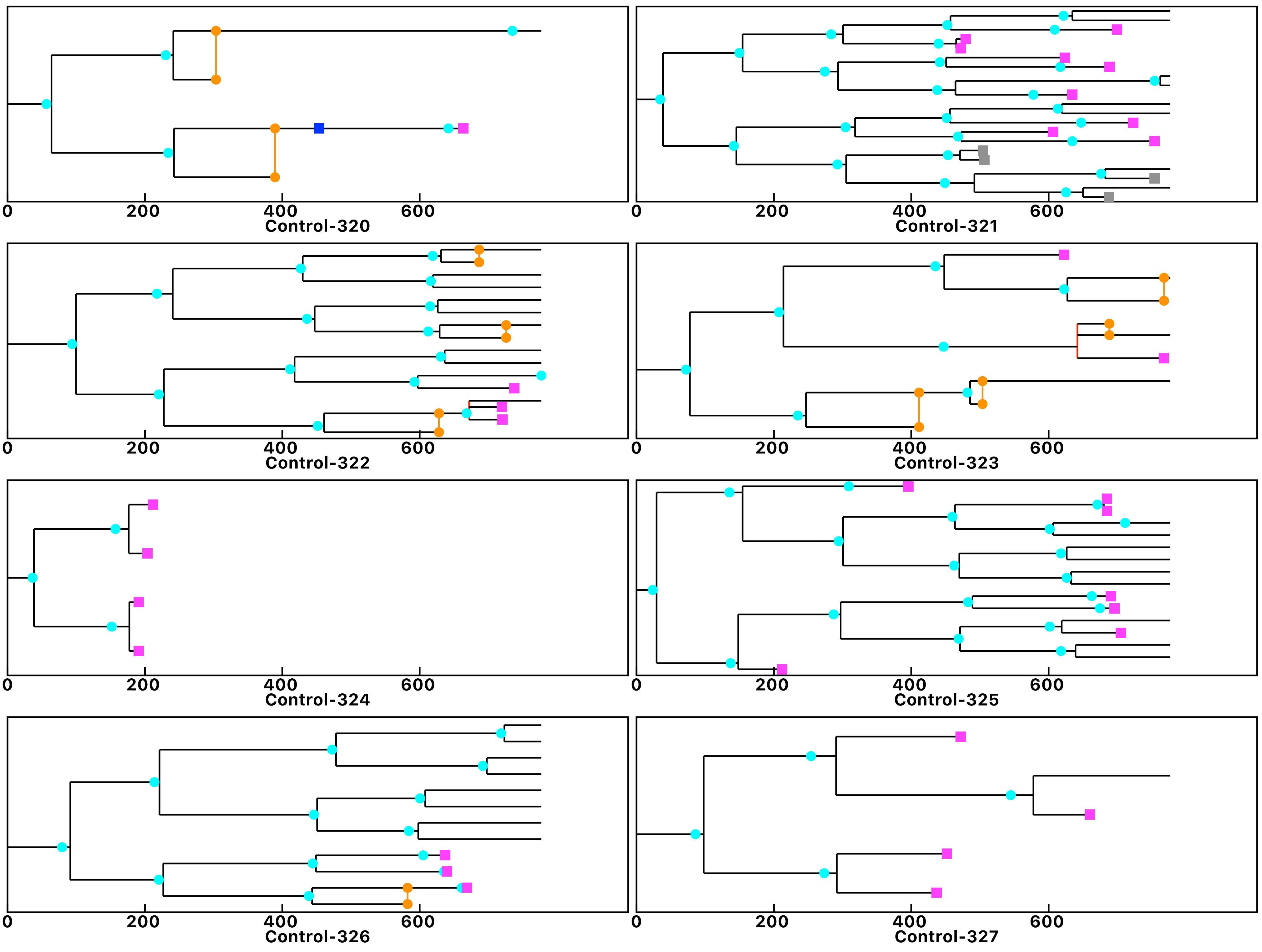

Analysis: MNNG, Treat.: Control, Cell: HeLa

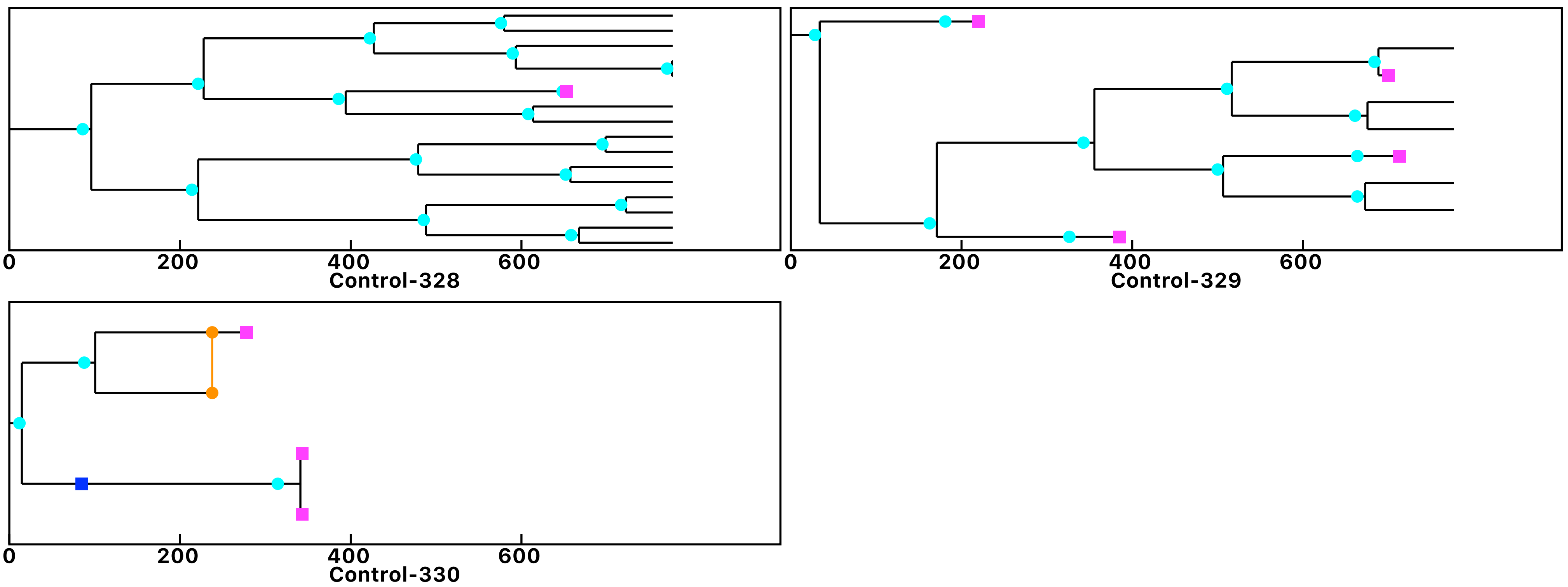

Supplement: S1 Fig — Cell lineage maps (Control) are shown. Light blue circle: mitosis, orange circle: cell fusion, pink square: cell death, blue square: incomplete cell division, black vertical line: bipolar cell division, red vertical line: multipolar cell division, and orange vertical line: cell fusion. (PDF) [file pone.0214512.s001.pdf]
