# Supplementary material for: Dose-dependent spatiotemporal responses of mammalian cells to an alkylating agent
Source: PLoS One. 2019 Mar 29;14(3):e0214512. doi: 10.1371/journal.pone.0214512 (PMC6440626; doi:10.1371/journal.pone.0214512)

Supplementary Figure S2

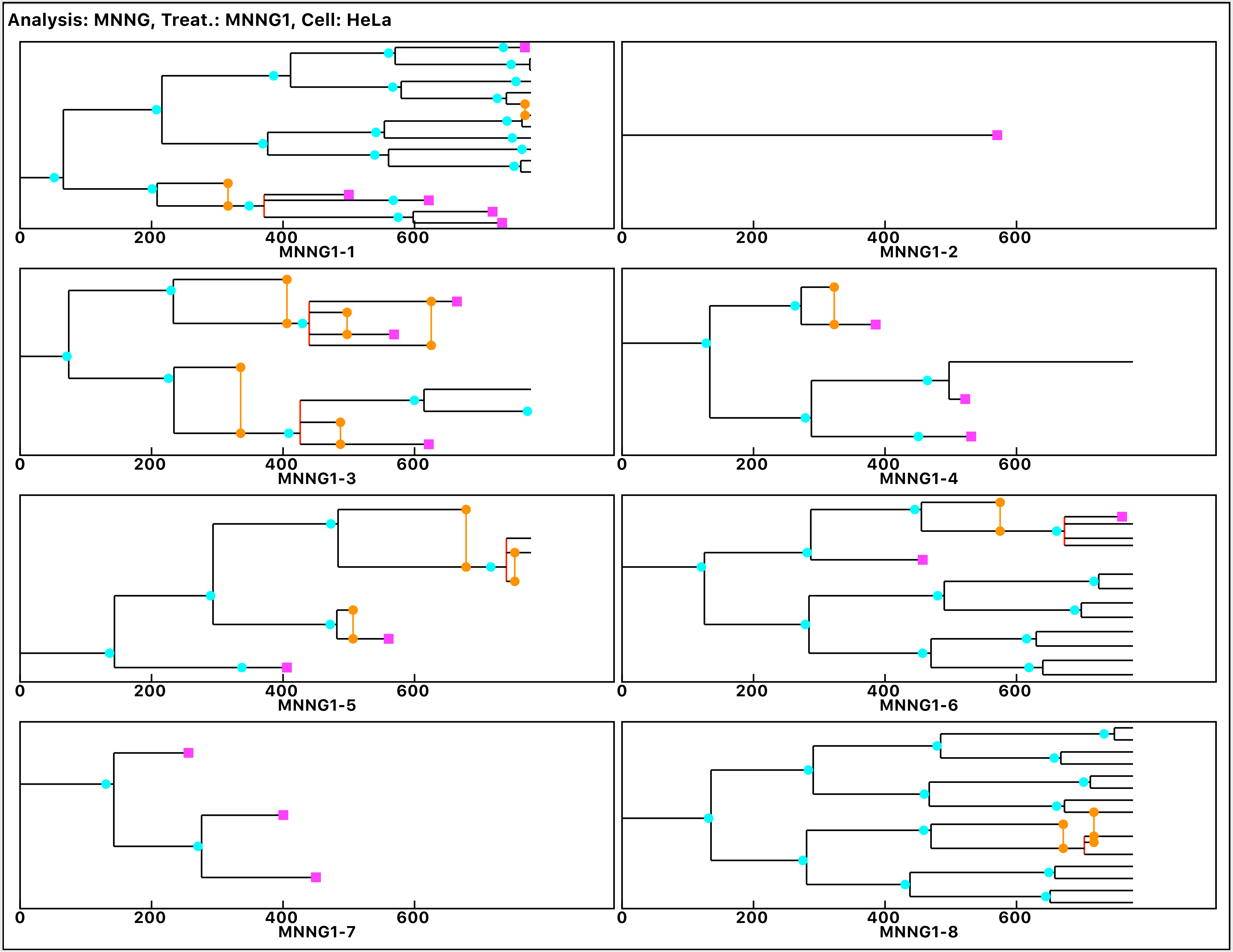
Analysis: MNNG, Treat.: MNNG1, Cell: HeLa
0
200
400
600
MNNG1-1
MNNG1-2
MNNG1-3
MNNG1-4
MNNG1-5
MNNG1-6
MNNG1-7
MNNG1-8

Analysis: MNNG, Treat.: MNNG1, Cell: HeLa

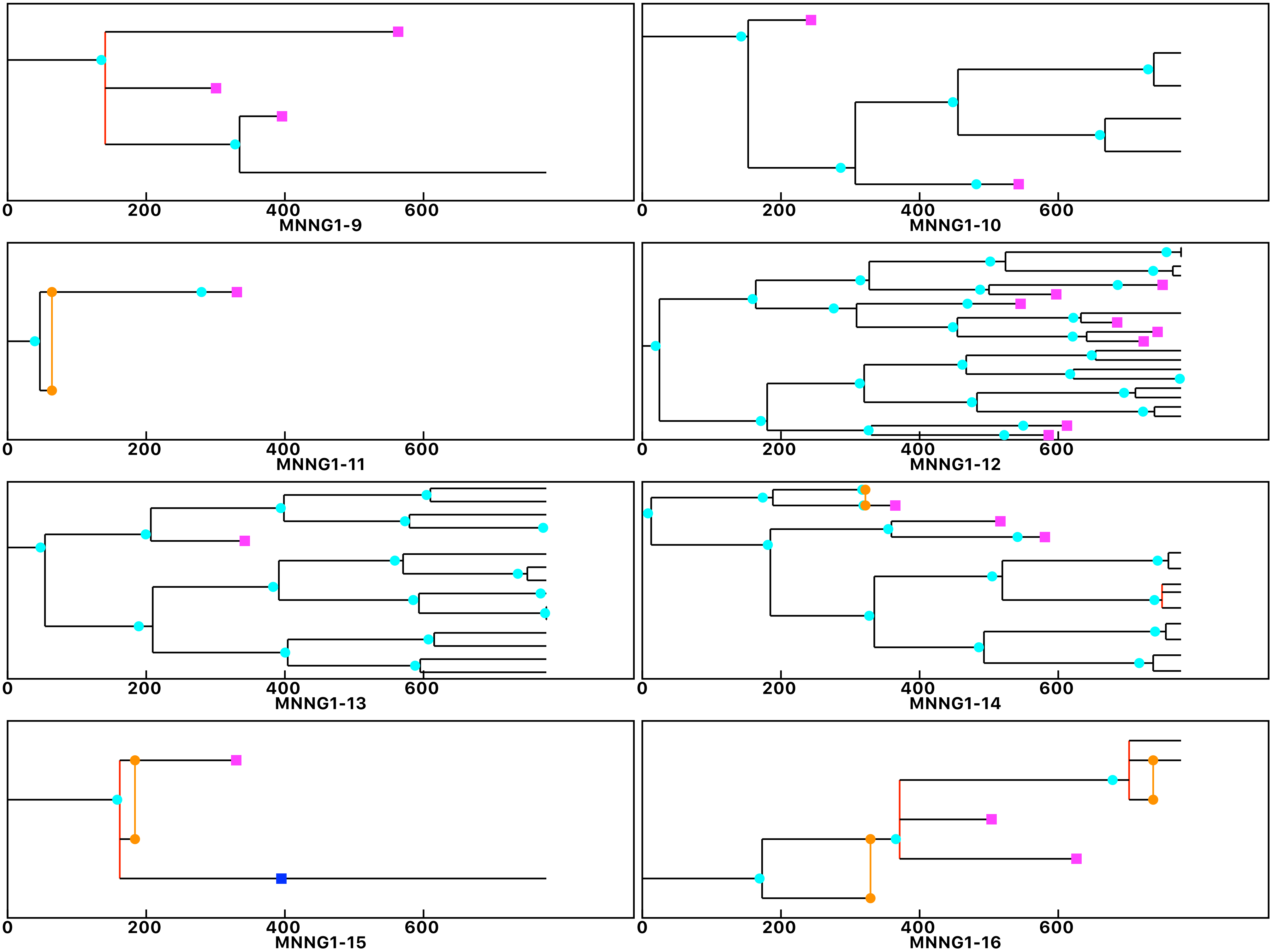

Analysis: MNNG, Treat.: MNNG1, Cell: HeLa

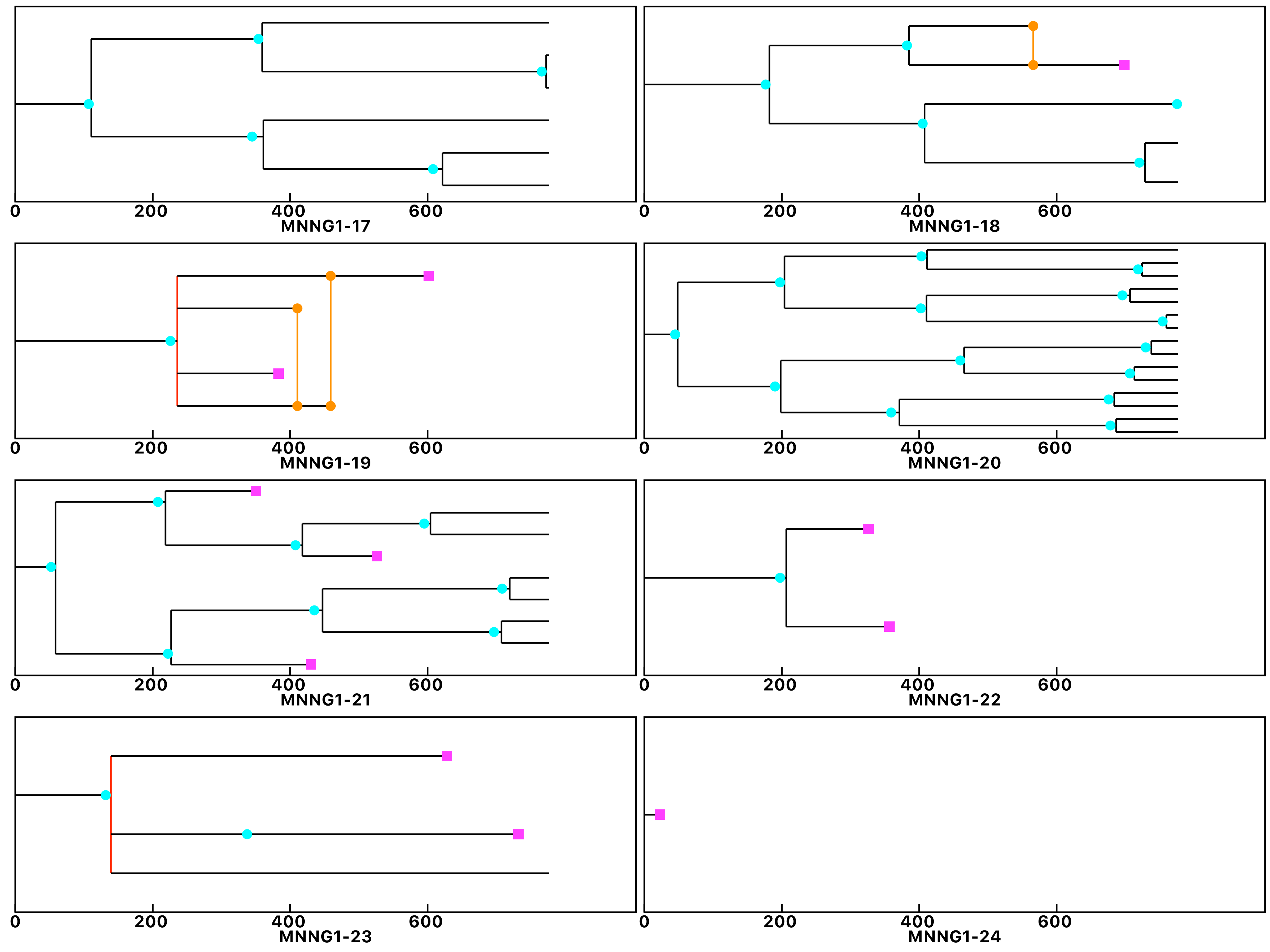

Analysis: MNNG, Treat.: MNNG1, Cell: HeLa

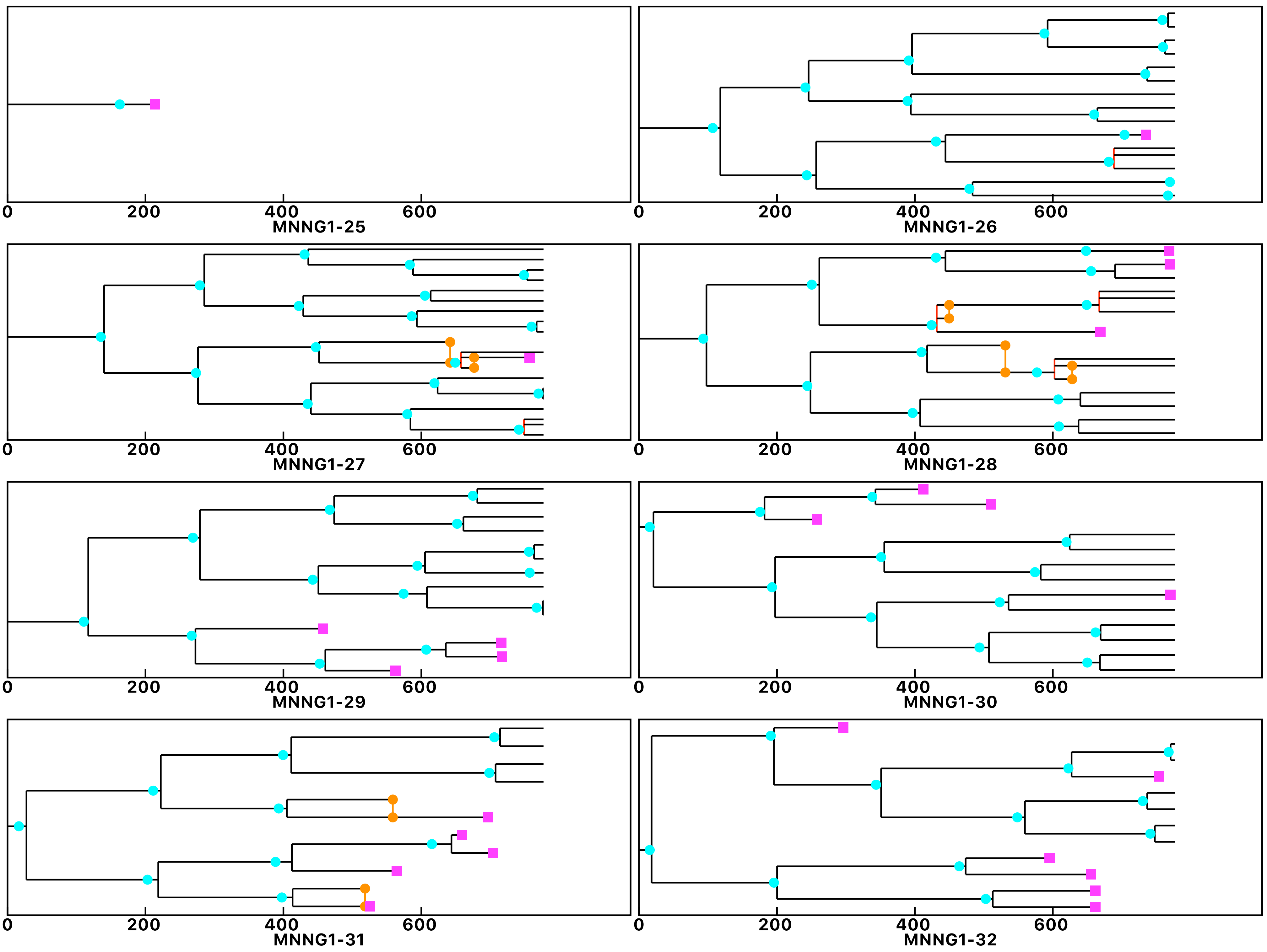

Analysis: MNNG, Treat.: MNNG1, Cell: HeLa

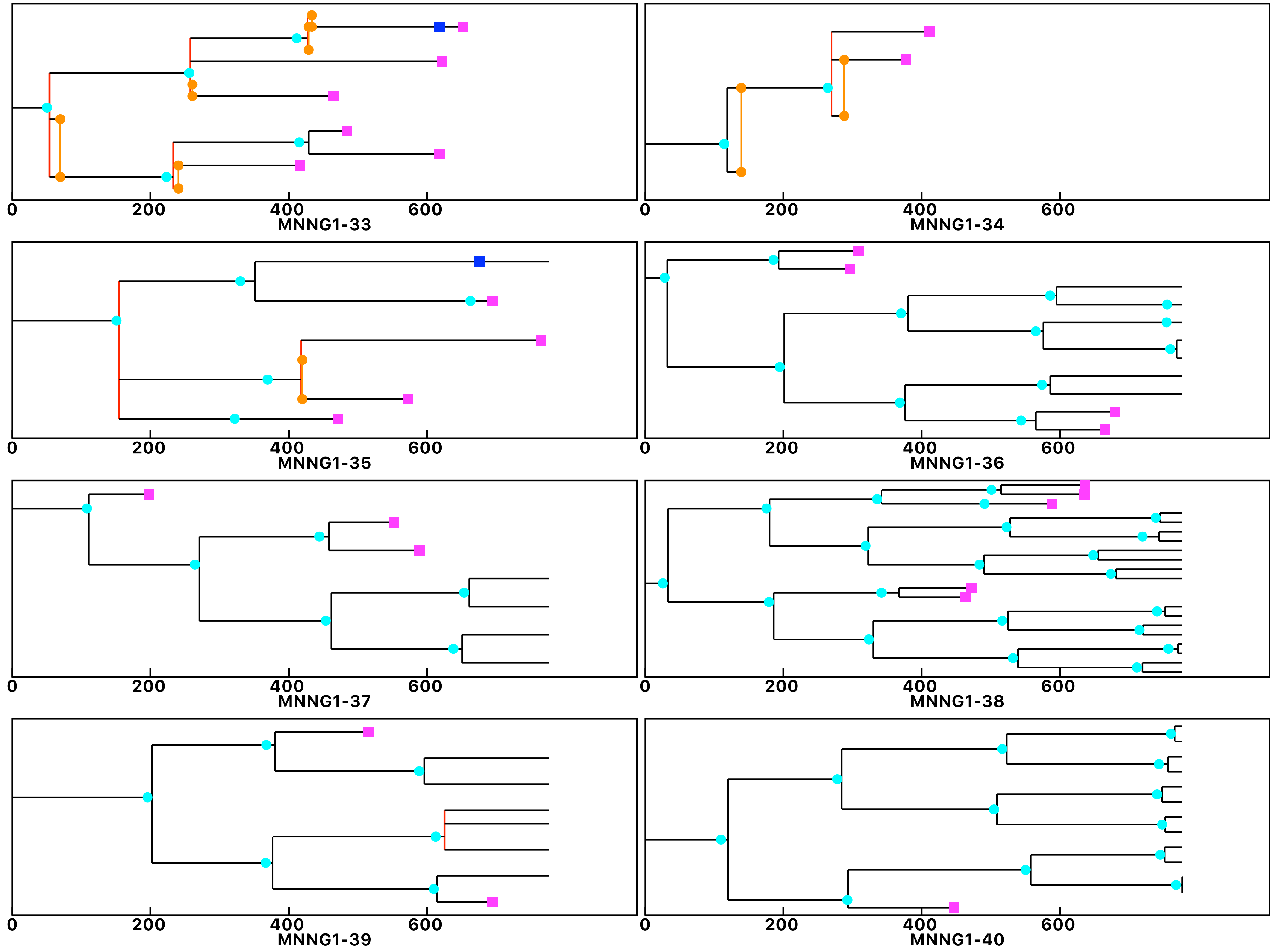

Analysis: MNNG, Treat.: MNNG1, Cell: HeLa

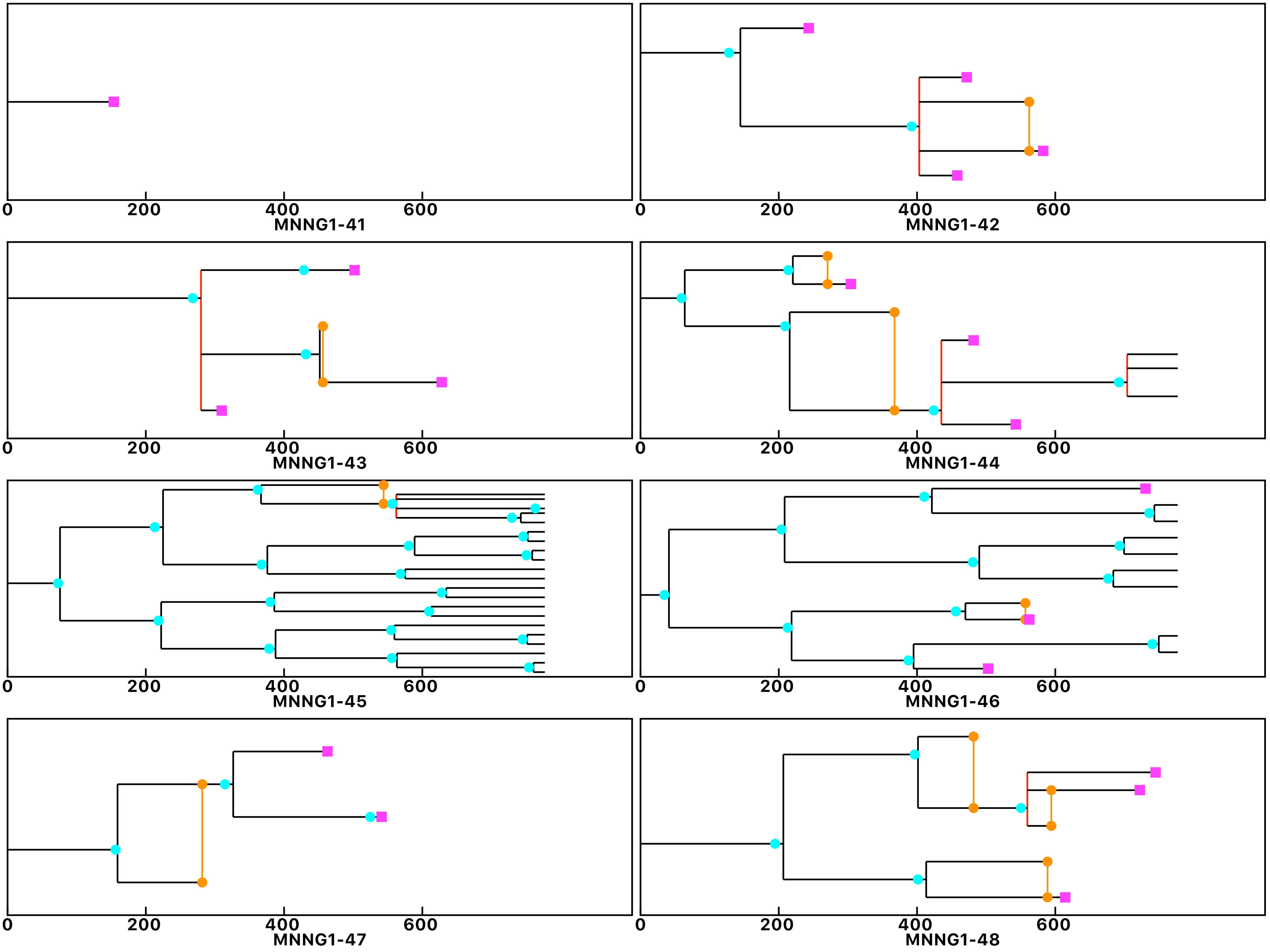

Analysis: MNNG, Treat.: MNNG1, Cell: HeLa

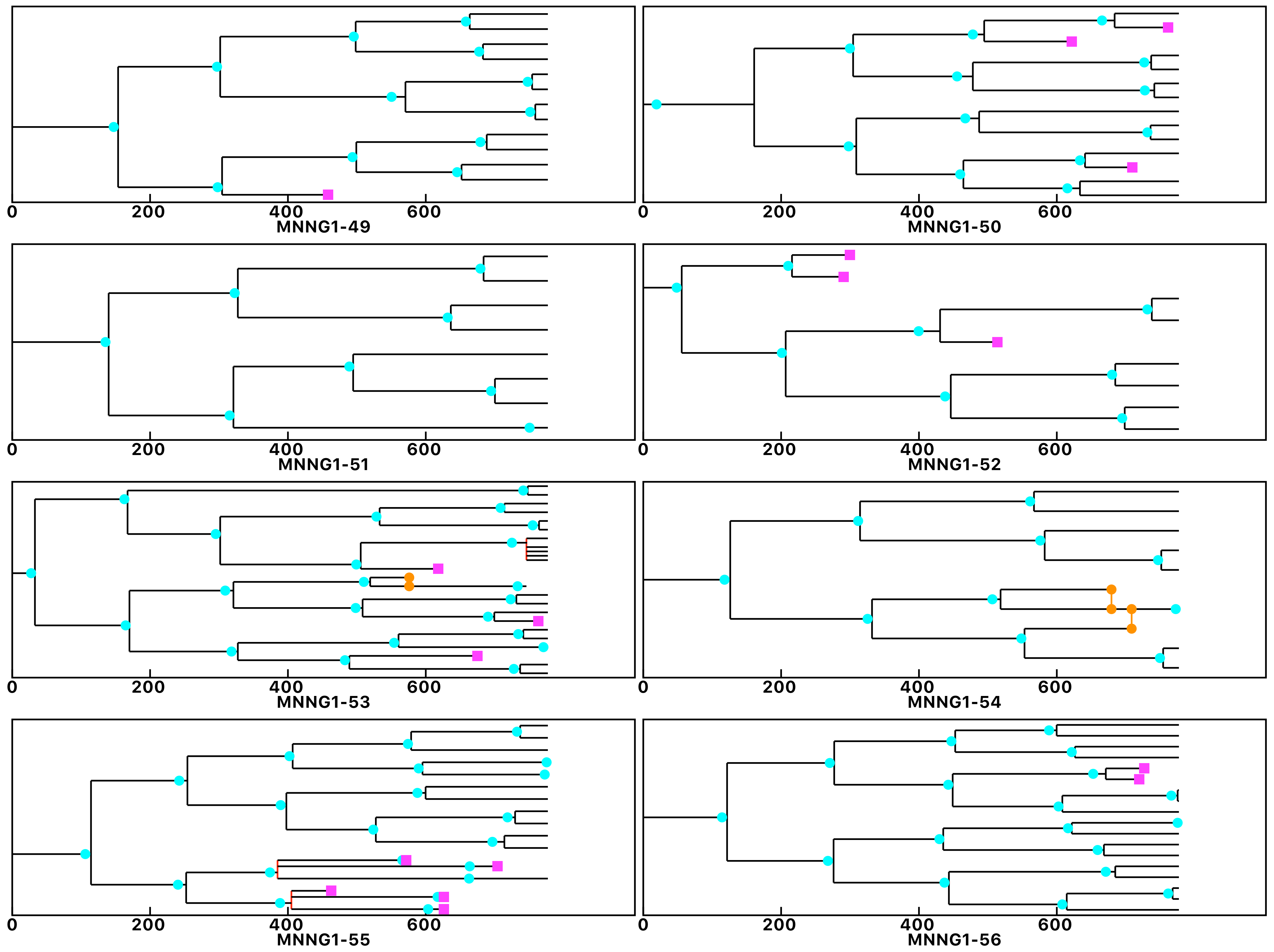

Analysis: MNNG, Treat.: MNNG1, Cell: HeLa

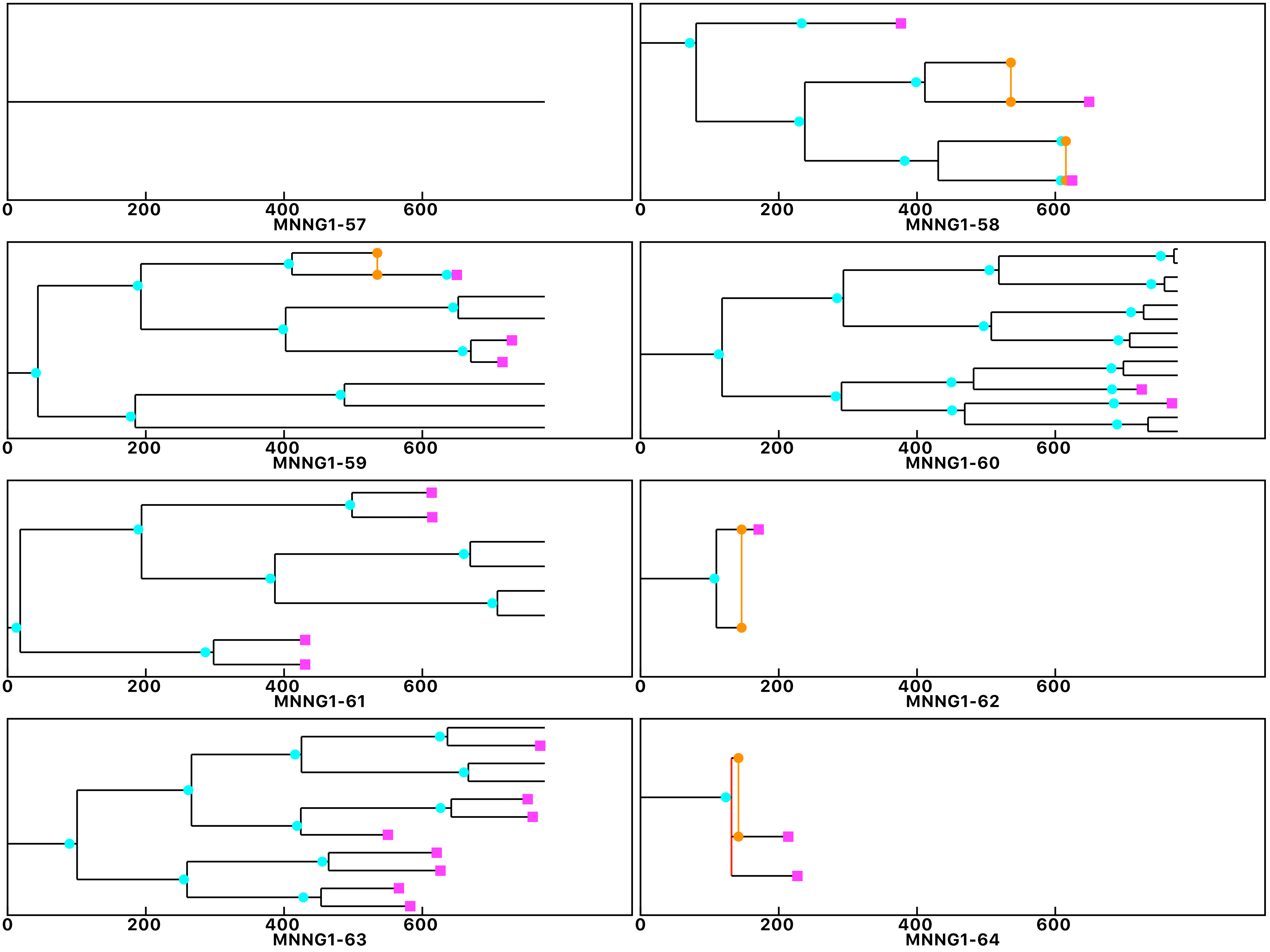

Analysis: MNNG, Treat.: MNNG1, Cell: HeLa

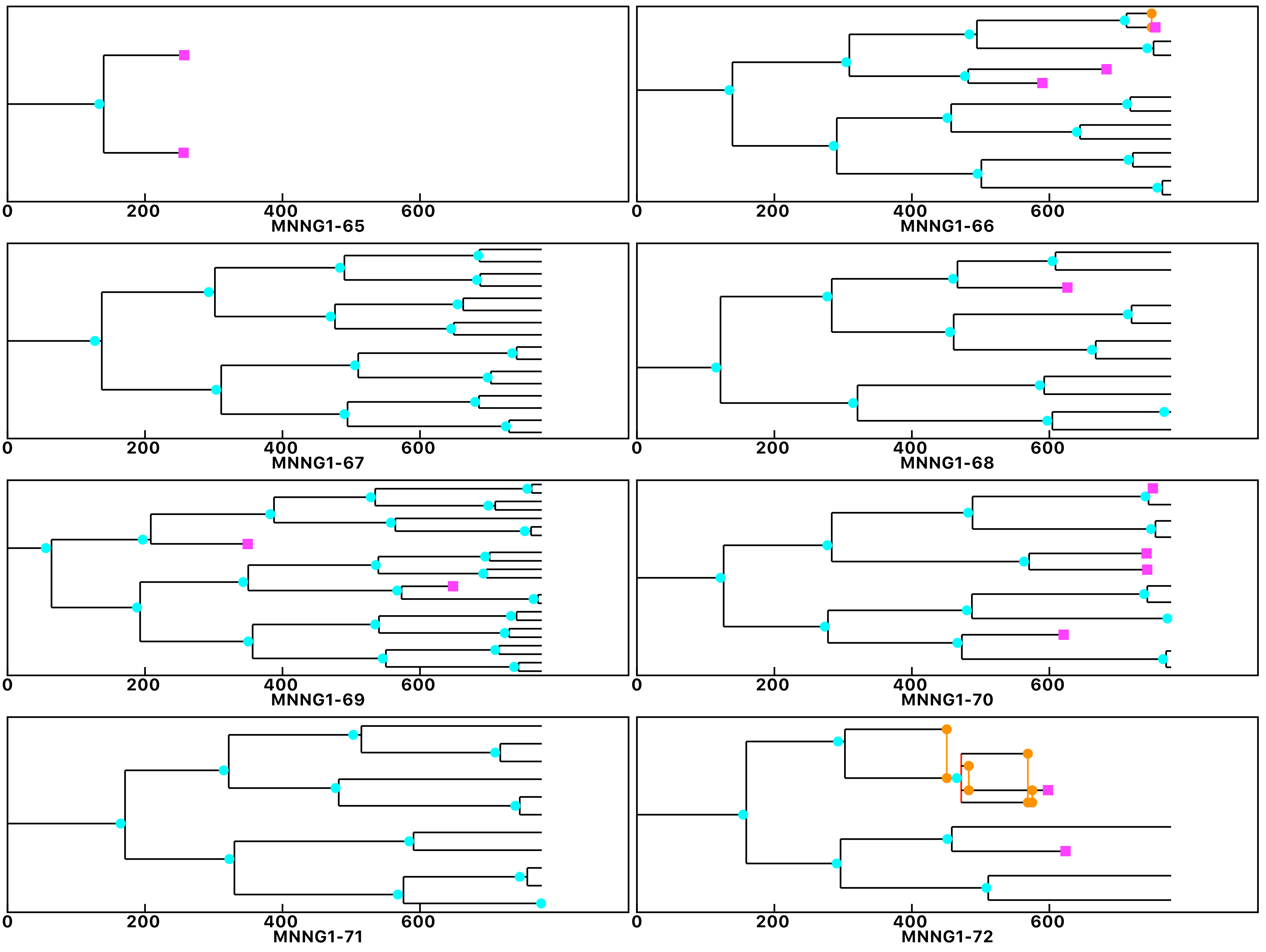

Analysis: MNNG, Treat.: MNNG1, Cell: HeLa

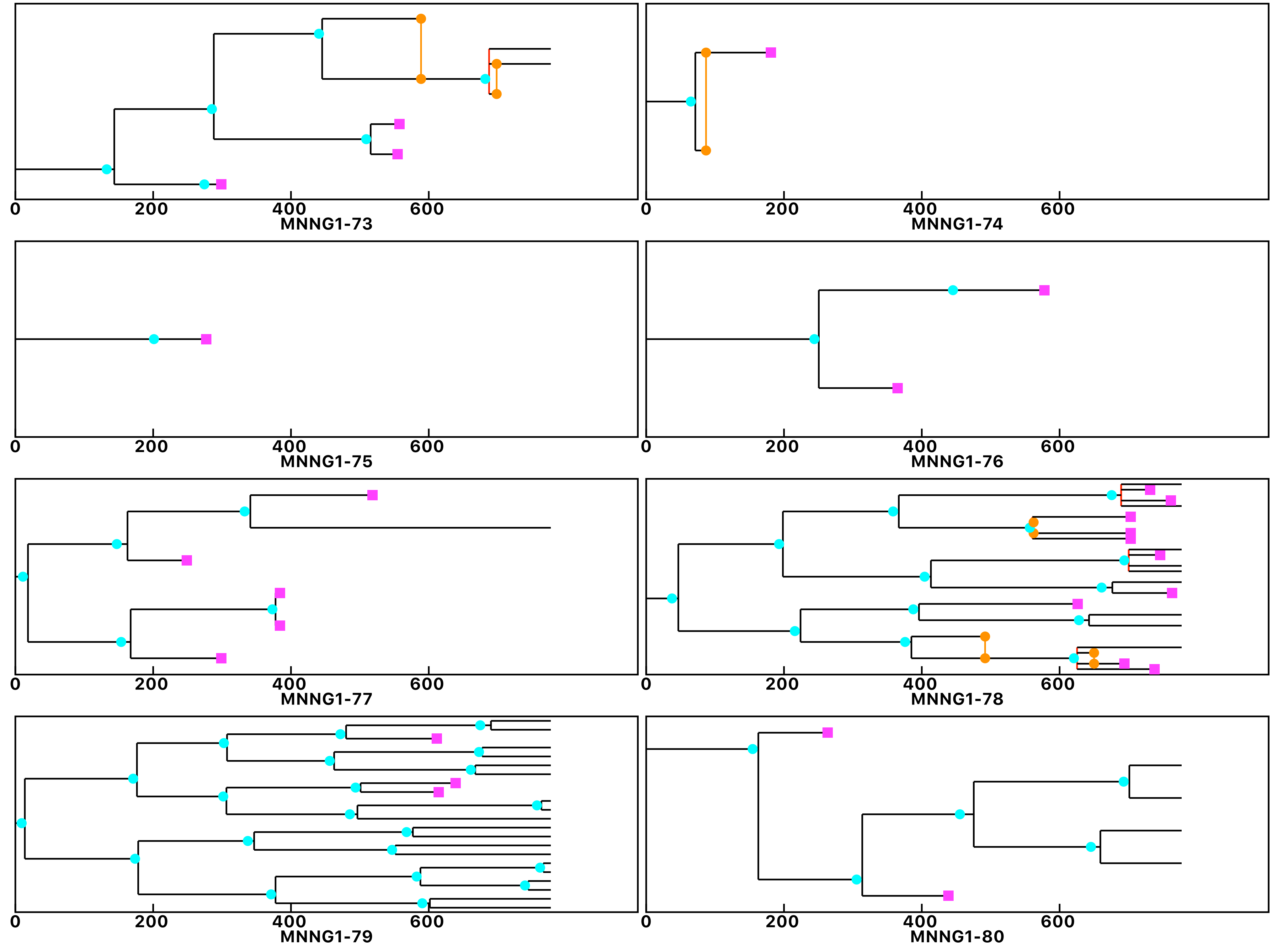

Analysis: MNNG, Treat.: MNNG1, Cell: HeLa

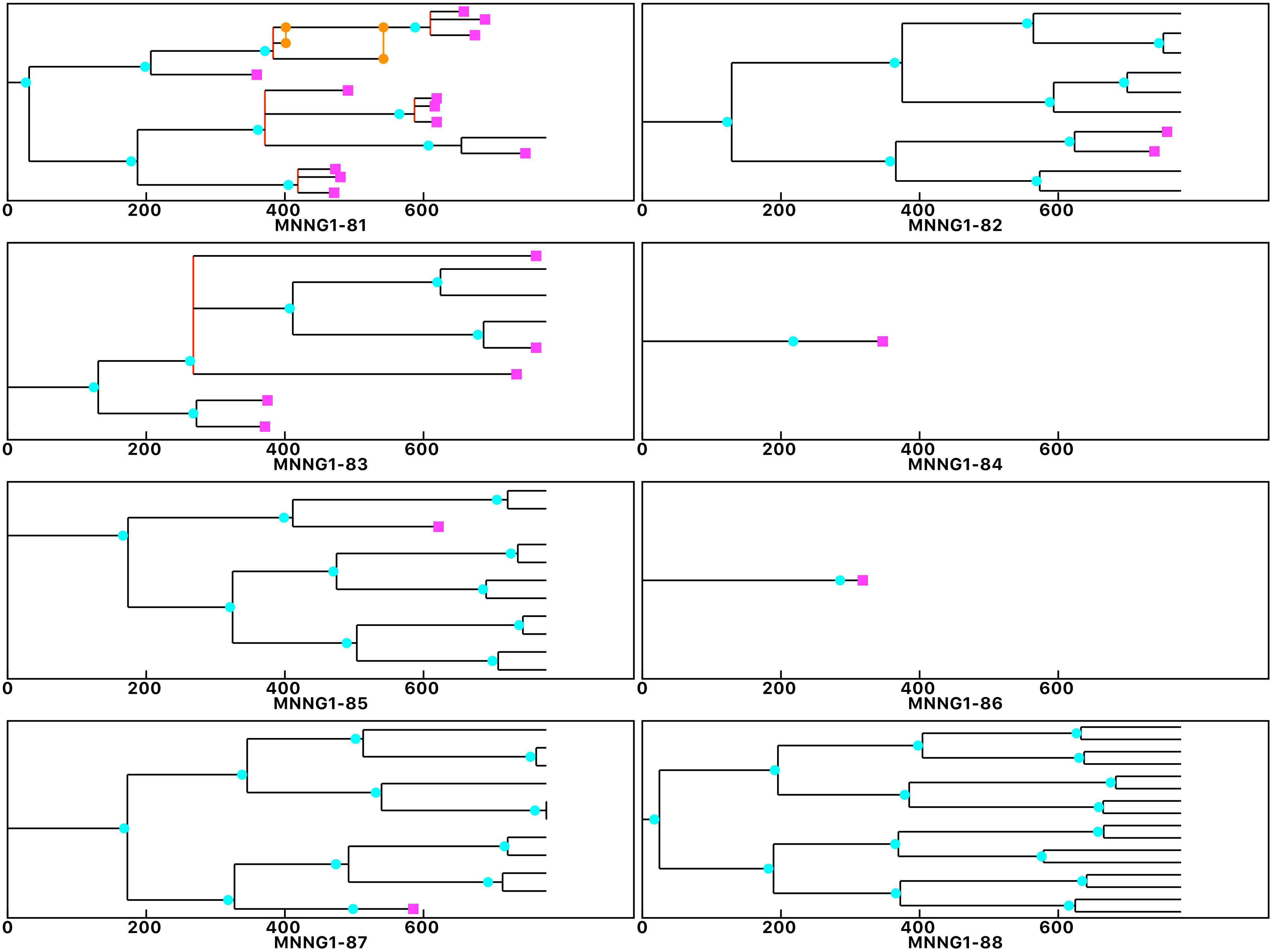

Analysis: MNNG, Treat.: MNNG1, Cell: HeLa

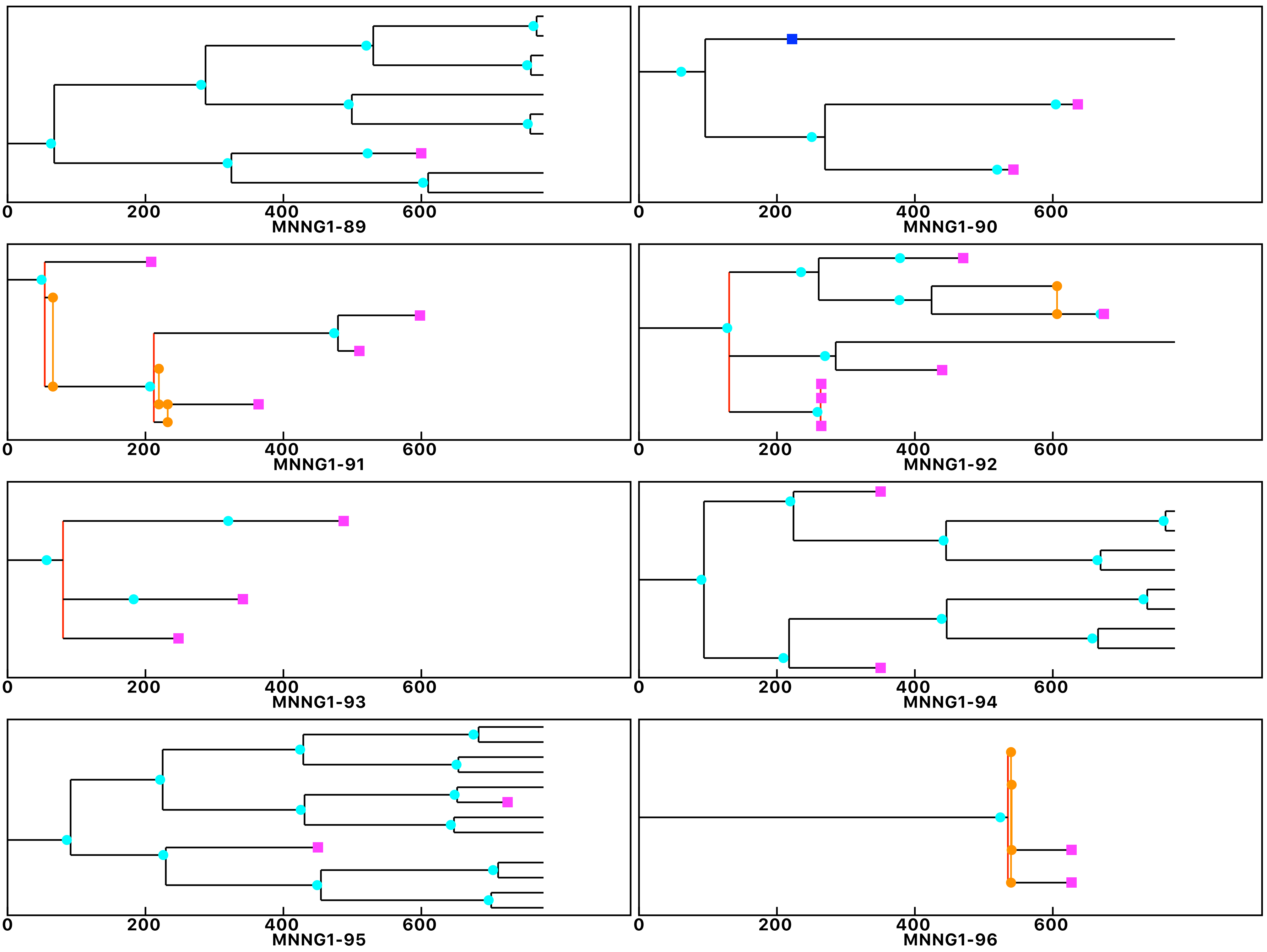

Analysis: MNNG, Treat.: MNNG1, Cell: HeLa

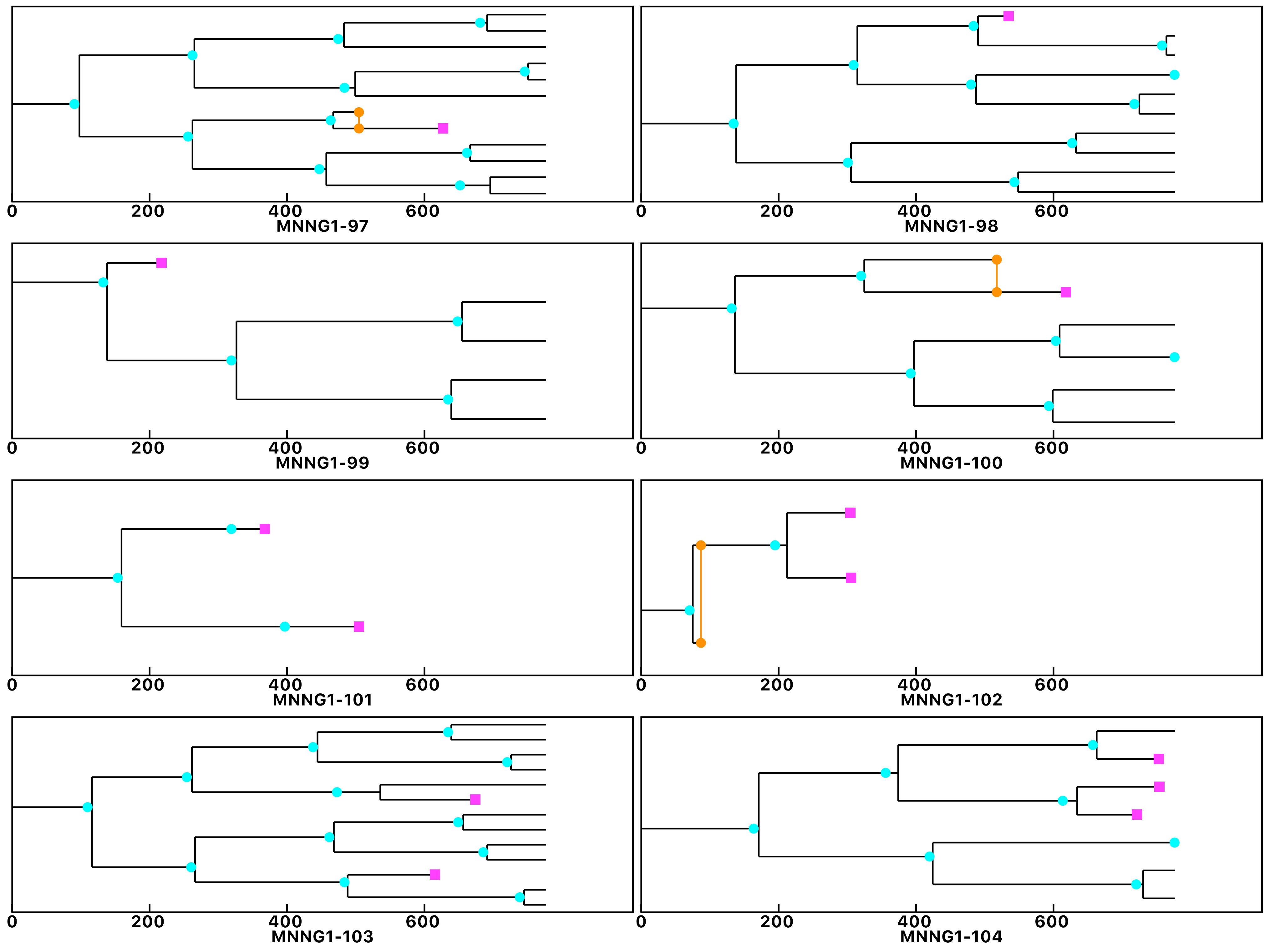

Analysis: MNNG, Treat.: MNNG1, Cell: HeLa

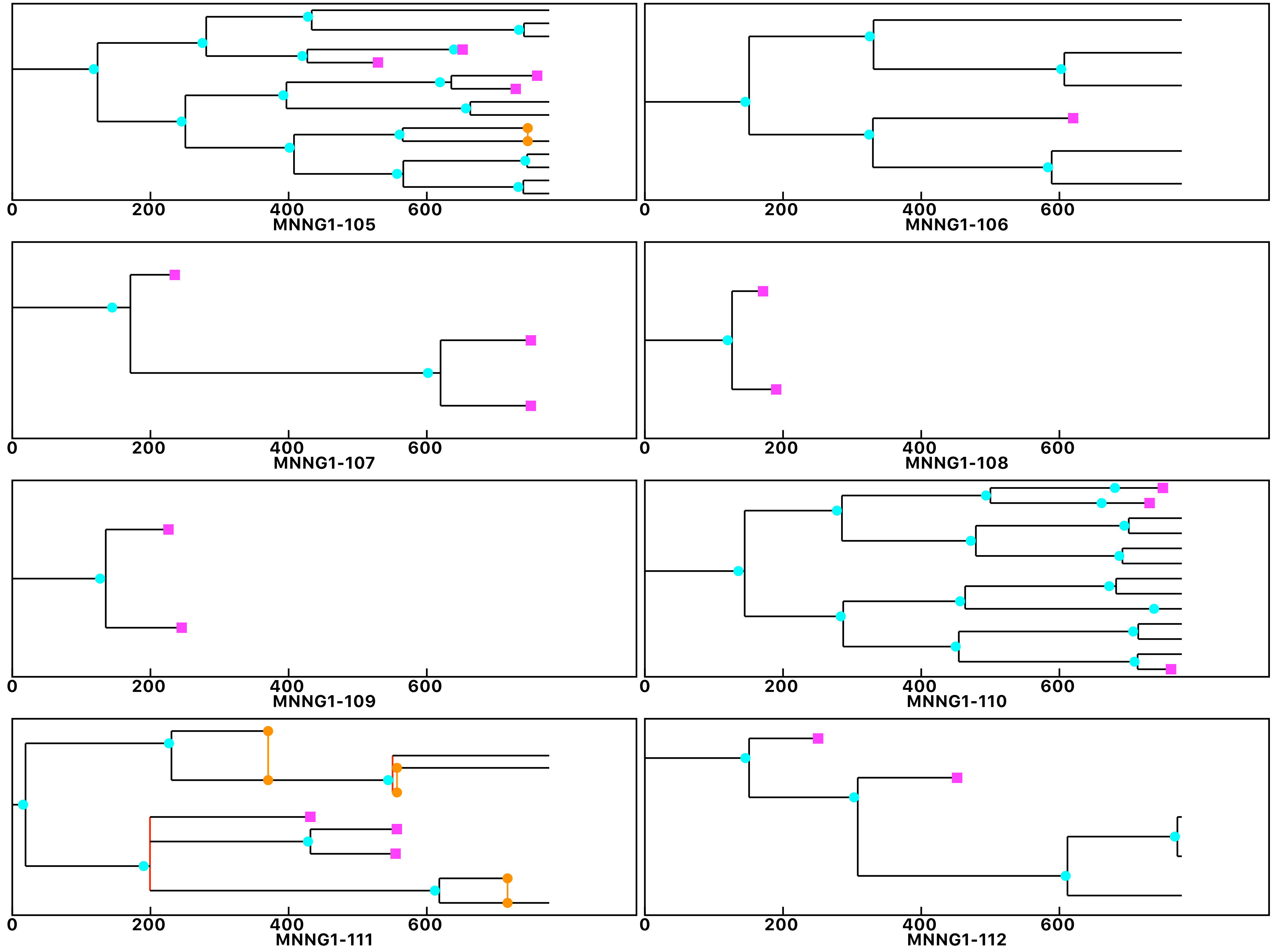

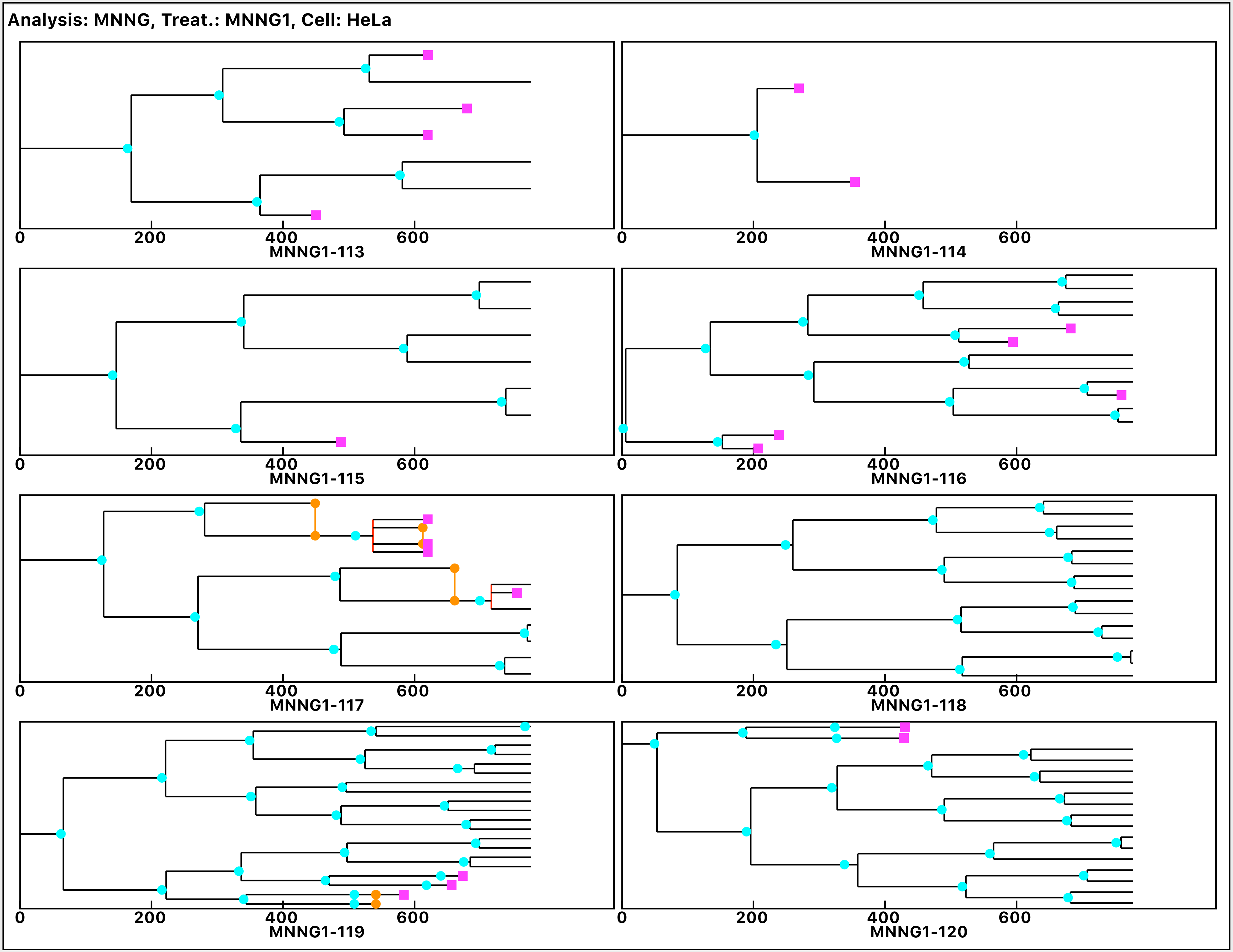
Analysis: MNNG, Treat.: MNNG1, Cell: HeLa
0
200
400
600
MNNG1-113
MNNG1-114
MNNG1-115
MNNG1-116
MNNG1-117
MNNG1-118
MNNG1-119
MNNG1-120

Analysis: MNNG, Treat.: MNNG1, Cell: HeLa

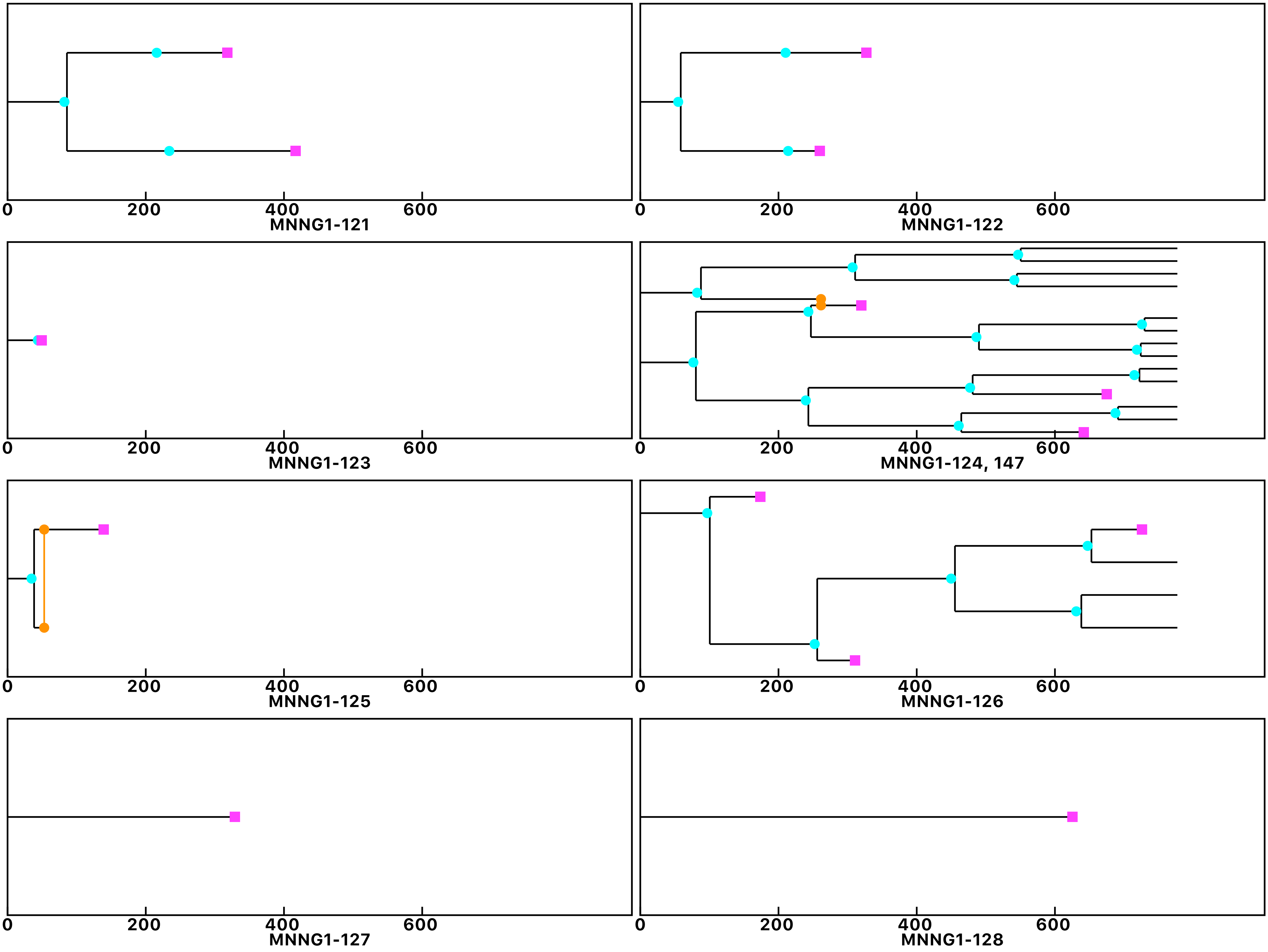

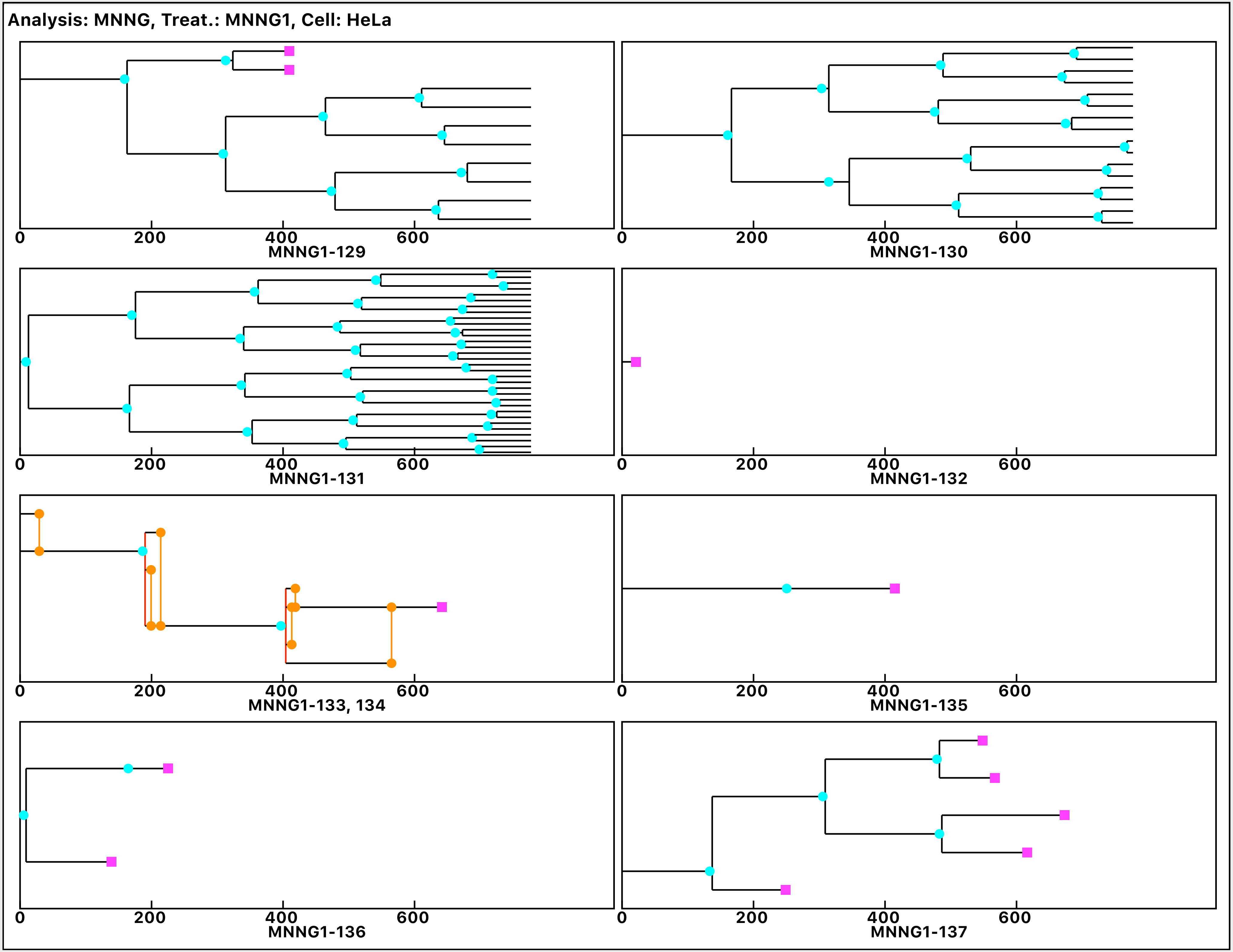

Analysis: MNNG, Treat.: MNNG1, Cell: HeLa
0
200
400
600
MNNG1-129
MNNG1-130
MNNG1-131
MNNG1-132
MNNG1-133, 134
MNNG1-135
MNNG1-136
MNNG1-137

Analysis: MNNG, Treat.: MNNG1, Cell: HeLa

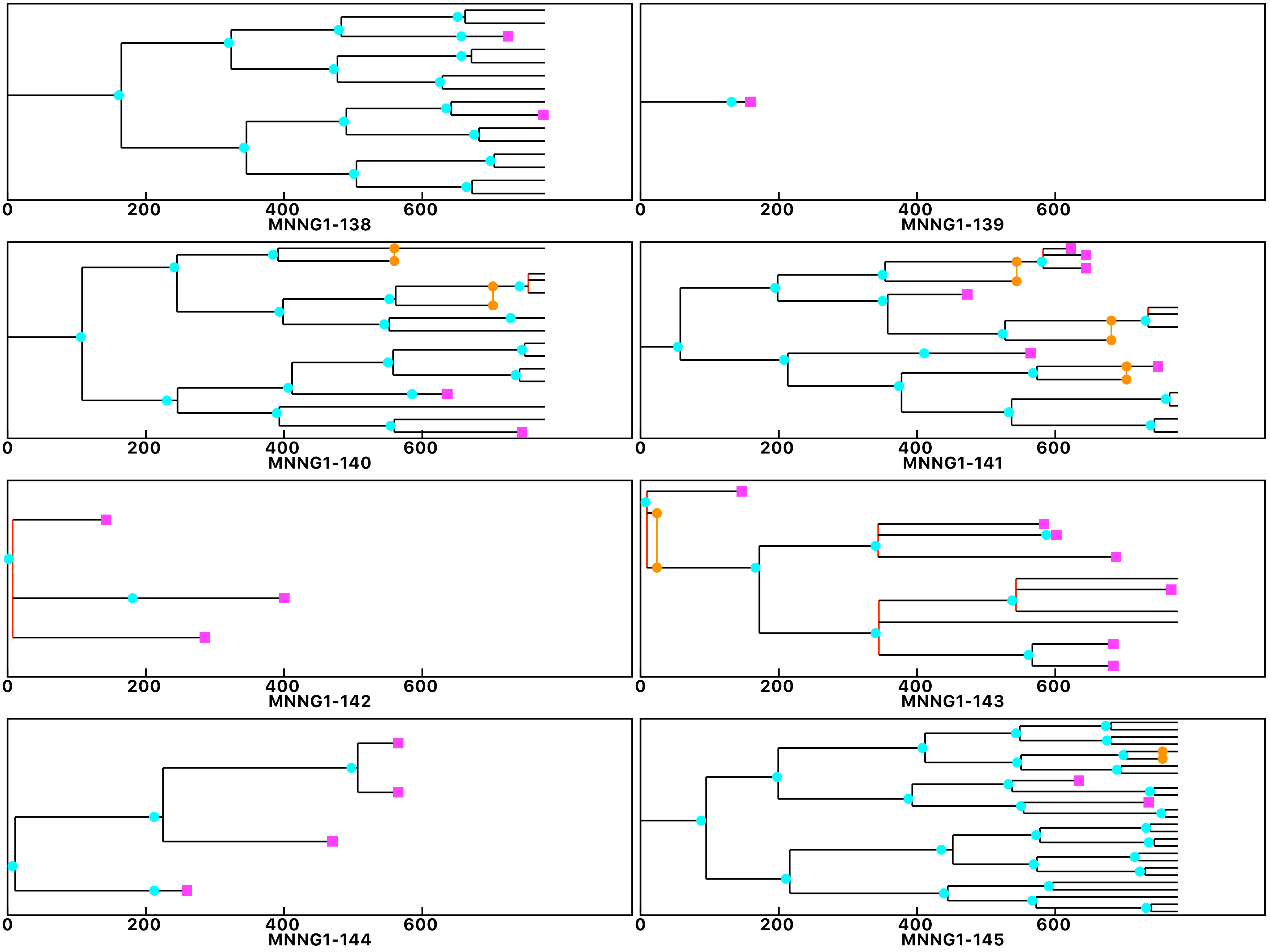

Analysis: MNNG, Treat.: MNNG1, Cell: HeLa

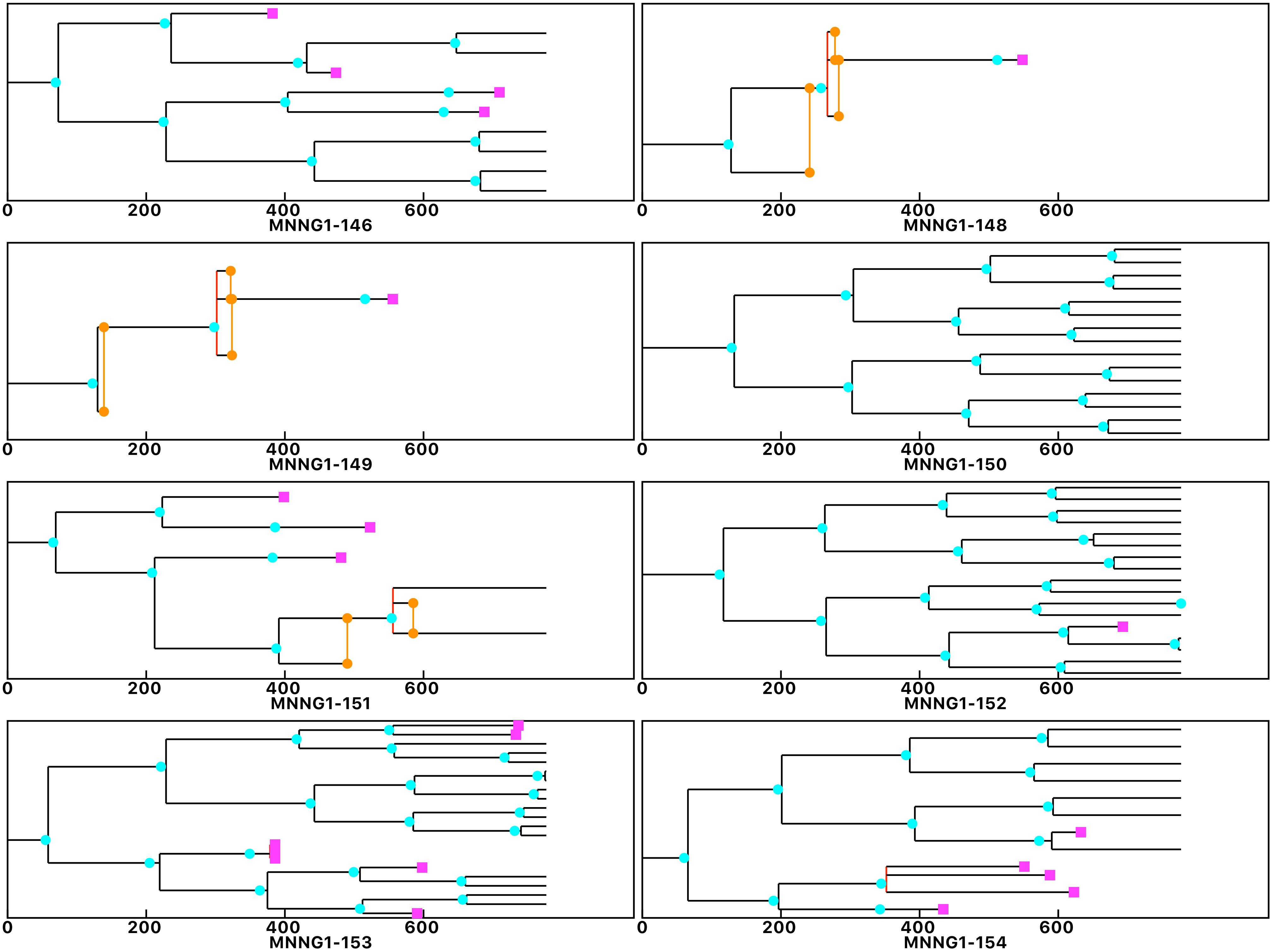

Analysis: MNNG, Treat.: MNNG1, Cell: HeLa

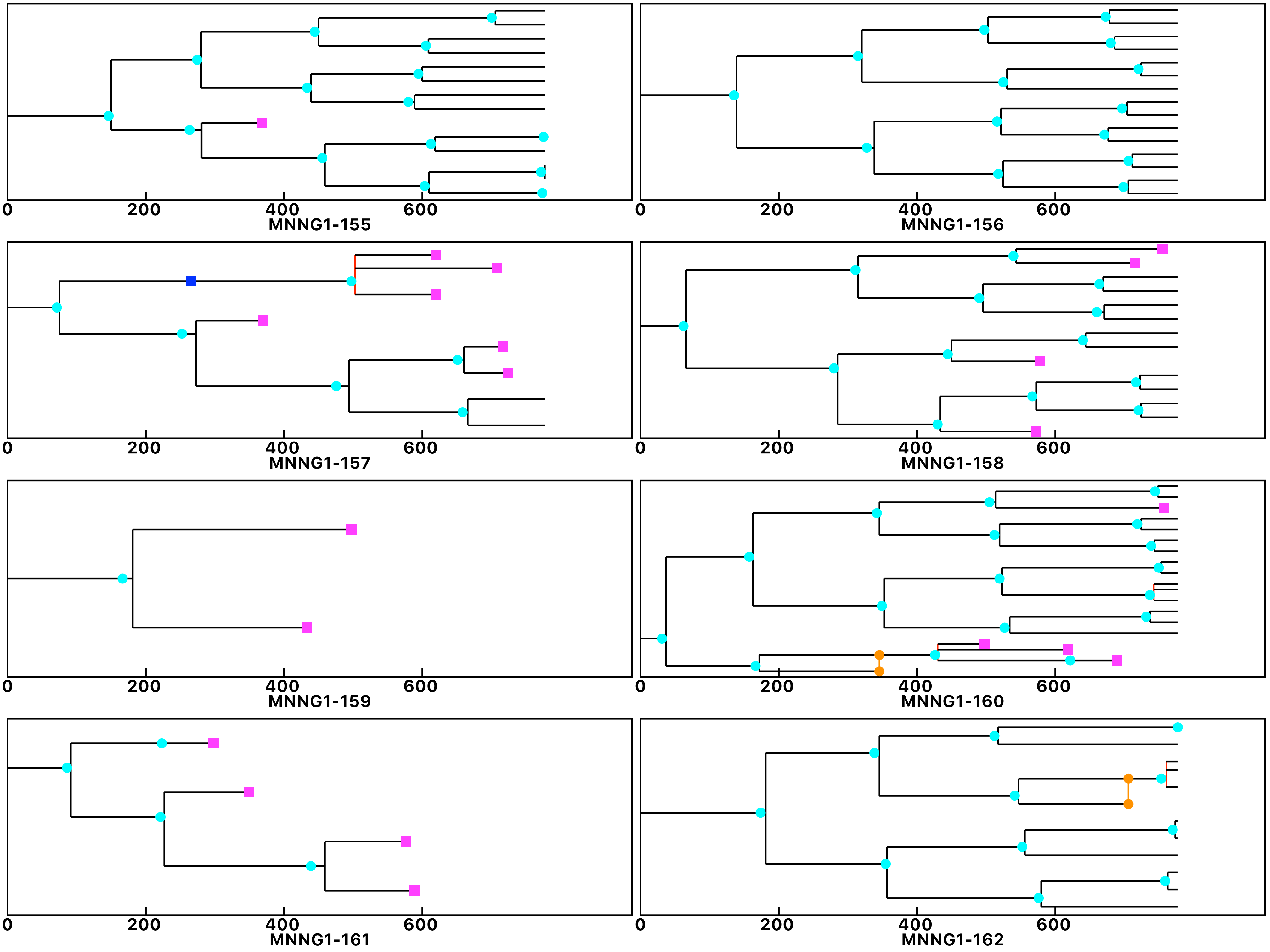

Analysis: MNNG, Treat.: MNNG1, Cell: HeLa

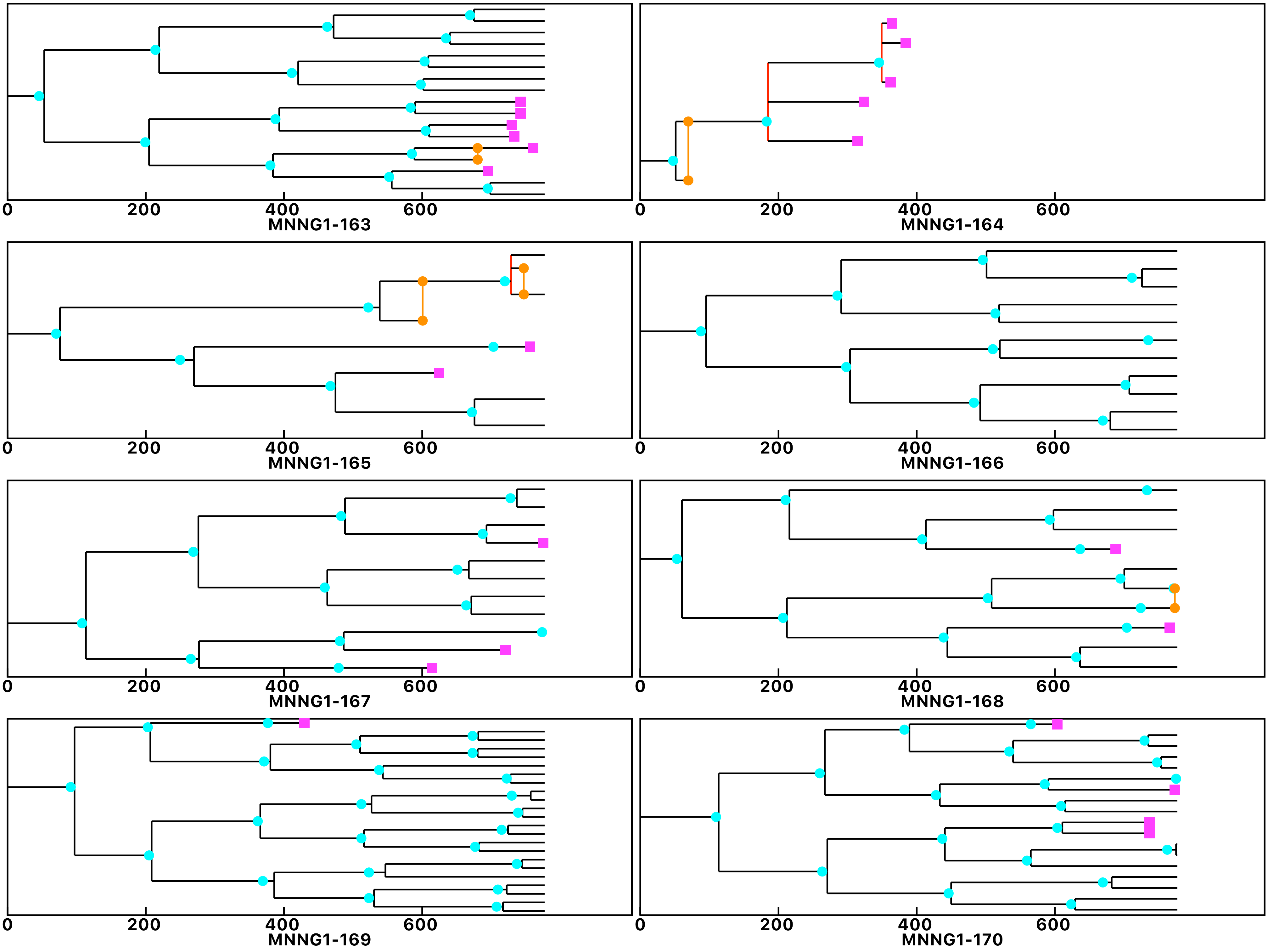

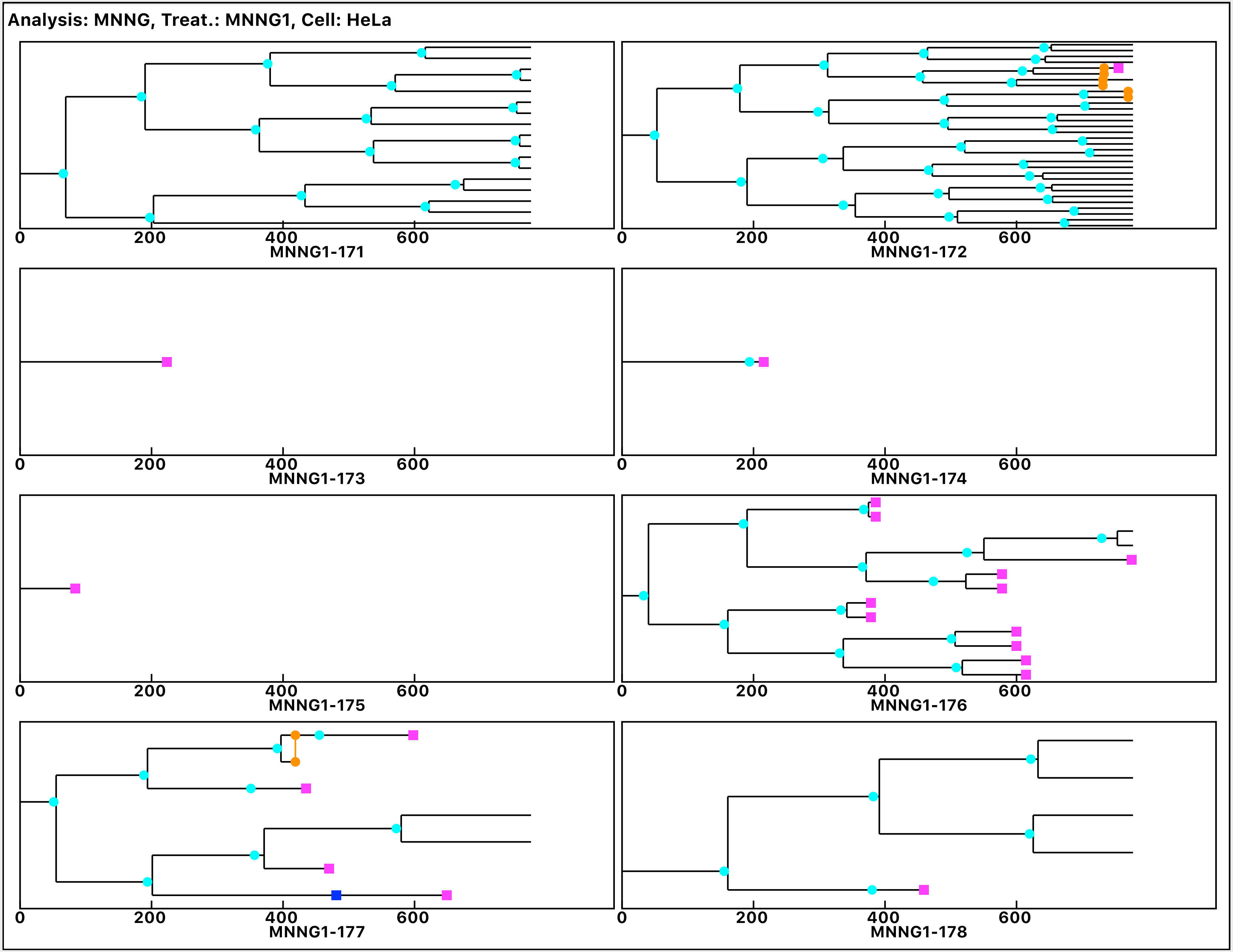
Analysis: MNNG, Treat.: MNNG1, Cell: HeLa
0
200
400
600
MNNG1-171
MNNG1-172
MNNG1-173
MNNG1-174
MNNG1-175
MNNG1-176
MNNG1-177
MNNG1-178

Analysis: MNNG, Treat.: MNNG1, Cell: HeLa

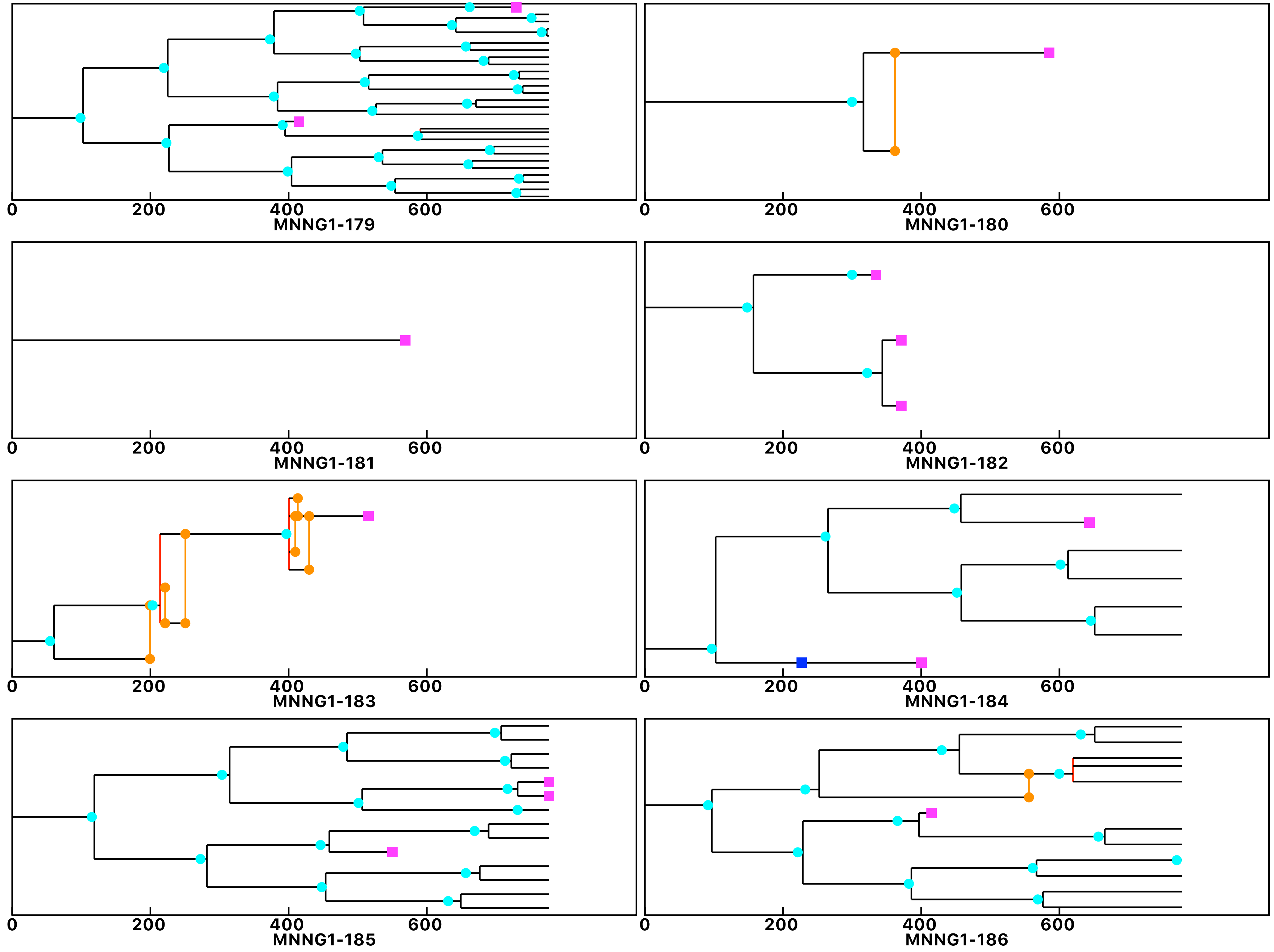

Analysis: MNNG, Treat.: MNNG1, Cell: HeLa

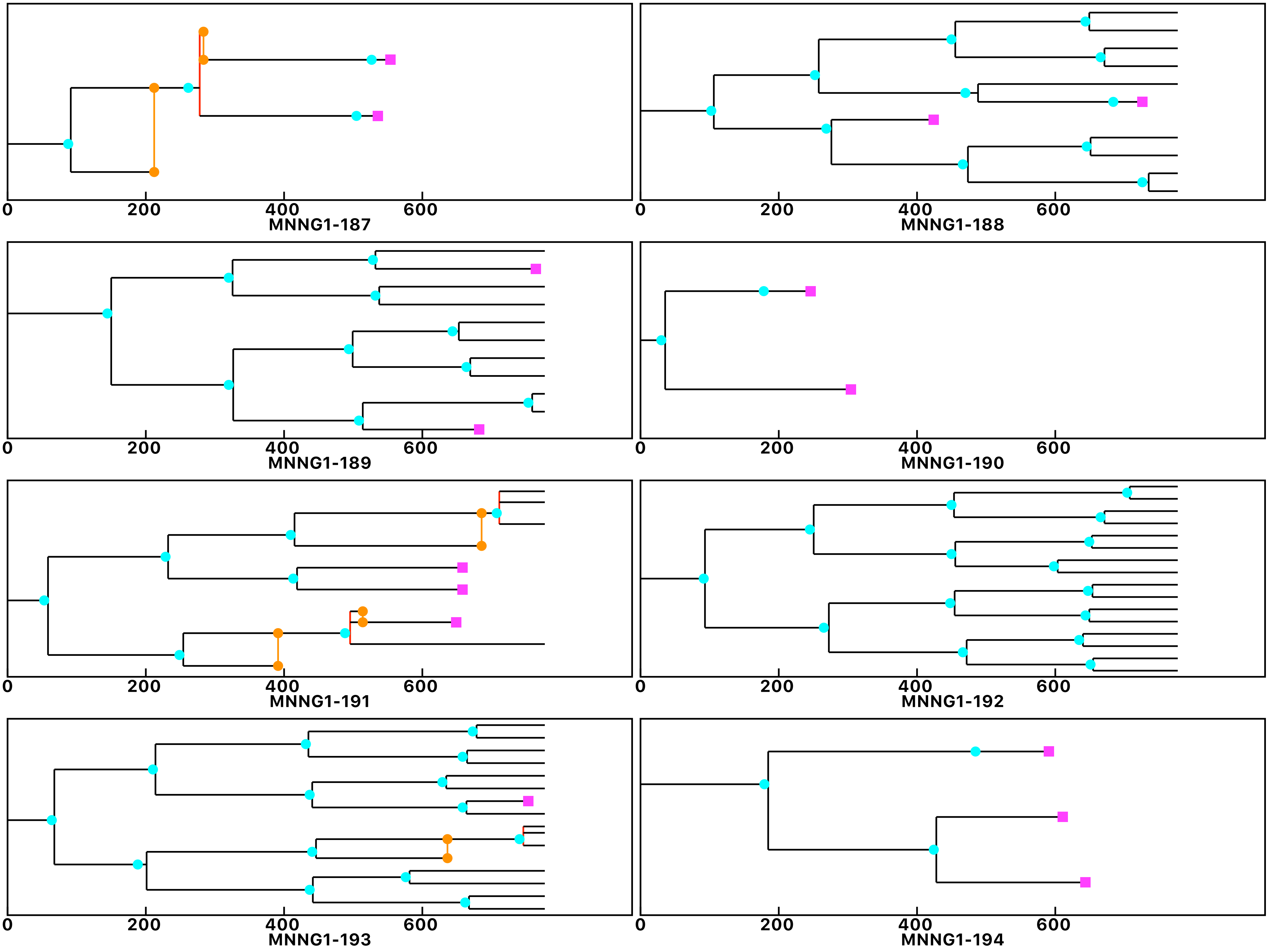

Analysis: MNNG, Treat.: MNNG1, Cell: HeLa

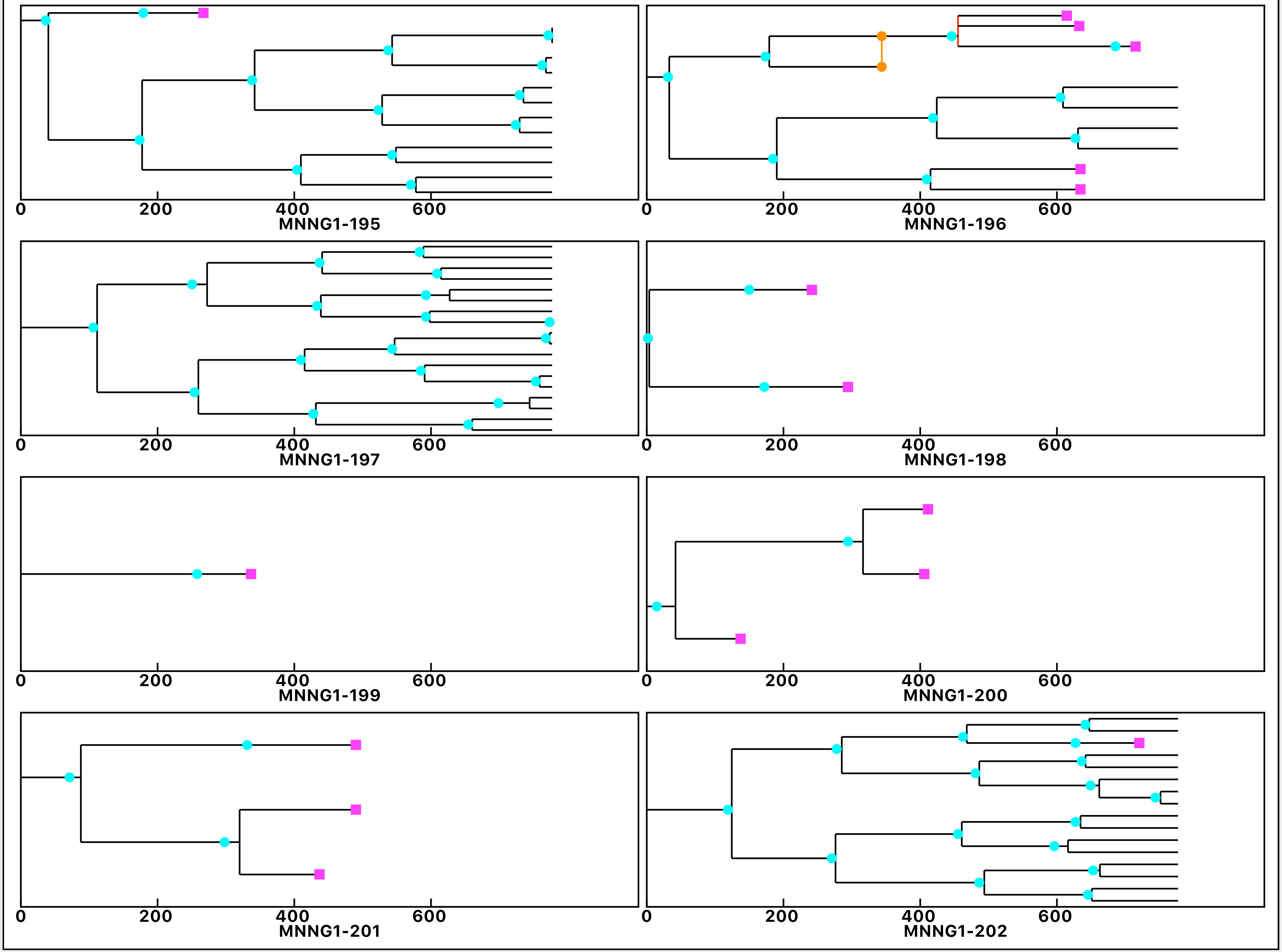

Analysis: MNNG, Treat.: MNNG1, Cell: HeLa

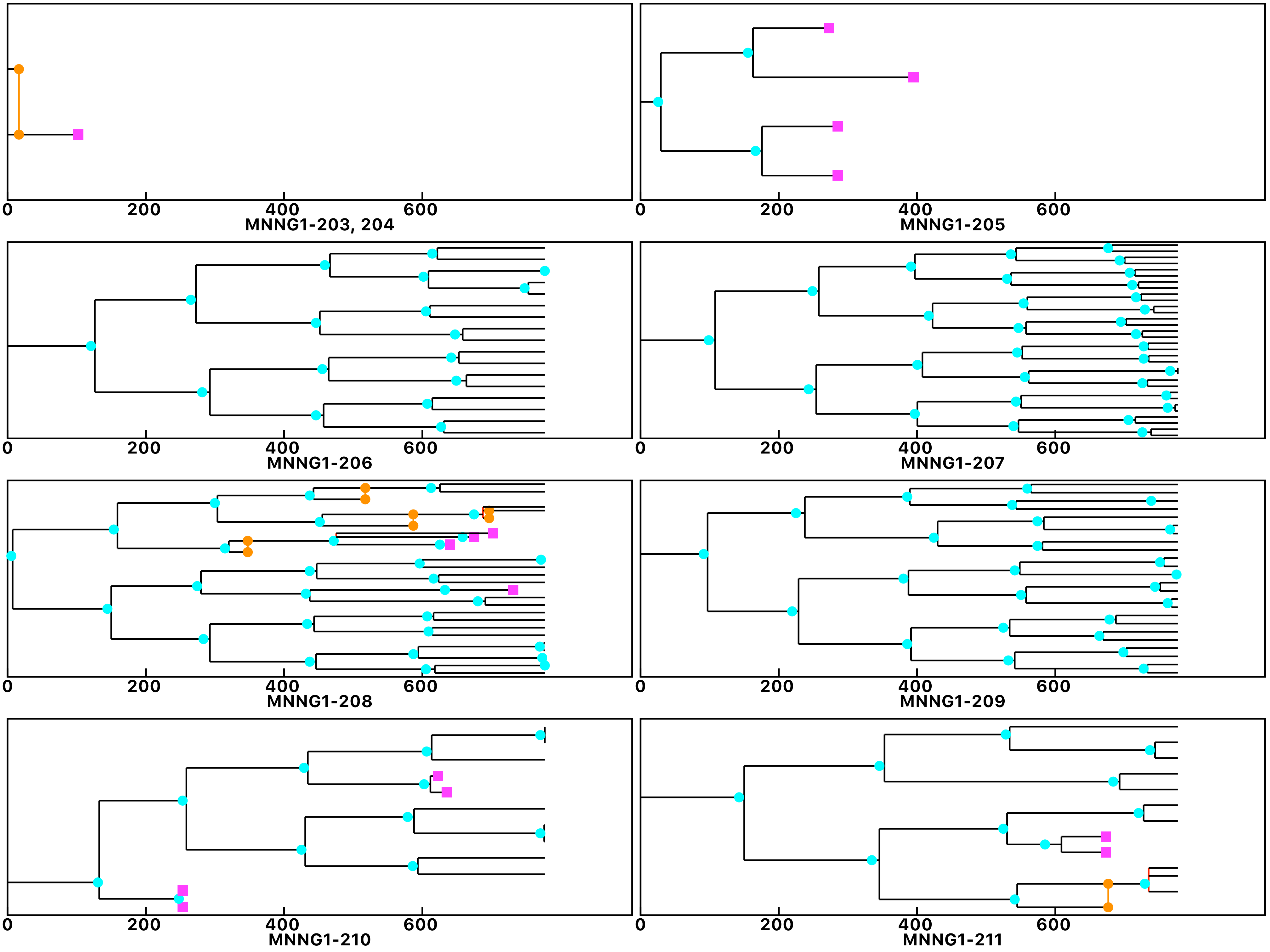

Analysis: MNNG, Treat.: MNNG1, Cell: HeLa

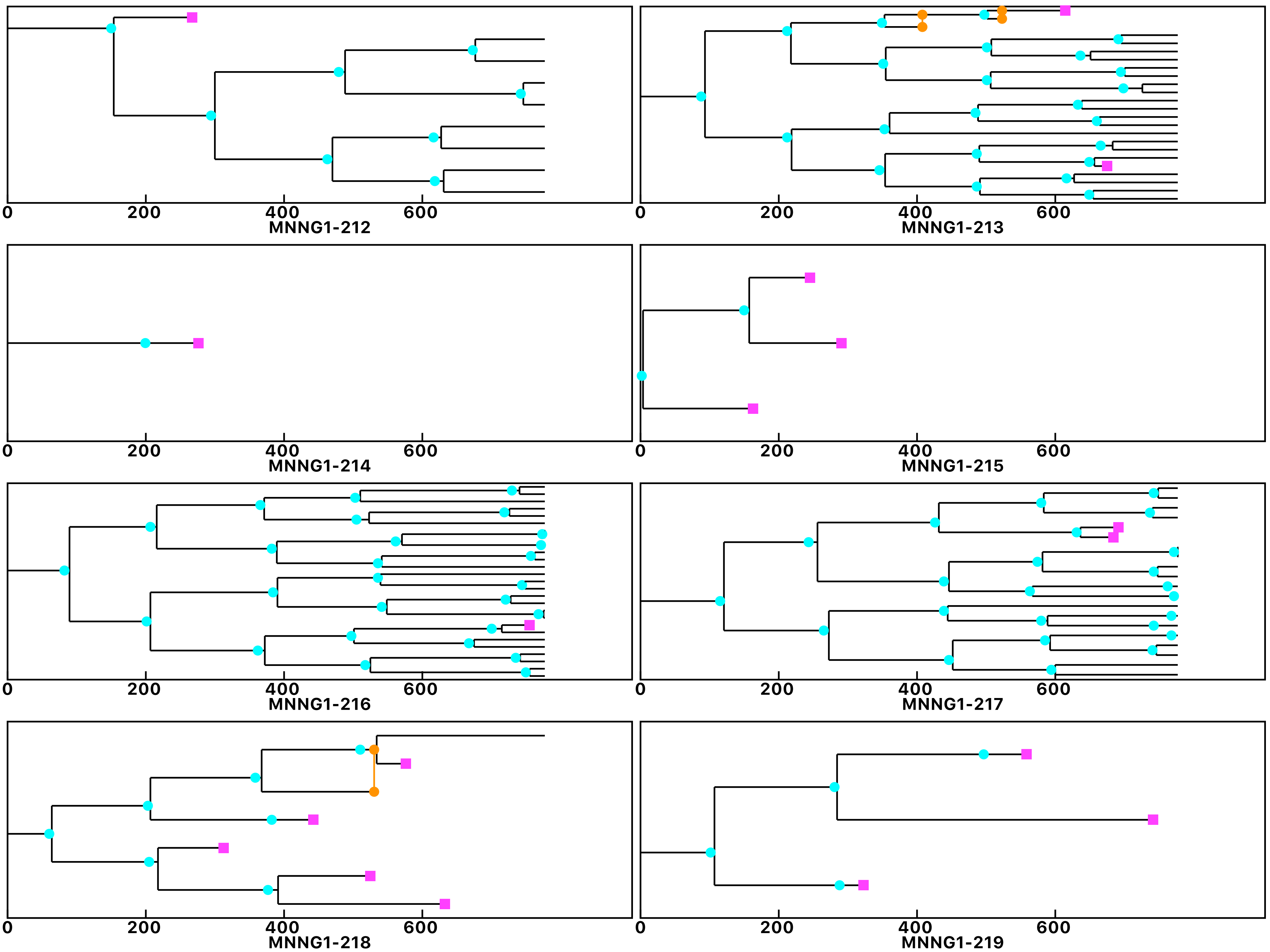

Analysis: MNNG, Treat.: MNNG1, Cell: HeLa

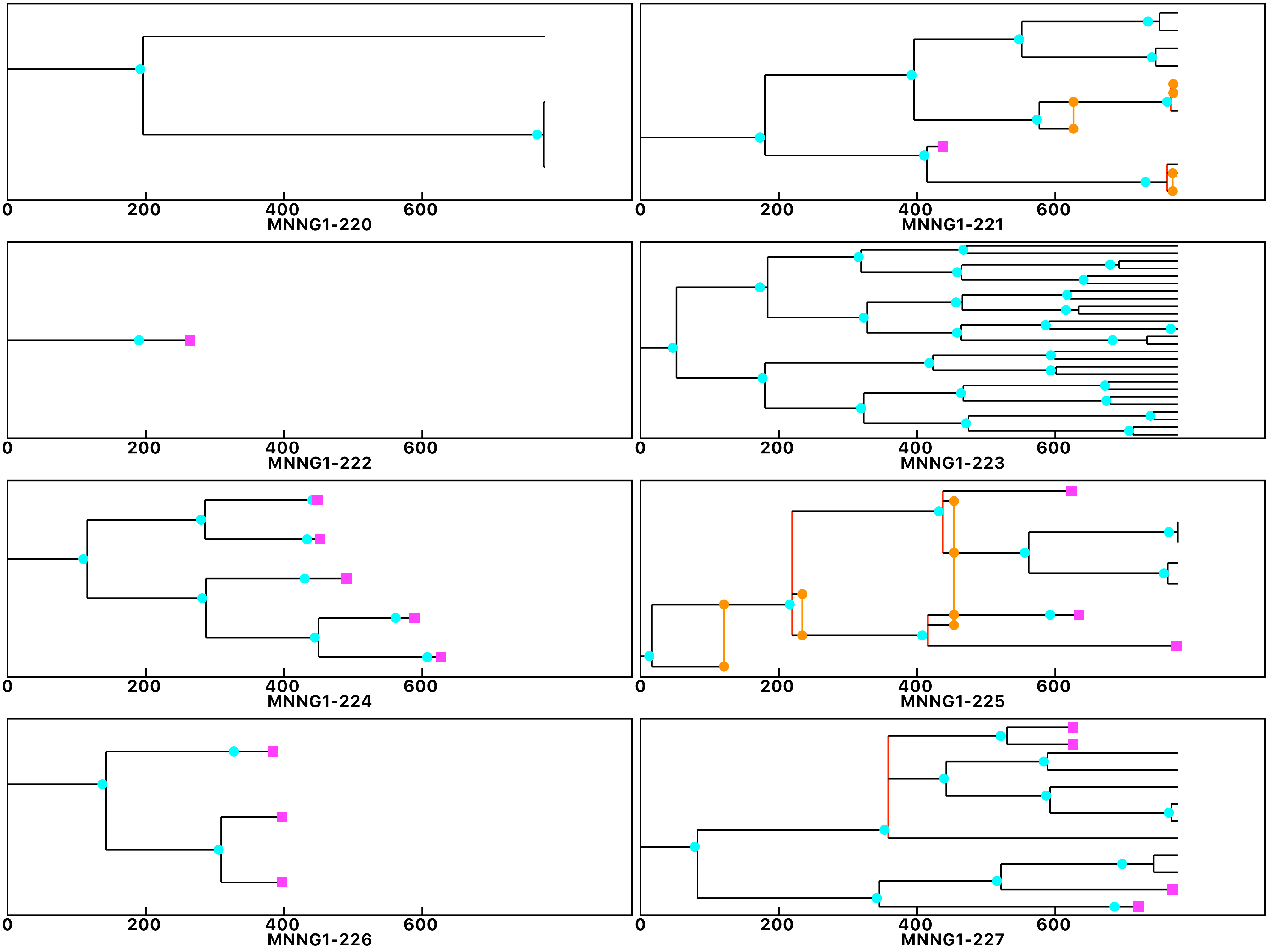

Analysis: MNNG, Treat.: MNNG1, Cell: HeLa

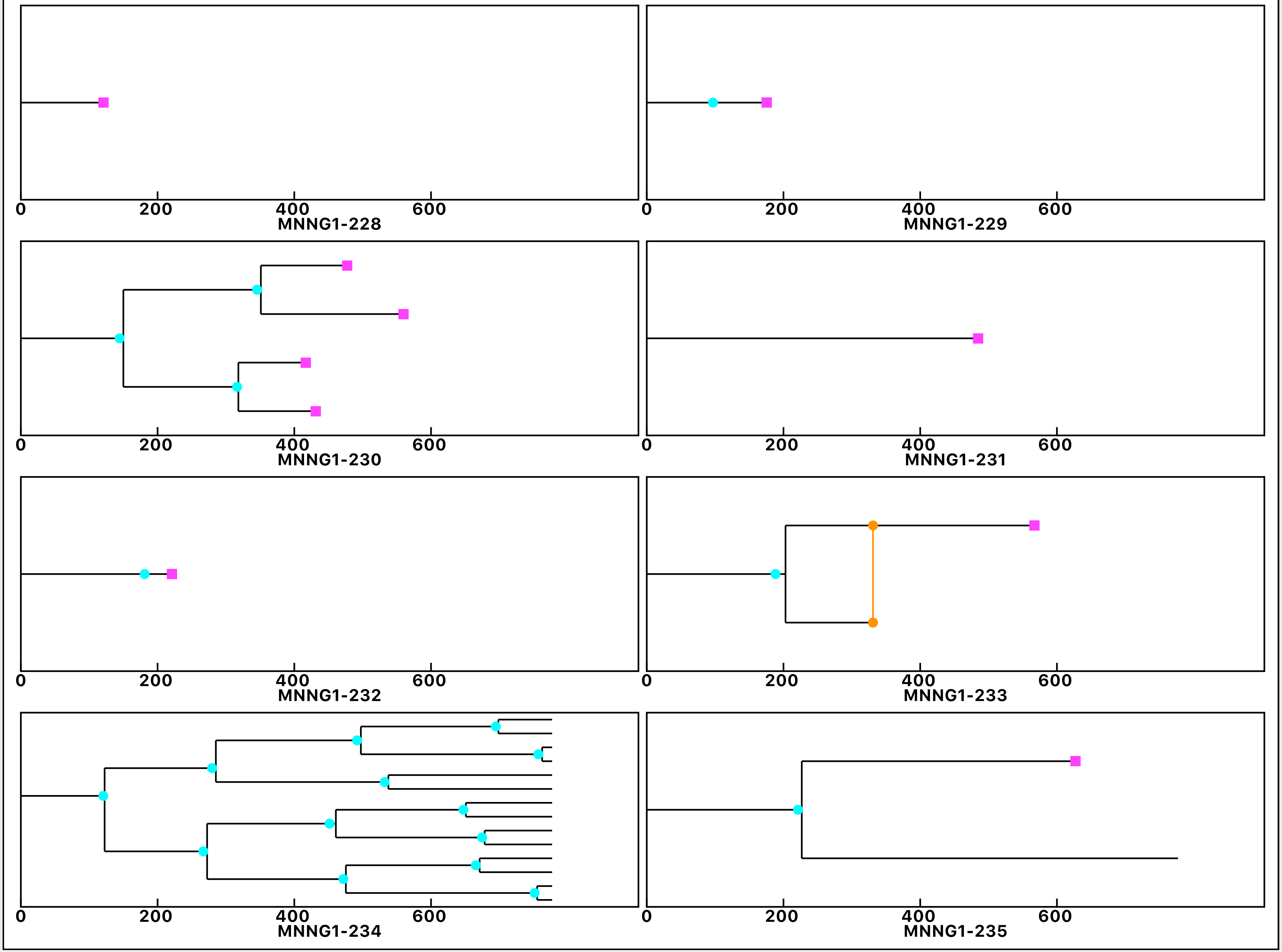

Analysis: MNNG, Treat.: MNNG1, Cell: HeLa

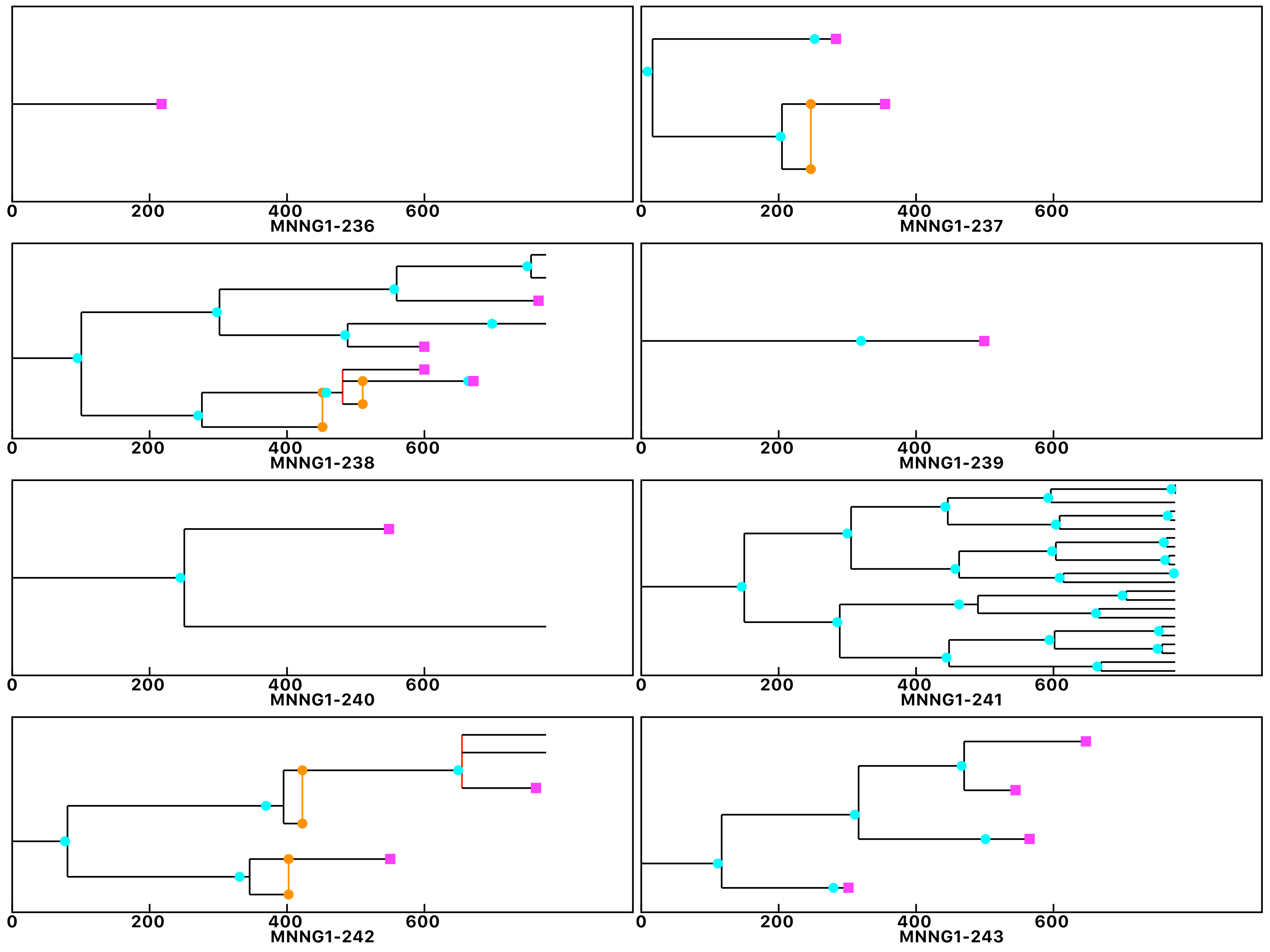

Analysis: MNNG, Treat.: MNNG1, Cell: HeLa

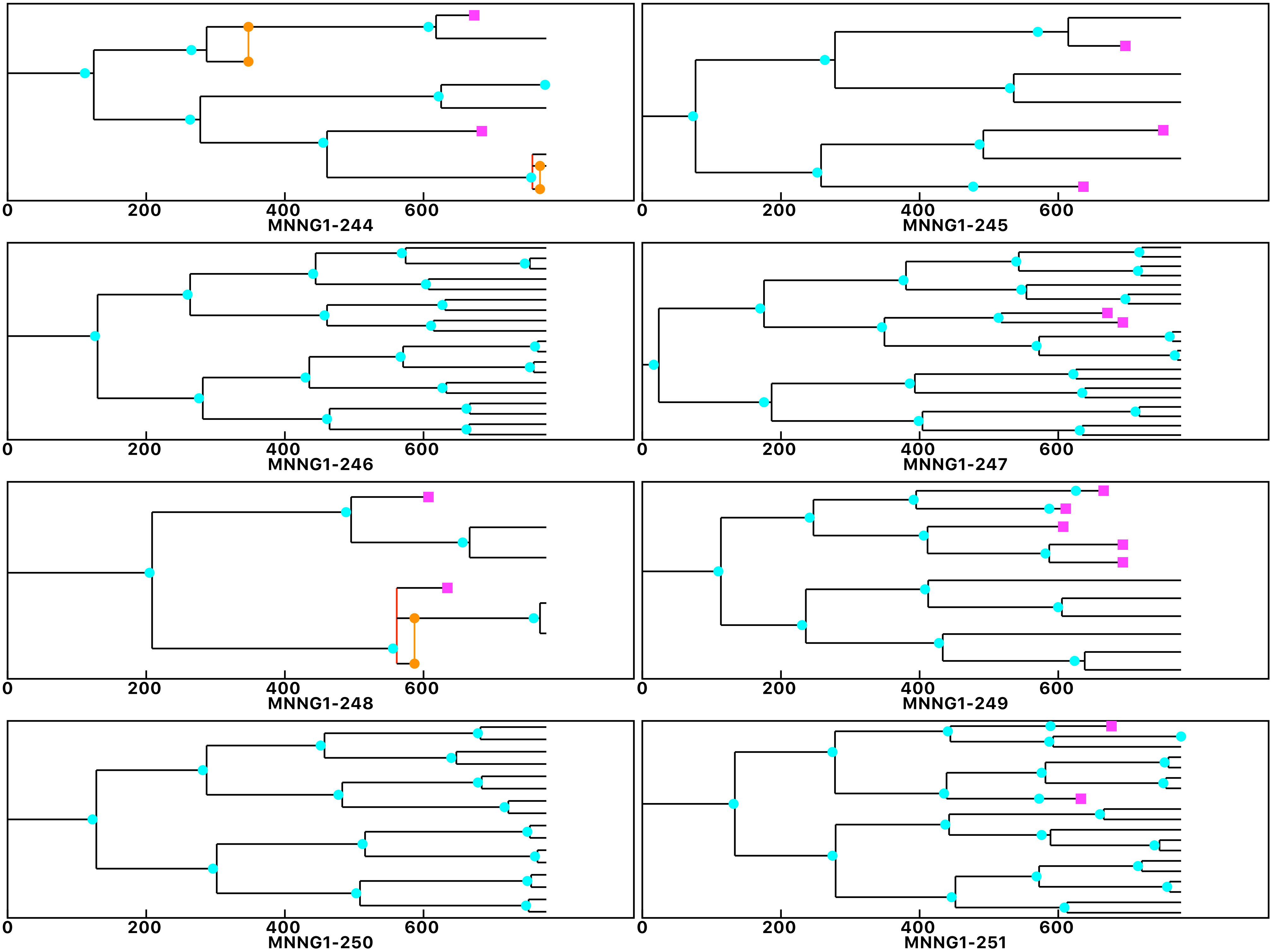

Analysis: MNNG, Treat.: MNNG1, Cell: HeLa

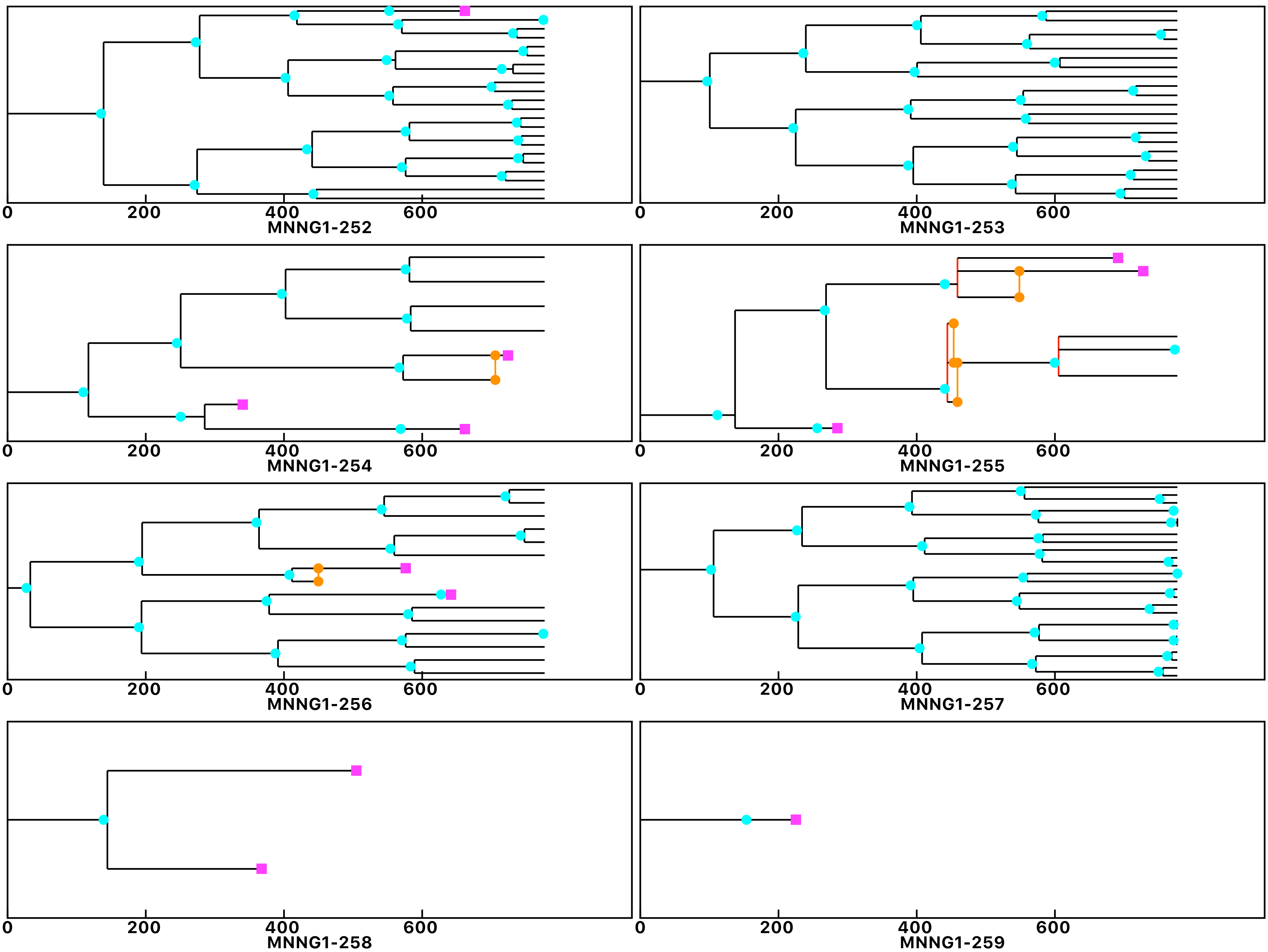

Analysis: MNNG, Treat.: MNNG1, Cell: HeLa

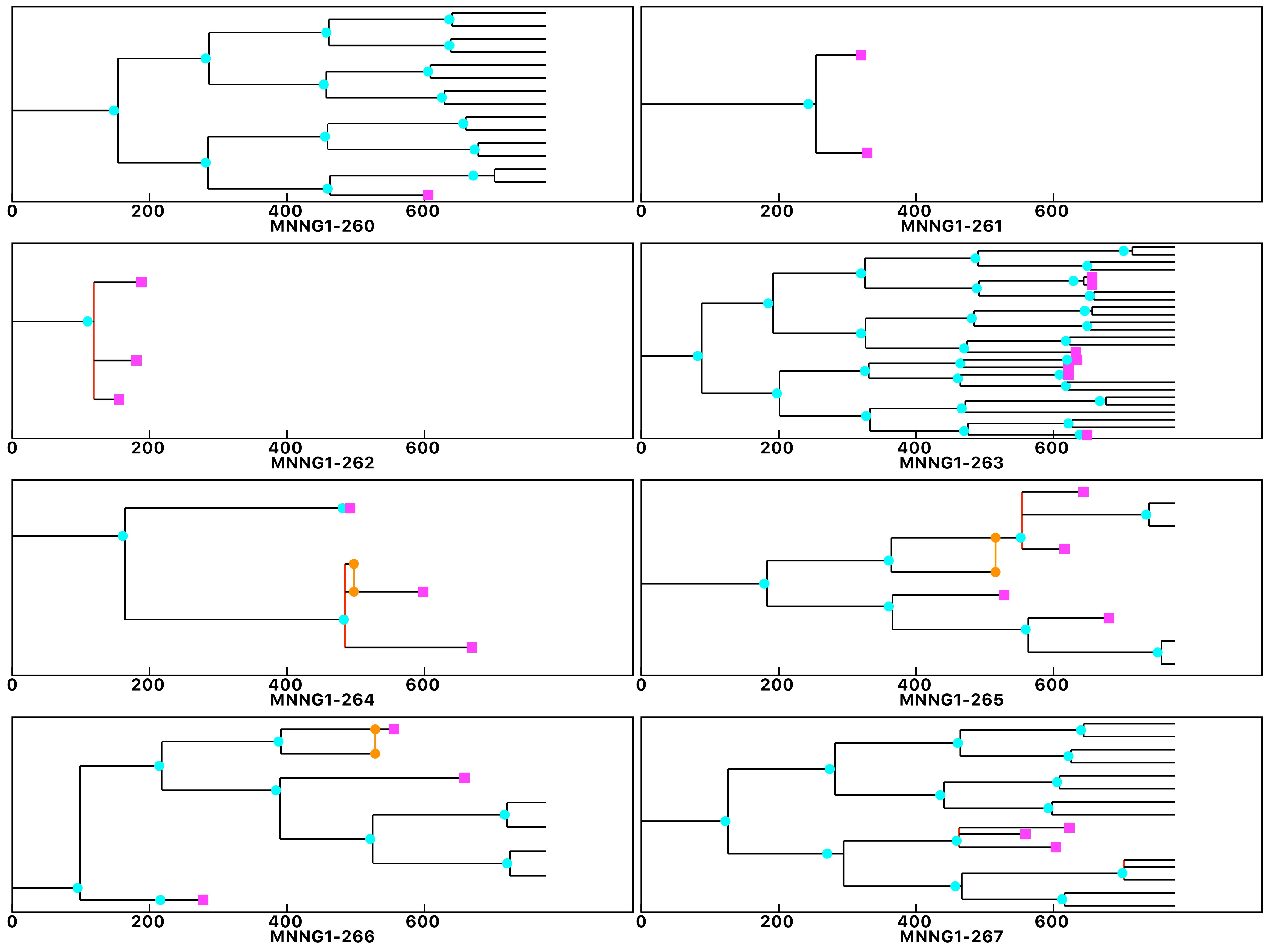

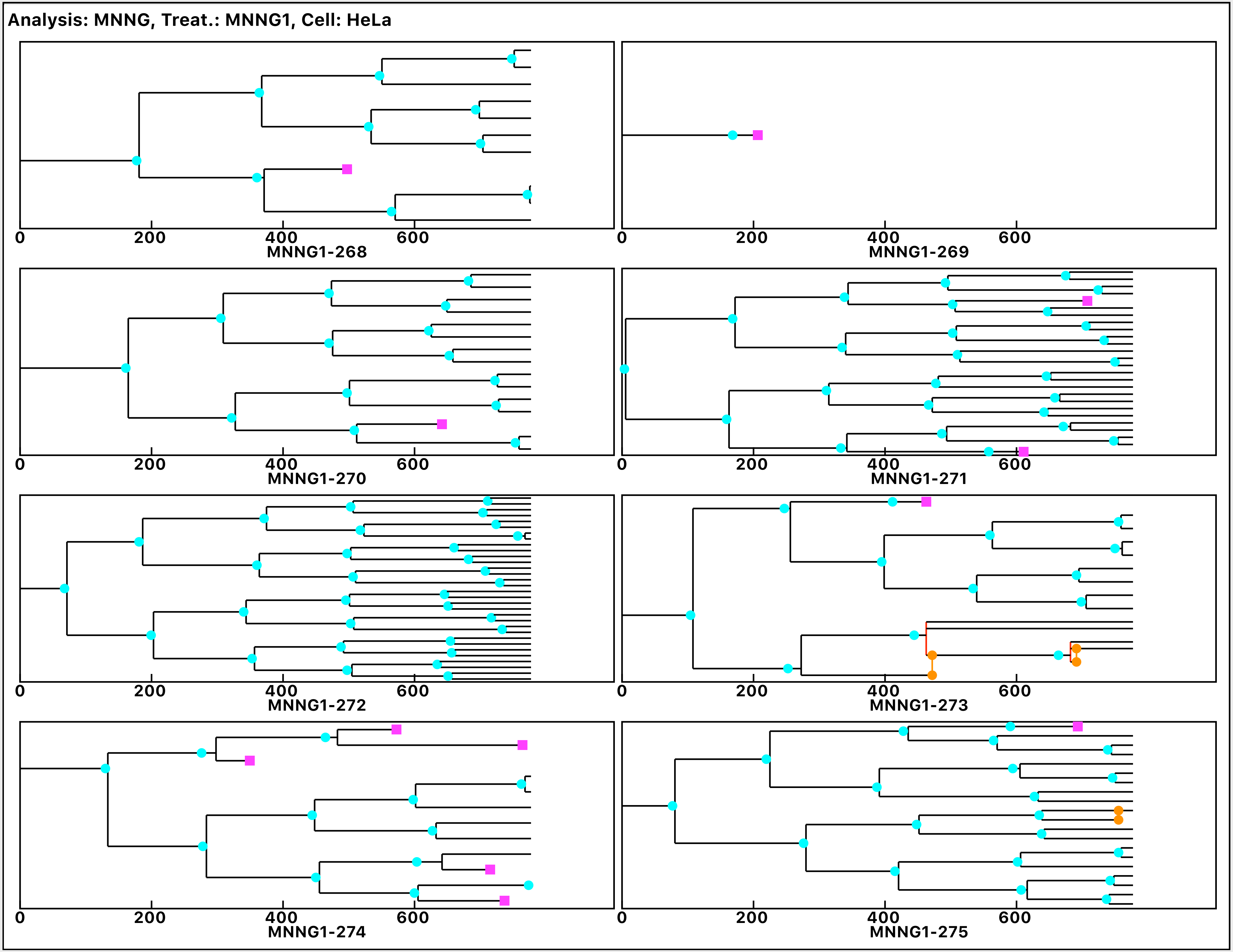

Analysis: MNNG, Treat.: MNNG1, Cell: HeLa
0
200
400
600
MNNG1-268
MNNG1-269
MNNG1-270
MNNG1-271
MNNG1-272
MNNG1-273
MNNG1-274
MNNG1-275

Analysis: MNNG, Treat.: MNNG1, Cell: HeLa

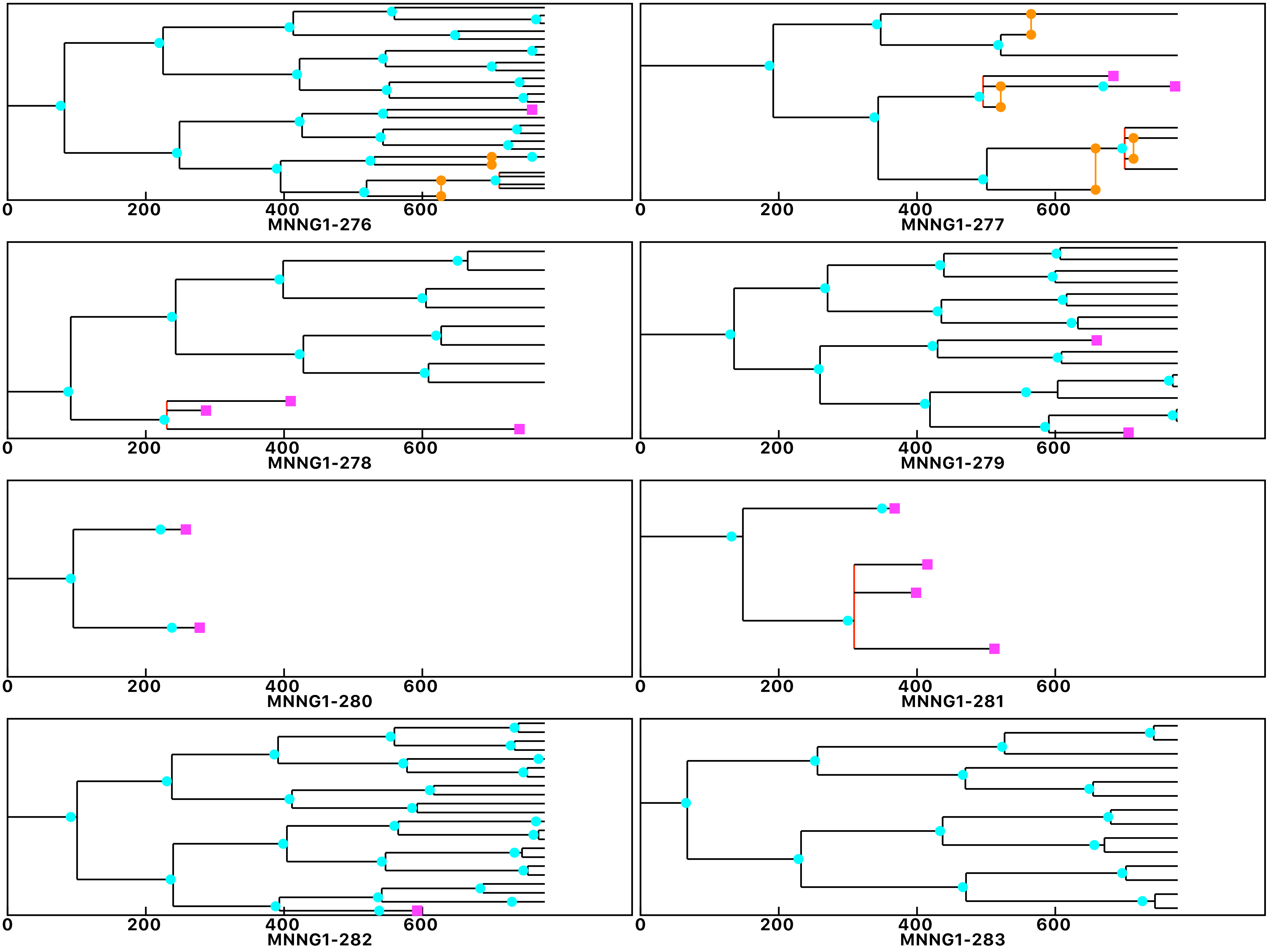

Analysis: MNNG, Treat.: MNNG1, Cell: HeLa

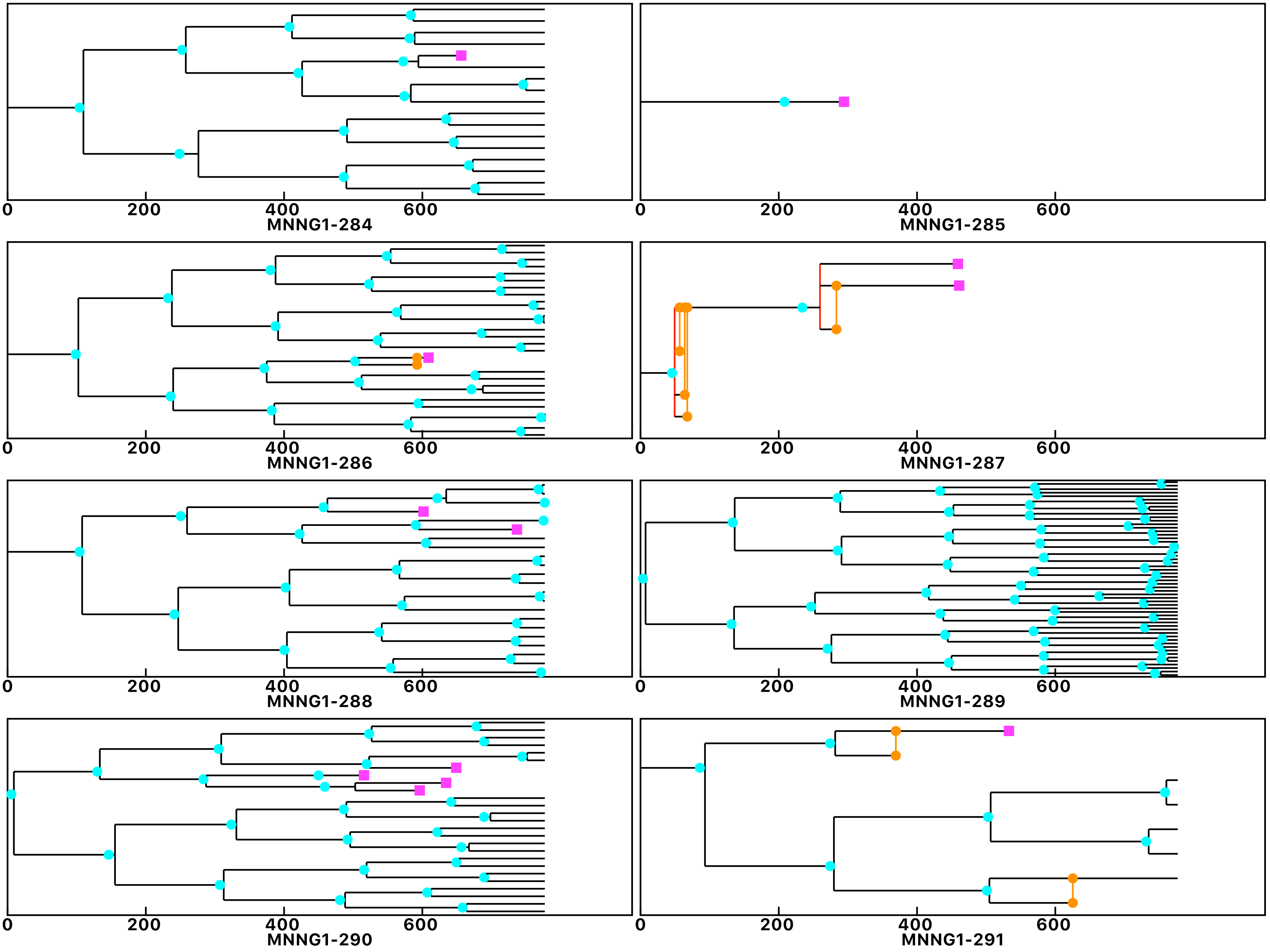

Analysis: MNNG, Treat.: MNNG1, Cell: HeLa

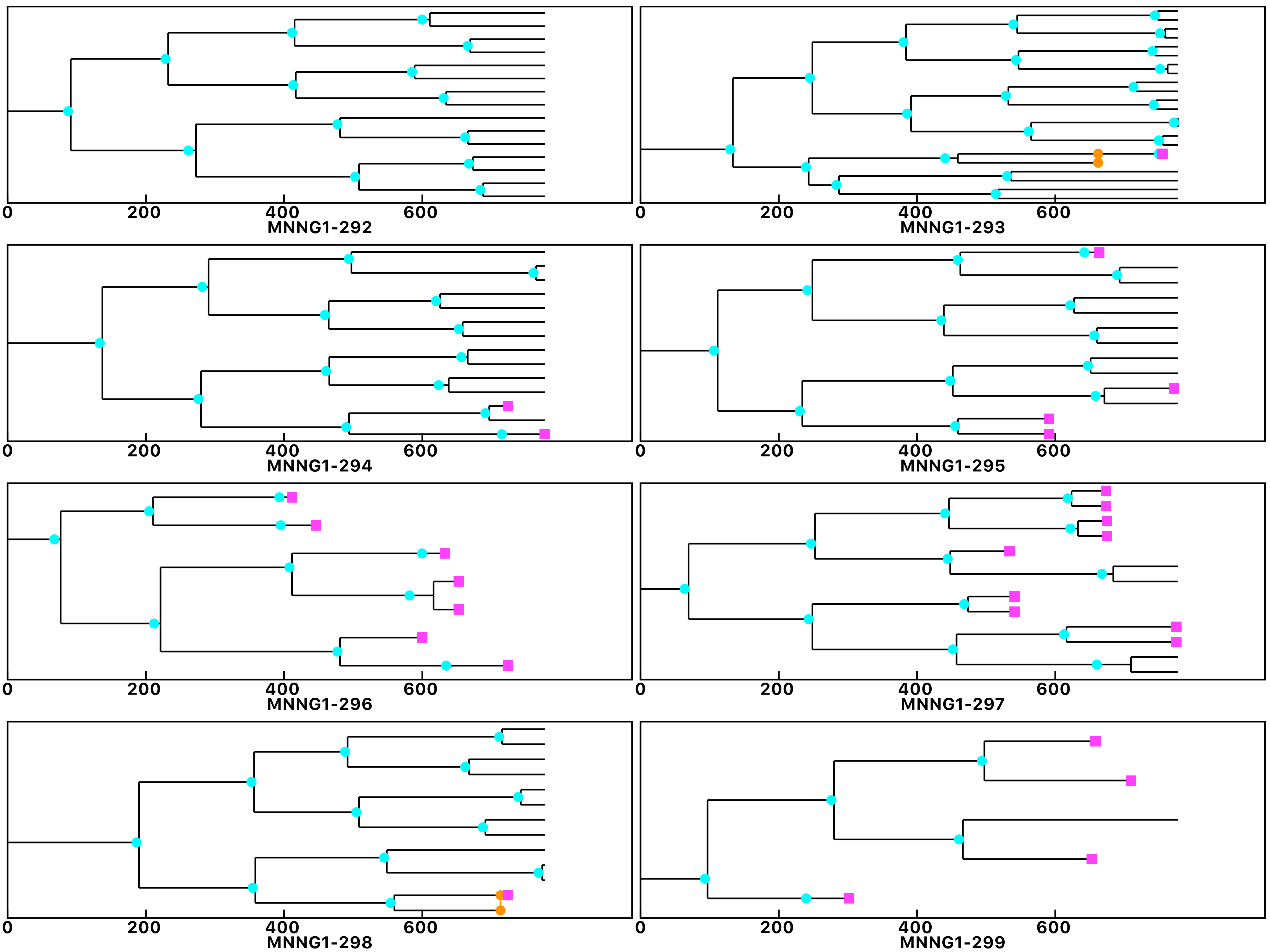

Analysis: MNNG, Treat.: MNNG1, Cell: HeLa

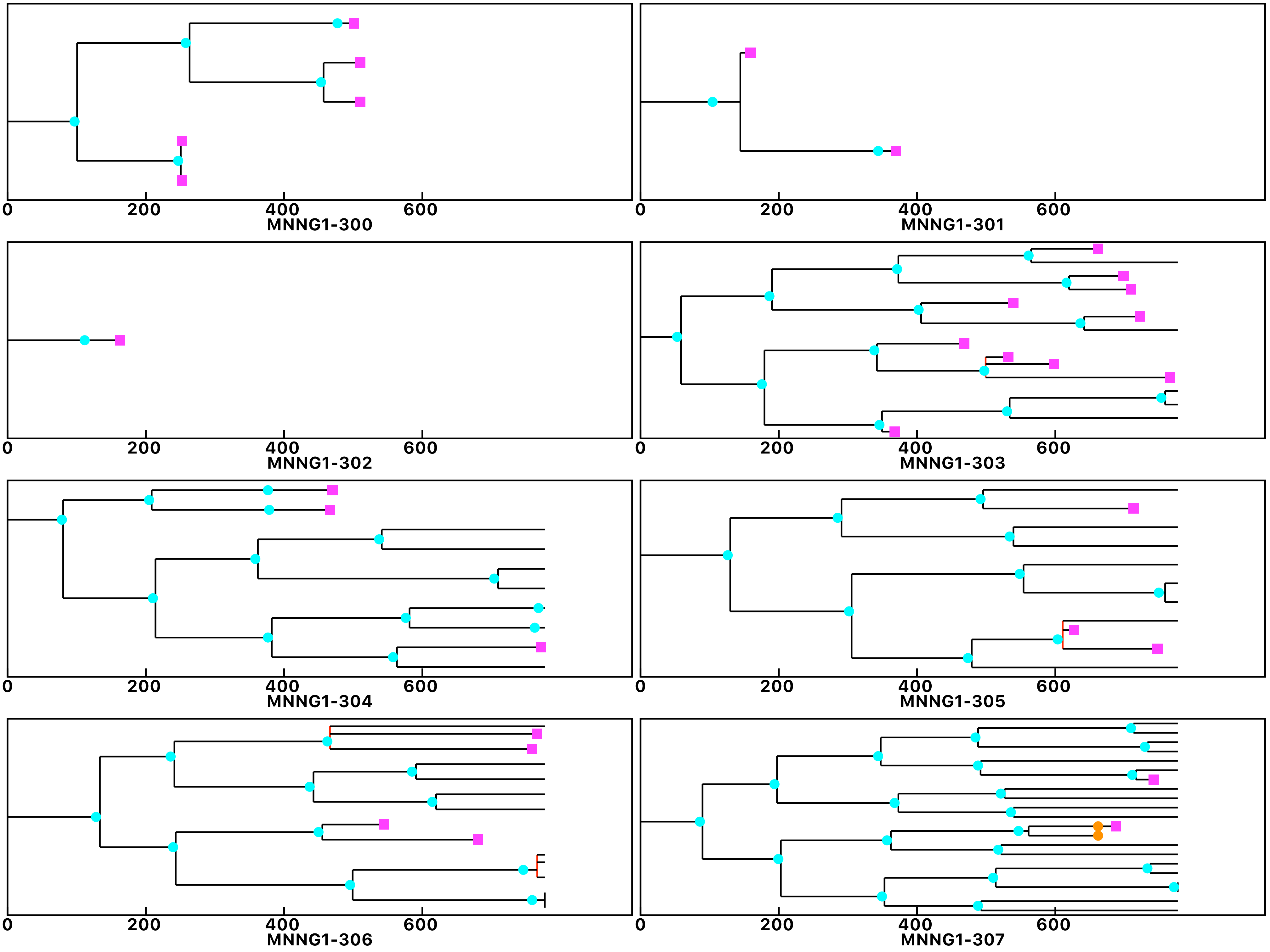

Analysis: MNNG, Treat.: MNNG1, Cell: HeLa

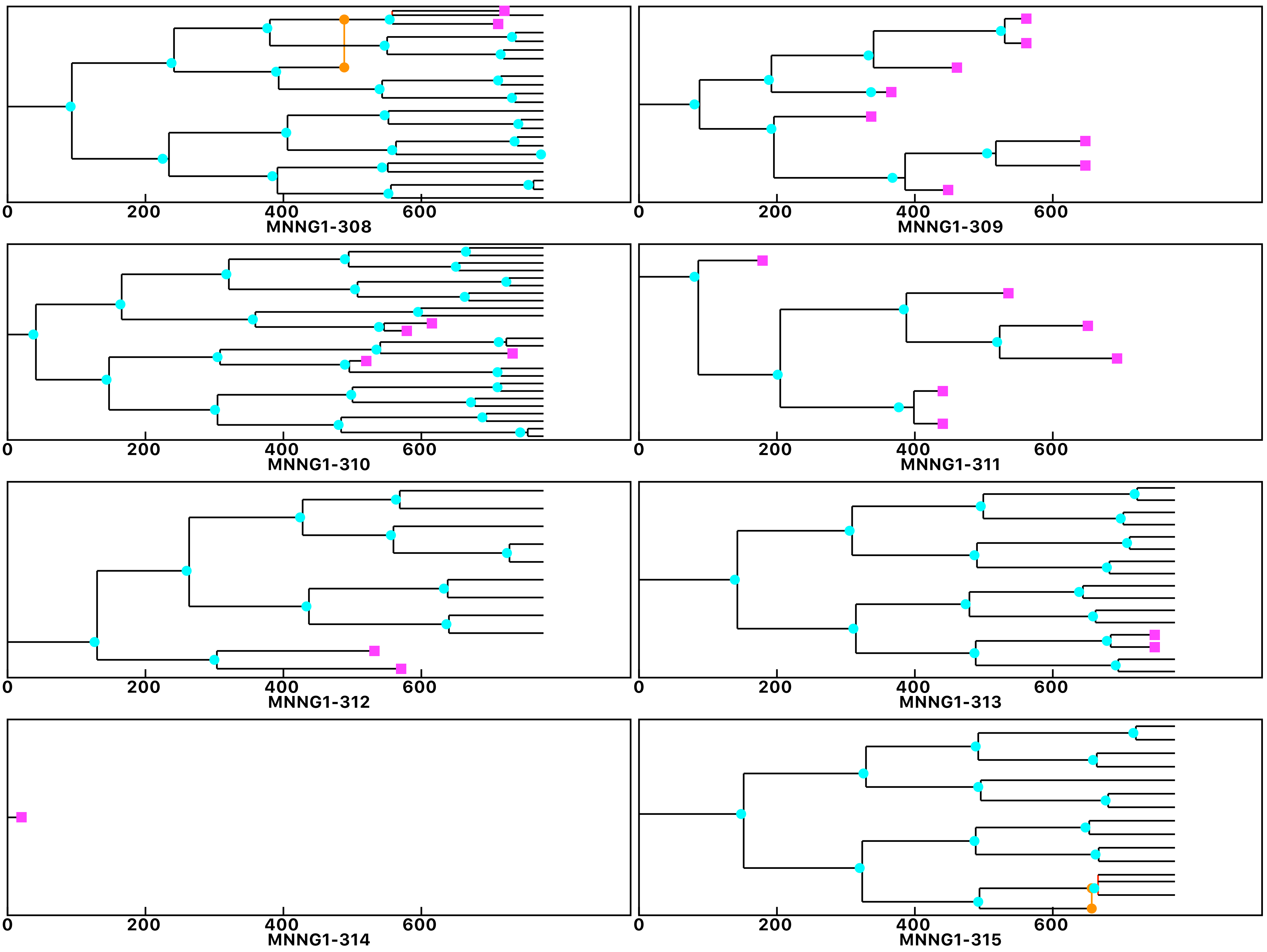

Analysis: MNNG, Treat.: MNNG1, Cell: HeLa

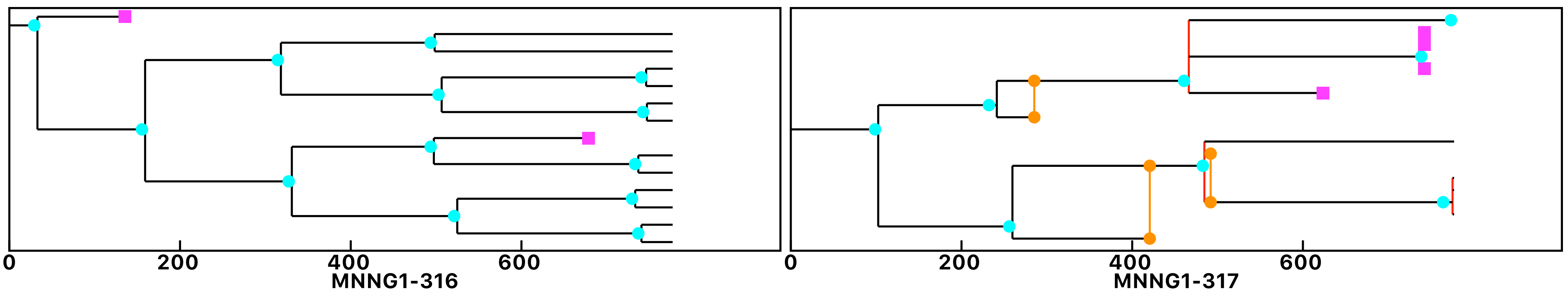

Supplement: S2 Fig — Cell lineage maps (MNNG-1μM: MNNG1) are shown. Light blue circle: mitosis, orange circle: cell fusion, pink square: cell death, blue square: incomplete cell division, black vertical line: bipolar cell division, red vertical line: multipolar cell division, and orange vertical line: cell fusion. (PDF) [file pone.0214512.s002.pdf]
