# Supplementary material for: Dose-dependent spatiotemporal responses of mammalian cells to an alkylating agent
Source: PLoS One. 2019 Mar 29;14(3):e0214512. doi: 10.1371/journal.pone.0214512 (PMC6440626; doi:10.1371/journal.pone.0214512)

Supplementary Figure S3

Analysis: MNNG, Treat.: MNNG2, Cell: HeLa

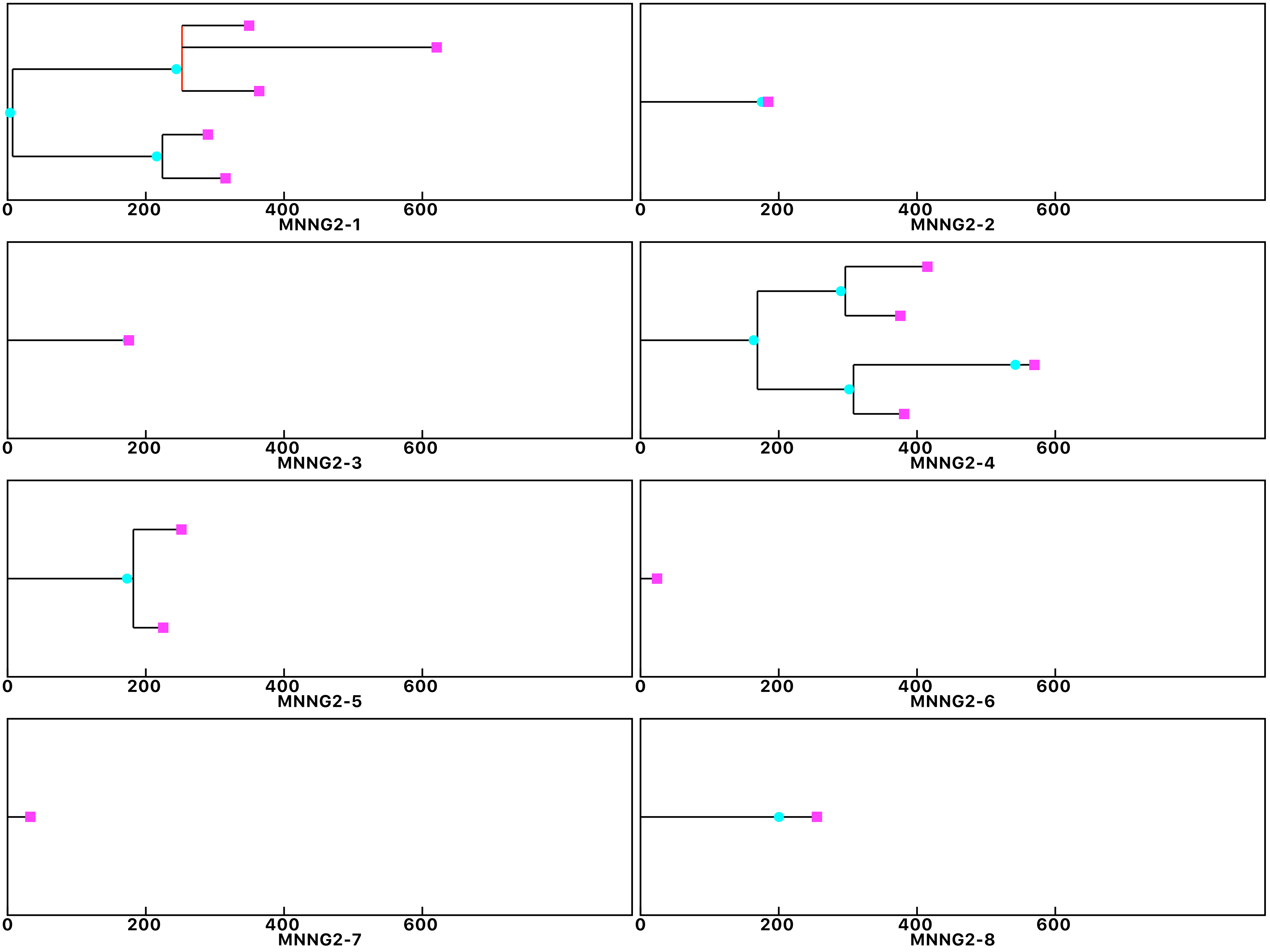

Analysis: MNNG, Treat.: MNNG2, Cell: HeLa

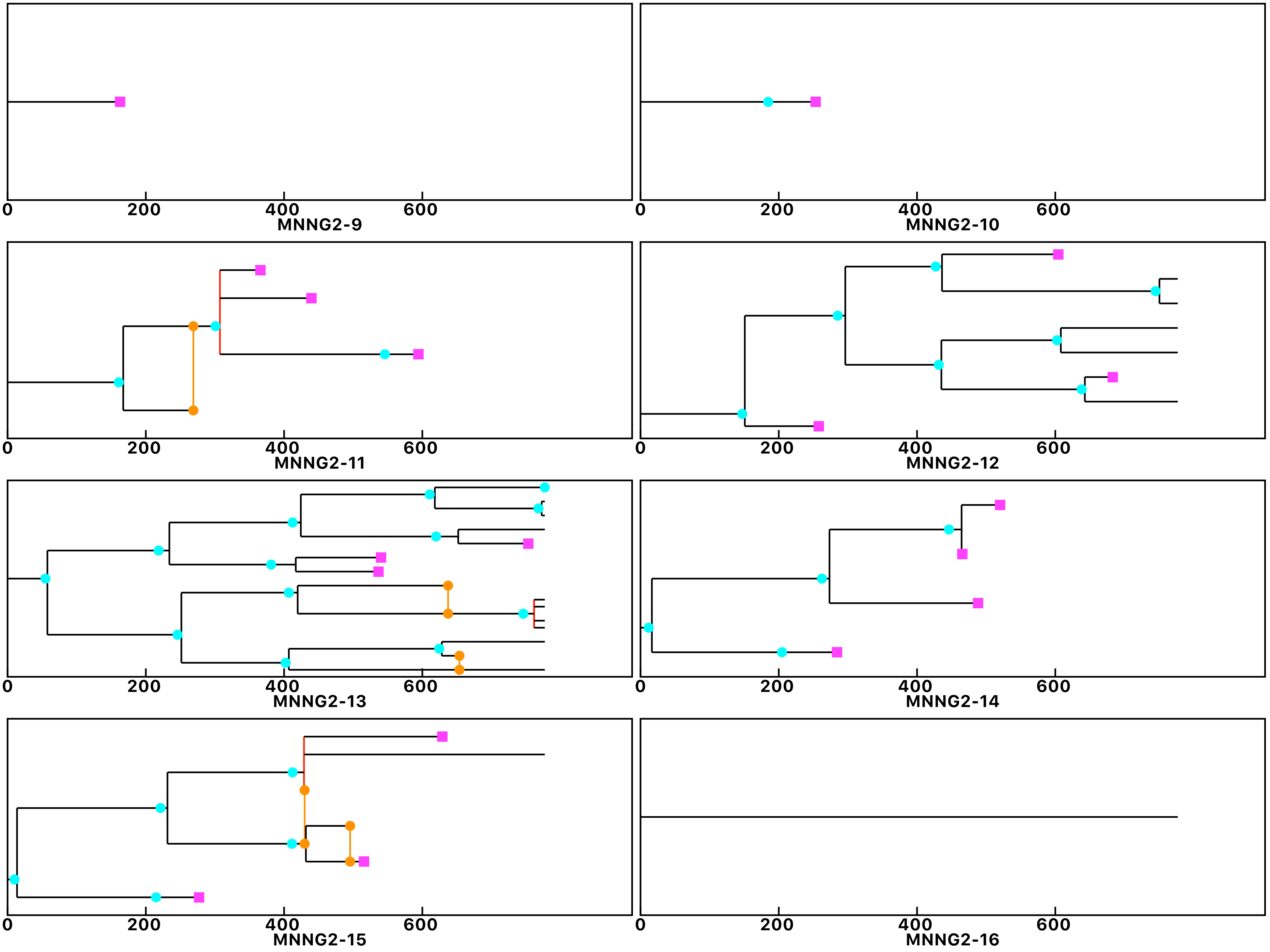

Analysis: MNNG, Treat.: MNNG2, Cell: HeLa

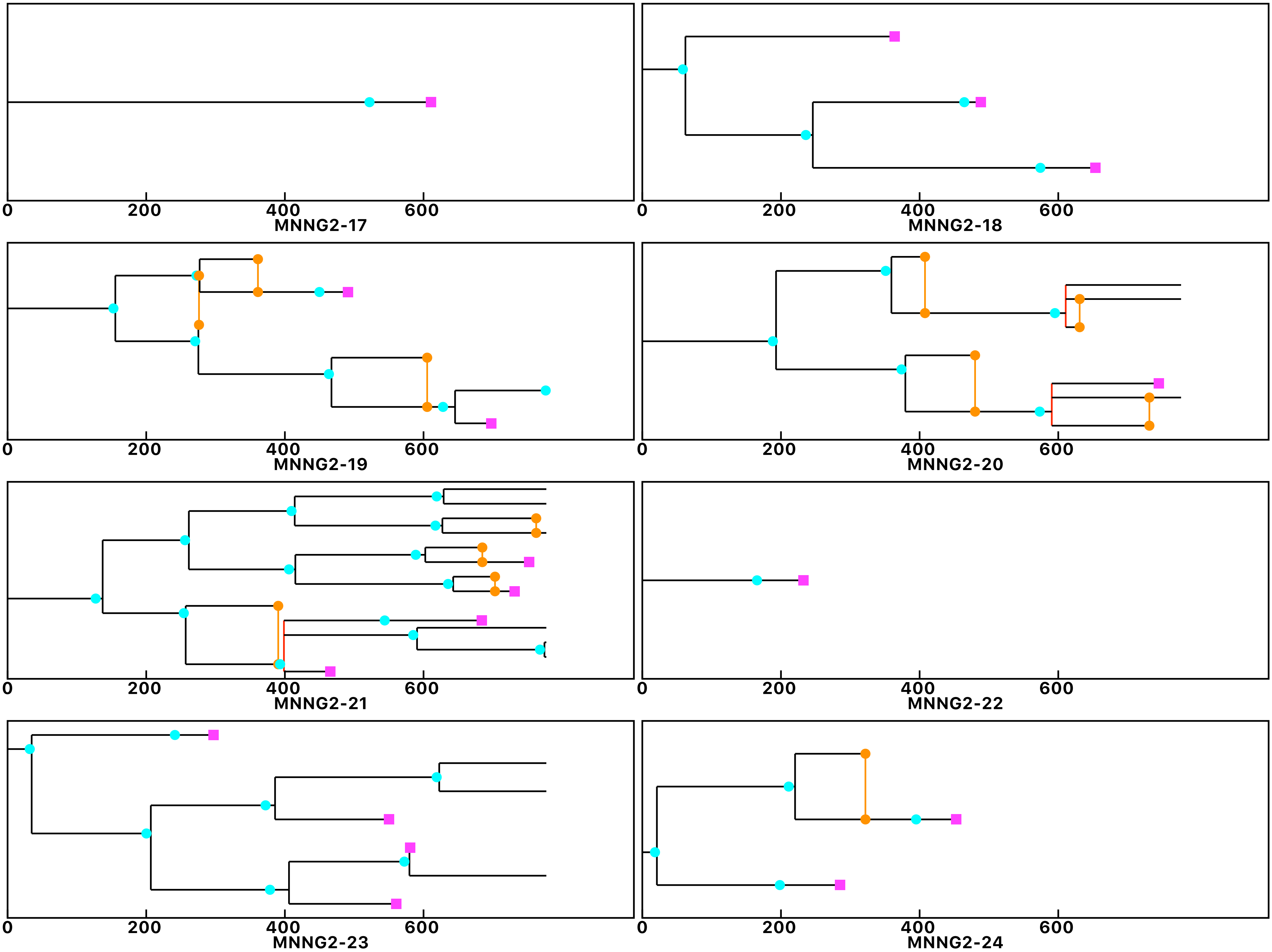

Analysis: MNNG, Treat.: MNNG2, Cell: HeLa

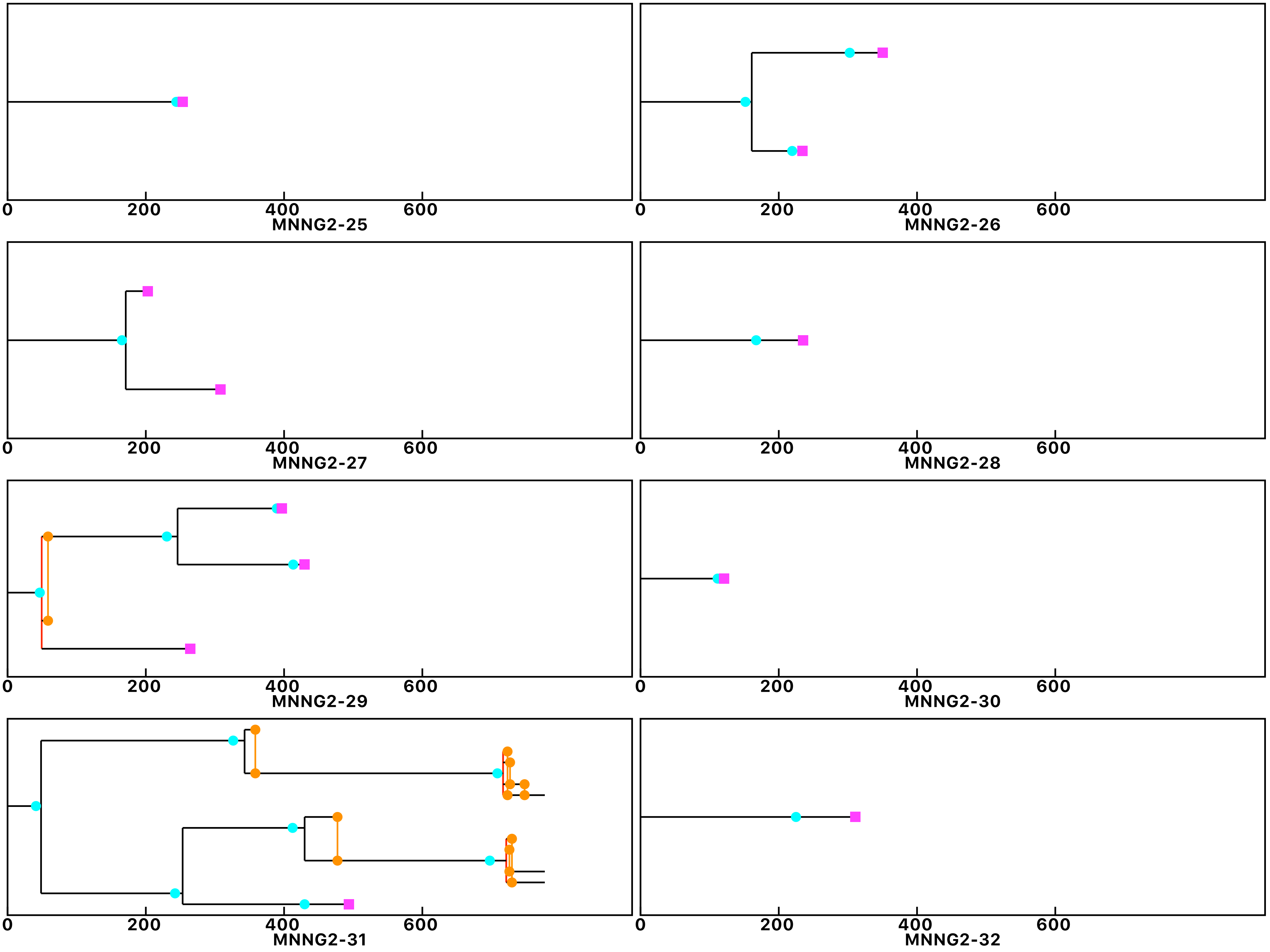

Analysis: MNNG, Treat.: MNNG2, Cell: HeLa

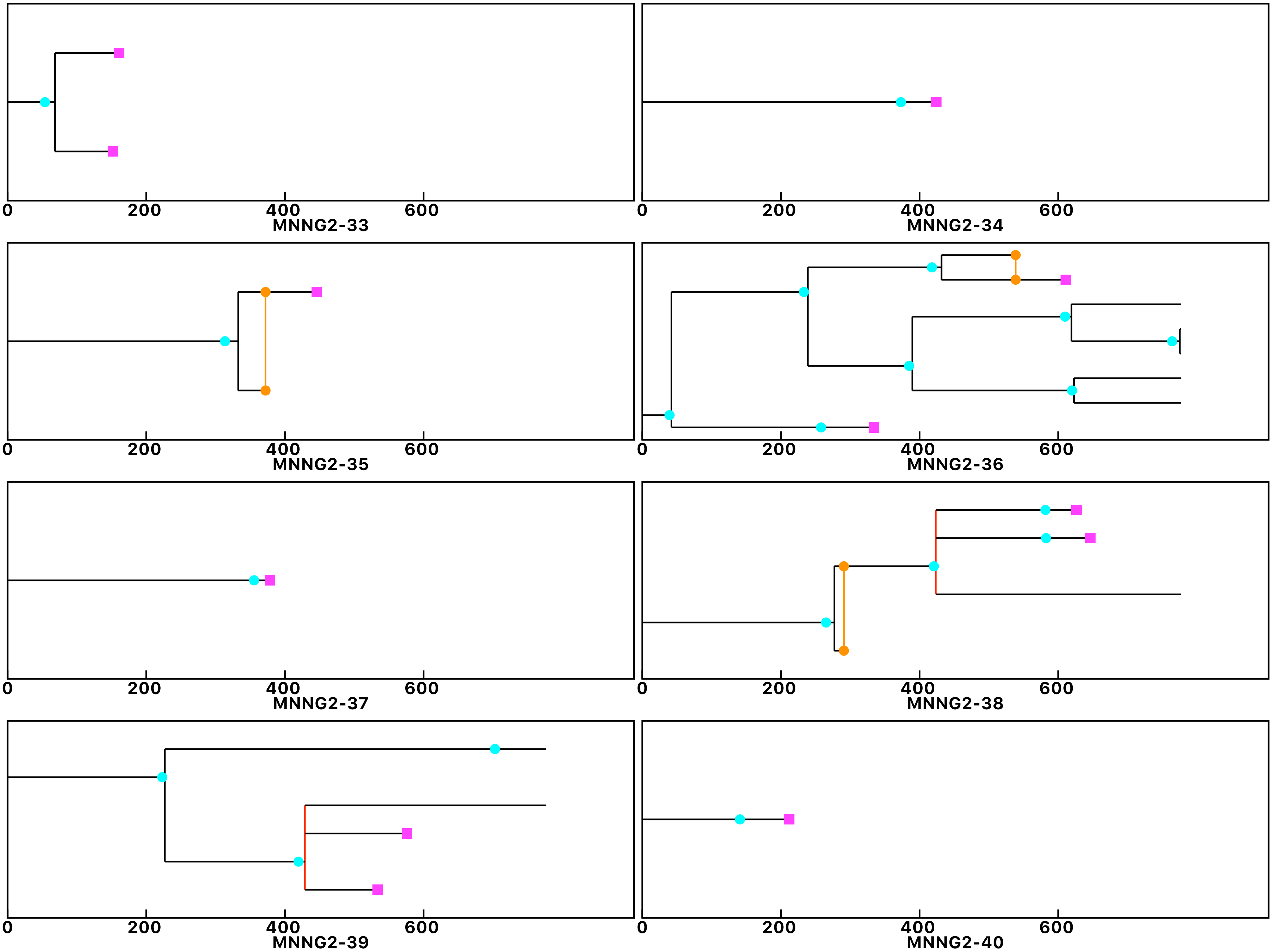

Analysis: MNNG, Treat.: MNNG2, Cell: HeLa

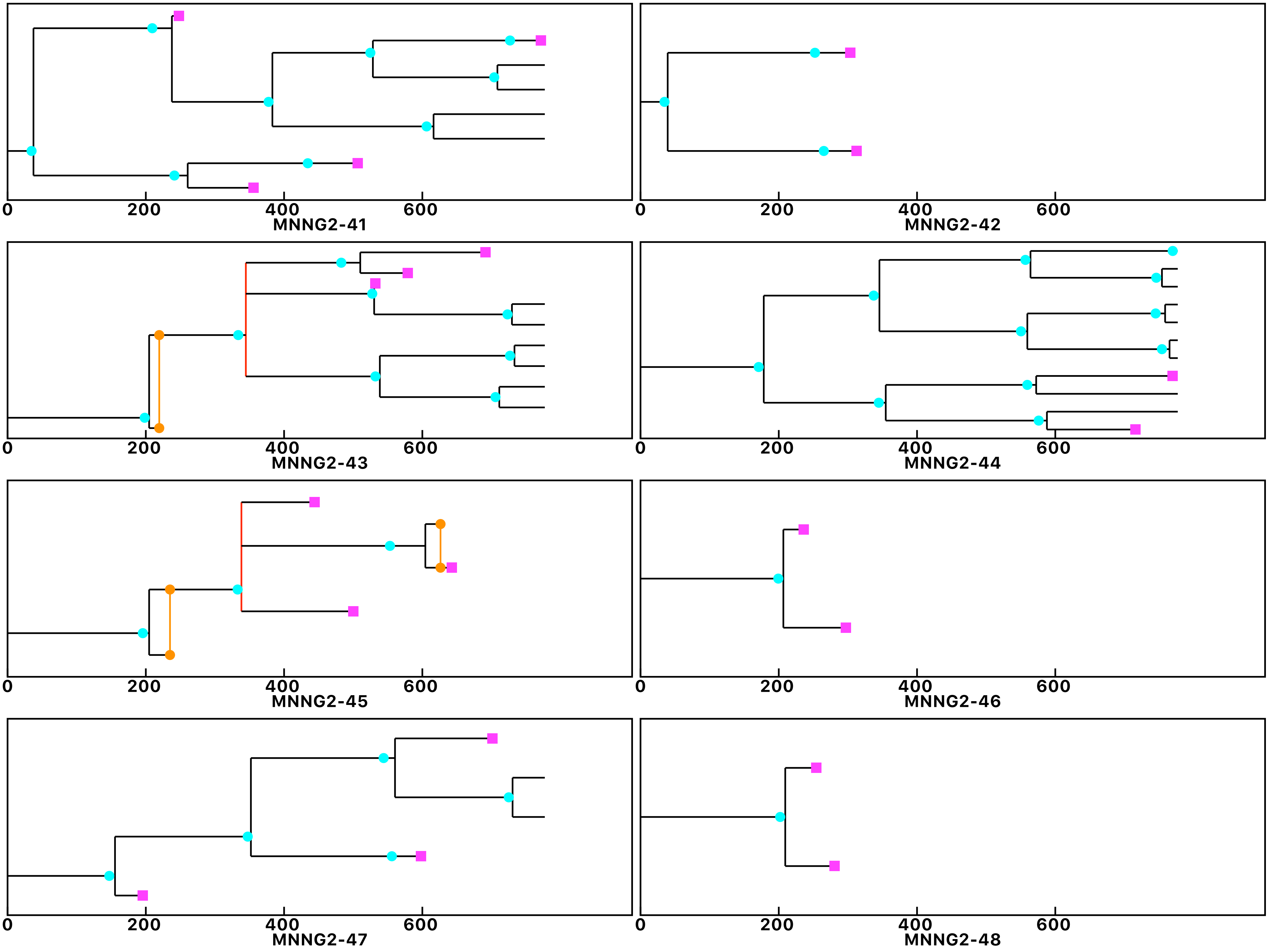

Analysis: MNNG, Treat.: MNNG2, Cell: HeLa

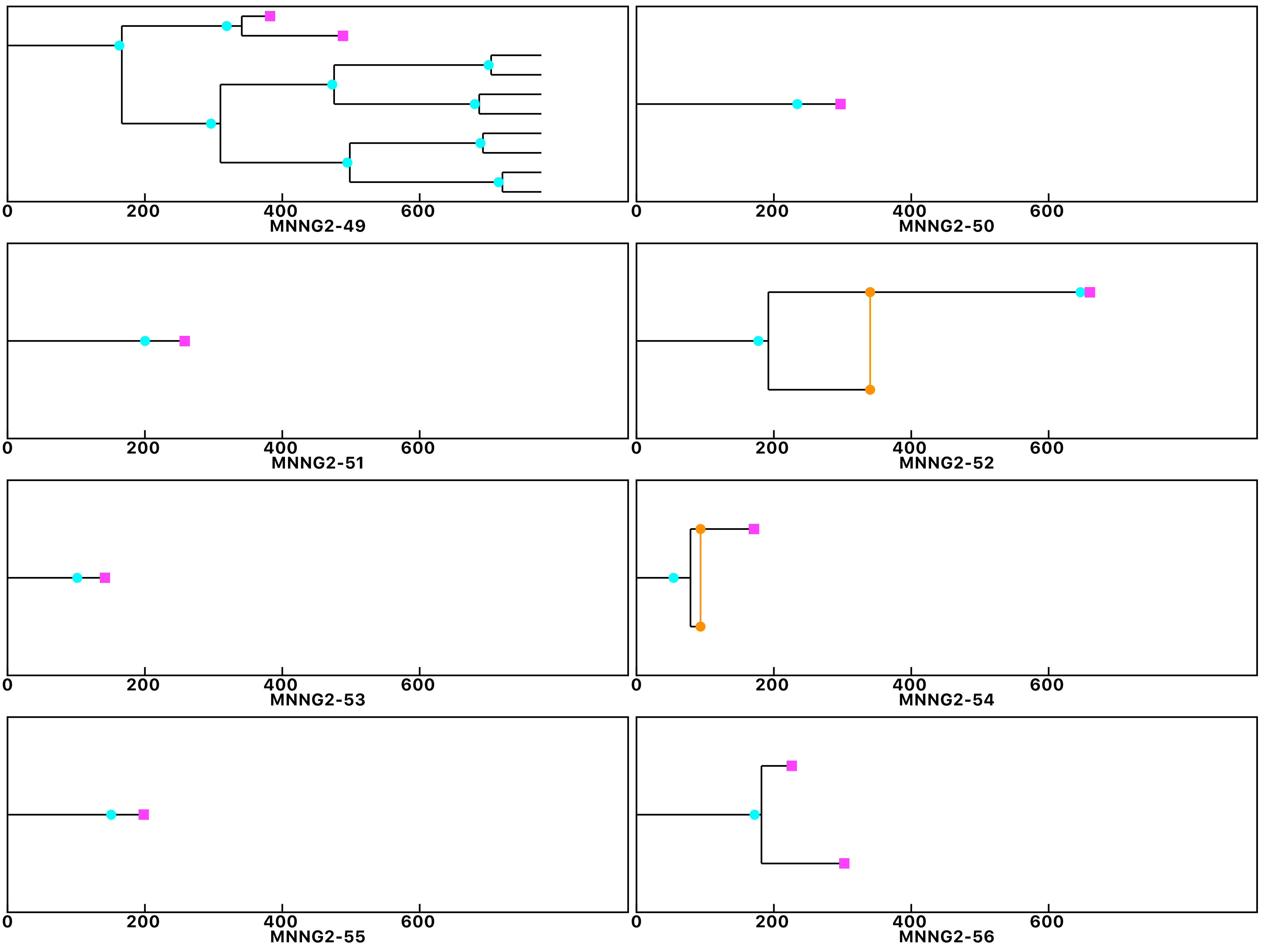

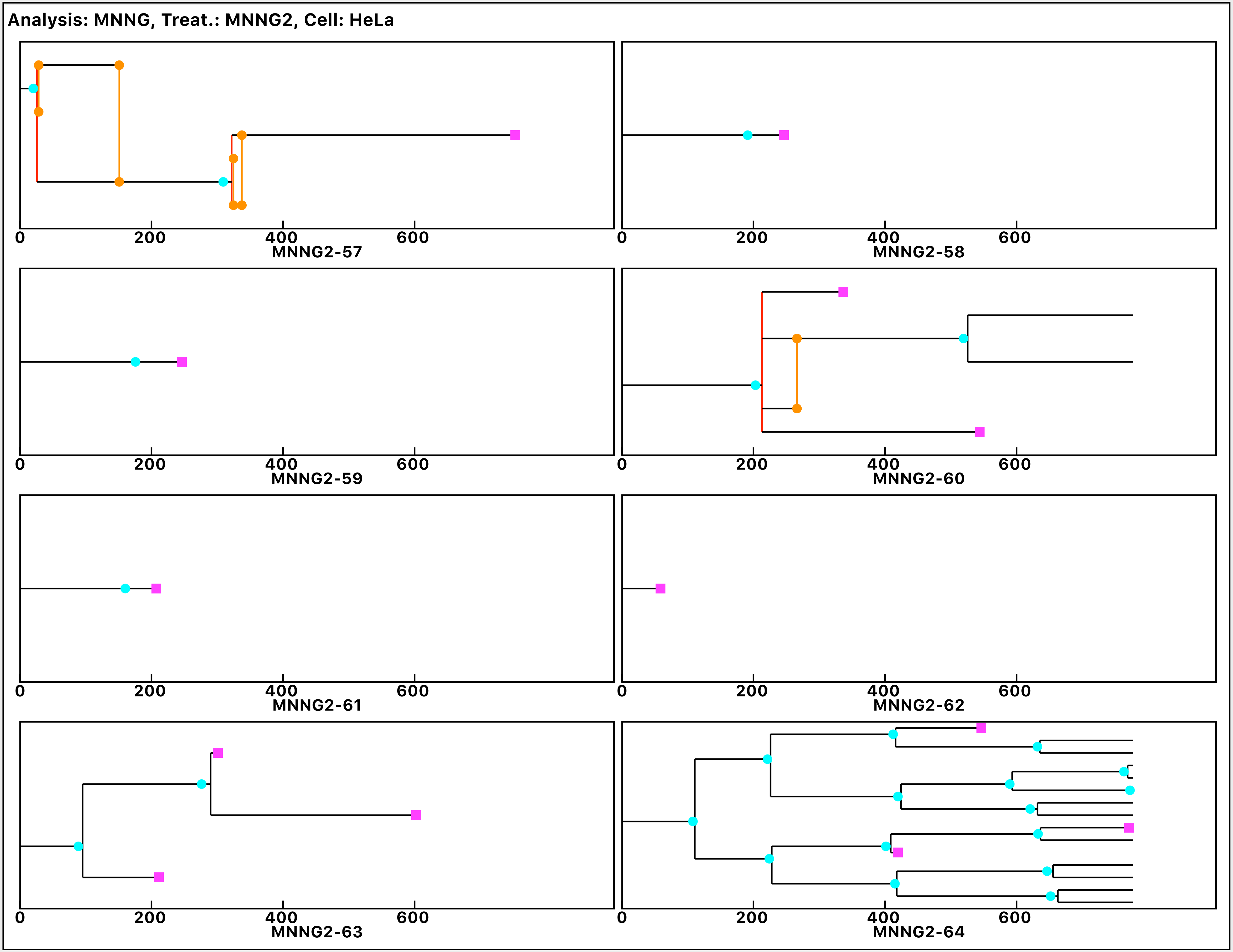

Analysis: MNNG, Treat.: MNNG2, Cell: HeLa
0
200
400
600
MNNG2-57
MNNG2-58
MNNG2-59
MNNG2-60
MNNG2-61
MNNG2-62
MNNG2-63
MNNG2-64

Analysis: MNNG, Treat.: MNNG2, Cell: HeLa

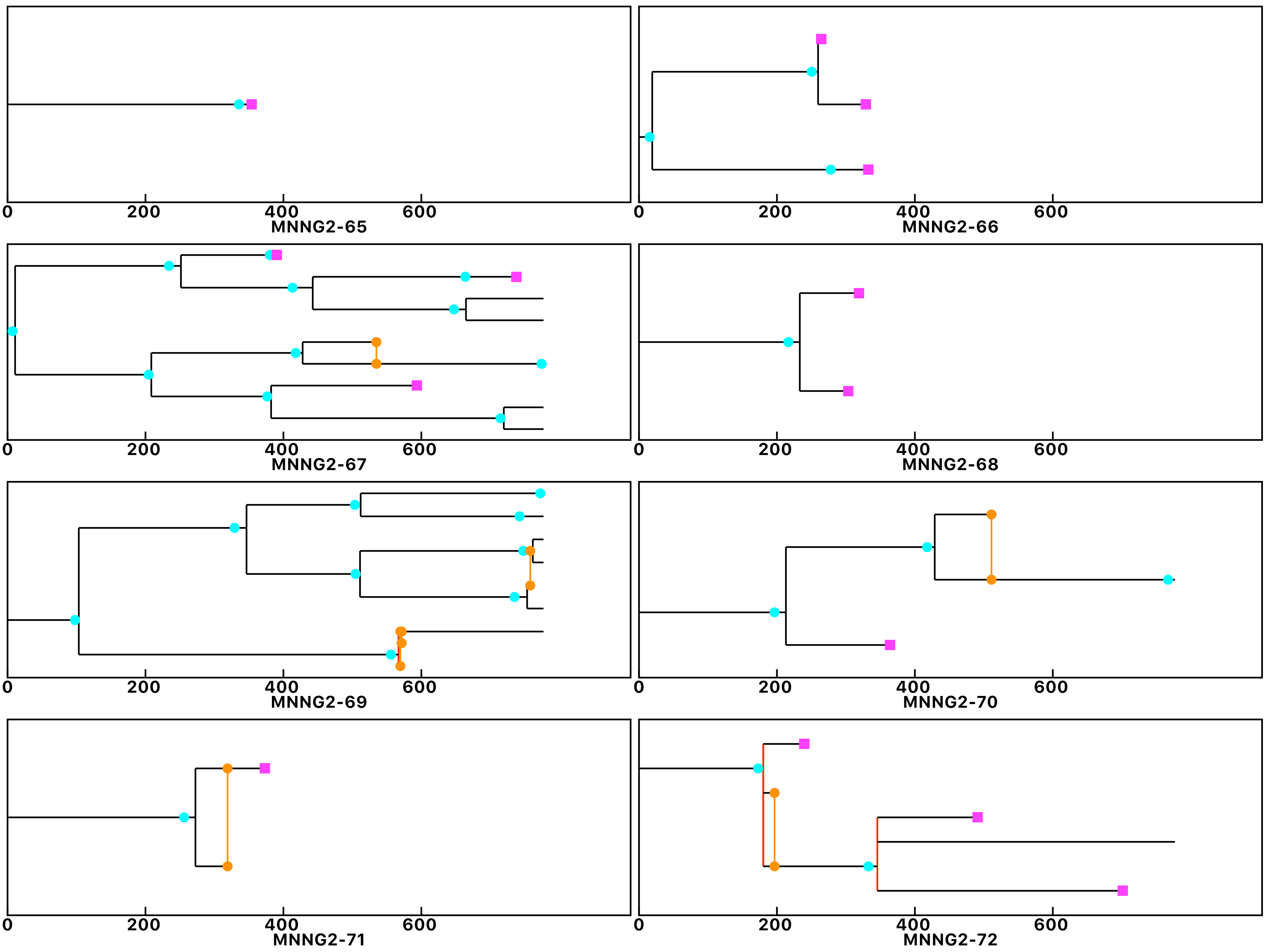

Analysis: MNNG, Treat.: MNNG2, Cell: HeLa

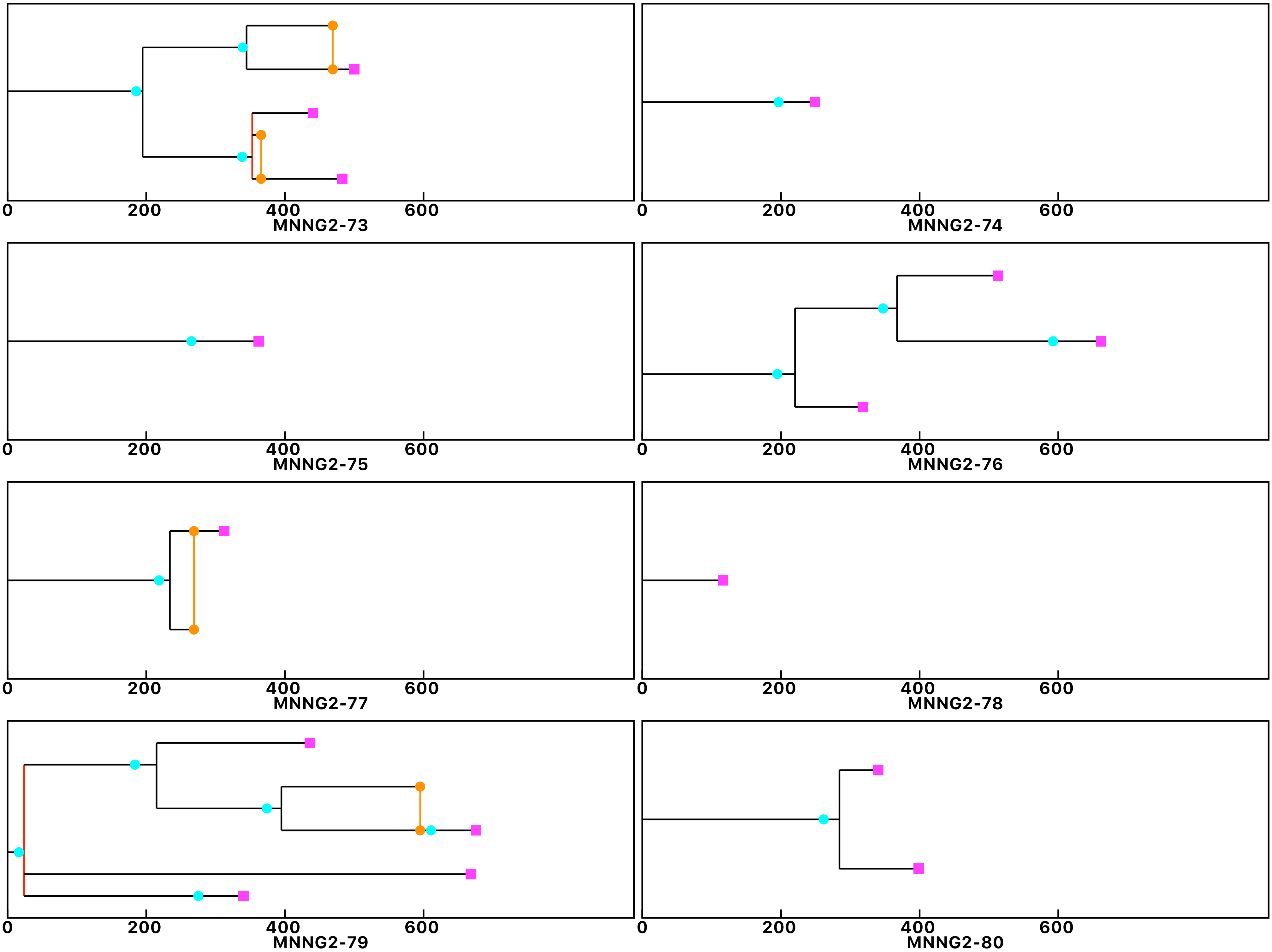

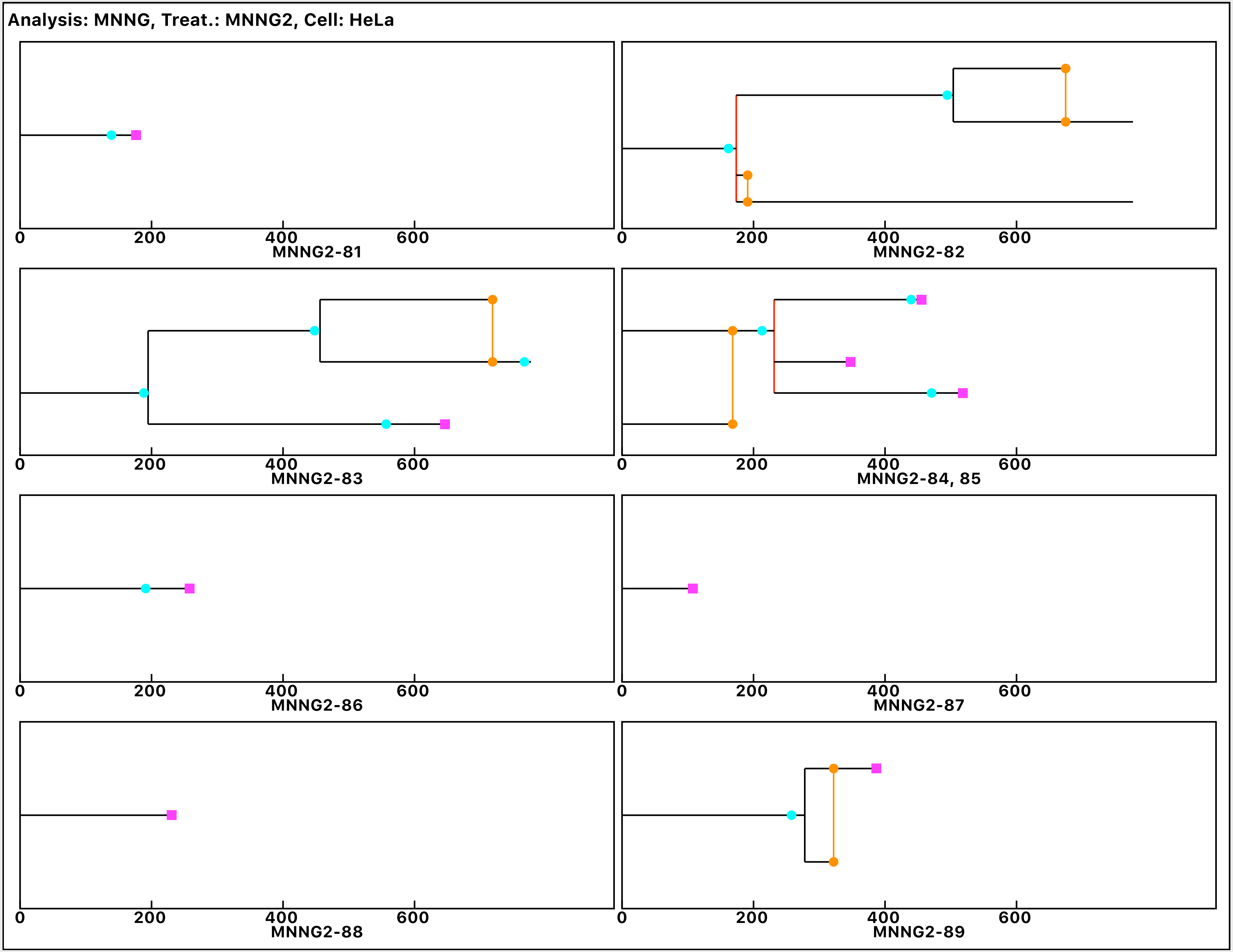
Analysis: MNNG, Treat.: MNNG2, Cell: HeLa
0
200
400
600
MNNG2-81
MNNG2-82
MNNG2-83
MNNG2-84, 85
MNNG2-86
MNNG2-87
MNNG2-88
MNNG2-89

Analysis: MNNG, Treat.: MNNG2, Cell: HeLa

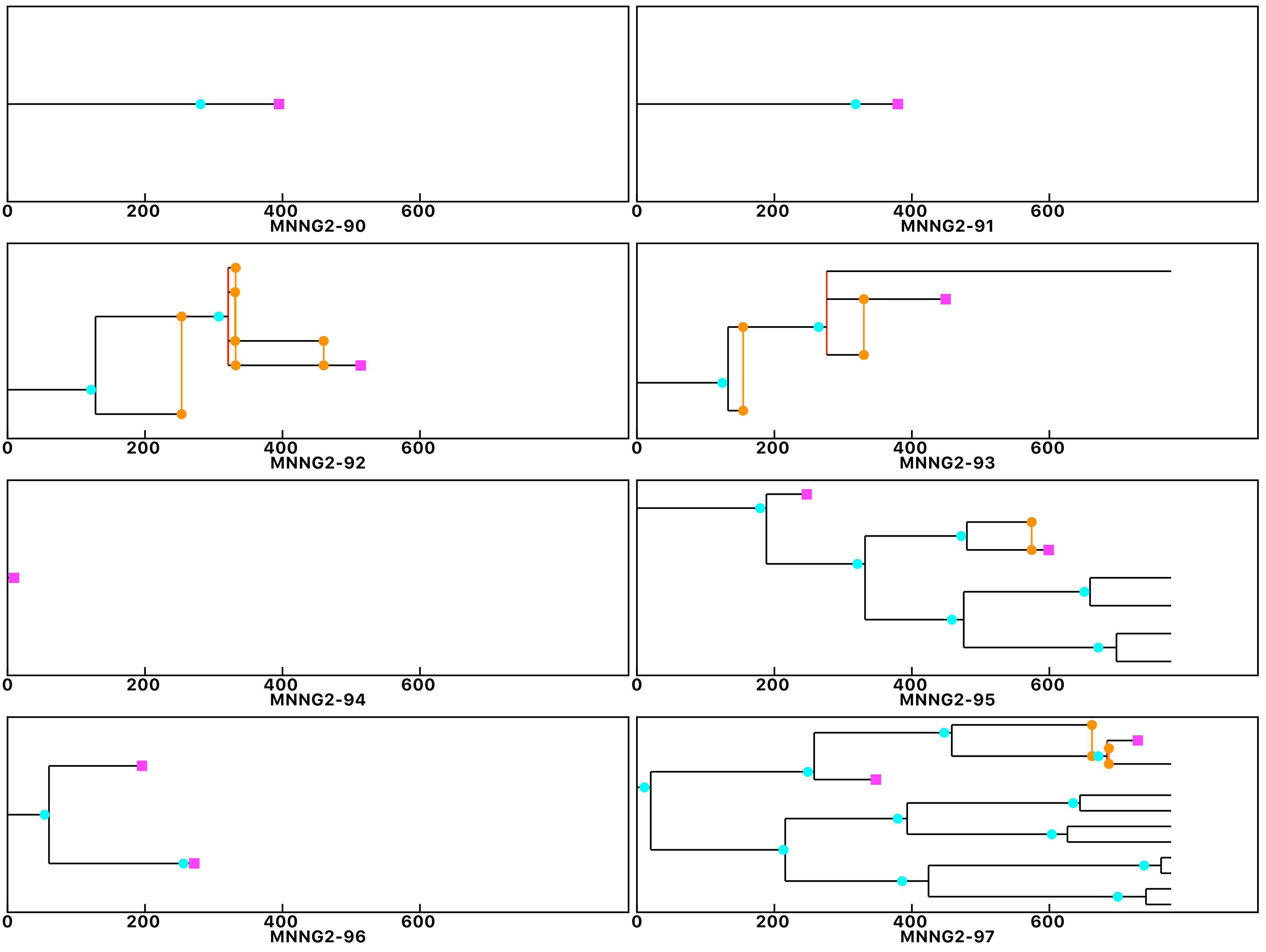

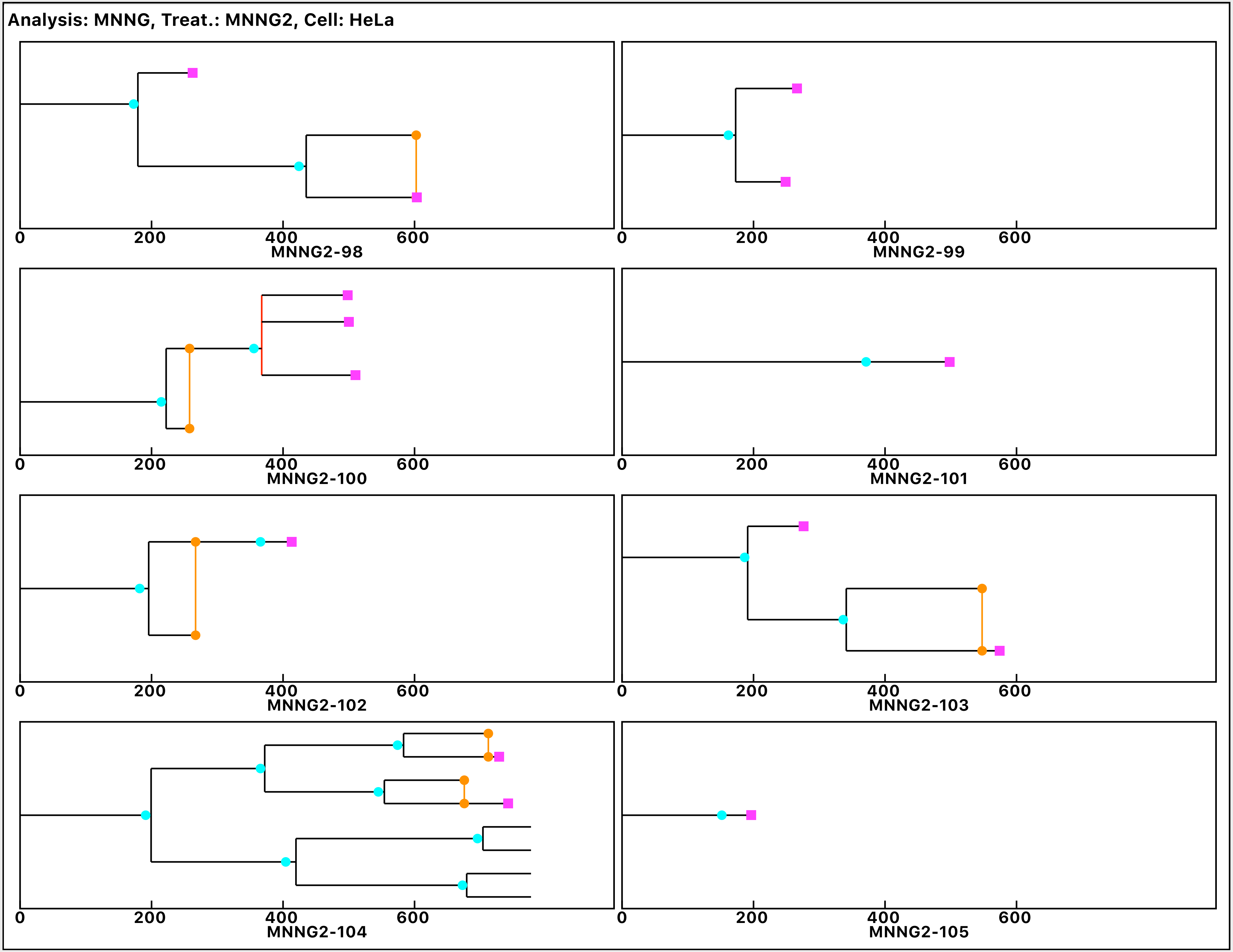

Analysis: MNNG, Treat.: MNNG2, Cell: HeLa
0
200
400
600
MNNG2-98
MNNG2-99
MNNG2-100
MNNG2-101
MNNG2-102
MNNG2-103
MNNG2-104
MNNG2-105

Analysis: MNNG, Treat.: MNNG2, Cell: HeLa

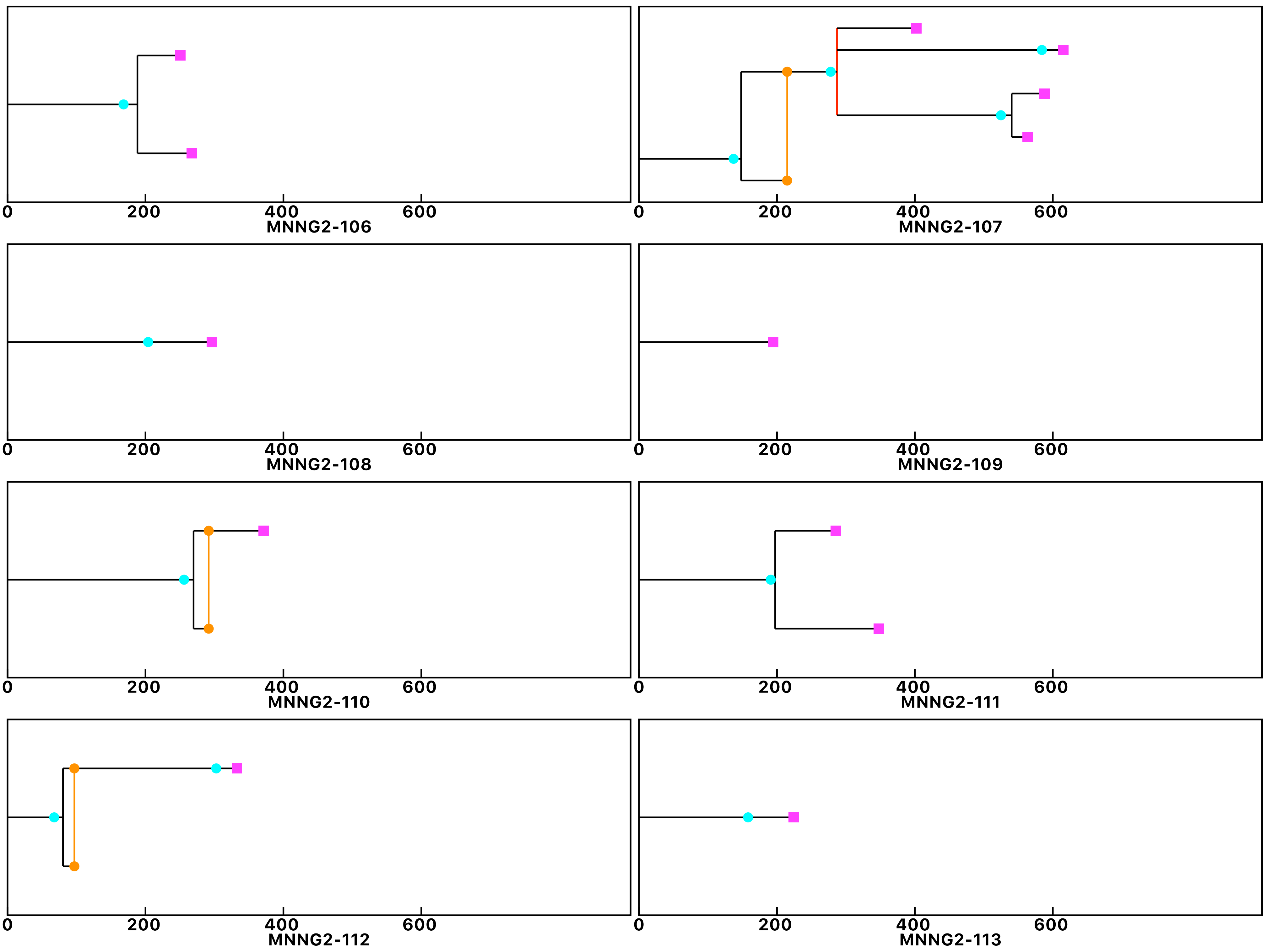

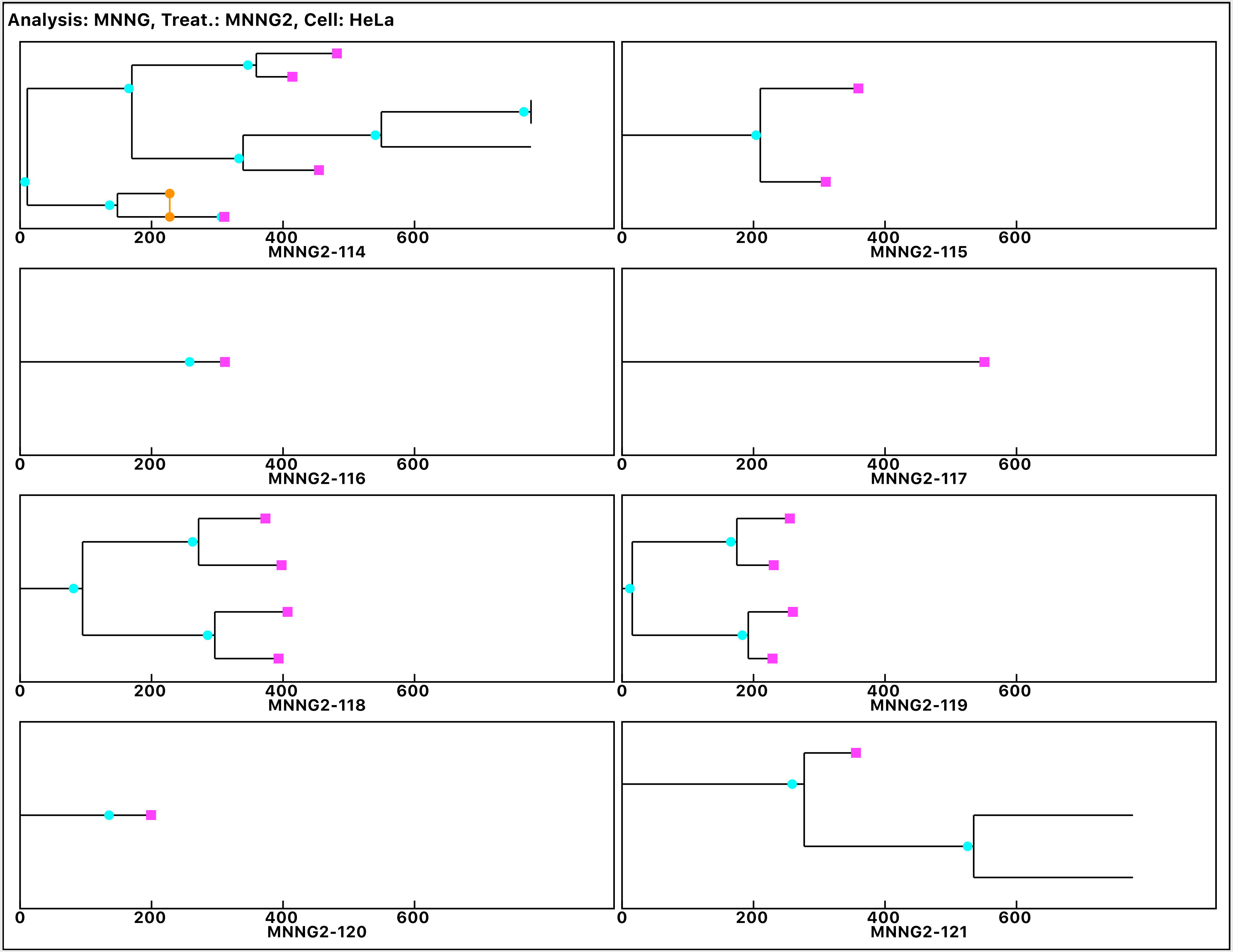
Analysis: MNNG, Treat.: MNNG2, Cell: HeLa
0
200
400
600
MNNG2-114
MNNG2-115
MNNG2-116
MNNG2-117
MNNG2-118
MNNG2-119
MNNG2-120
MNNG2-121

Analysis: MNNG, Treat.: MNNG2, Cell: HeLa

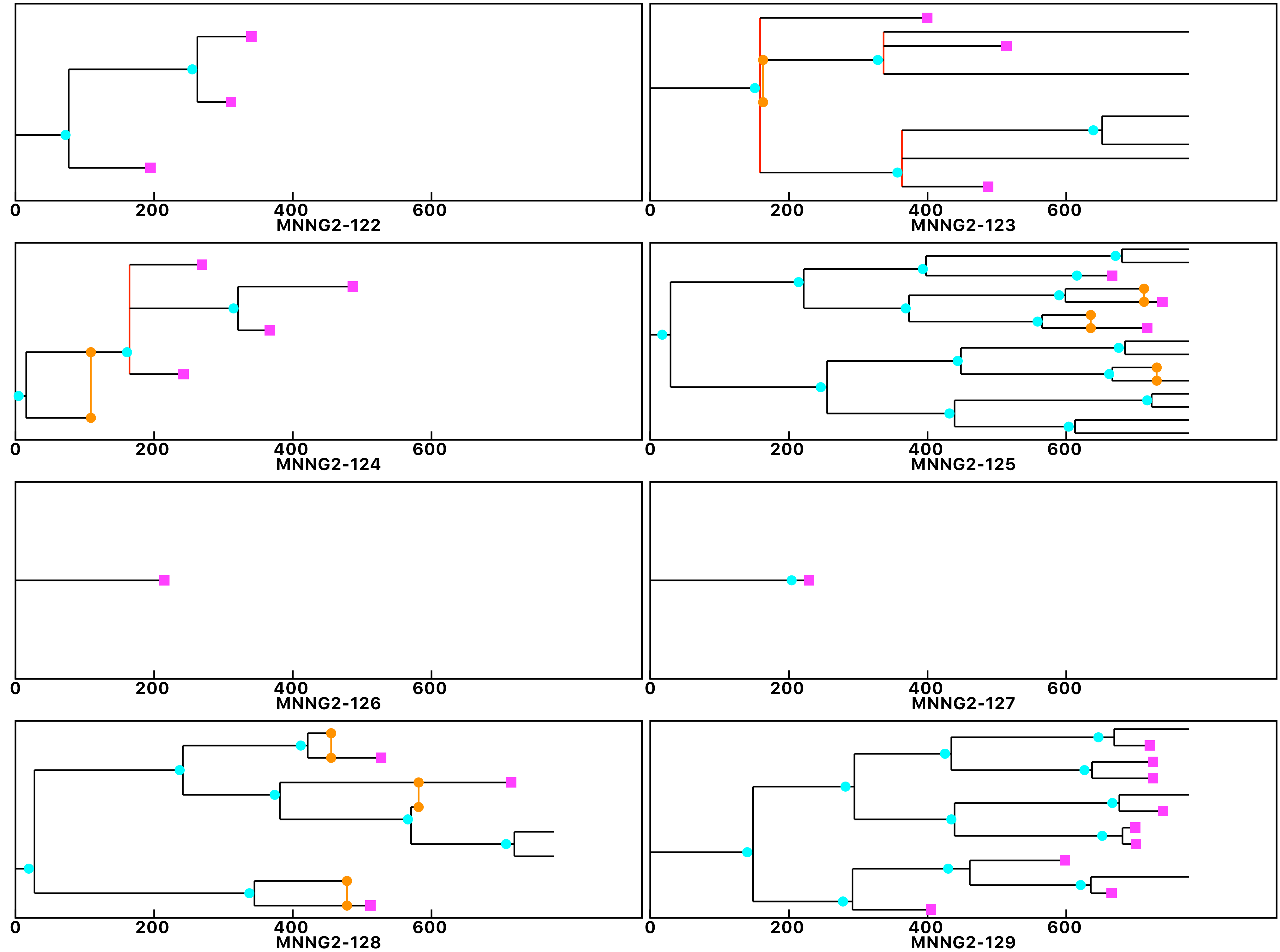

Analysis: MNNG, Treat.: MNNG2, Cell: HeLa

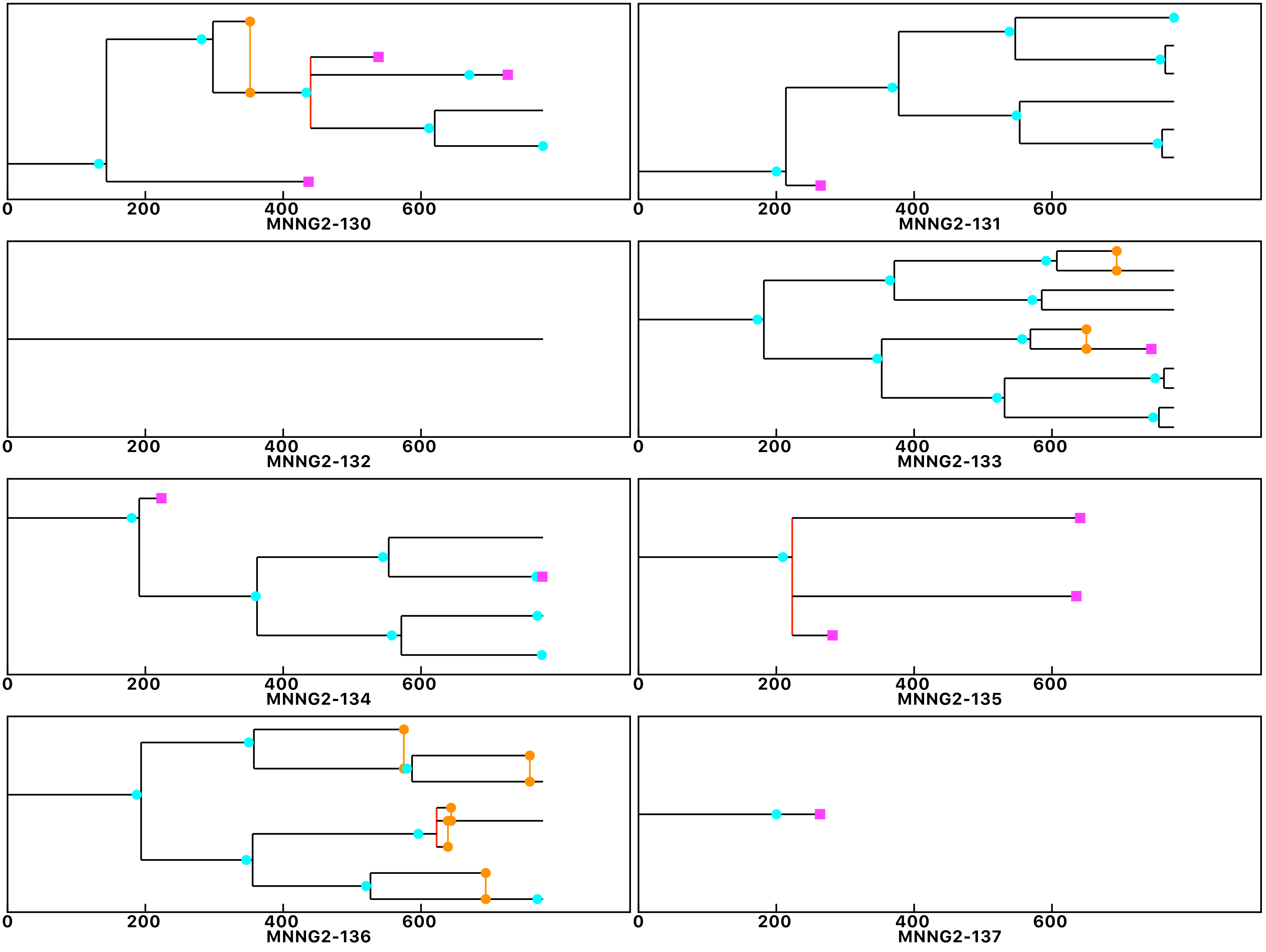

Analysis: MNNG, Treat.: MNNG2, Cell: HeLa

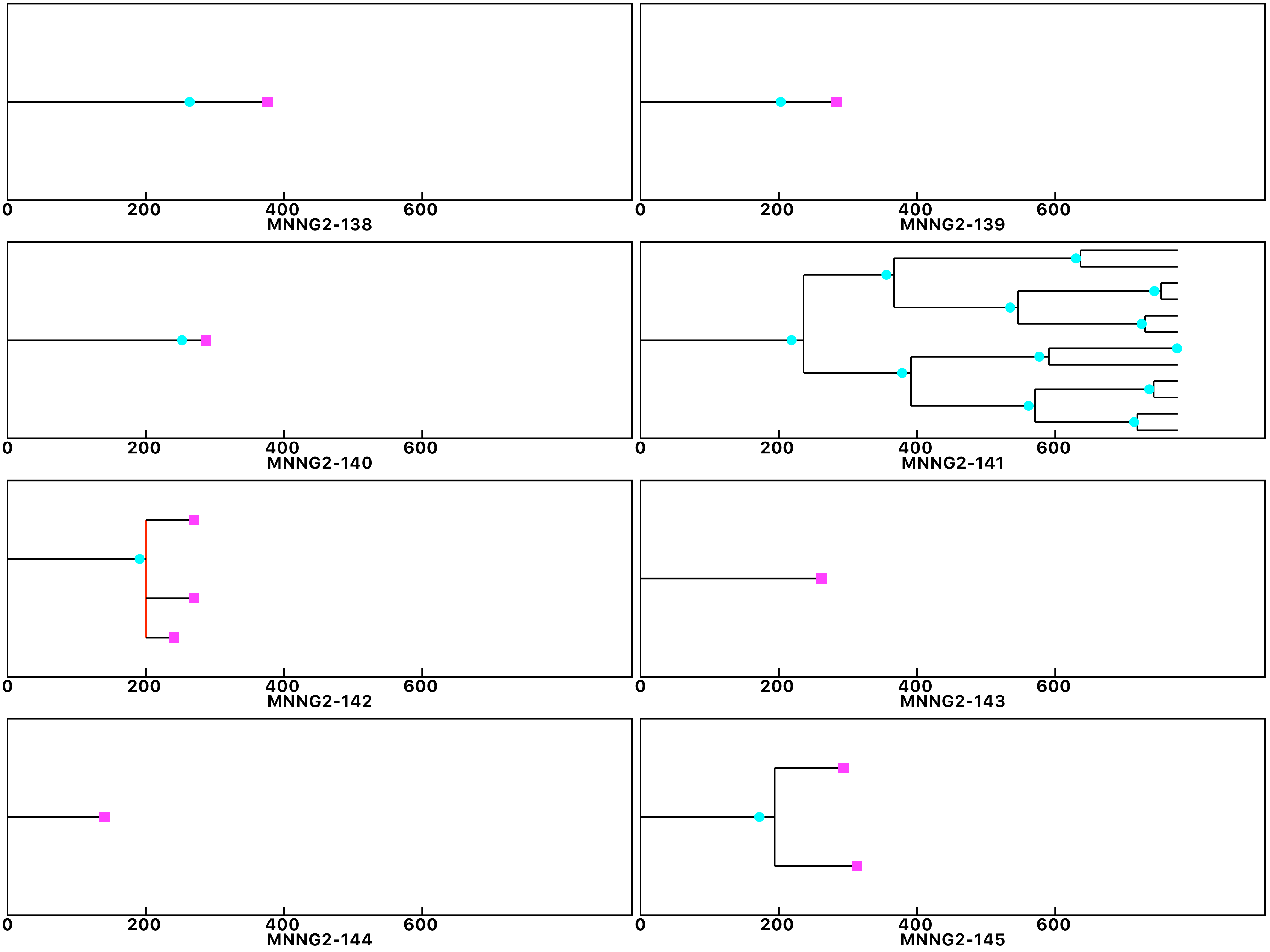

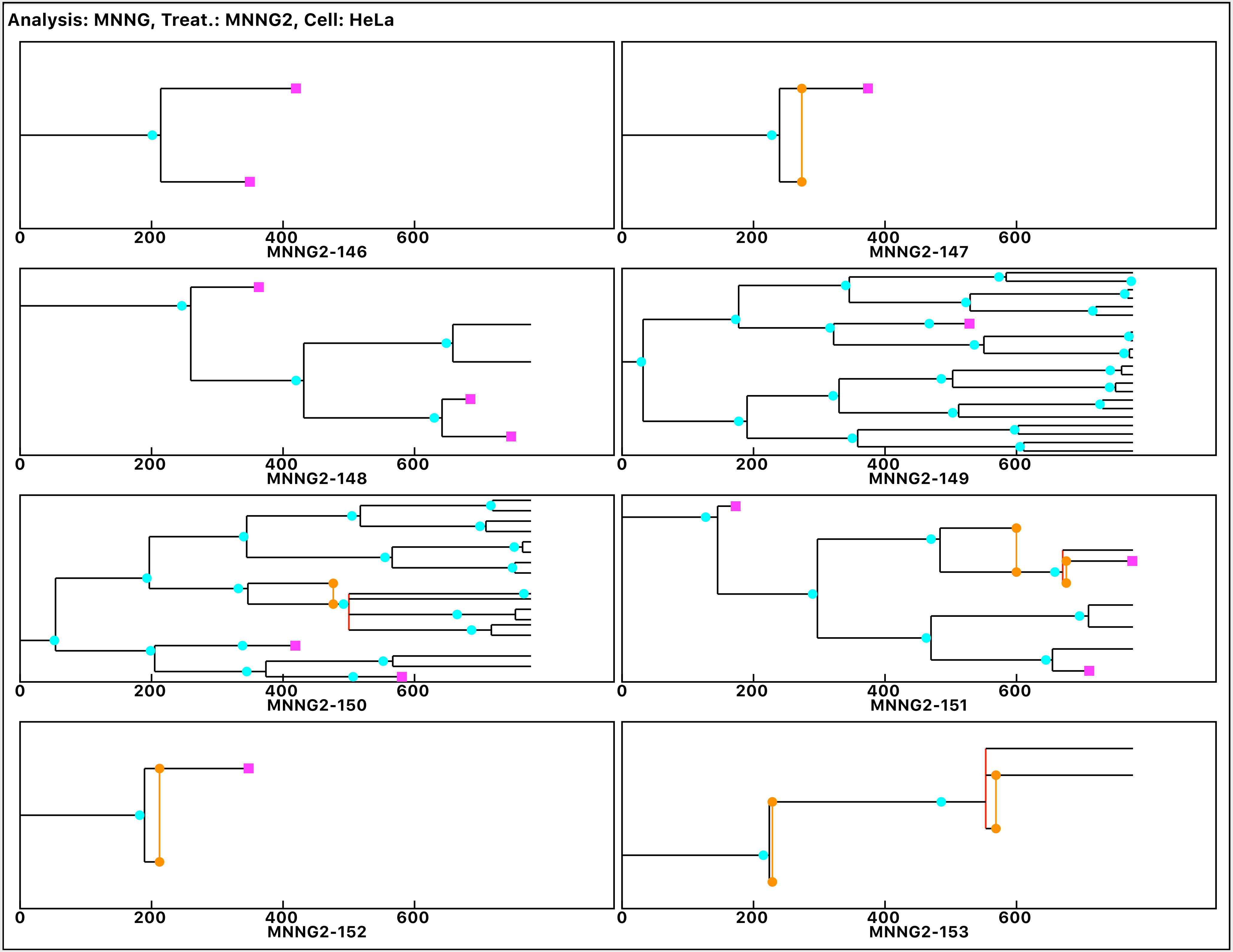
Analysis: MNNG, Treat.: MNNG2, Cell: HeLa
0
200
400
600
MNNG2-146
MNNG2-147
MNNG2-148
MNNG2-149
MNNG2-150
MNNG2-151
MNNG2-152
MNNG2-153

Analysis: MNNG, Treat.: MNNG2, Cell: HeLa

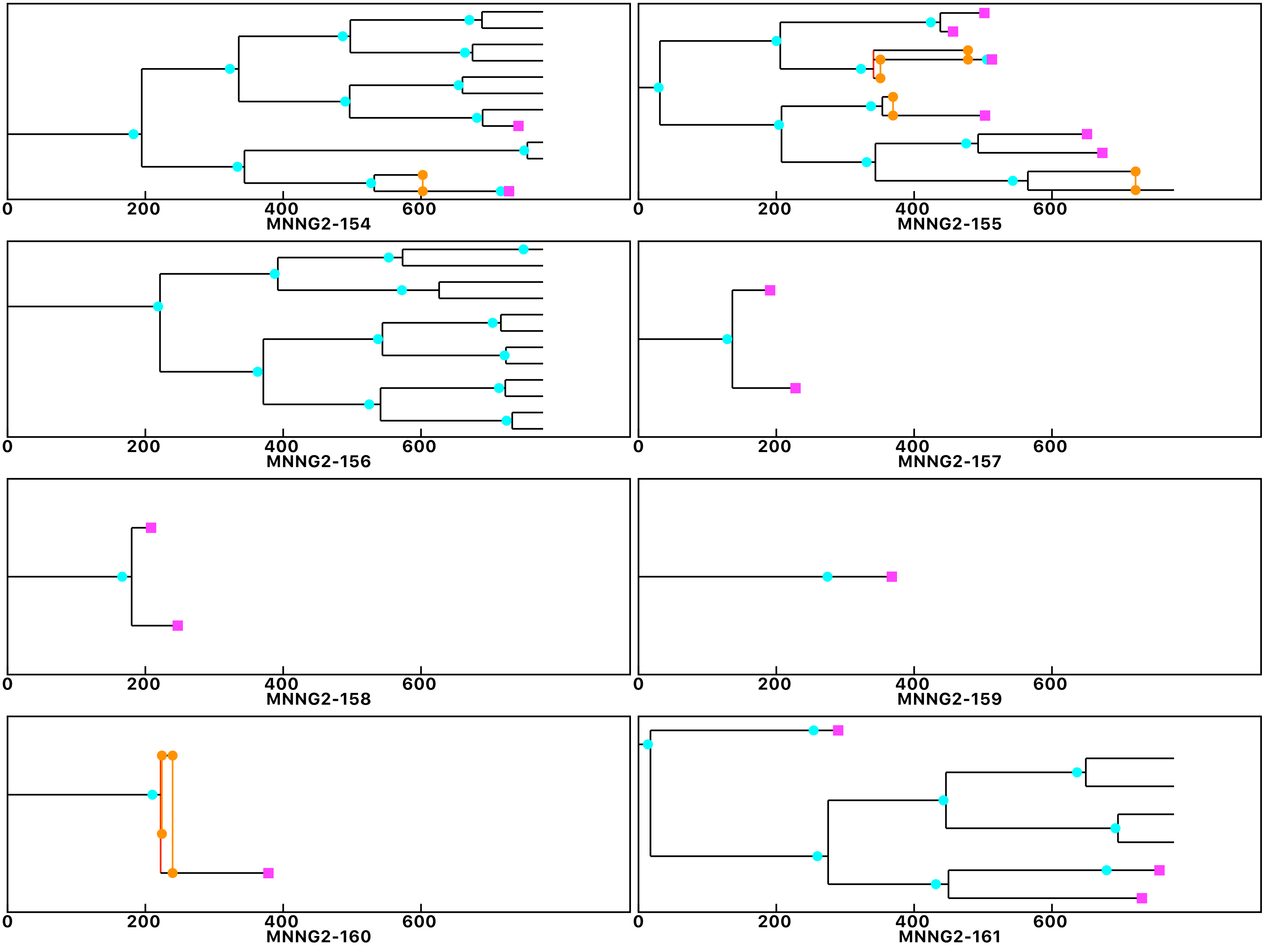

Analysis: MNNG, Treat.: MNNG2, Cell: HeLa

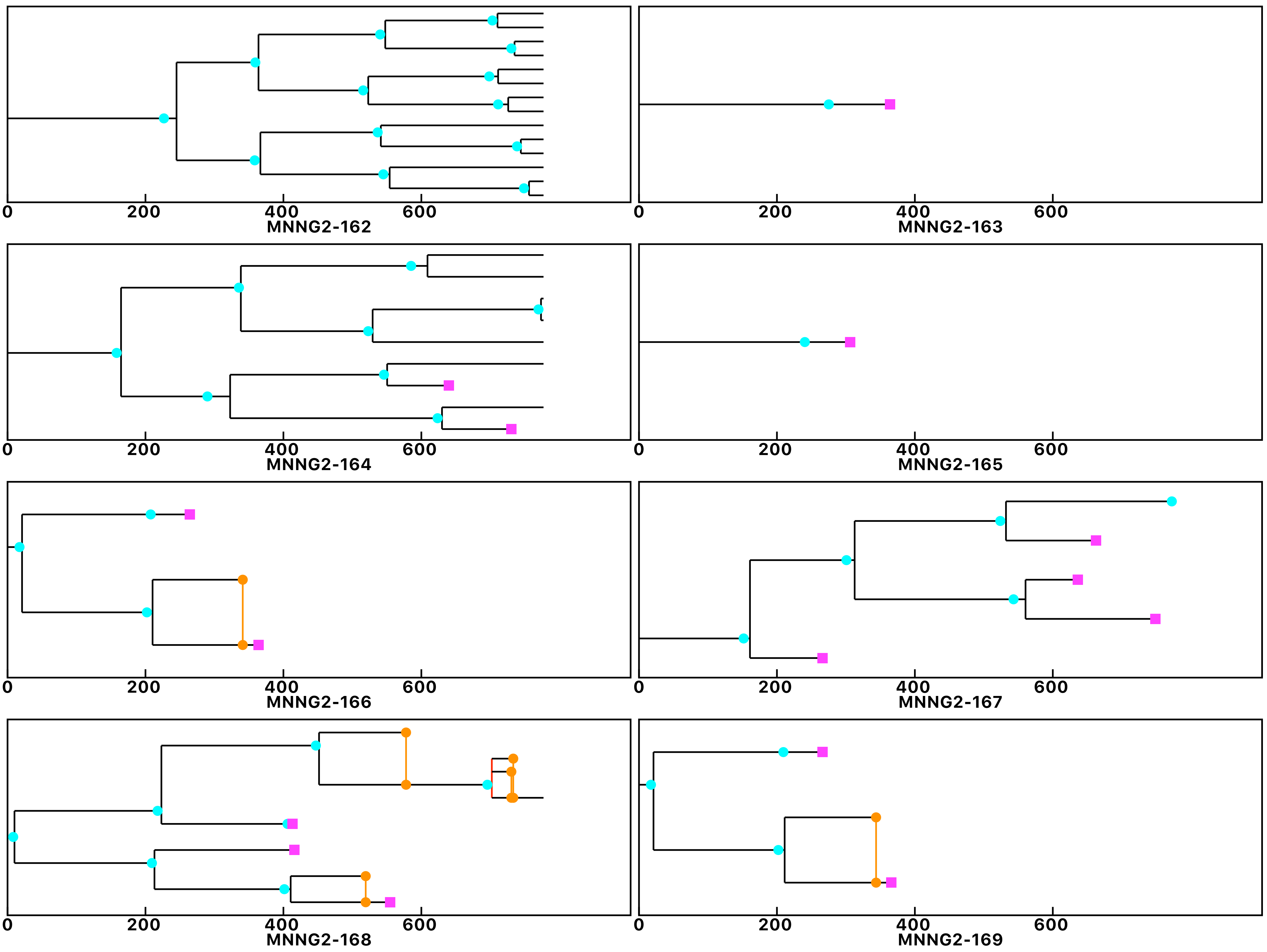

Analysis: MNNG, Treat.: MNNG2, Cell: HeLa

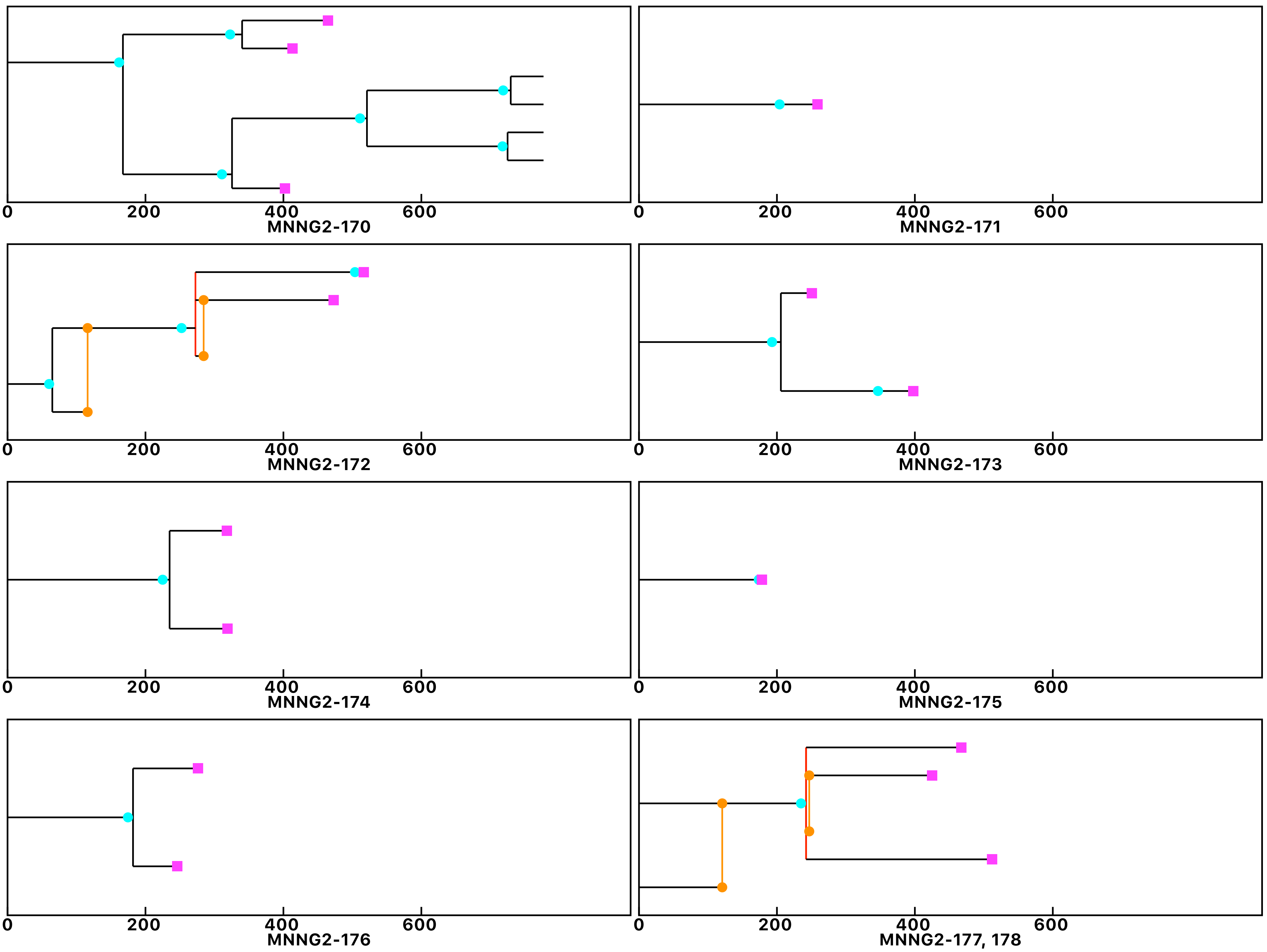

Analysis: MNNG, Treat.: MNNG2, Cell: HeLa

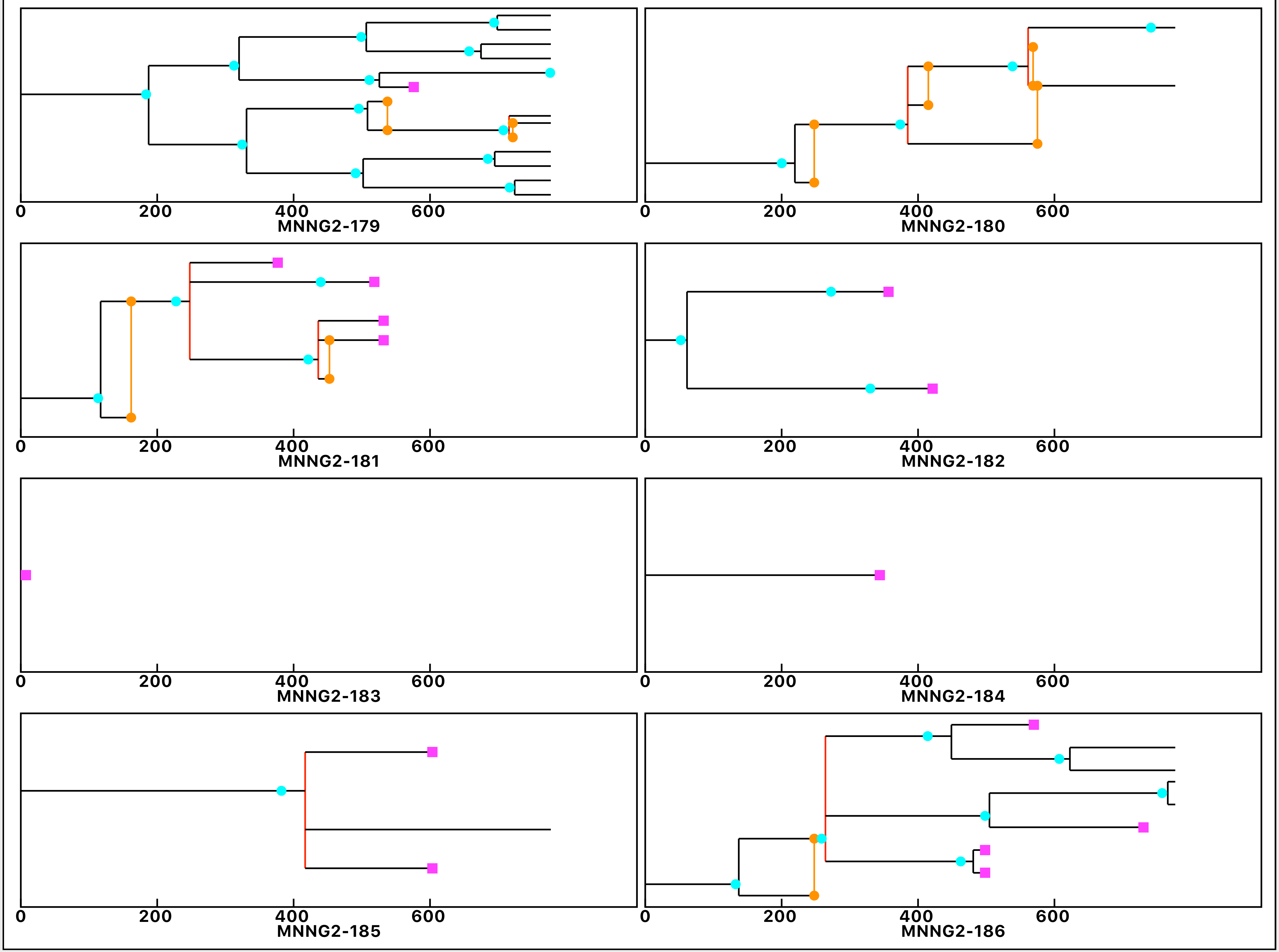

Analysis: MNNG, Treat.: MNNG2, Cell: HeLa

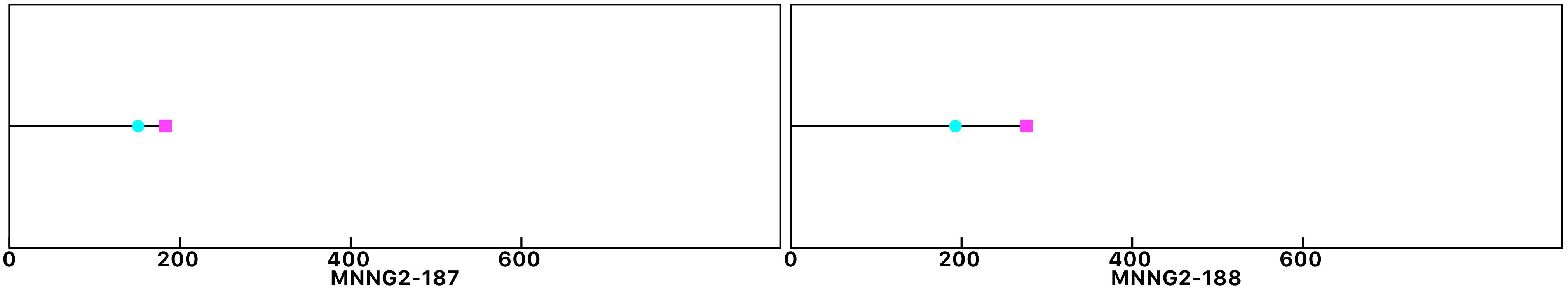

Supplement: S3 Fig — Cell lineage maps (MNNG-2μM; MNNG2) are shown. Light blue circle: mitosis, orange circle: cell fusion, pink square: cell death, blue square: incomplete cell division, black vertical line: bipolar cell division, red vertical line: multipolar cell division, and orange vertical line: cell fusion. (PDF) [file pone.0214512.s003.pdf]
