# Supplementary material for: Dose-dependent spatiotemporal responses of mammalian cells to an alkylating agent
Source: PLoS One. 2019 Mar 29;14(3):e0214512. doi: 10.1371/journal.pone.0214512 (PMC6440626; doi:10.1371/journal.pone.0214512)

Supplementary Figure S4

Analysis: MNNG, Treat.: MNNG5, Cell: HeLa

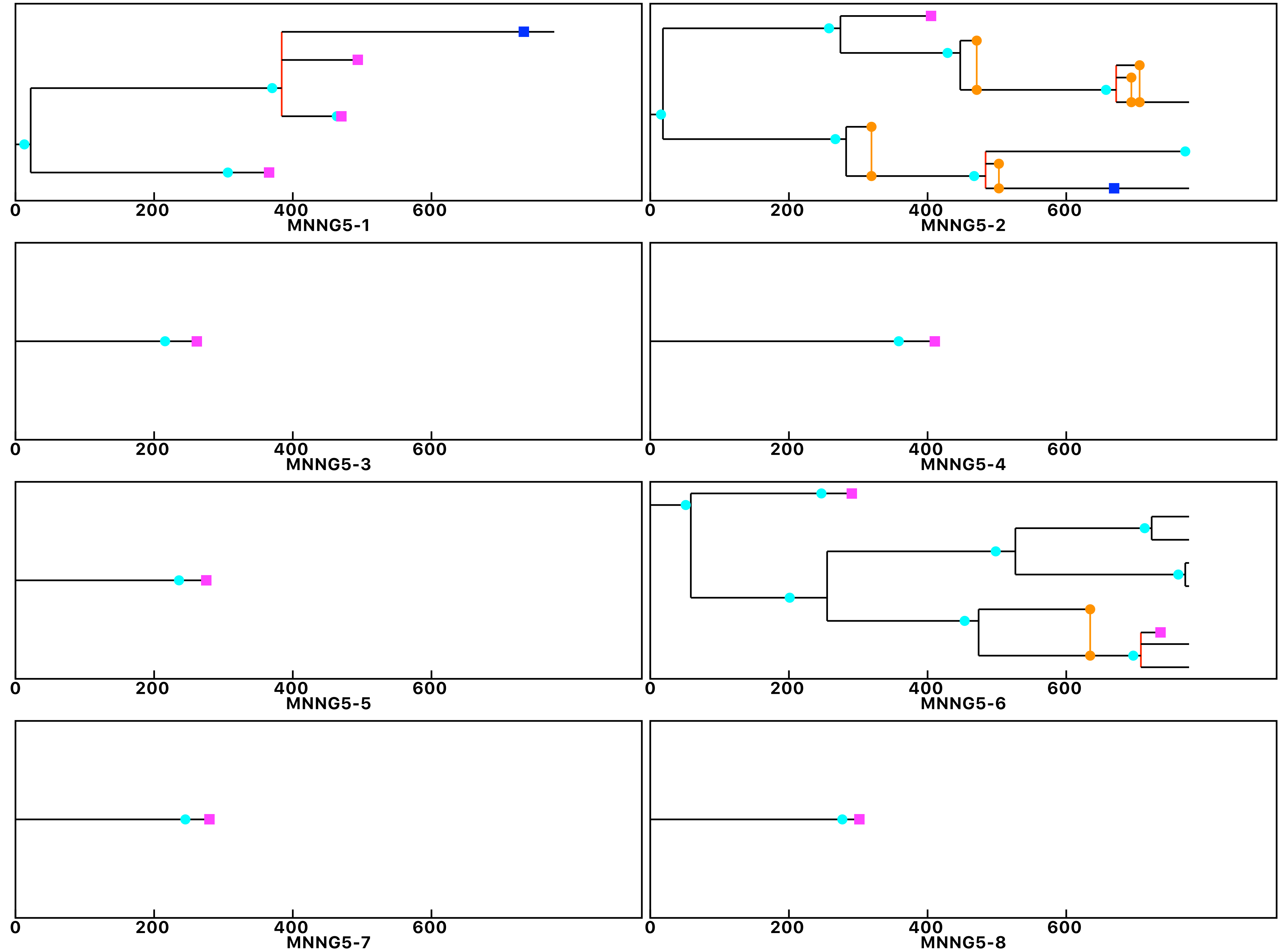

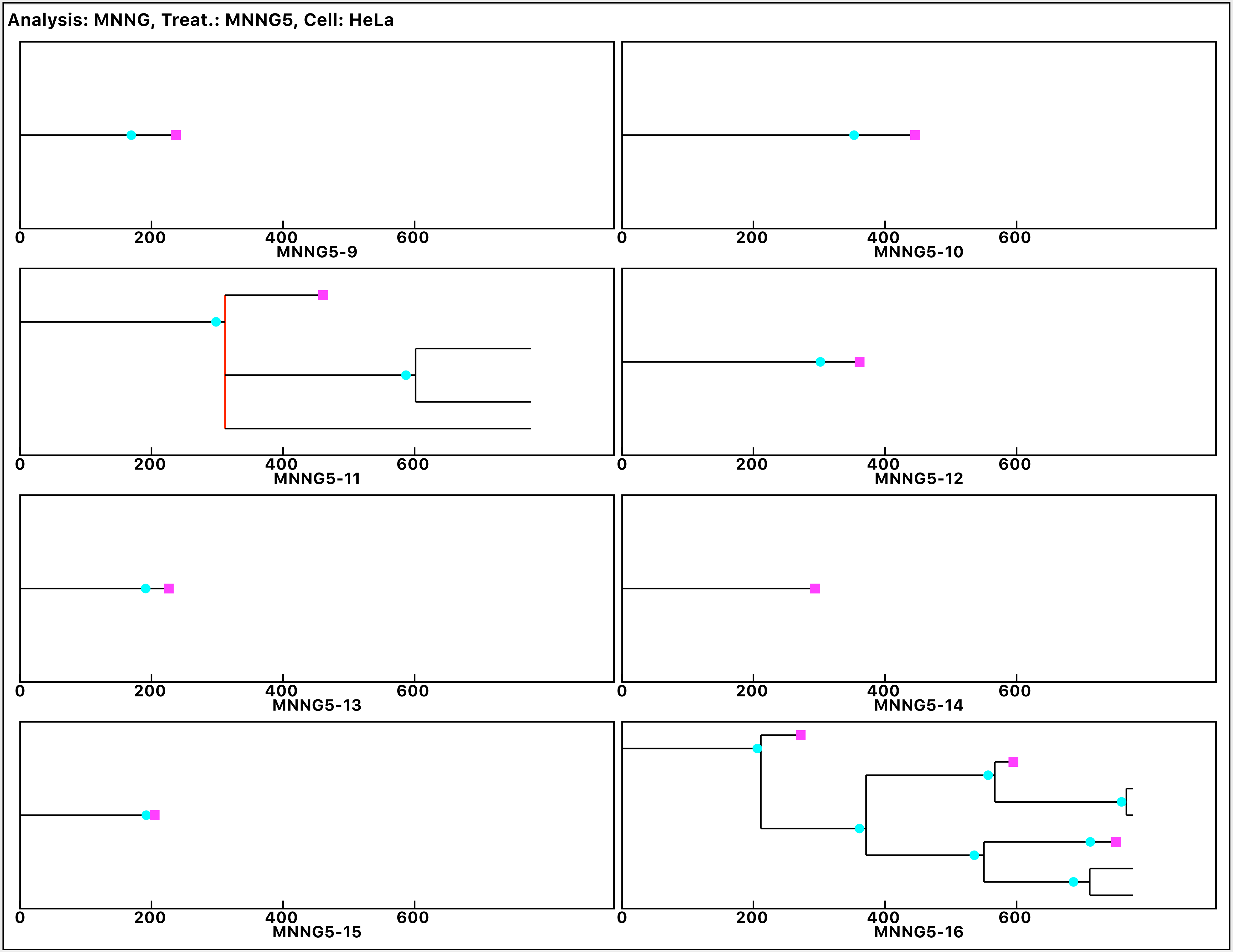
Analysis: MNNG, Treat.: MNNG5, Cell: HeLa
0
200
400
600
MNNG5-9
MNNG5-10
MNNG5-11
MNNG5-12
MNNG5-13
MNNG5-14
MNNG5-15
MNNG5-16

Analysis: MNNG, Treat.: MNNG5, Cell: HeLa

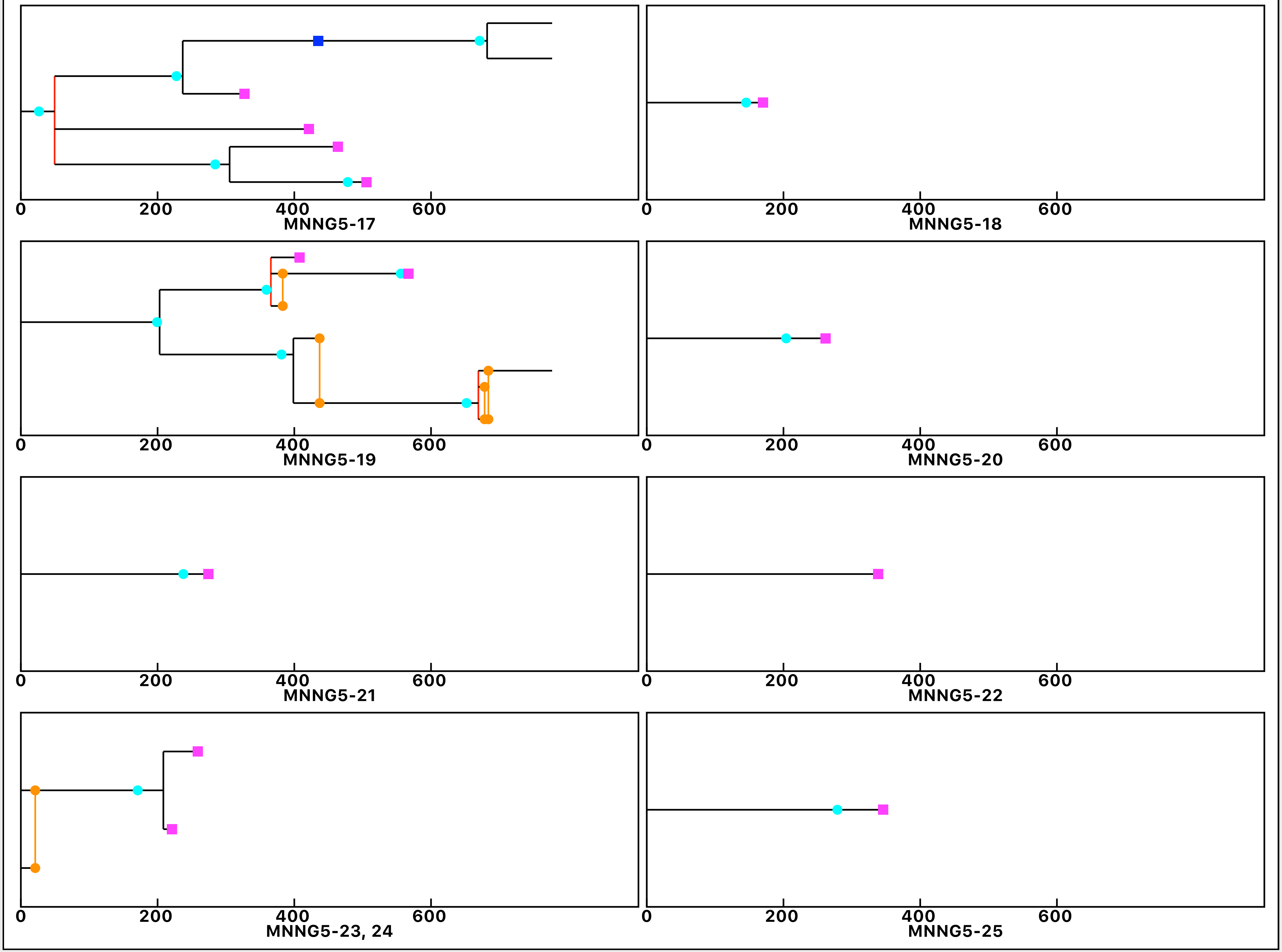

Analysis: MNNG, Treat.: MNNG5, Cell: HeLa

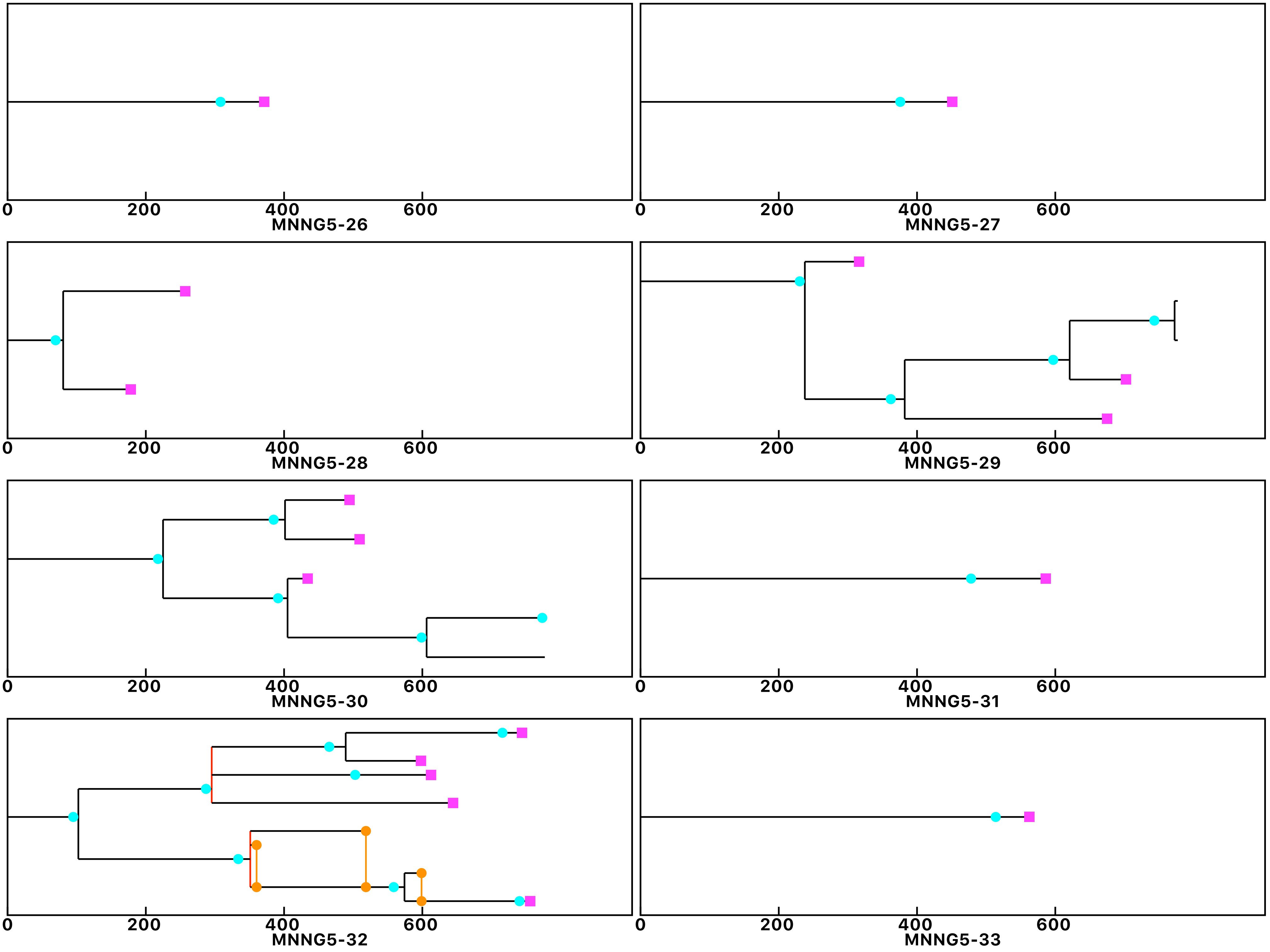

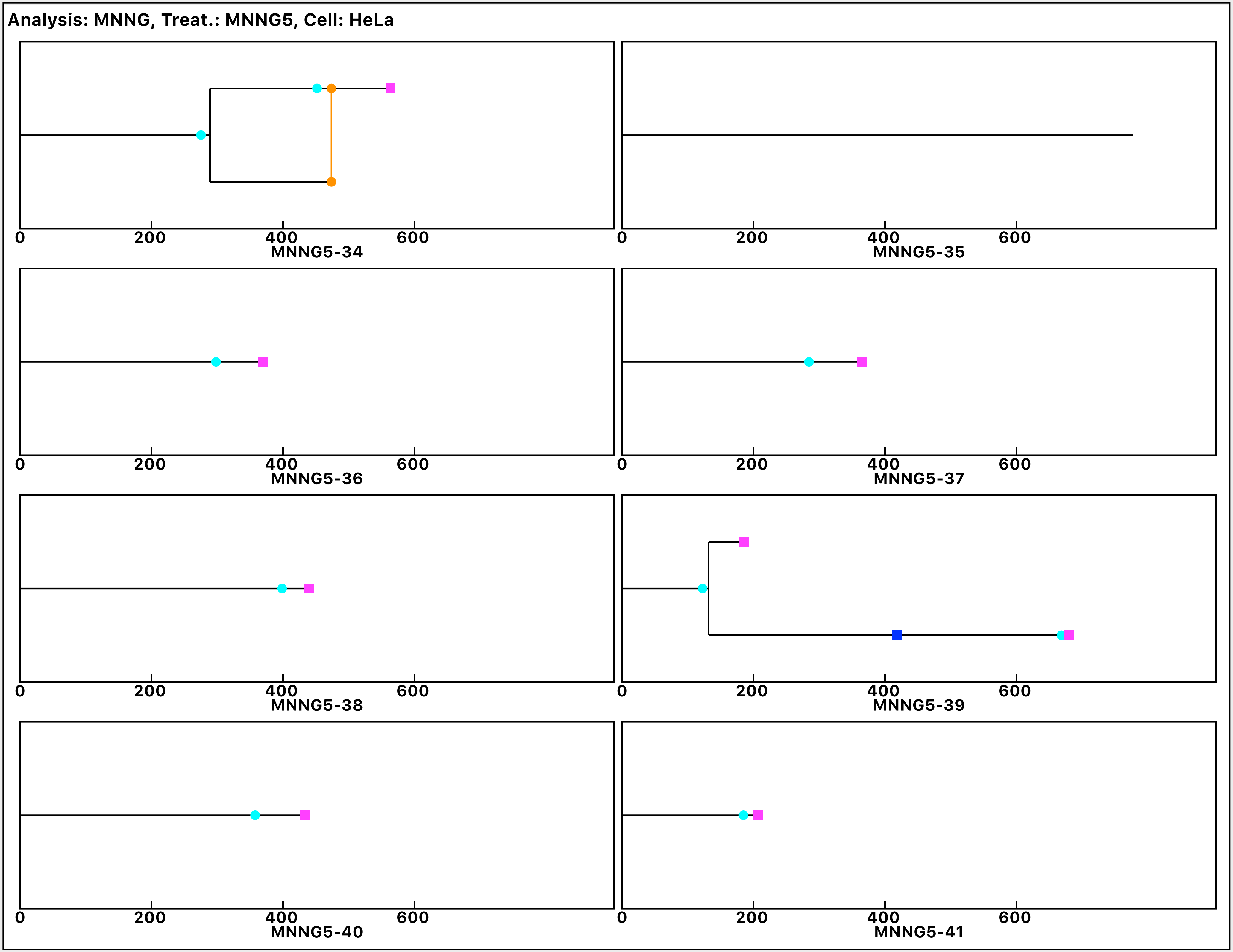
Analysis: MNNG, Treat.: MNNG5, Cell: HeLa
0
200
400
600
MNNG5-34
0
200
400
600
MNNG5-35
0
200
400
600
MNNG5-36
0
200
400
600
MNNG5-37
0
200
400
600
MNNG5-38
0
200
400
600
MNNG5-39
0
200
400
600
MNNG5-40
0
200
400
600
MNNG5-41

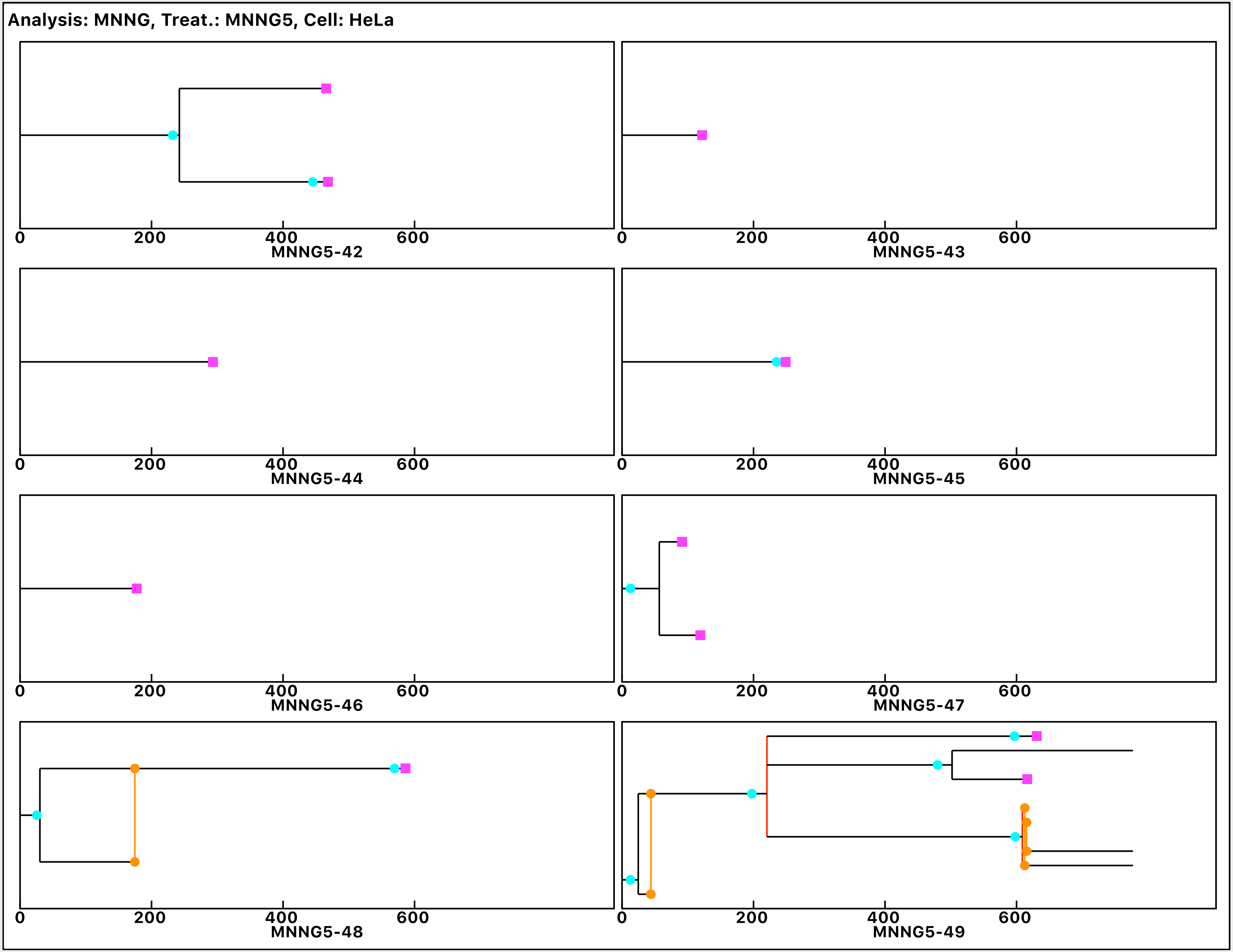
Analysis: MNNG, Treat.: MNNG5, Cell: HeLa
0
200
400
600
MNNG5-42
MNNG5-43
MNNG5-44
MNNG5-45
MNNG5-46
MNNG5-47
MNNG5-48
MNNG5-49

Analysis: MNNG, Treat.: MNNG5, Cell: HeLa

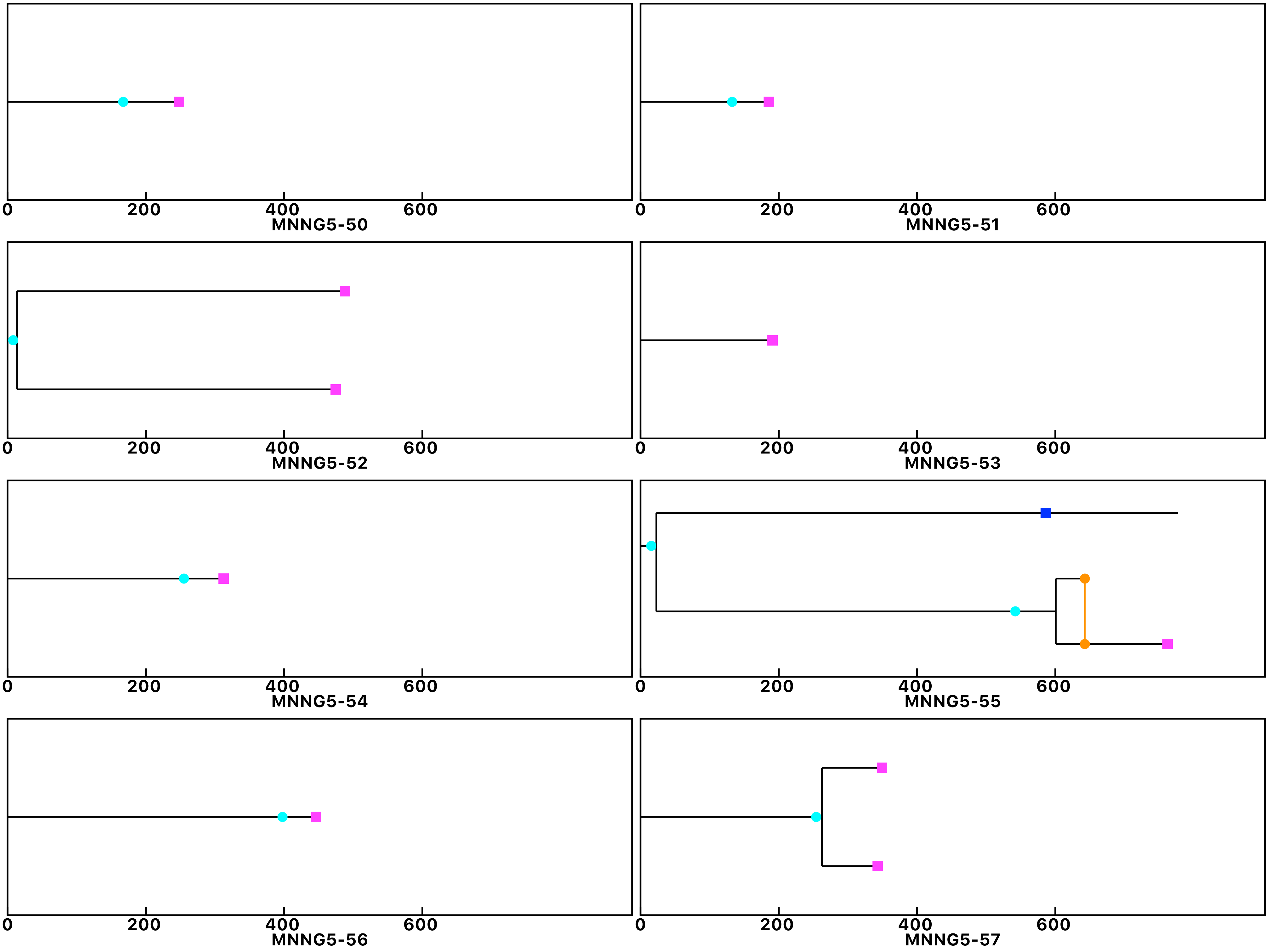

Analysis: MNNG, Treat.: MNNG5, Cell: HeLa

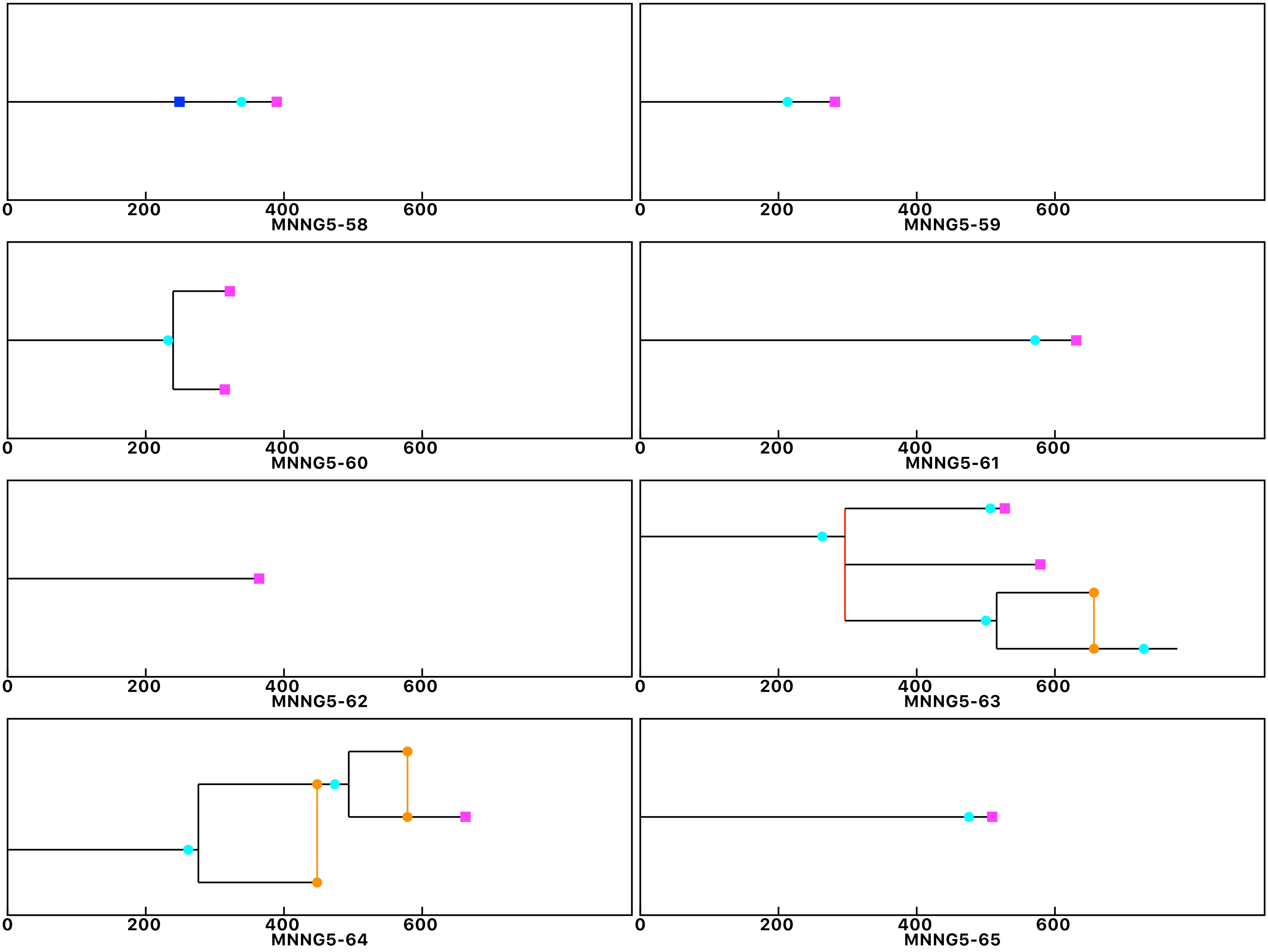

Analysis: MNNG, Treat.: MNNG5, Cell: HeLa

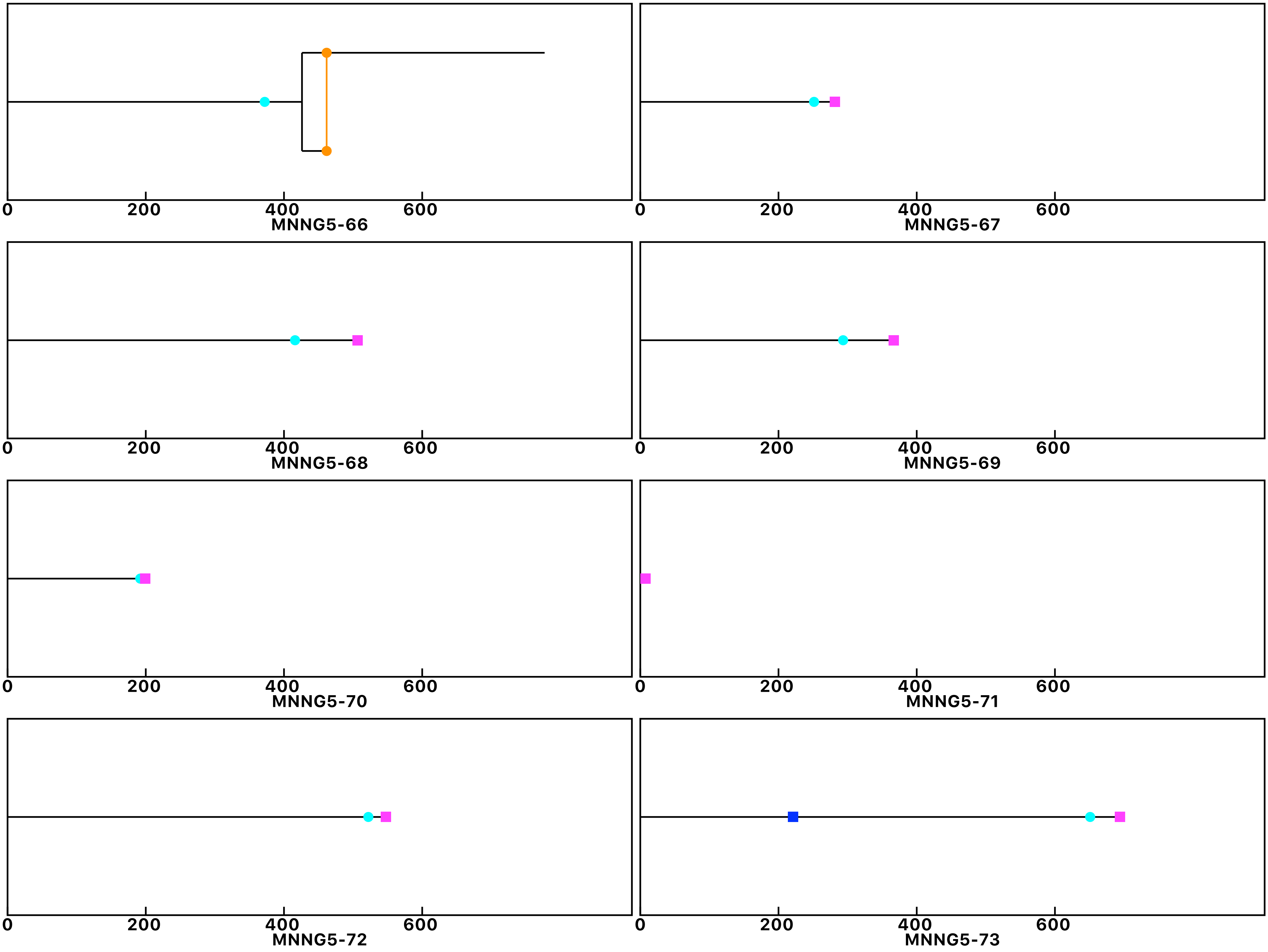

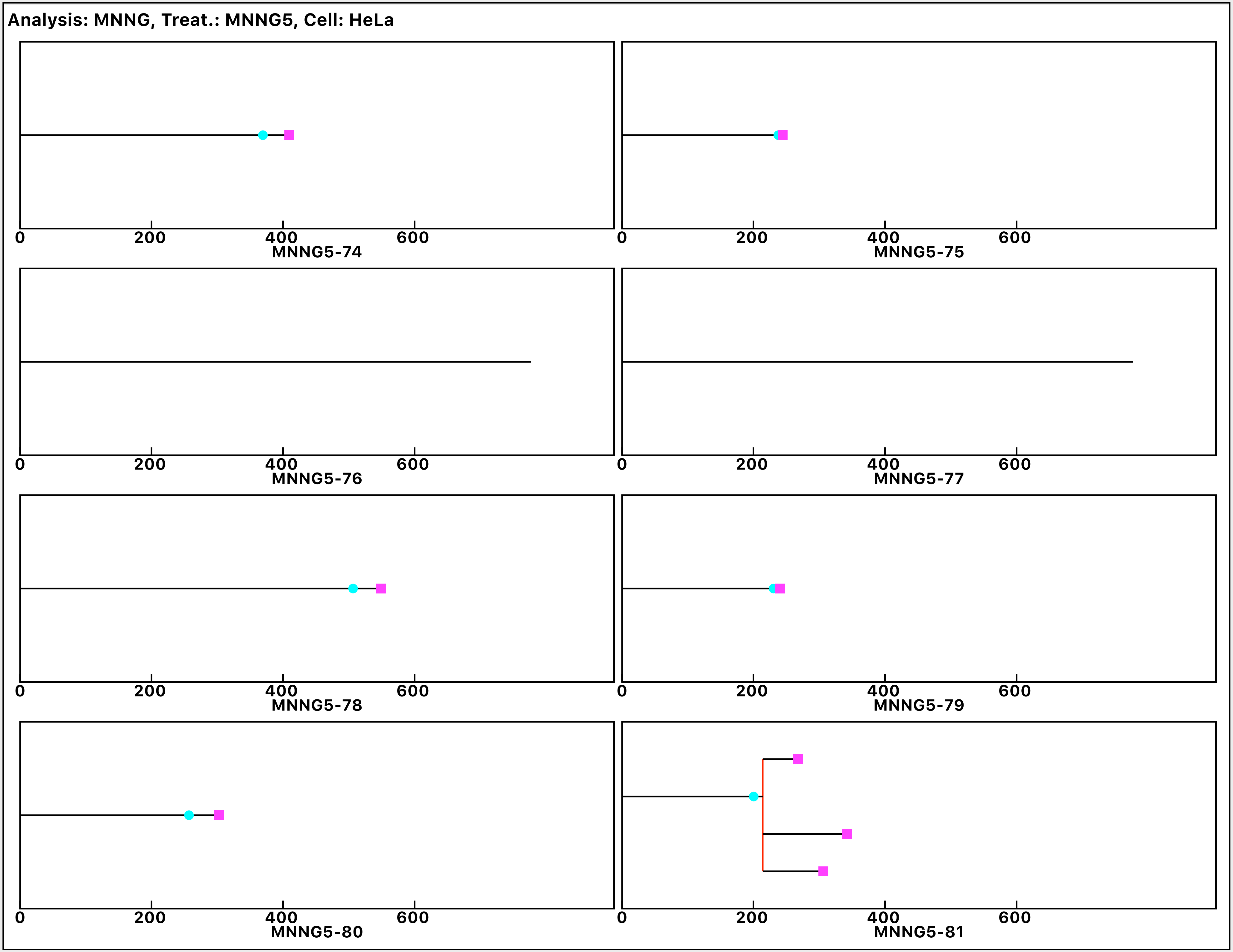
Analysis: MNNG, Treat.: MNNG5, Cell: HeLa
0
200
400
600
MNNG5-74
MNNG5-75
MNNG5-76
MNNG5-77
MNNG5-78
MNNG5-79
MNNG5-80
MNNG5-81

Analysis: MNNG, Treat.: MNNG5, Cell: HeLa

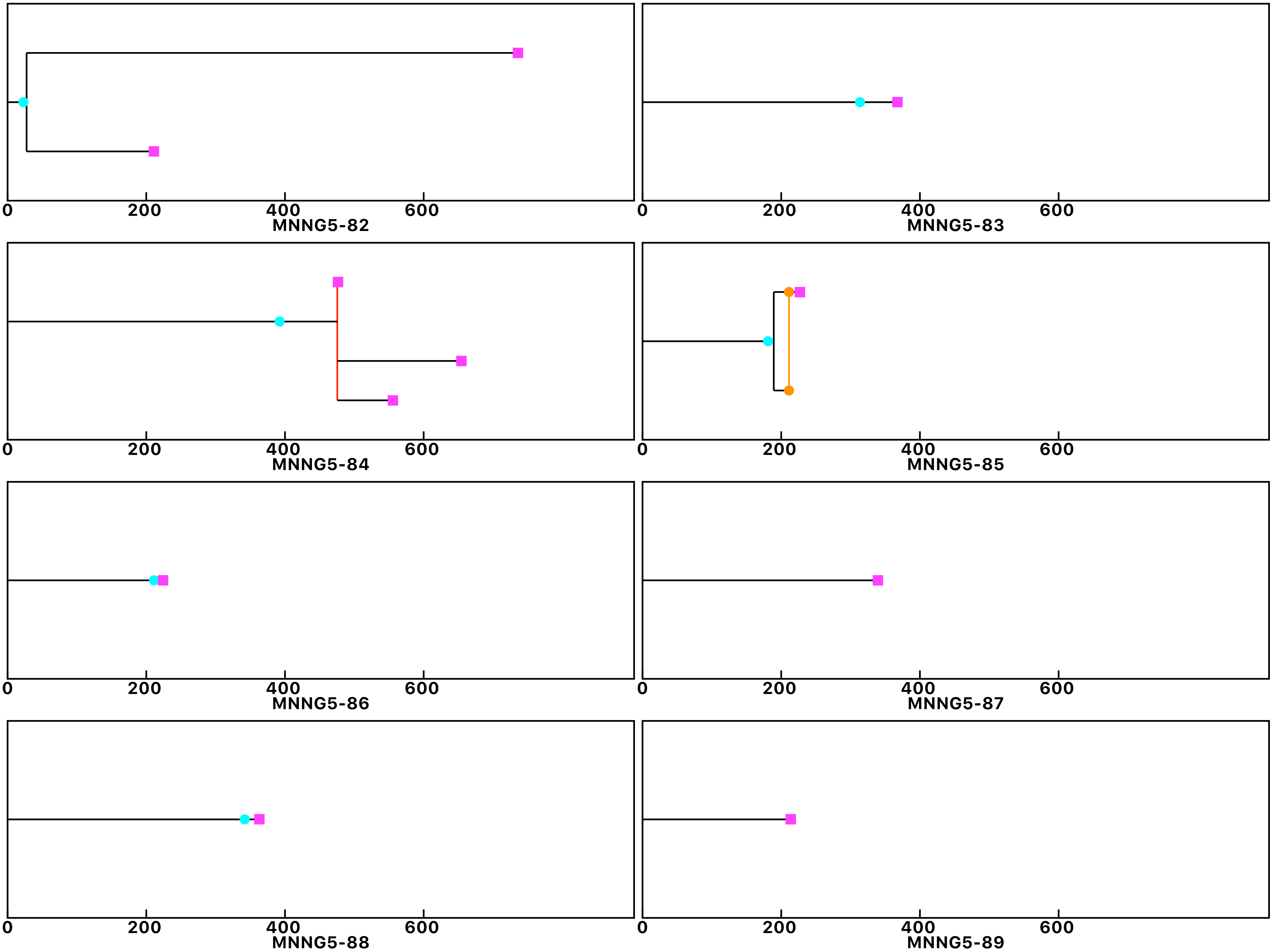

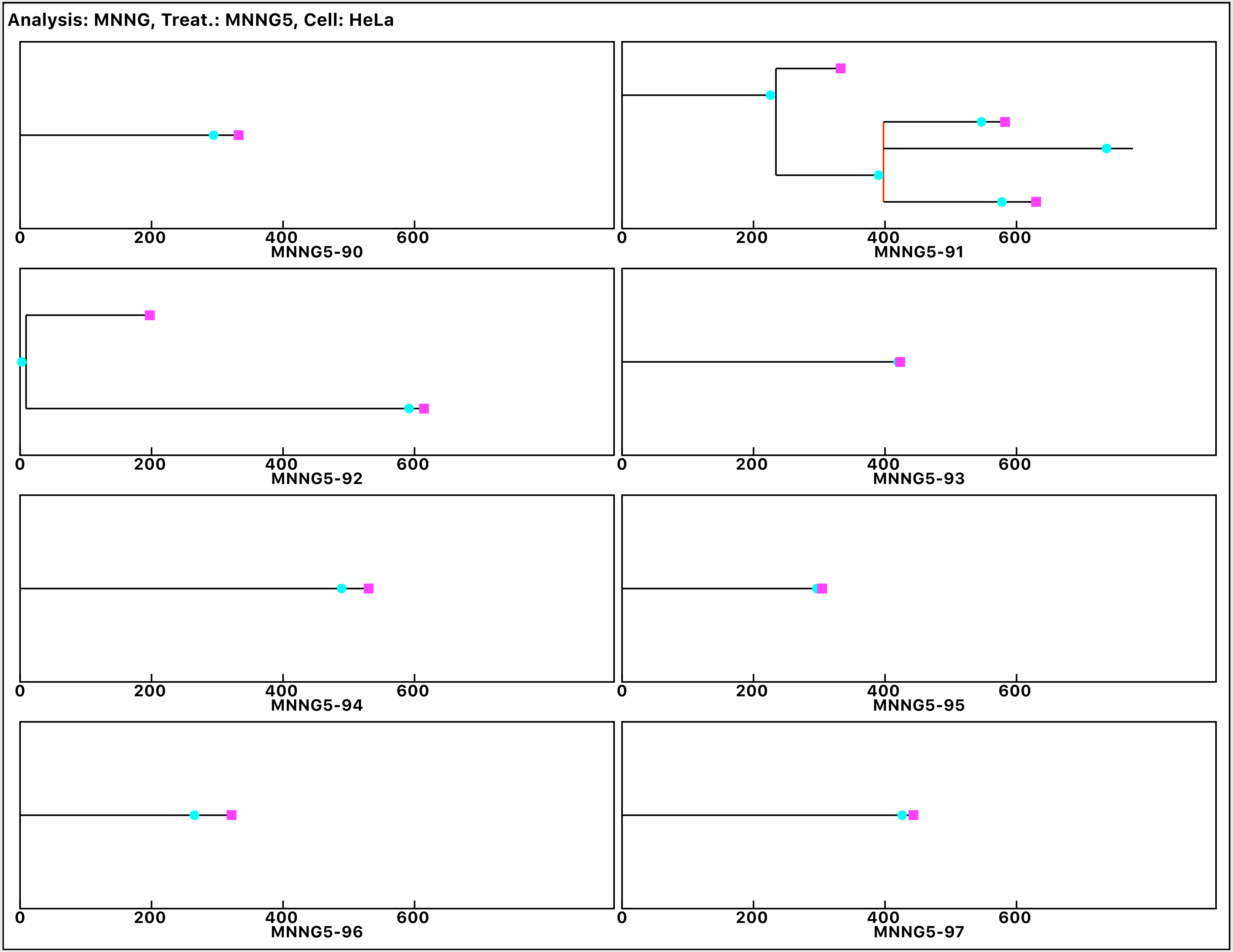
Analysis: MNNG, Treat.: MNNG5, Cell: HeLa
0
200
400
600
MNNG5-90
MNNG5-91
MNNG5-92
MNNG5-93
MNNG5-94
MNNG5-95
MNNG5-96
MNNG5-97

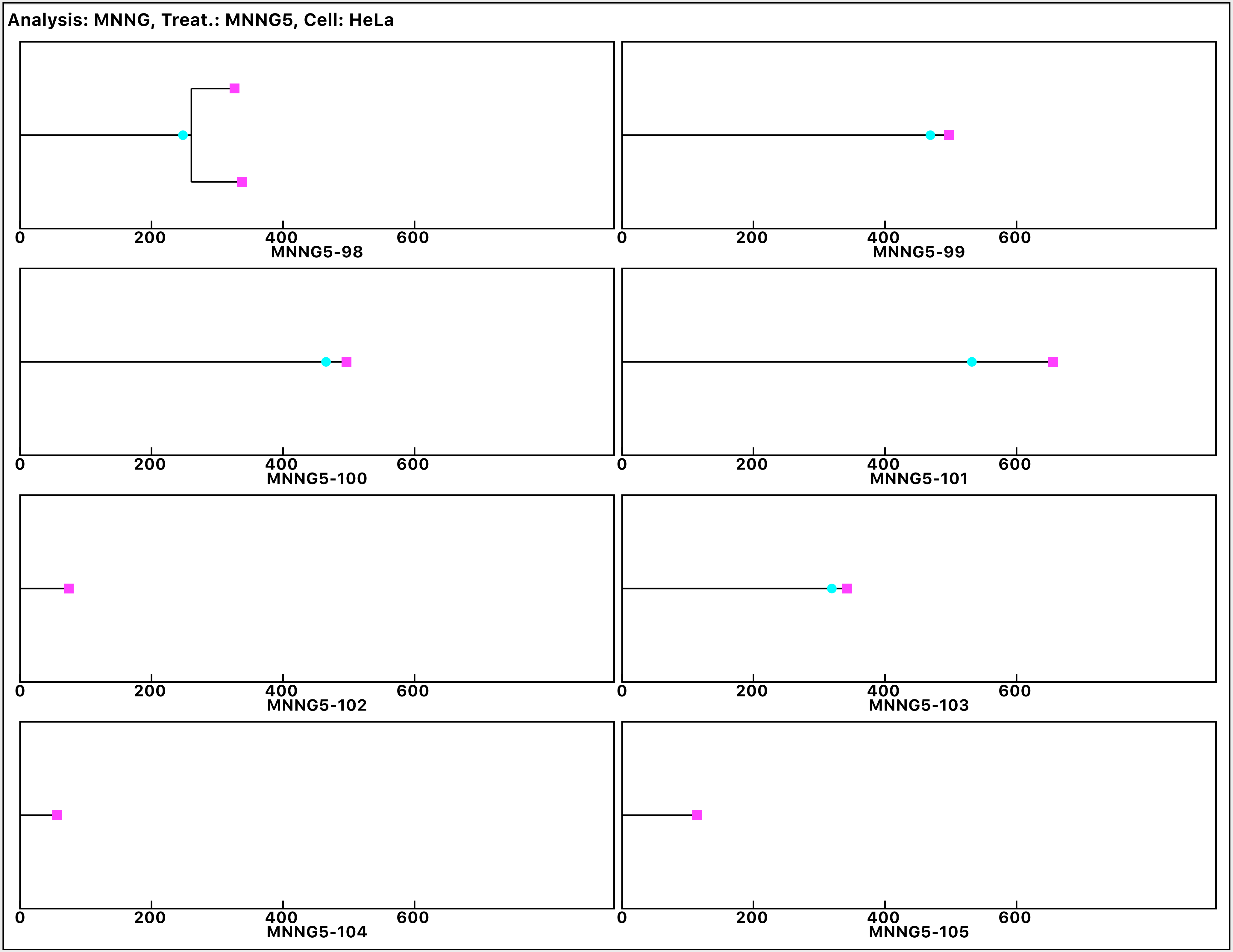
Analysis: MNNG, Treat.: MNNG5, Cell: HeLa
0
200
400
600
MNNG5-98
MNNG5-99
MNNG5-100
MNNG5-101
MNNG5-102
MNNG5-103
MNNG5-104
MNNG5-105

Analysis: MNNG, Treat.: MNNG5, Cell: HeLa

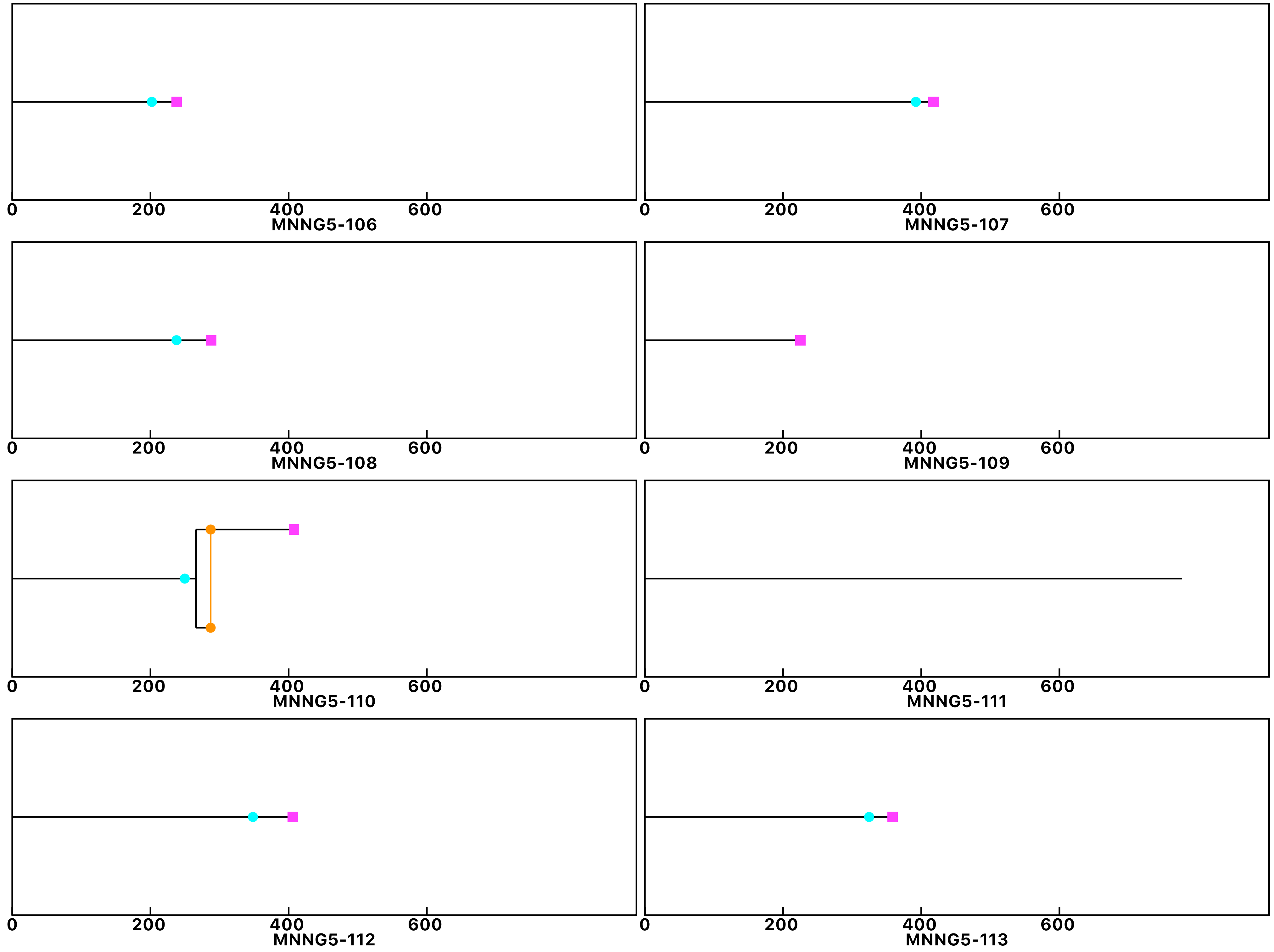

Analysis: MNNG, Treat.: MNNG5, Cell: HeLa

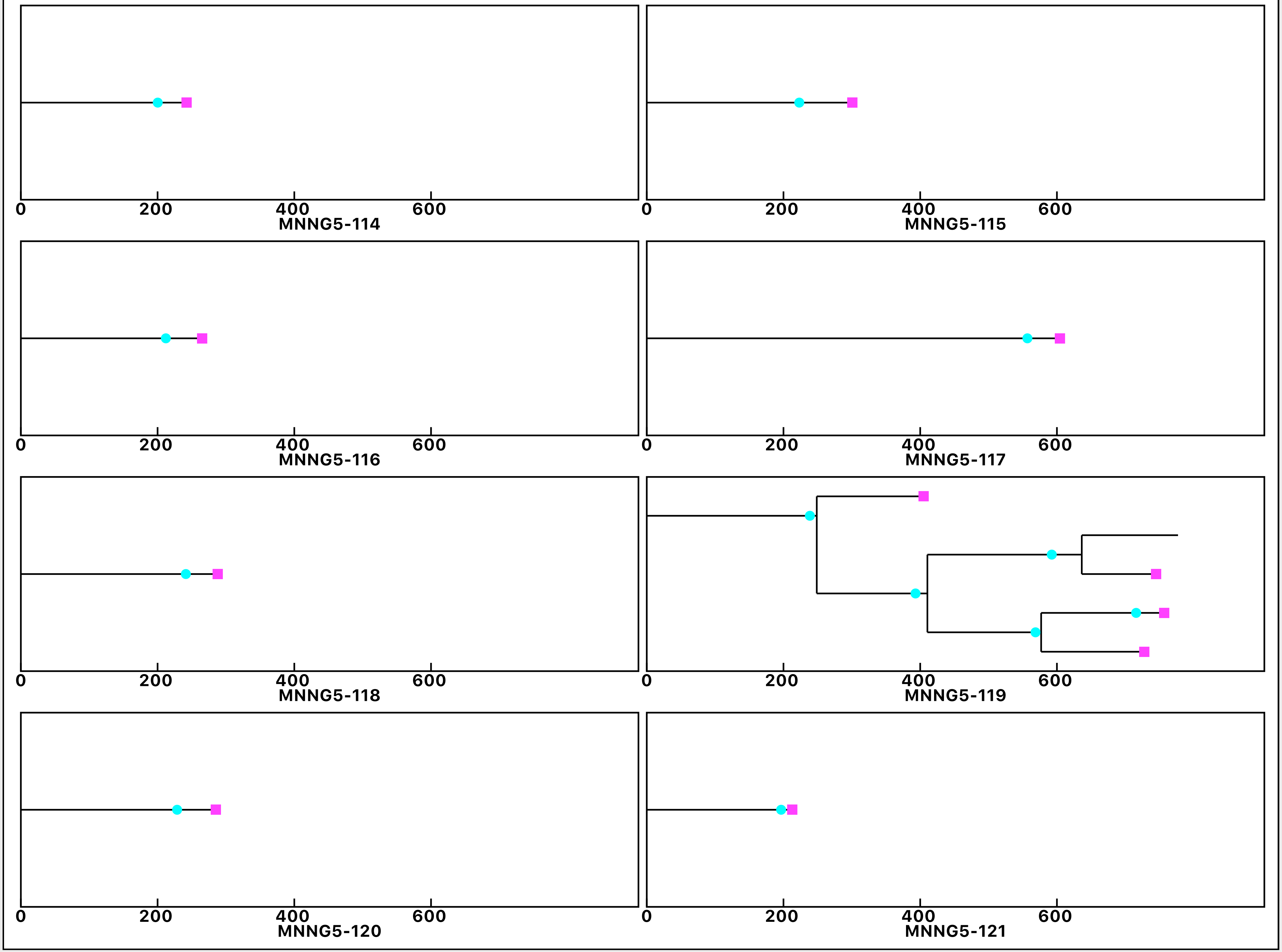

Analysis: MNNG, Treat.: MNNG5, Cell: HeLa

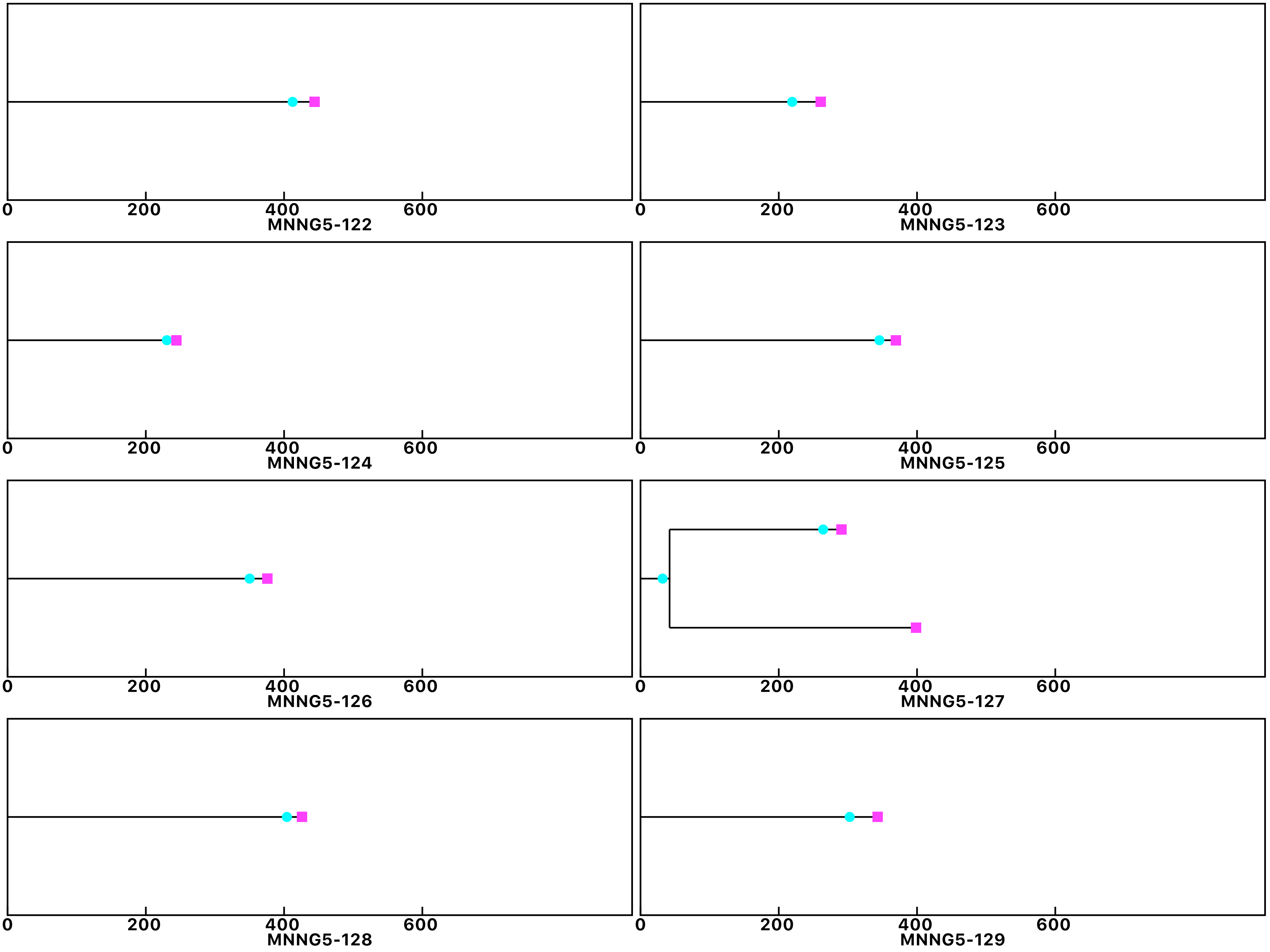

Analysis: MNNG, Treat.: MNNG5, Cell: HeLa

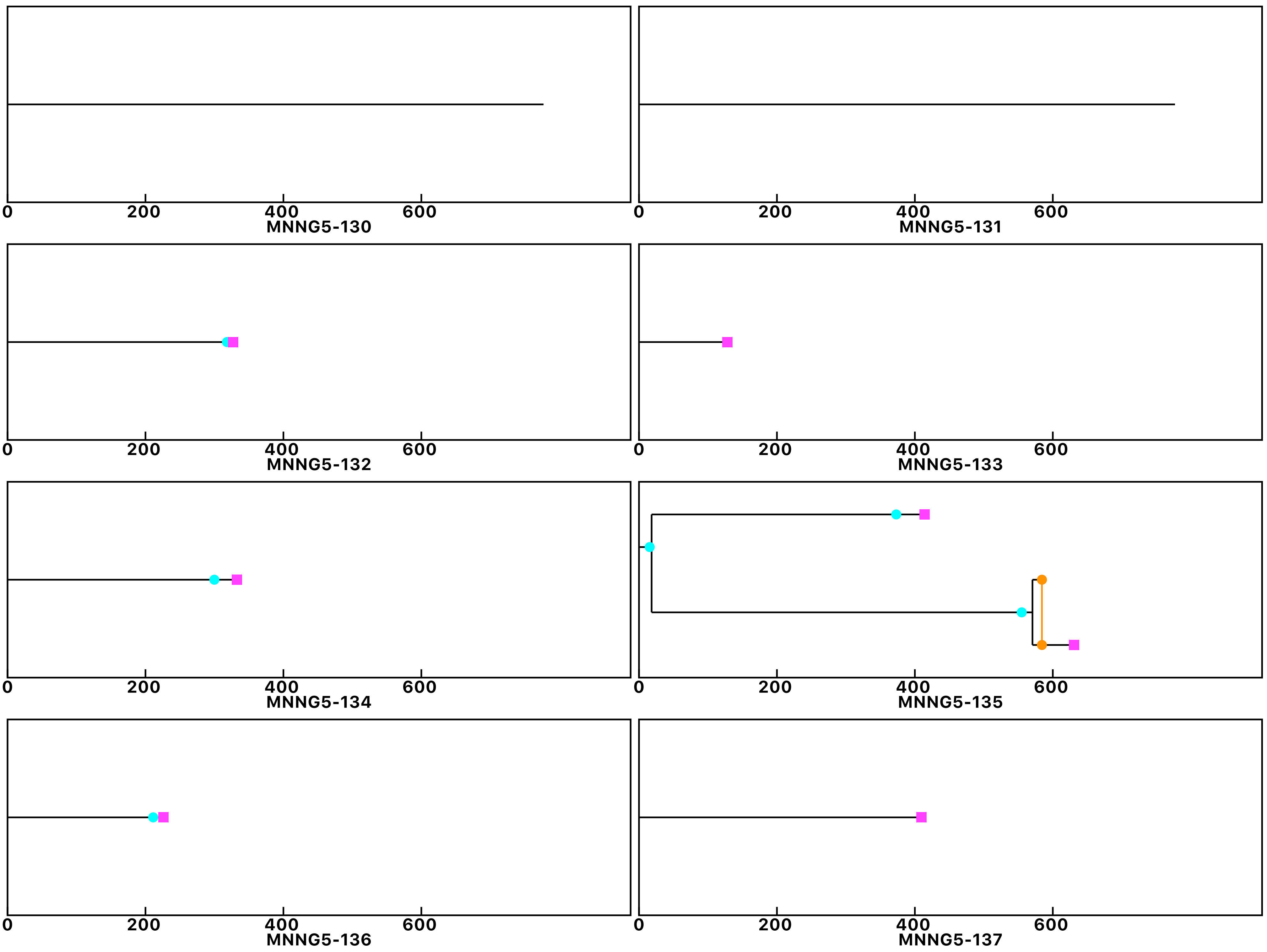

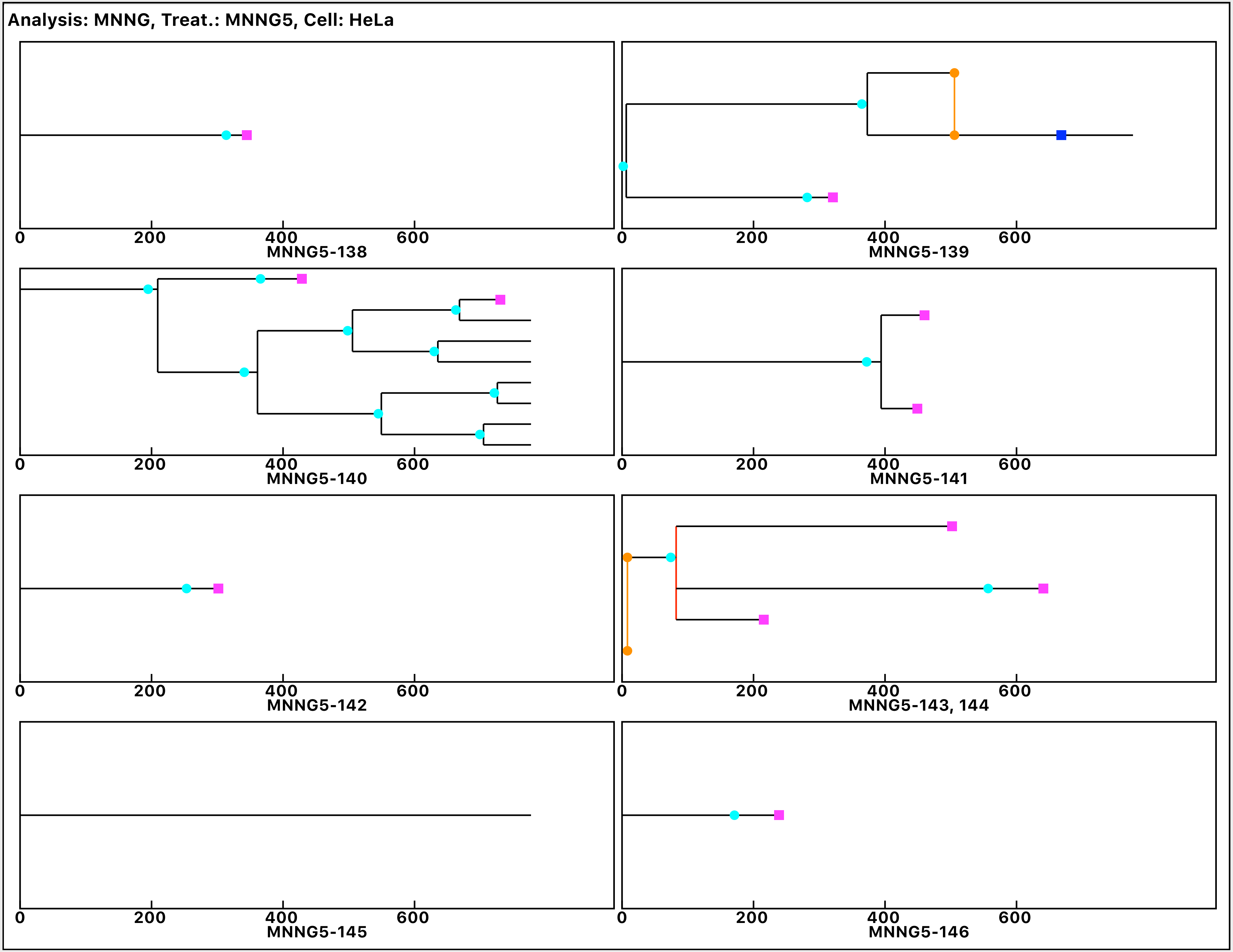
Analysis: MNNG, Treat.: MNNG5, Cell: HeLa
0
200
400
600
MNNG5-138
MNNG5-139
MNNG5-140
MNNG5-141
MNNG5-142
MNNG5-143, 144
MNNG5-145
MNNG5-146

Analysis: MNNG, Treat.: MNNG5, Cell: HeLa

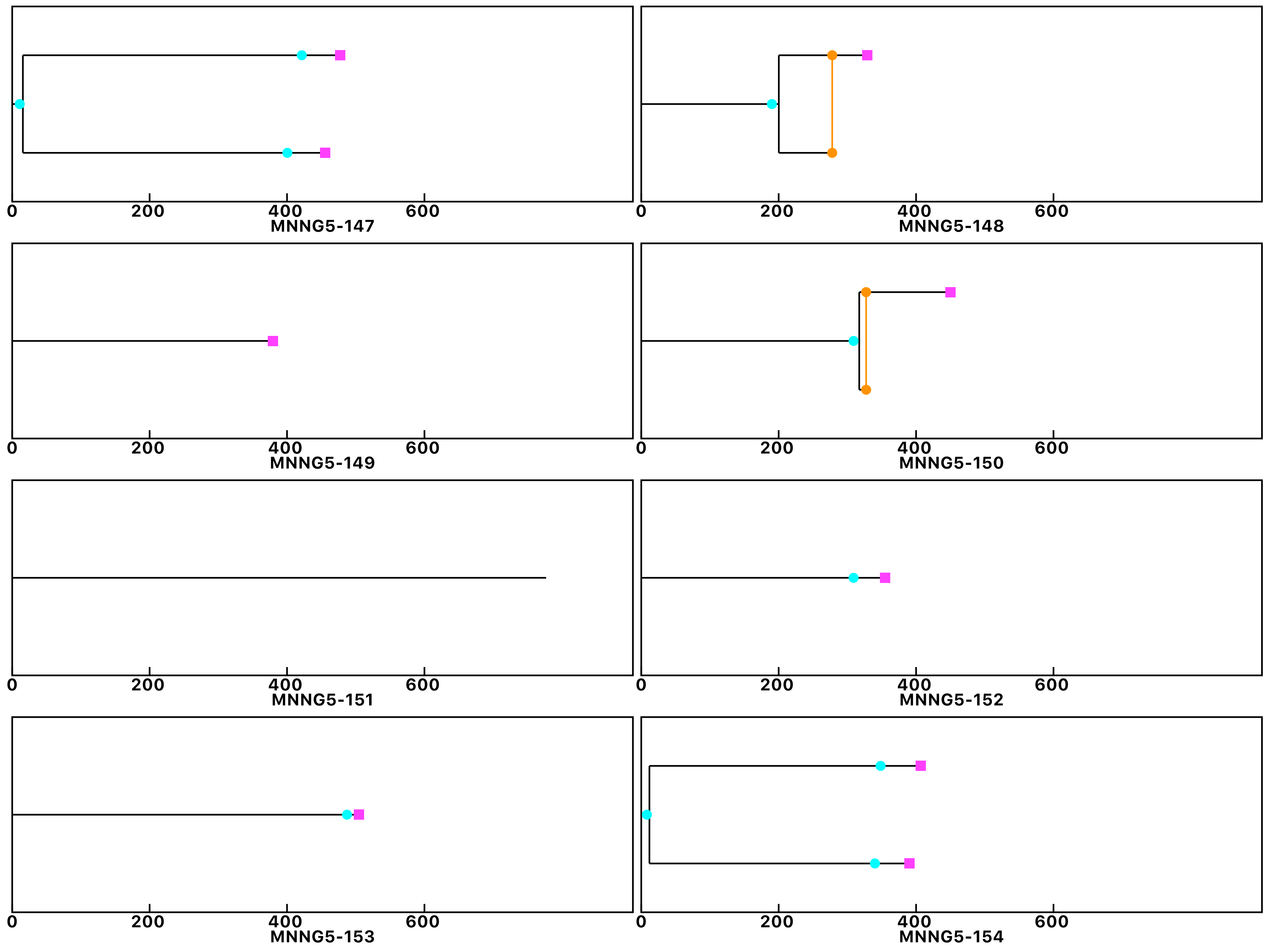

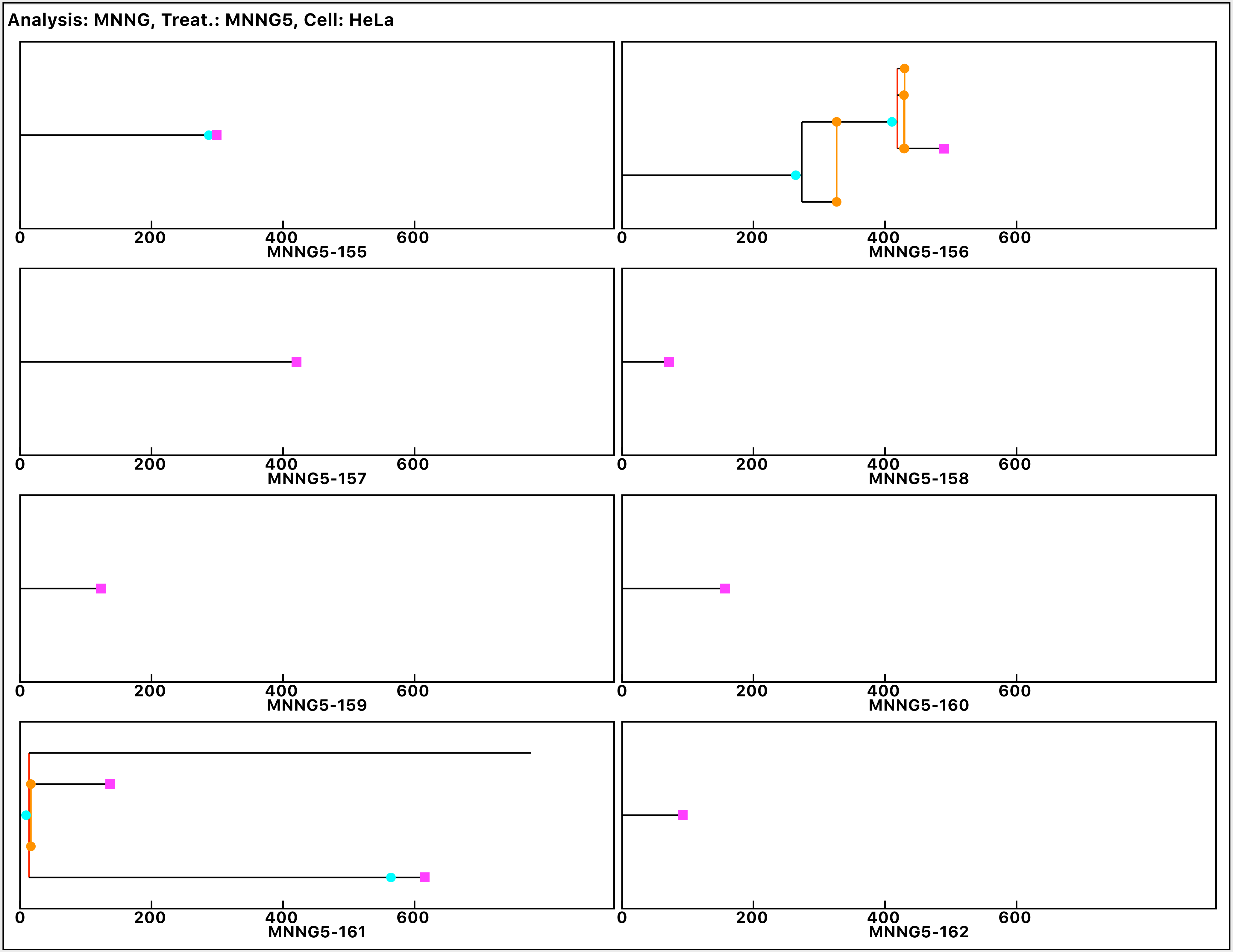
Analysis: MNNG, Treat.: MNNG5, Cell: HeLa
0
200
400
600
MNNG5-155
MNNG5-156
MNNG5-157
MNNG5-158
MNNG5-159
MNNG5-160
MNNG5-161
MNNG5-162

Analysis: MNNG, Treat.: MNNG5, Cell: HeLa

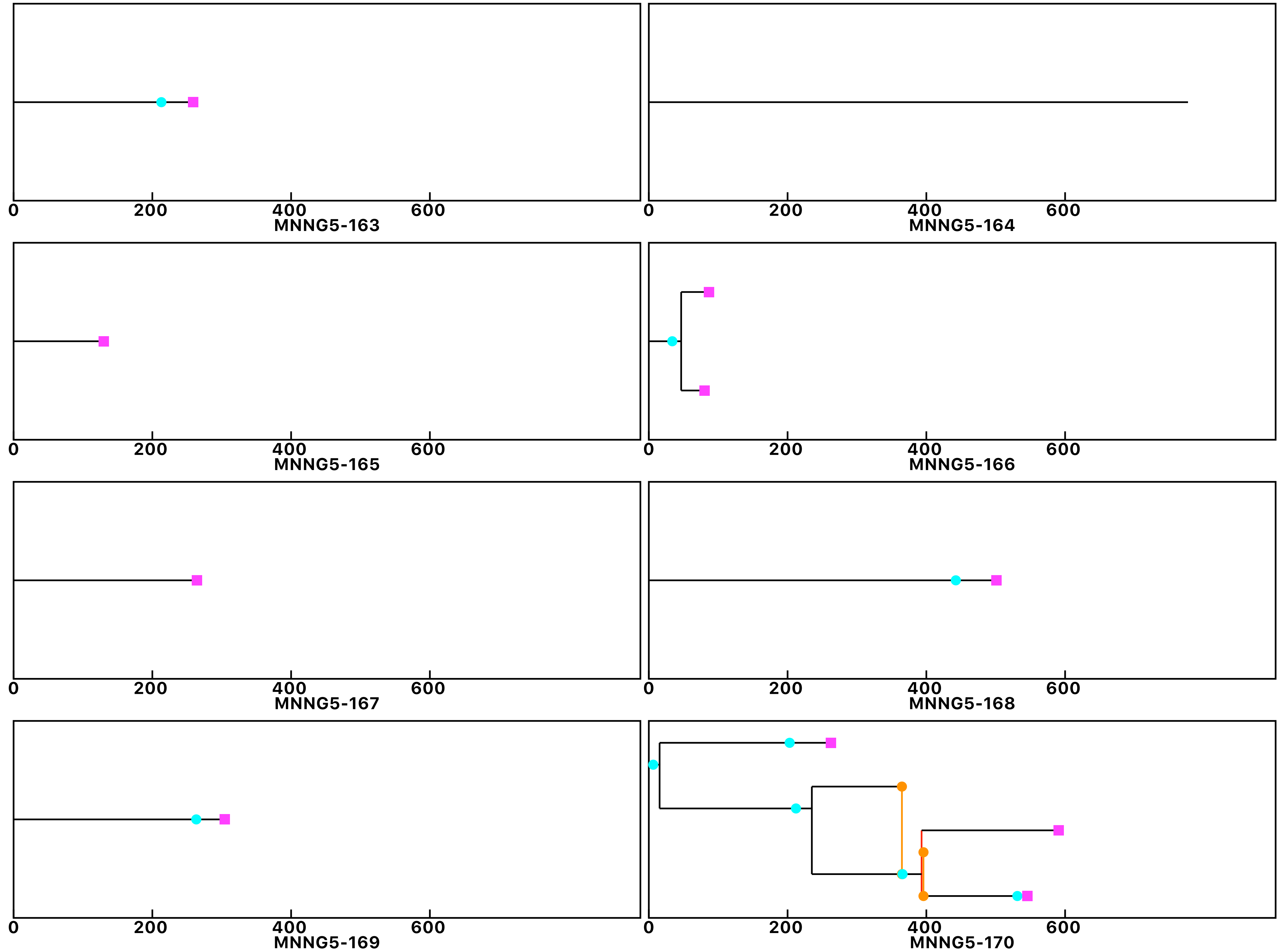

Analysis: MNNG, Treat.: MNNG5, Cell: HeLa

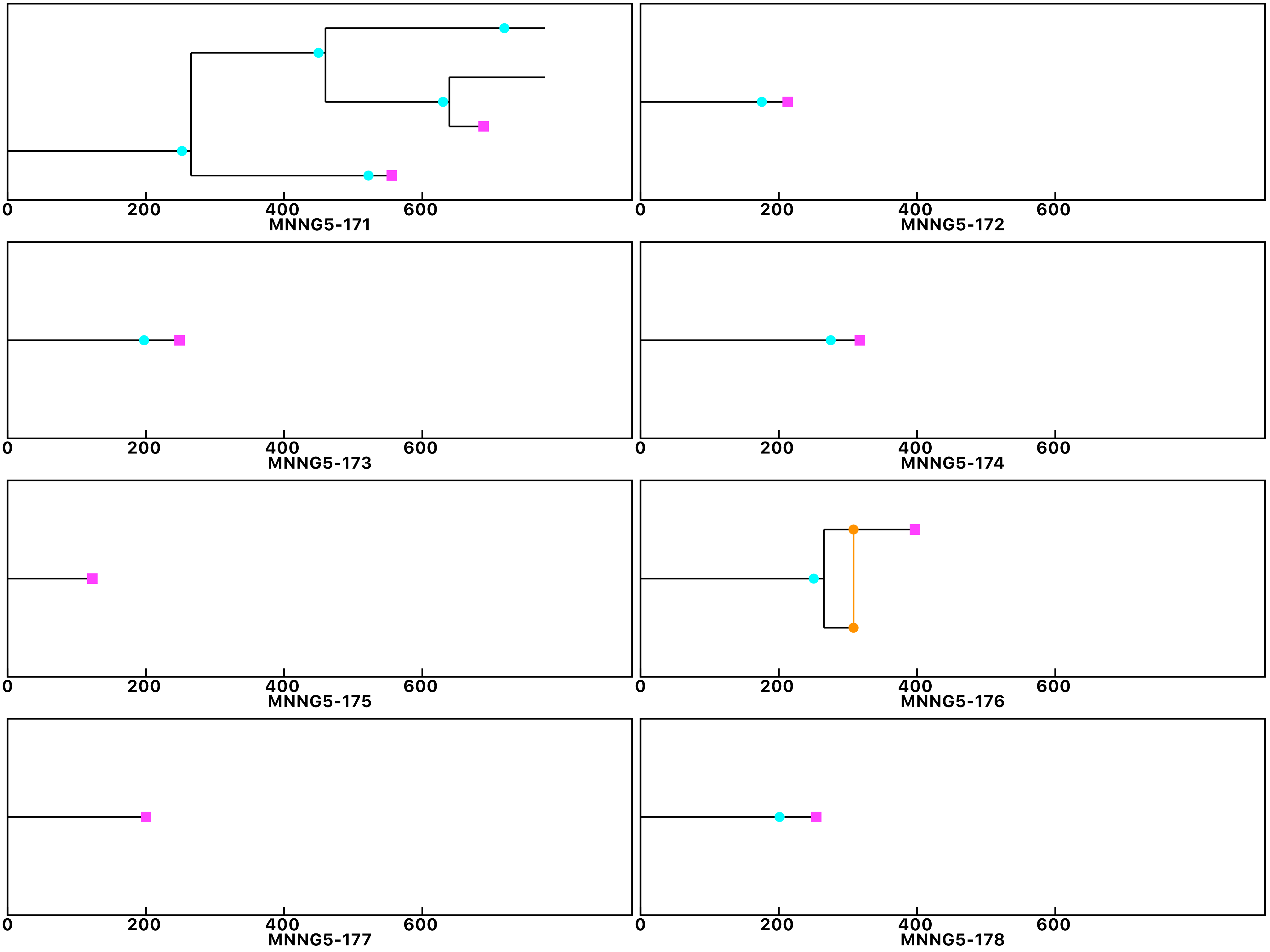

Analysis: MNNG, Treat.: MNNG5, Cell: HeLa

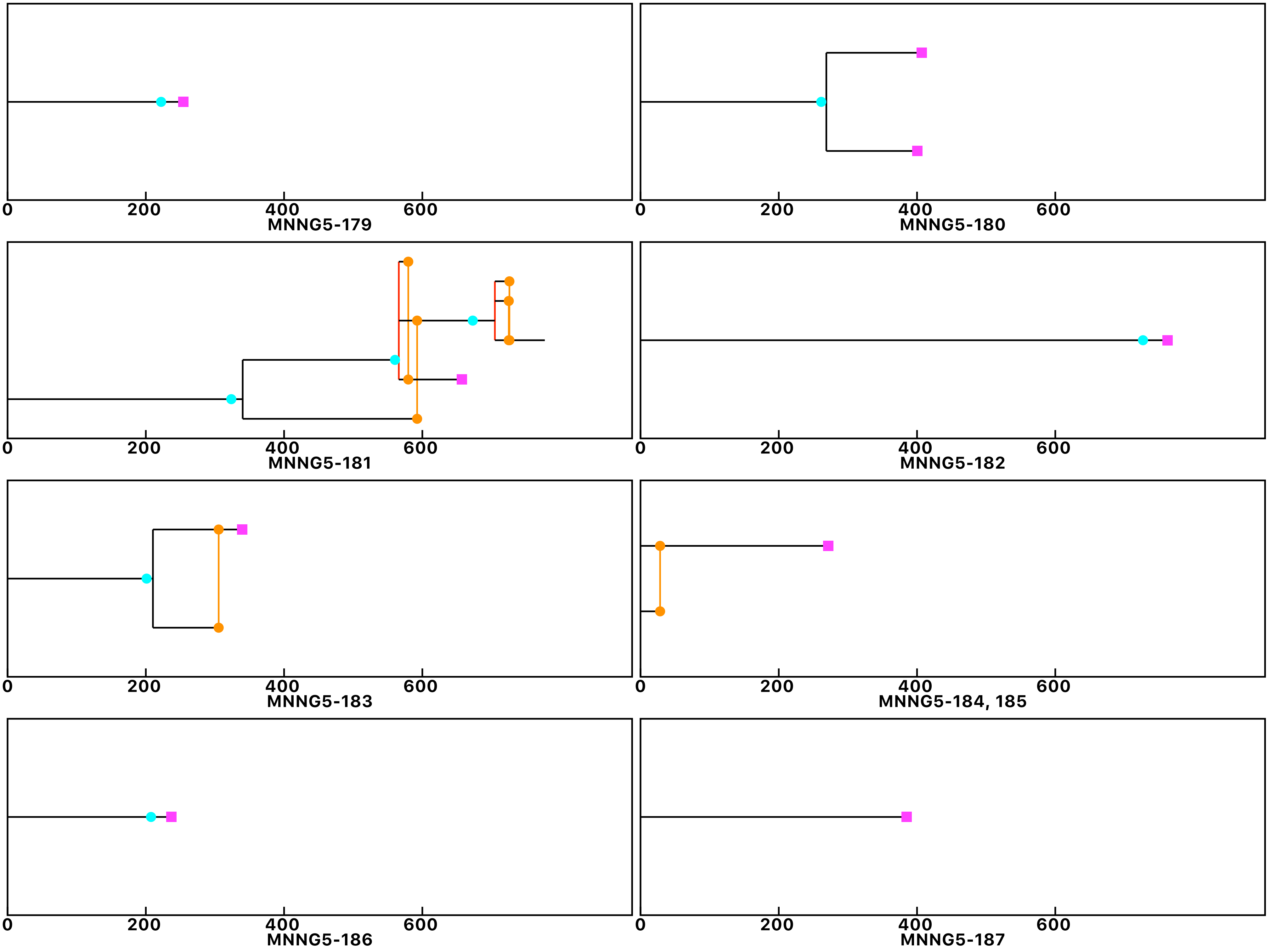

Analysis: MNNG, Treat.: MNNG5, Cell: HeLa

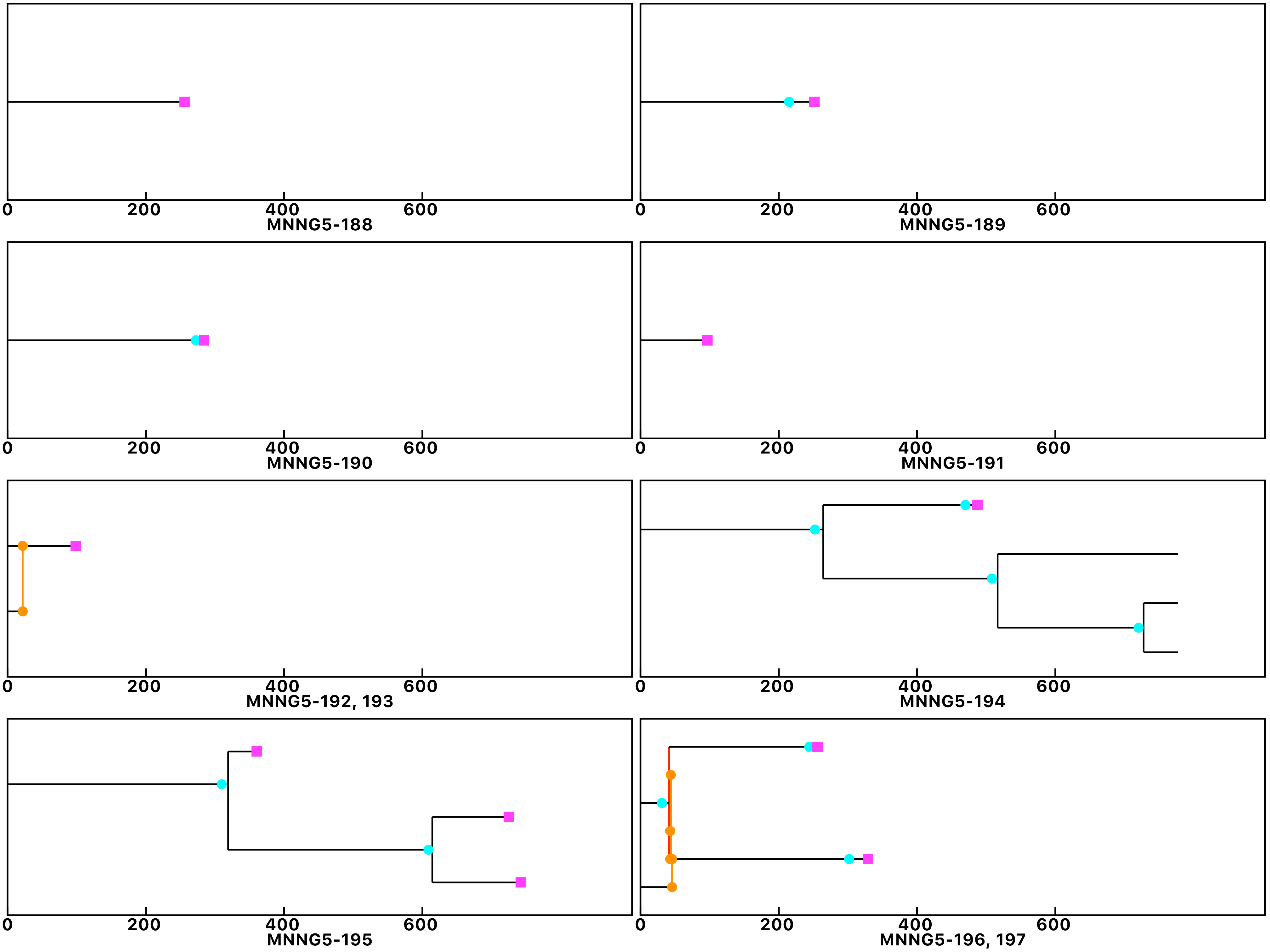

Analysis: MNNG, Treat.: MNNG5, Cell: HeLa

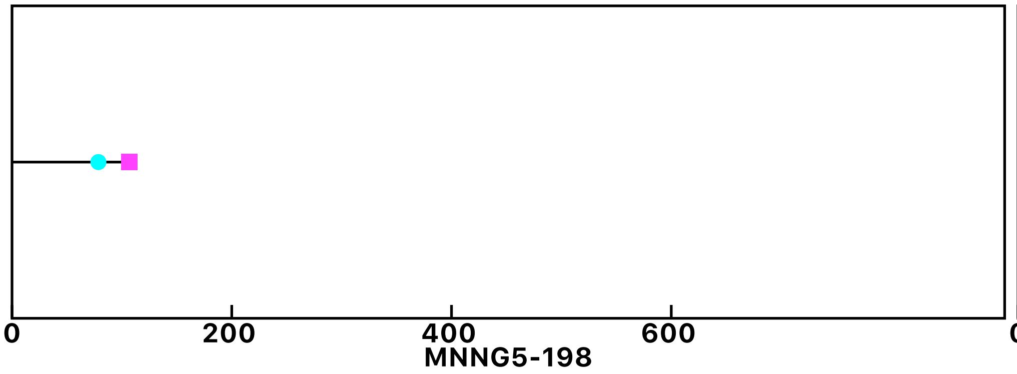

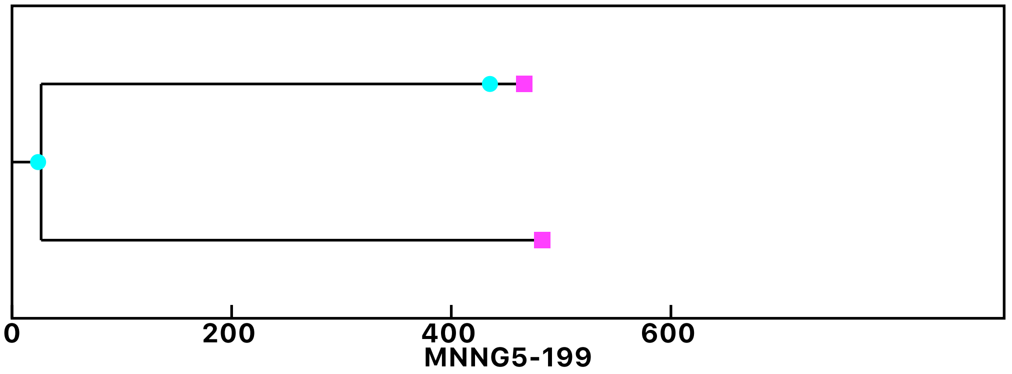

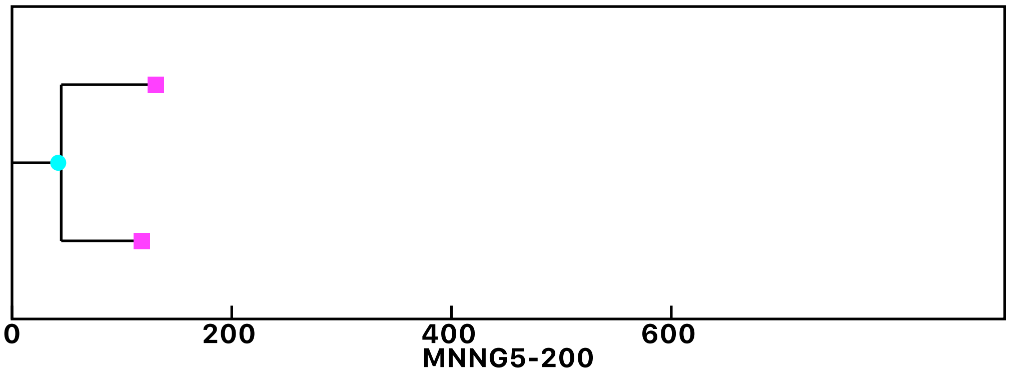

Supplement: S4 Fig — Cell lineage maps (MNNG-5μM:MNNG5) are shown. Light blue circle: mitosis, orange circle: cell fusion, pink square: cell death, blue square: incomplete cell division, black vertical line: bipolar cell division, red vertical line: multipolar cell division, and orange vertical line: cell fusion. (PDF) [file pone.0214512.s004.pdf]
