# Supplementary figures and images for: Dose-dependent spatiotemporal responses of mammalian cells to an alkylating agent
Source: PLoS One. 2019 Mar 29;14(3):e0214512. doi: 10.1371/journal.pone.0214512 (PMC6440626; doi:10.1371/journal.pone.0214512)

Supplementary Figure 5

A

Long-term live cell imaging video

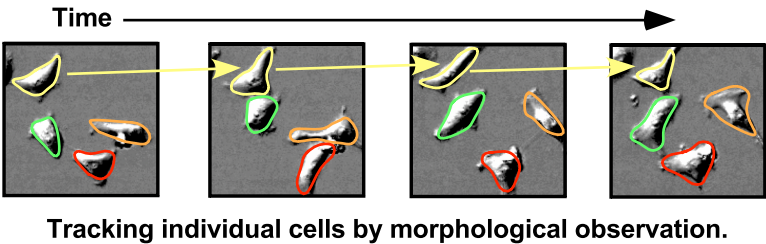

B

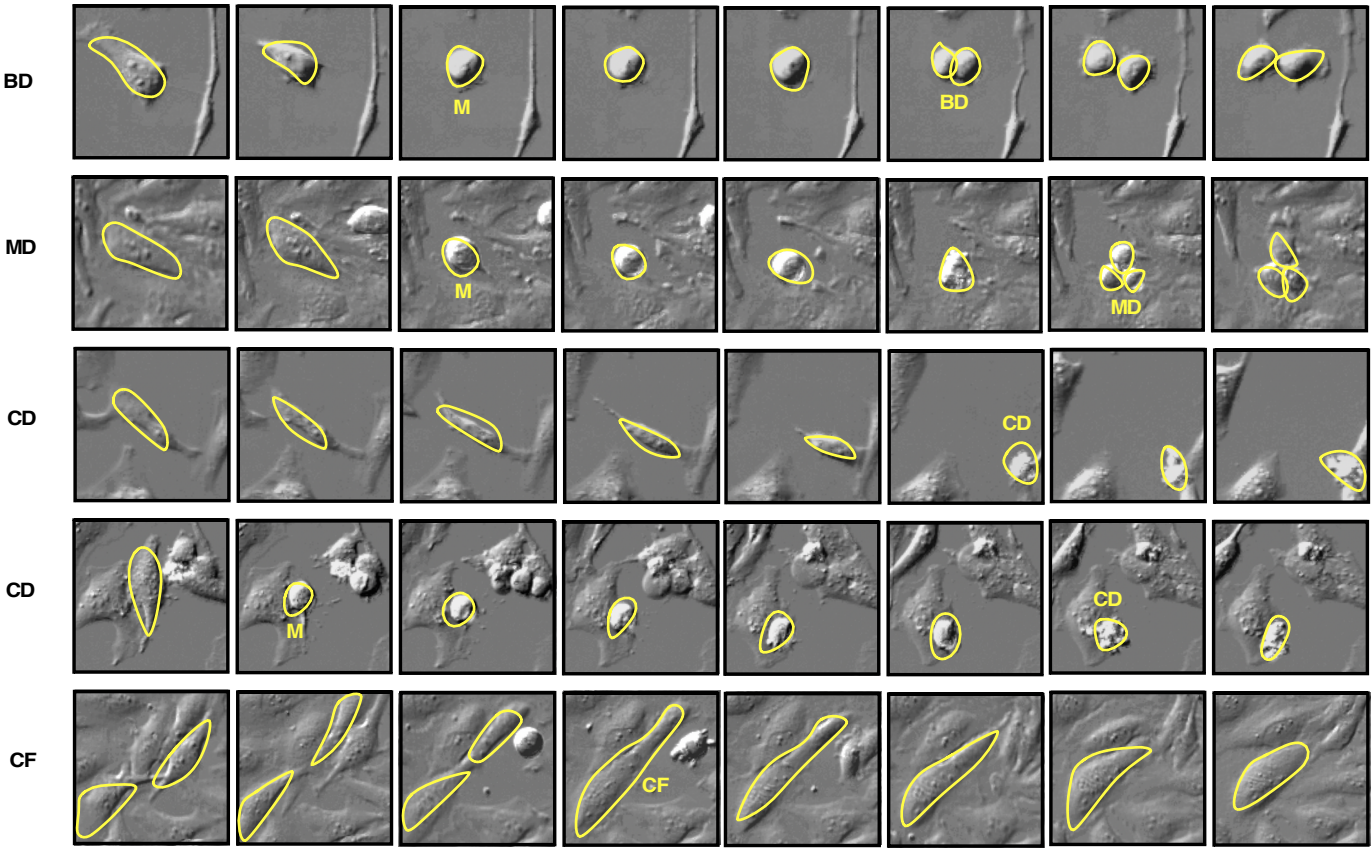

Supplement: S5 Fig — A. Individual cells recorded in a live cell imaging video were identified (represented by different color of circles) and tracked visually as indicated by arrows. B. List of categorized cellular events, M, BD, MD, CF, and CD, are shown. Tripolar cell division is shown as an example of MD. (PDF) [file pone.0214512.s005.pdf]

Supplementary Figure 7

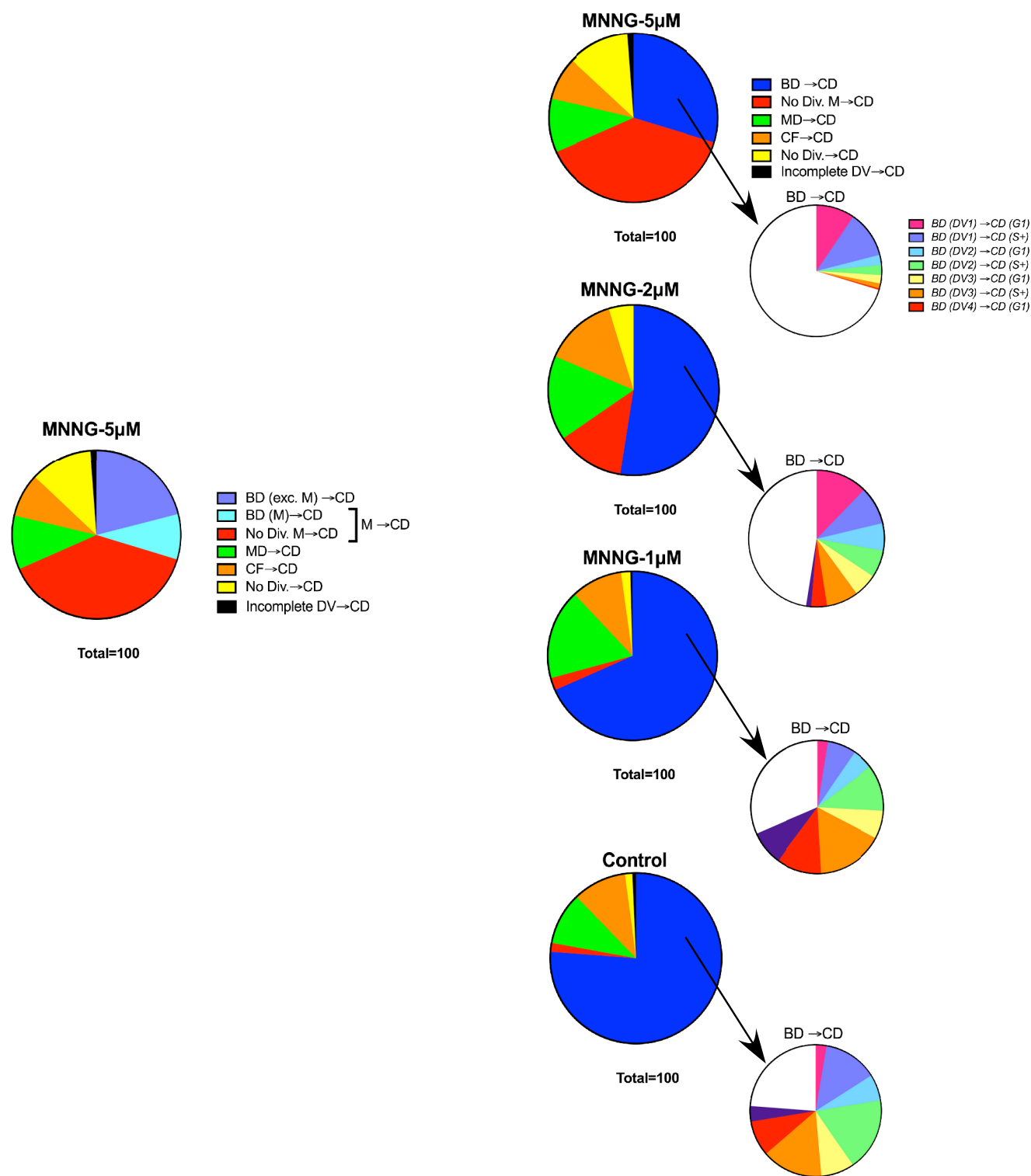

Supplement: S7 Fig — Results shown in Table 1 are illustrated schematically using pie charts. The left side of the pie chart corresponds to data shown in Table 1 (MNNG-5μM). The right side of the pie charts (MNNG-2μM, MNNG-1μM and Control) correspond to data shown in Table 1 (MNNG-2μM, MNNG-1μM and Control). The right side of the pie chart (MNNG-5μM) was created using the same categorization that was used for MNNG-2μM, MNNG-1μM and Control. (PDF) [file pone.0214512.s007.pdf]

Supplementary Figure 8

A

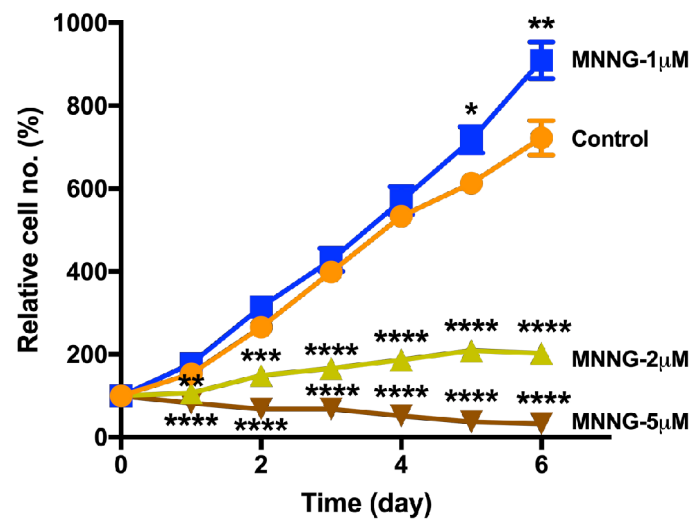

B

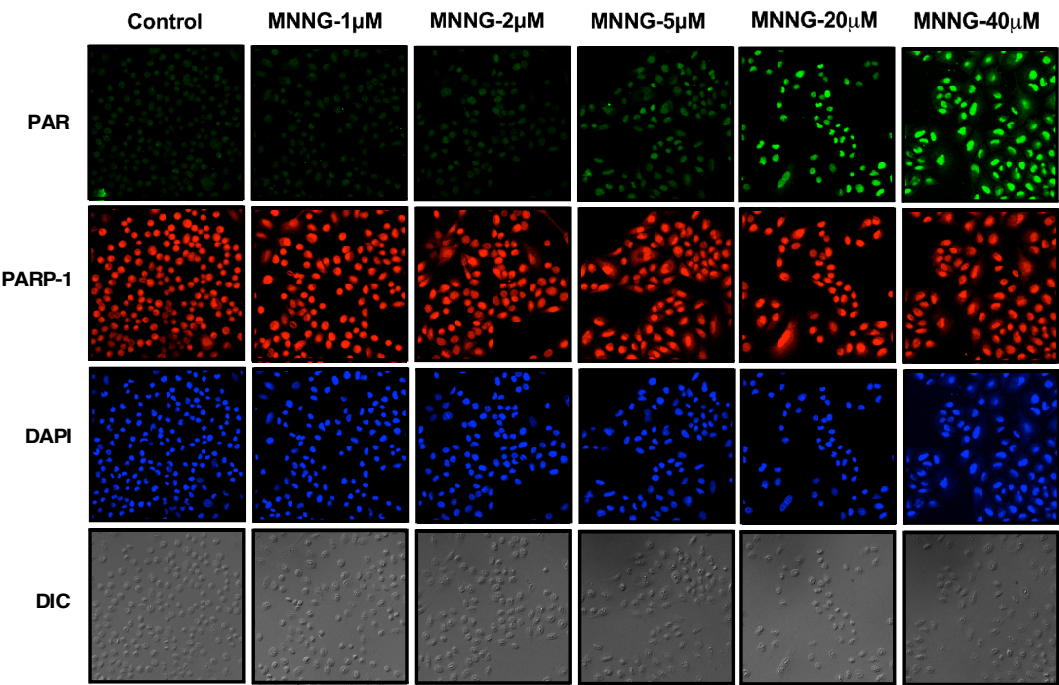

Supplement: S8 Fig — A. Numbers of cells were determined every 24 h (n = 6). The initial numbers of cells were normalized by 100. One-way ANOVA (Tukey’s multiple comparison test) was performed for each time point. The significance of differences between Control and MNNG-1μM, MNNG-2μM, and MNNG-5μM are shown: *p<0.05, **p<0.01, ***p<0.001, and ****p<0.0001. Results shown as the mean ± SEM. B. After exposure of cells to various doses of MNNG for 30 min, indirect immunofluorescence was performed using anti-poly(ADP-ribose) polymerase-1 (PARP-1) antibody and anti-ADP-ribose polymer (PAR) antibody. Cells were also stained with DAPI. (PDF) [file pone.0214512.s008.pdf]
