# Supplementary material for: Dose-dependent spatiotemporal responses of mammalian cells to an alkylating agent
Source: PLoS One. 2019 Mar 29;14(3):e0214512. doi: 10.1371/journal.pone.0214512 (PMC6440626; doi:10.1371/journal.pone.0214512)
Supplement: S6 Fig — Schematic illustrations of cellular events, i.e. BD, CF, CD and MD, are shown in the upper panel. The patterns of cell death induction listed in Table 1 are shown in the lower panel. (PDF) [file pone.0214512.s006.pdf]

Supplementary Figure 6

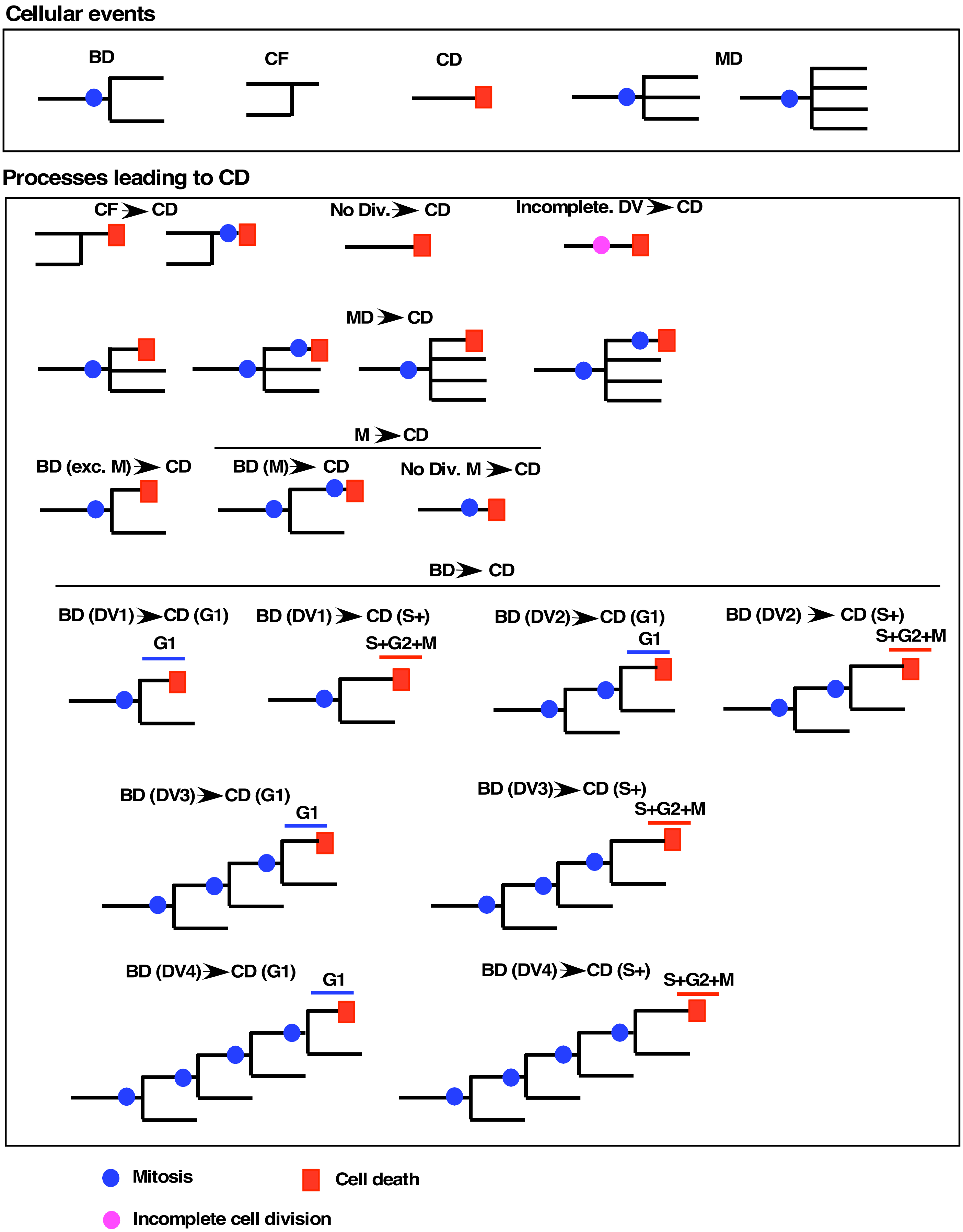

Rancourt et al.
